# γ-Amino Alcohols via Energy Transfer Enabled Brook Rearrangement

**DOI:** 10.1021/jacs.4c01667

**Published:** 2024-04-03

**Authors:** Ranjini Laskar, Subhabrata Dutta, Jan C. Spies, Poulami Mukherjee, Ángel Rentería-Gómez, Rebecca E. Thielemann, Constantin G. Daniliuc, Osvaldo Gutierrez, Frank Glorius

**Affiliations:** † Organisch-Chemisches Institut, University of Münster, Corrensstrasse 36, 48149 Münster, Germany; ‡ Department of Chemistry, Texas A&M University, 77843 College Station, Texas, United States

## Abstract

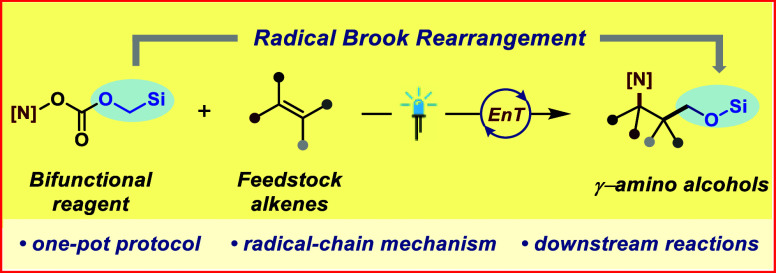

In the long-standing quest to synthesize fundamental building blocks with key functional group motifs, photochemistry in the recent past has comprehensively established its attractiveness. Amino alcohols are not only functionally diverse but are ubiquitous in the biologically active realm of compounds. We developed bench-stable bifunctional reagents that could then access the sparsely reported γ-amino alcohols directly from feedstock alkenes through energy transfer (EnT) photocatalysis. A designed 1,3-linkage across alkenes is made possible by the intervention of a radical Brook rearrangement that takes place downstream to the EnT-mediated homolysis of our reagent(s). A combination of experimental mechanistic investigations and detailed computational studies (DFT) indicates a radical chain propagated reaction pathway.

## Introduction

**Figure 1:**
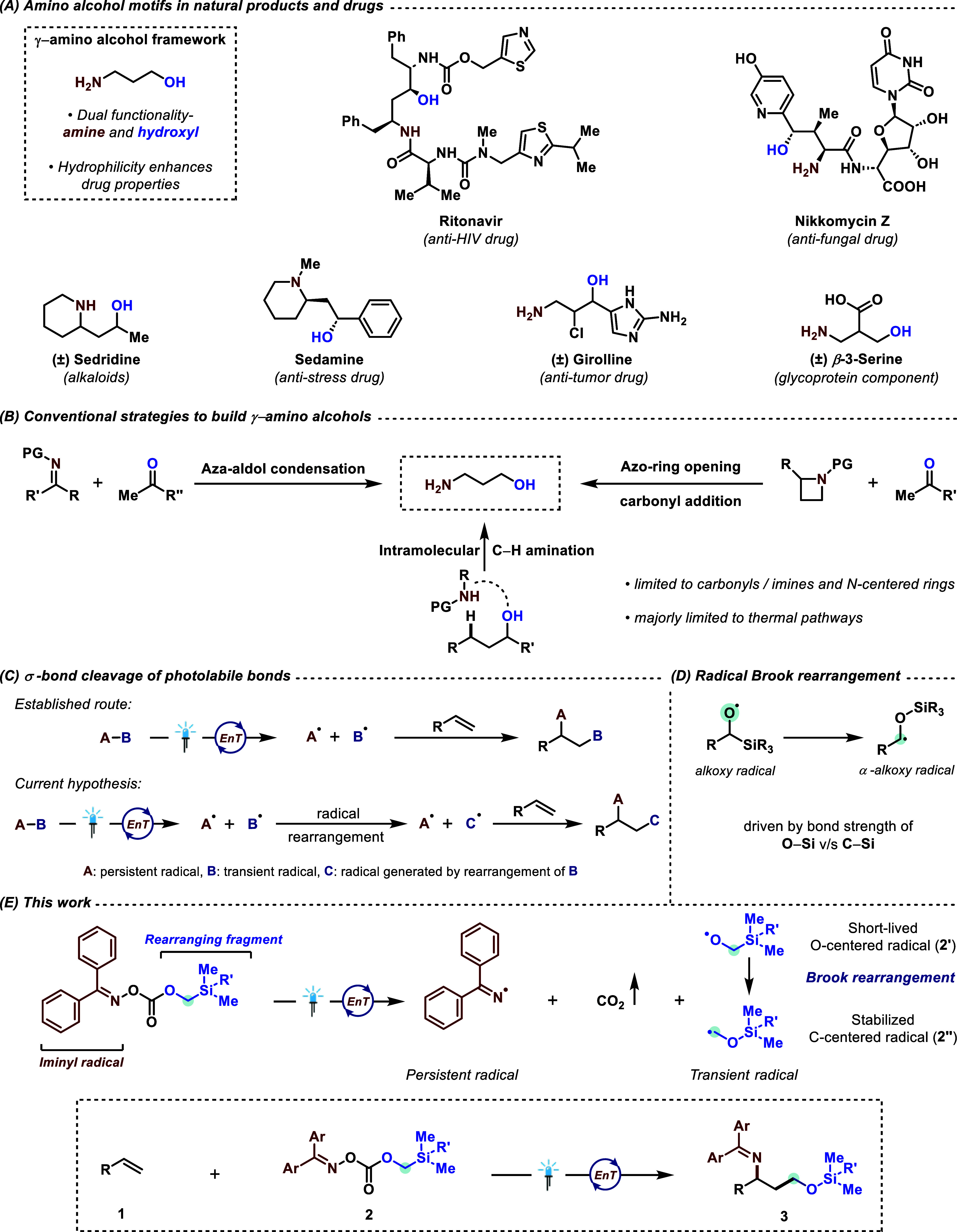
(A) γ-Amino alcohols highlighting dual chemical functionality and their prevalence in bioactive compounds, (B) conventional methods to synthesize 1,3-amino alcohols, (C) σ-bond homolysis coupled with concurrent radical rearrangement, (D) radical Brook rearrangement, and (E) working hypothesis (this work).

Amino alcohols—one of the most sought-after chemical motifs—are equipped with the potent amine (NH_2_) and hydroxyl (OH) functional groups within their core motif. These entities are known to bind with Lewis acids^1^ and transition metals^2^ making them flexible building blocks in synthetic, material, as well as in polymer chemistry.^3^ As per the 2019 FDA-approved report, out of 24 small-molecule drugs, 13 were found to have either amino acid (AA) or amino alcohol residues, outlining the success of amino alcohols in modern drug design.^4^ Updating on these statistics, the following year the FDA report established that over 30% of small-molecule drugs contain residues of AAs or are derived from amino alcohols.^5^ Moreover, amino alcohols are highly water-soluble and, hence, can be handled robustly in water-sensitive reaction conditions.^6^ To be competent in generating 1,3-amino alcohols in a one-pot procedure from alkene feedstocks through visible-light energy would facilitate easy access to these handy chemical frameworks.^7^ While most synthetic efforts focused on 1,2-amino alcohols and their reactivity, relatively fewer studies have explored γ-amino alcohols—the higher analogues for that matter.^8^ 1,3-Amino alcohols, the rarer homologues of their generic 1,2-counterparts, prominently feature as pharmacologically relevant molecules (for example, anti-HIV,^9^ antitumor^10^ drugs), natural products (alkaloids, envelope glycoprotein^11^), and also as chiral auxiliaries^12^ (Figure 1A).

So far, the *de novo* construction of 1,3-amino alcohols has been fairly limited, in terms of starting materials and methodologies.^13,14^ The synthetic progression of γ-amino alcohols is outlined by conventional methods in literature, such as aza-aldol condensation, ring-opening of aziridines and azetidines followed by carbonyl additions, and metal-mediated C–H functionalizations, to name a few (Figure 1B).^15^ Reports, however, on synthesis of the titular motifs through photocatalysis remains peculiarly underexplored.^16^

Visible light induced energy transfer (EnT) processes are well-known for their ability to induce excited state reactivity under mild conditions.^17,18^ Based on our group’s expertise in this field, we chose to investigate an innovative approach in this stride, as to date, there exist no reports leading to 1,3-amino alcohols by using this strategy. Photocatalysis-driven N–X (X = O and S) bond homolysis has gained wide recognition in the past few years.^19−22^ This neat strategy enables the installation of two fragments of interest across the alkenes for difunctionalization reactions (Figure 1C). The primary barrier to the ease of such simultaneous addition reactions would be a nondesired radical–radical homocoupling, which may be intercepted by a kinetic phenomenon known as the persistent radical effect (PRE).^23^ The differentiating lifetimes of the two radical species formed—persistent radical **A** and transient radical **B**—escalate selectivity.^24^ The major motivations at the commencement in this domain are bifaceted—(i) designing and conceptualizing reagents with unexplored persistent/transient radical motifs and (ii) synthesizing industrially relevant and biologically active product motifs that engage in pharmaceutical drug space.^25^

In this scenario, the challenge comprises the construction of a 1,3-linkage across a double bond to achieve the targeted γ-amino alcohols, since previous reports have illustrated only 1,2 additions and 1,4 additions in cases of two distinct alkenes. There is a recent stand-alone report of a 1,2,5-trifunctionalization across two alkenes; however, this is a special case with a 1,2-boron shift within an allylic boronic species, which stands of course as a prerequisite.^21^ While it is established that bifunctional reagents^26^ containing a photolabile N–X bond form chemically distinct entities that stay intact during the addition across an olefin, we desired for an exception. We postulated an in situ conversion of the transient radical **B** to a more stable radical **C** prior to the addition to olefin (Figure 1C). Inspired by the classical Brook rearrangement,^27^ we contemplated whether the radical version^28^ could meet the specified requirements (Figure 1D). In this regard, we proposed the design of our novel oxime-carbonate bifunctional reagent **2**.^20^ The introduction of a Si group on this reagent was expected to trigger the radical Brook rearrangement (RBR) downstream of the homolysis of reagent **2** (Figure 1E). This would lead to the transfer of the silyl group to the adjacent oxygen center, forming a strong covalent O–Si bond (120–130 kcal·mol^–1^, vs C–Si bond energy of 75–85 kcal·mol^–1^).^29^ Leading from our hypothesis, this would cause the transient alkoxy radical **2’** from species **2** to generate the more stabilized carbon-centered radical **2’’**, which could then undergo 1,2-addition to alkenes, while formally producing 1,3-amino alcohols (Figure 1E). The achievement of targeted 1,3-amino alcohols pointed to the success of our hypothesis and confirmed a kinetic triumph of the rapid RBR, which takes place before the alkoxy radical can directly add to the alkene (no 1,2-oxyimination products were observed). With these first results in the success of constructing protected γ-amino alcohols starting from feedstock alkenes, we strove for a more substantial overview of this radical Brook rearrangement-assisted energy transfer pathway.

## Results and Discussions

### Reagent Design and Screening Conditions

**Figure 2:**
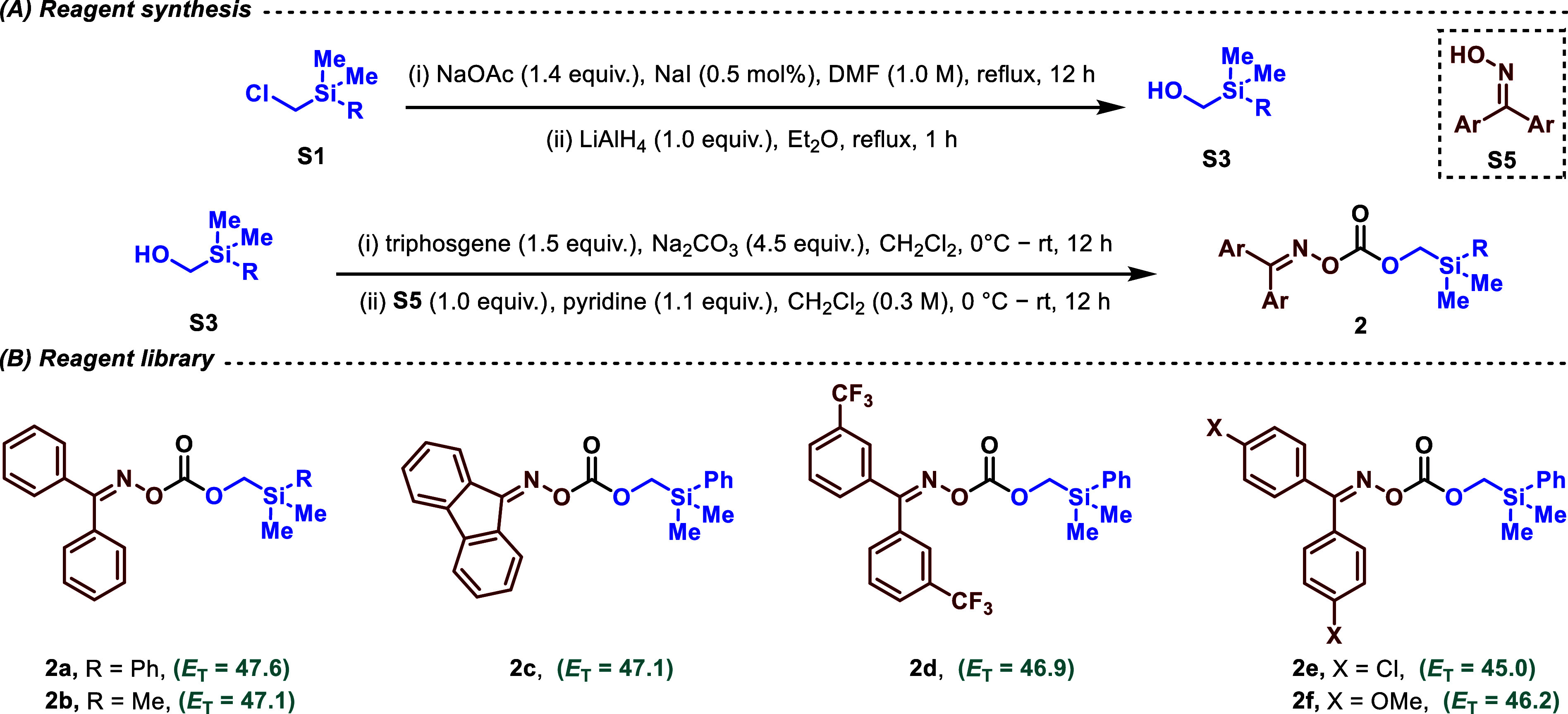
(A) Reagent synthetic pathway and (B) reagent library; triplet energies (kcal·mol^–1^) are calculated using uB3LYP-D3/def2-svp-CPCM (DCM) level of theory. DMF = *N*,*N*-dimethylformamide.

At the outset of designing bifunctional reagents, it is imperative to decide on a retrosynthetic route that guides the choice of accessible starting materials. To synthesize our Si-headed oxime carbonates, we identified the two major factions to be diaryloximes **S5** and aliphatic alcohols **S3** with a Si head on its farthest end (Figure 2A). Following a two-step synthesis from the silyl alcohols **S3**, with a final nucleophilic substitution on the oximes, the reagent motifs were assembled from commercially available starting materials (**S1** or **S3**, depending on the specific structures).

With the triplet-state energy for **2a** calculated (47.6 kcal·mol^–1^), we started our screen with thioxanthone (TXT) as the preliminary choice for photocatalyst (*E*_T_ = 65.5 kcal·mol^–1^).^30^ Gratifyingly, we observed the formation of our targeted product in 65% ^1^H NMR yield with acrylonitrile (**1a**) as the substrate. Instigated by the initial hit, we started screening through a series of parameters such as solvent, concentration, equivalents of the reagent, and a list of different photocatalysts (refer to Supporting Information, section 2.3). Hence, the reaction yield could be improved to 78% (^1^H NMR yield) by employing Ir[*d*F(CF_3_)ppy]_2_(dtbbpy))PF_6_ (Ir–F, *E*_T_ = 61.6 kcal·mol^–1^) as the photocatalyst with **1a** as the standard alkene.

### Reaction Scope

At this point, we planned to span our scope studies in two broad divisions—(i) with the synthesized reagents and (ii) with alkenes. Reagents **2b**, **2d**, and **2e** offered the desired protected γ-amino alcohol product in synthetically useful yields of 78%, 25%, and 55%, respectively. However, **2c** and **2f** gave only trace amounts of product. To this end, deviation of the reaction outcomes may involve multiple parameters such as triplet-state energies, reagent solubility, as well as steric and electronic properties.

**Figure 3:**
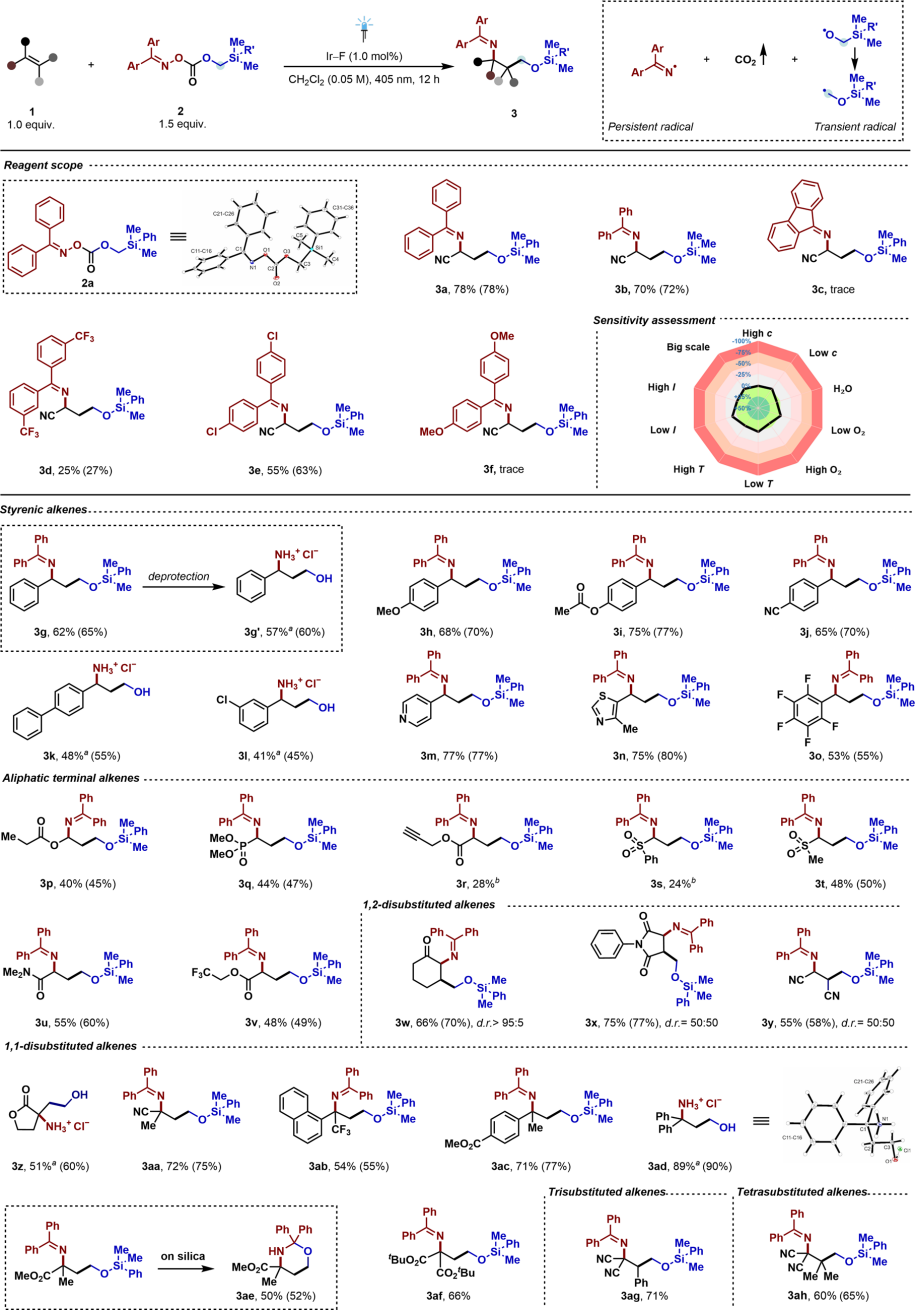
Scope table and sensitivity screen. Standard conditions: 0.2 mmol of **1**, 0.3 mmol of **2**, Ir−F (1.0 mol %), and CH_2_Cl_2_ (0.05 M). Crude ^1^H NMR yields are given in parentheses unless otherwise mentioned. ^*a*^Yield after isolation of deprotected salts (0.2 mmol)—deprotection with 2.5 mL of MeOH and 2.5 mL of 1.0 M HCl solution under air for 1 h. ^*b*1^H NMR yields are reported.

Styrenic alkenes lead to the formation of the corresponding 1,3-amino alcohol in decent to excellent yields. Varied substitutions on the aromatic ring were tolerated; notable examples would be products which have an electron-donating (**3h**) and a withdrawing group (**3j**) at the *para*-position, halogen substitution at the *meta*-position (**3l**), and pentafluoro substitution (**3o**) (Figure 3). The presence of pyridine (**3m**) and thiazole (**3n**) rings delivered excellent yields of the difunctionalized products of 77% and 75%, wherein the success of heteroarenes in the scope indicates broad applicability due to their prominence in pharmaceutical drugs.^31^ Competently, we managed to obtain the targeted protecting-group-free γ-amino alcohols (**3g’**, **3k**, and **3l**) in good yields after acidic hydrolysis. These were procured as their respective hydrochloride salts. Aliphatic terminal alkenes added another set of functional group tolerance to the scheme of the reaction. Ester (**3p**), phosphate (**3q**), terminal alkyne (**3r**), sulfones (**3s**, **3t**), amide (**3u**), and trifluoromethyl (**3v**) groups are well-tolerated, affording the corresponding products in moderate to good yields.

The reaction also supports the use of disubstituted alkenes with both 1,1- and 1,2-substitution patterns. Both cyclic alkenes like the nonsymmetric cyclohexenone (**3w**), symmetric phthalimidic alkene (**3x**), and acyclic alkenes (**3y**) are compatible substrates, affording the diastereomers as expected through their generated chiral centers. For **3w**, we note a high diastereomeric ratio (*d.r*. > 95:5) yielding majorly a cyclic *trans*-γ-amino alcohol owing to the addition of the generated radicals to the alkene in a sterically controlled manner.

1,1-Disubstituted alkenes also displayed exceptional suitability for the current protocol, exemplified by diphenylethylene with the best yielding entry **3ad** (deprotected product, yield 89%). The product **3ae** was cyclized on silica gel during the standard column chromatography isolation. More sterically hindered alkenes could be incorporated in the reaction protocol, showcased for both tri- (**3ag**) and tetra-substituted alkenes (**3ah**). However, no product formation was observed for unactivated or electron-rich alkenes. This is in line with the electronic nature of the C-centered transient radical **2a’’** which is seminucleophilic in nature.^32^ In **2a’’**, the presence of vacant 3d orbitals of Si makes the reaction possible for remote electron-donating groups such as **3h**. This also accounts for the relative stabilization of the transient radical **2a’’** (elaborated in Computational studies).

### Product Diversifications

**Figure 4:**
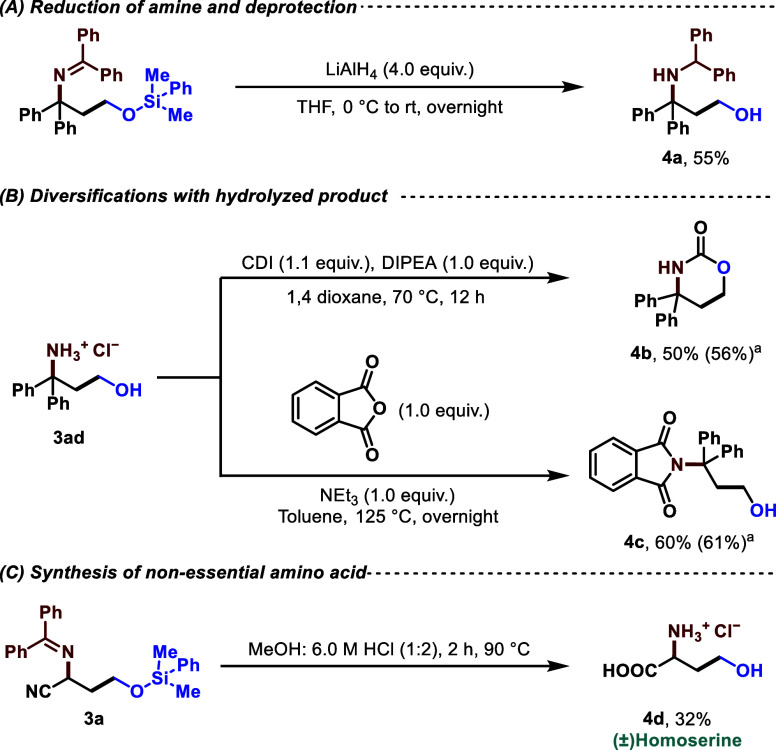
Product diversifications. ^*a*^Crude ^1^H NMR yield was determined using CH_2_Br_2_ as an internal standard. CDI = 1,1′-Carbonyldiimidazole; DIPEA = *N*,*N*-Diisopropylethylamine.

The amine and hydroxy functionalities of amino alcohols are two key starting points in terms of designing downstream reactions. In this regard, we have already mentioned the complete deprotection of some scope entries as 1,3-amino alcohol hydrochloride salts (entries **3g’**, **3k**, **3l**, **3z**, **3ad**). We managed to completely reduce the imine and deprotect the silyl group using LiAlH_4_, generating γ-amino alcohol **4a** in good conversion (Figure 4A). On the hydrolyzed product **3ad**, a cyclization with 1,1′-carbonyldiimidazole (CDI), often used in the peptide industry to link amino acids,^33^ was conducted to form carbamate **4b**,^34^ an entity with excellent proteolytic stability and a favorite class of modern drug discovery compounds (Figure 4B). Second, a single-step orthogonal protection of the amine functionality of **3ad** led to phthalimide **4c**, another class of medicinally significant products (Figure 4B).^35^ Inspired by the rising prevalence of peptidyl building blocks in various industries and the drug market,^36^ we aimed to synthesize an amino acid with an inherent 1,3-amino alcohol handle. The catalytic product **3a** with its nitrile group seemed to be the perfect substrate for this. Upon complete hydrolysis of **3a** under strong acidic conditions (6.0 M HCl) and elevated temperatures at 90 °C, we successfully synthesized racemic homoserine **4d**, a nonessential amino acid as well as an intermediate for the synthesis of three different essential amino acids (Figure 4C).

### Mechanistic Investigations

**Figure 5:**
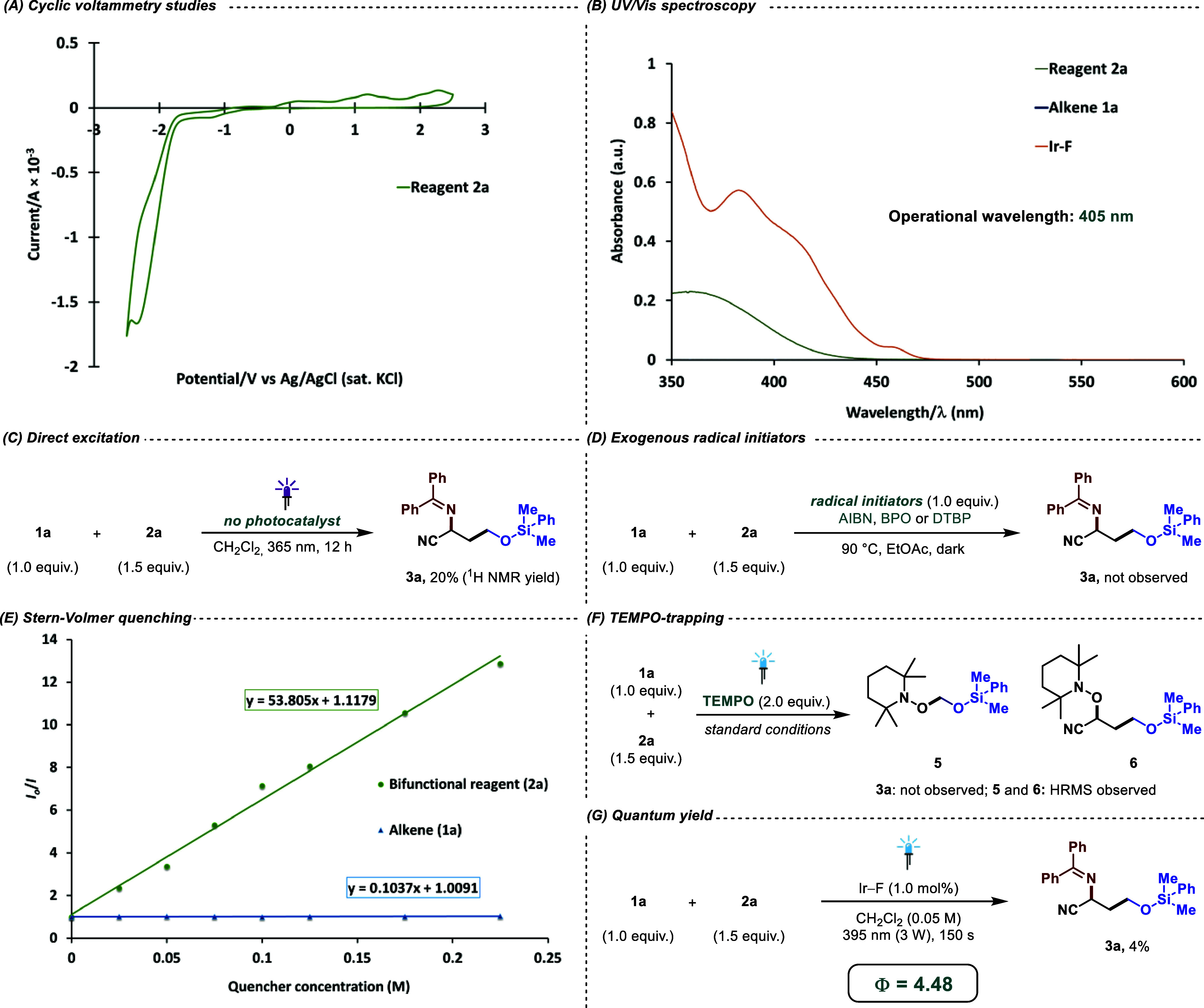
Mechanistic investigations. (A) Cyclic voltammetry studies, (B) UV/vis spectroscopy, (C) direct excitation experiments, (D) employment of thermal radical initiators as control (AIBN: Azobis(isobutyronitrile), BPO: Benzoyl peroxide, DTBP: di-*tert*-butyl peroxide), (E) Stern–Volmer quenching analysis, (F) TEMPO-trapping experiments, and (G) quantum yield analysis.

We conducted a series of mechanistic studies to further elucidate the reaction pathway. Cyclic voltammetry measurements showed reagent **2a** to undergo one single reduction event at −2.29 V vs the SCE (Figure 5A). This is in line with the assumption that the photocatalyst Ir–F {*E*_1/2_(M*/M^+^) = −0.89 V vs SCE)}^37^ does not facilitate the reduction process, thereby discarding a plausible photoredox cycle in this case. The UV/visible absorption spectrum indicates a prominent absorption of Ir–F around the operational wavelength, which indicates that the photocatalyst is the primary absorbing species (Figure 5B). In addition, **2a** is also seen to absorb around this wavelength, which made it worth investigating the photocatalytic reaction under direct excitation conditions. This led us to perform the standard reaction at 365 nm in the absence of an Ir–F (Figure 5C). As expected, it delivered the anticipated product in a 20% ^1^H NMR yield. The independent addition of three distinct radical initiators in the dark did not deliver any catalytic product, thereby demonstrating the pivotal role of the photocatalyst (Figure 5D). Stern Volmer quenching studies indicated **2a** as the sole species quenching the photocatalyst, as **1a** showed no signs of quenching Ir–F (Figure 5E). To identify the radicals and estimate their reactivities, a trapping experiment with TEMPO was conducted. The formation of the desired catalytic product was completely ceased, while a transient radical adduct (**5**) and an adduct of α-oxy radical added to the alkene **1a** (**6**) were observed with TEMPO (Figure 5F). In our best guess, the persistent radical undergoes homocoupling^38^ in this time frame, as is detected in the ESI spectra. Additionally, we performed the quantum yield experiments using 395 nm wavelength to achieve a quantum yield, Φ = 4.48, for our present protocol (Figure 5G). This indicates a plausible radical chain propagation in addition to the radical recombination pathway.^22^

### Computational Studies (DFT)

**Figure 6:**
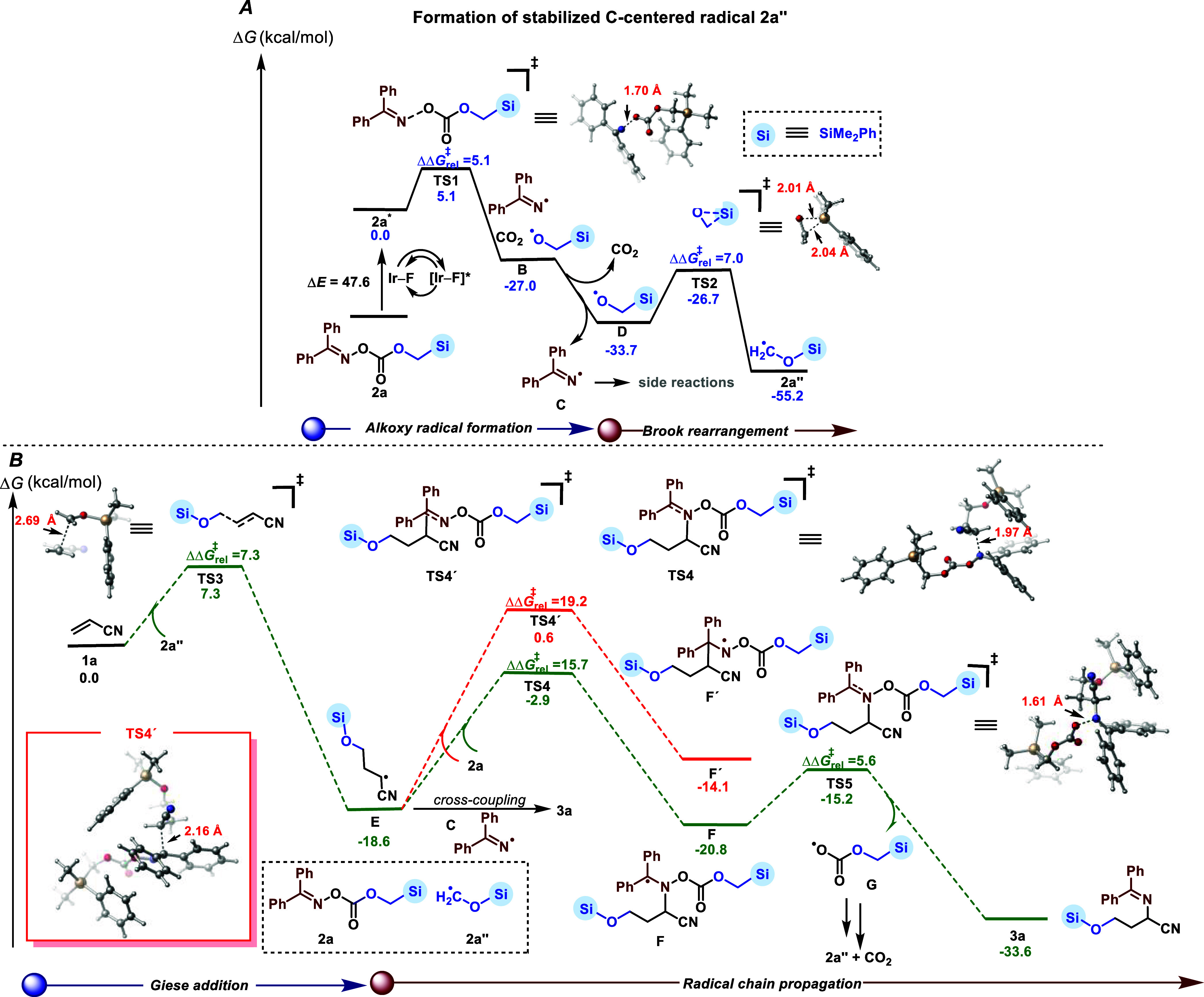
Proposed mechanism was supported by computational studies. Calculated free Gibbs energies [CPCM(DCM) uB3LYP-D3/def2-svp] are given in kcal·mol^–1^. For details, see the Supporting Information.

Primarily, to gain insights into the radical-chain mechanism, we turned to dispersion-corrected density functional theory (DFT) calculations (see Supporting Information for additional details). As shown in Figure 6A, N–O and C–O bonds cleavage takes place in the triplet state of **2a** (47.6 kcal·mol^–1^ uphill in energy which can be accessed by triplet–triplet EnT with excited Ir–F)^18^ via **TS1** with a small energy barrier (ΔΔ*G*^‡^_rel_ = 5.1 kcal·mol^–1^). This generates the ambiphilic N-centered iminyl radical **C** and the nucleophilic alkoxy radical **D** with spontaneous release of CO_2_ (33.7 kcal·mol^–1^ downhill from the excited-state intermediate 2a*; Figure F8, see Supporting Information for details). Then, **D** can undergo irreversible Brook rearrangement via **TS2** (ΔΔ*G*^‡^_rel_ = 7.0 kcal·mol^–1^) to form the stabilized C-centered radical **2a’’**. Then, we hypothesized that the long lifetime of diphenyliminyl radical **C** allows the transient radical **2a’’** to add exclusively to the terminal position of the alkene, generating the more stable radical **E** (Figure 6B). As expected, DFT calculations showed that the addition of **2a’’** radical onto acrylonitrile **1a** via **TS3** proceeds regioselectively and irreversibly via a low energy barrier of 7.3 kcal·mol^–1^ to deliver intermediate **E** (downhill in energy by 18.6 kcal·mol^–1^). In addition, we explored the addition of alternative radicals over **1a** (e.g., the ambiphilic radical **C**, nucleophilic alkoxy radical **D**, etc.). However, the energy barriers were found to be significantly higher (>16 kcal·mol^–1^, Figure F10, see Supporting Information) and thus were not considered further. Next, a selective radical–radical cross-coupling of **E** and **C** to form **3a** would be feasible based on the PRE, but given the experimental evidence (ϕ = 4.48) and the relative concentration of the oxime ester **2a** versus the iminyl radical (**C**), intermediate radical **E** could undergo addition to **2a** via **TS4** (barrier of 15.7 kcal·mol^–1^) along the radical chain pathway,^22^ leading to the formation of radical intermediate **F**. Then, **F** can rapidly fragment and form product **3a** via **TS5** (barrier of 5.6 kcal·mol^–1^), simultaneously releasing **G**. Finally, after facile decarboxylation, radical **D** is formed along with CO_2_.

The radical **D** then undergoes Brook rearrangement resulting in the formation of **2a’’** which selectively adds to another alkene, thus restarting the cycle. In addition, we also considered an alternative path for the addition of radical **E** onto oxime ester (**2a**) through **TS4′** but ruled out this pathway based on a higher energy barrier compared to **TS4** (19.2 vs 15.7 kcal·mol^–1^).

## Conclusion

In a one-pot protocol, alkenes were photocatalytically converted into 1,3-amino alcohols by employing bifunctional reagents that could undergo N–O bond homolysis, followed by radical Brook rearrangement. A diverse substrate scope and notable product diversifications contribute to the broad significance of this study. Mechanistic experiments, carried out to decipher the reaction pathway, point toward an EnT-based mechanism through radical chain propagation, which is in line with our computational studies (DFT). We hope that the herein-exhibited mechanistic combination of bifunctional reagent homolysis along with rearrangement reactions stands as a gateway, unfolding substantial room in the realm of energy transfer photocatalysis.

## Supporting Information Available

All experimental data and characterization of compounds synthesized (PDF)
