# Supplementary material for: γ-Amino Alcohols via Energy Transfer Enabled Brook Rearrangement
Source: J Am Chem Soc. 2024 Apr 3;146(15):10899–907. doi: 10.1021/jacs.4c01667 (PMC11027157; doi:10.1021/jacs.4c01667)
Supplement: Supplementary file 1 — ja4c01667_si_001.pdf [file ja4c01667_si_001.pdf]

Supporting Information

γ–Amino Alcohols via Energy Transfer Enabled Brook Rearrangement

Ranjini Laskar,[a,§] Subhabrata Dutta,[a,§] Jan C. Spies,[a] Poulami Mukherjee,[b] Ángel Rentería-Gómez,[b] Rebecca E. Thielemann,[a] Constantin. G. Daniliuc,[a] Osvaldo Gutierrez,[b,*] Frank Glorius[a,*]

[§] *denotes equal contribution*

[a] Organisch-Chemisches Institut, University of Münster, Corrensstrasse 36, 48149 Münster, Germany. [b] Department of Chemistry, Texas A&M University, College Station, Texas 77843, USA.

*E-Mail: glorius@uni-muenster.de

og.labs@tamu.edu

Contents

1 General considerations S4

2 Experimental observations and characterization data S6

2.1 Reagent synthesis S6

2.1.1 Procedure for (dimethyl(phenyl)silyl) methanol S6

2.1.2 General procedure for diarylmethanone oximes S6

2.1.3 General procedure for reagent synthesis (GP-A) S7

2.1.4 Synthesis of reagents S8

2.2 Alkene synthesis and library S11

2.2.1 Synthesis of alkenes S11

2.3 Optimization studies and control experiments S12

2.3.1 Deviation from standard conditions S12

2.4 General procedures for catalytic reactions S13

2.4.1 General procedure for products isolated as silyl ethers S13

2.4.2 General procedure for products isolated as amino alcohols S13

2.5 Synthesis of 1,3-amino alcohols S14

2.5.1 Silyl-oxime carbonate scope S14

2.5.2 Alkene scope S16

2.5.3 Limitations S28

2.6 Sensitivity assessment S29

2.7 Product diversifications S31

2.7.1 Complete reduction of the standard product S31

2.7.2 One-step transformation of 1,3-amino alcohol to carbamate S31

2.7.3 One-step orthogonal functionalization of 1,3-amino alcohol to phthalimide S32

2.7.4 One-step synthesis of non-essential amino acid homoserine S33

2.8 Crystallographic data S34

3 Mechanistic investigations S36

3.1 Cyclic voltammetry studies S36

3.2 UV/Visible absorption spectroscopy S37

3.3 Direct excitation S37

3.4 Cross-relationship between yield and photocatalyst triplet energies S38

3.5 Stern Volmer quenching S39

3.6 TEMPO-trapping experiment S40

3.7 Radical initiators S41

3.8 Concept of transient-radical shift and Brook rearrangement S41

3.9 Quantum yield calculations S42

3.9.1 Determination of the photon flux S42
3.9.2 Determination of the quantum yield S43
4 Computational studies S44
4.1 Proposed mechanism S44
4.2 Addition of alternative radicals with alkene **1a** S46
4.3 Cartesian coordinates S47
5 Spectra S84
6 References S130

1 General considerations

Unless otherwise stated, all reactions were carried out under a positive atmosphere of argon in oven-dried or flame-dried glassware. Prior to the reaction set-up, glassware was evacuated and backfilled with argon three times. The solvents dichloromethane (DCM), methanol (MeOH), toluene, tetrahydrofuran (THF), *N*,*N*-dimethylformamide (DMF) and diethyl ether (Et_2O) applied in synthesis were purified by solvent purification system (SPS) over standard drying materials with positive argon flow and stored under argon. Ethyl acetate (EtOAc), acetonitrile (MeCN), acetone, and triethylamine (NEt_3) were stored over molecular sieves under argon.

Photochemical set-up and light sources Photochemical reactions were performed in a Hepatochem EvoluChem™ PhotoRedOx Box Duo device and irradiated with two EvoluChem™ HCK1012-01-006 LEDs (30 W, λ_{max} = 365 nm) or two EvoluChem™ HCK1012-010 LEDs (18 W, λ_{max} = 405 nm) or two EvoluChem™ HCK1012-01-008 (30 W, λ_{max} = 450 nm). To shield irradiation, the setup was covered with a small cardboard box. The reaction temperature was determined to be between 30 °C and 33 °C using this setup.

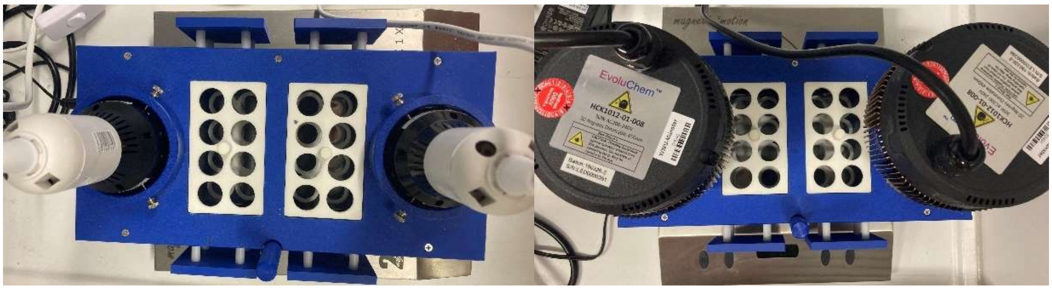

Figure S1. Hepatochem EvoluChem™ PhotoRedOx Box Duo device with two EvoluChem™ (405nm [left] or 450 nm [right]).

Column chromatography and solvents Analytical thin layer chromatography (TLC) was performed using silica gel 60 F254 aluminum plates by Merck. TLC plates were visualized by exposure to short wave ultraviolet light (254 nm or 365 nm) and/or were dipped into a solution of $KMnO_4$ (3.0 g) and K_2CO_3 (10.0 g) in H_2O (300 mL). Column chromatography was carried out using silica gel (40-63 mesh) by Merck eluting with the below mentioned solvent system under positive pressurized air flow. Pentane, DCM, ethyl acetate, toluene and diethyl ether for column chromatography or recrystallization were purchased of technical grade and further purified *via* distillation. Unless otherwise mentioned, dry solvents were used to perform the preparation of starting materials and catalytic reactions. The following solvents were purchased from ACROS Organics, Fischer Scientific and Sigma-Aldrich (HPLC grade) and purified using a custom solvent purification system (SPS) with activated alumina columns (built by the "Feinmechanische Werkstatt des Organisch-Chemischen Instituts, WWU Münster") and collected under positive argon pressure: MeCN, THF, Et_2O, DMF, hexane, toluene, MeOH and DCM.

NMR and deuterated solvents NMR-spectra were recorded at room temperature (in few cases high or low temperature measurements were recorded and indicated as such) on a Bruker Avance II 300, Bruker Avance II 400, Bruker Avance Neo 400, Agilent DD2 500 or on an Agilent DD2 600 spectrometer. Chemicals shifts (δ) are quoted in ppm downfield of tetramethylsilane. The residual solvent signals were used as references for ^1H and ^{13}C NMR spectra (relative to tetramethylsilane at 0.0 ppm, $CDCl_3$: δ_H = 7.26 ppm, δ_C = 77.16 ppm; CD_2Cl_2: δ_H = 5.32 ppm, δ_C = 54.00 ppm; D_2O: δ_H = 4.79 ppm; MeOD δ_H = 3.30 ppm, δ_C = 49.10 ppm). In case of D_2O, ^{13}C spectra were not referenced. The multiplicity of all signals was described with standard abbreviations as follows: s = singlet, d = doublet, t = triplet, q = quartet, p = quintet, h = sextet;

hept = heptet; m = multiplet, br = broad signal. All the NMRs were processed using Mestrenova 14 applying standard phase and baseline corrections. Coupling constants (*J*) are quoted in Hz. Crude yields were determined by ^{1}H using CH_2Br_2 as internal standard.

GC-MS Samples for GC-MS were filtered over a plug of silica and eluated with EtOAc prior to analysis or were directly collected from the eluted section from column chromatography on silica gel. GC-MS spectra were recorded on an Agilent Technologies 7890A GC-system using an Agilent 5975C VL MSD or an Agilent 5975 inert Mass Selective Detector (EI) and a HP-5MS column (0.25 mm x 30 m, film: 0.25 μm).

HRMS High-resolution mass spectra (HRMS) were obtained by the mass department of the Organisch-Chemisches Institut, Universität Münster, using electron ionization (EI) on an Exactive GC-MS by Thermo Fisher Scientific or electrospray ionisation (ESI) on a Bruker Daltonics, MicroToF spectrometer.

Chemicals The alkenes were mostly all commercially available ones, as mentioned later, with a few exceptions. (Chloromethyl)dimethylphenylsilane and (trimethylsilyl)methanol was purchased from ABCR chemicals and used as received. Photocatalysts (Ir[*d*F(CF_3)ppy]$_2$(dtbbpy))PF_6 (Ir–F),[1] [Mes_2Acr]ClO_4,[2] [Ir(ppy)$_2$(dtbpy)]PF_6[3] 4-CzIPN,[4] and *fac*-Ir(ppy)$_3$[5] was prepared following reported literature procedures. Thioxanthone was purchased from BLD Pharm.

2 Experimental observations and characterization data

2.1 Reagent synthesis

2.1.1 Procedure for (dimethyl(phenyl)silyl) methanol

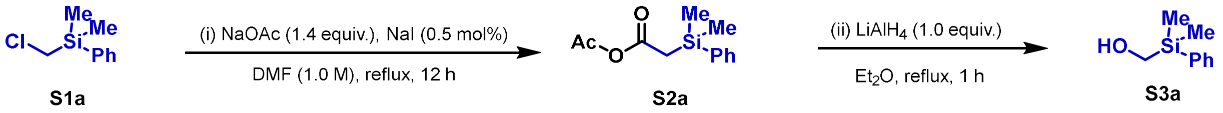

The silyl alcohol **S3a** was synthesized according to the modified reported literature.[6] Sodium acetate (3.1 g, 38.0 mmol,1.4 equiv.), sodium iodide (540 mg, 3.6 mmol, 0.5 mol%) were weighed in a Schlenk tube and flushed with argon. To this (chloromethyl)dimethylphenylsilane **S1a** (5.0 g, 27 mmol, 1.0 equiv.), and dry DMF (30 mL) were added in succession and stirred under reflux for 12 h. After completion, the reaction was checked by GC-MS and ice-cold water was added to it. Then the mixture was extracted with Et_2O (3x10 mL). The combined organic layers were washed with water (3 x 20 mL), HCl (20 mL, 2.0 M), and a saturated aqueous solution of $NaHCO_3$ (20 mL). This was dried over $MgSO_4$ and concentrated under reduced pressure. The crude (dimethylphenylsilyl)methyl acetate **S2a** (5.4 g) was dissolved in dry Et_2O (30 mL) and added to a stirred mixture of $LiAlH_4$ (1.1 g, 27 mmol, 1.0 equiv.) in dry Et_2O (30 mL), which had been refluxed for 5 min and then cooled, at a rate to keep it under gentle reflux without heating. Refluxing was continued for 1 h. Water (10 mL, ice-cold) was added carefully to the reaction mixture. The precipitated aluminium hydroxide was then dissolved with HCl (solution of 12 mL of concentrated HCl 37% reagent grade, and 22 mL of water). The organic phase was separated and the aqueous phase was extracted with Et_2O (2 x 20 mL). The combined organic layers were washed with water (20 mL) and a saturated aqueous solution of $NaHCO_3$ (20 mL), dried ($MgSO_4$) and concentrated under reduced pressure. The organic residue was dry loaded with silica and a flash column chromatography (pentane/EtOAc = 90/10) was run to isolate the pure alcohol (isolated yield, after 2nd step, 80%, 3.6 g). The analytics are in good parity with literature.

^{1}H NMR (400 MHz, $CDCl_3$): δ 7.61 – 7.54 (m, 2H), 7.38 (dd, J = 5.0, 1.9 Hz, 3H), 3.59 (s, 2H), 0.36 (s, 6H).

^{13}C NMR (101 MHz, $CDCl_3$): δ 136.8, 133.8, 129.3, 127.9, 55.3, -3.0.

2.1.2 General procedure for diarylmethanone oximes

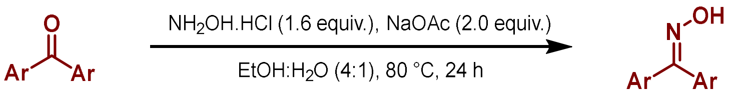

Diarylmethanone oximes was prepared following reported literature procedure.[7,8] In a 250 mL round-bottom flask equipped with a findenser, diarylketone (50 mmol, 1.0 equiv.) were dissolved in the mixture of EtOH/H_2O (v/v, 4:1, 125 mL). Then, hydroxylamine hydrochloride (5.6 g, 80 mmol, 1.6 equiv.) and NaOAc (8.2 g, 100 mmol, 2.0 equiv.) were added in one portion. The reaction mixture was refluxed at 80˚C overnight and the consumption of the starting material was observed by TLC till 24 h (single spot, by TLC). After that, the reaction was cooled to room temperature and concentrated under reduced pressure to remove the ethanol as much as possible. Then the white solid was diluted with water (55 mL), extracted with ethyl acetate (3x80 mL) and dried over anhydrous $MgSO_4$. Evaporation of the solvent afforded the product in distinctive yields. This resultant solid was used directly for the preparation of the silyl carbonate reagents.

2.1.3 General procedure for reagent synthesis (GP-A)

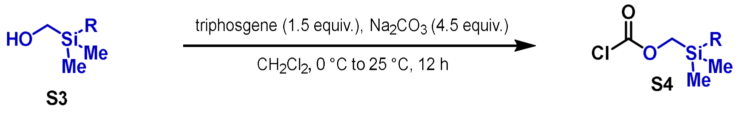

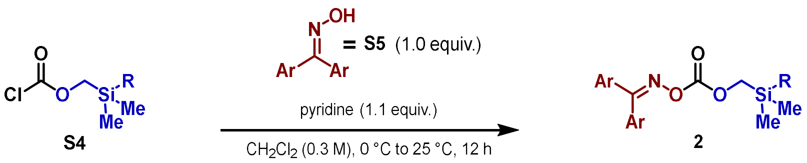

To a 150 mL Schlenk tube, triphosgene (15 mmol, 1.5 equiv.), Na_2CO_3 (45 mmol, 4.5 equiv.) was charged and back-filled a three times with argon.[9] To this mixture, the silyl alcohol **S3** (10 mmol, 1.0 equiv.) diluted with 10 mL DCM, was added with stirring at 0˚C. Another 30 mL DCM was added to this, and the resultant solution was stirring at room temperature overnight. After checking reaction progress by TLC, the resultant residue was filtered through a sintered funnel and the filtrate was collected. The white residue was washed three times with toluene (3x10 mL) and the silicon-headed acyl chloride was obtained by evaporating the toluene and DCM from the filtrate. This was furthered onto the next step directly.

To a new oven-heated Schlenk tube, the residual diarylmethanone oxime (10.0 mmol, 1.0 equiv.) was charged and back-filled with argon three times. This was dissolved in 20 mL dry DCM and stirred at 0˚ C for 5 minutes. To this stirring mixture, the resultant acyl chloride **S4** (10.0 mmol, 1.0 equiv.) from the previous step was added dropwise, being dissolved in another 20 mL dry DCM. Pyridine (11 mmol, 1.1 equiv.) was then added dropwise at this temperature, and a white precipitate formation was observed. This was left stirring overnight for 12 h and monitored by TLC.[10] Thereafter, the product was washed with water (20 mL) three times. The combined organic layers were dried over $MgSO_4$ and the solvent was removed. The organic residue was dry loaded with silica and a column chromatography (pentane/EtOAc) was carried out to obtain the pure reagent **2**(s).

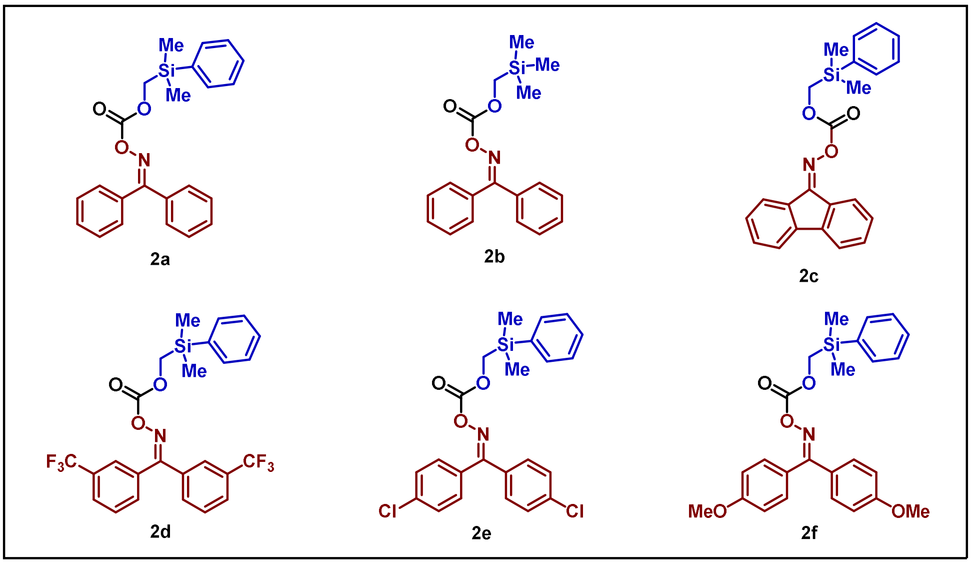

Fig F1. Library of bifunctional reagents as synthesized following **GP-A**.

2.1.4 Synthesis of reagents

Diphenylmethanone O-(((dimethyl(phenyl)silyl)methoxy)carbonyl) oxime (2a)

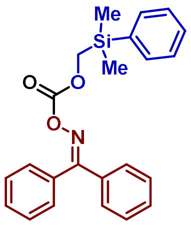

Was synthesized from diphenylmethanone oxime (1.9 g, 10 mmol, 1.0 equiv.) and (dimethyl(phenyl)silyl)methanol (1.6 g, 10 mmol., 1.0 equiv.) following **GP-A**. Purification by column chromatography on silica gel (pentane/EtOAc = 97/3 − 93/7) afforded the desired product (2.0 g, 5.2 mmol, 52%) as a white solid. $\mathbf{R_f}$ (pentane/EtOAc = 95/5) = 0.3.

^{1}H NMR (400 MHz, $CDCl_3$) δ 7.60 – 7.53 (m, 4H), 7.49 – 7.41 (m, 4H), 7.41 – 7.30 (m, 7H), 4.16 (s, 2H), 0.38 (s, 6H).

^{13}C NMR (101 MHz, $CDCl_3$) δ 164.4, 155.4, 135.9, 134.9, 133.9, 132.3, 130.9, 129.8, 129.8, 129.2, 129.2, 128.5, 128.3, 128.1, 61.8, -4.7.

HRMS (ESI$^+$): $[M+Na]^+$ 412.1345; found 412.1339.

Diphenylmethanone O-(((trimethylsilyl)methoxy)carbonyl) oxime (2b)

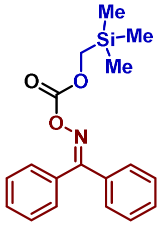

Was synthesized from diphenylmethanone oxime (1.9 g, 10 mmol, 1.0 equiv.) and (trimethylsilyl)methanol (1.0 g, 10 mmol., 1.0 equiv., *commercially available*) following **GP-A**. Purification by column chromatography on silica gel (pentane/EtOAc = 95/5 − 90/10) afforded the desired product (2.2 g, 7.0 mmol, 70%) as a white solid. $\mathbf{R_f}$ (pentane/EtOAc = 95/5) = 0.3.

^{1}H NMR (400 MHz, $CDCl_3$) δ 7.60 – 7.53 (m, 2H), 7.51 – 7.40 (m, 4H), 7.39 – 7.31 (m, 4H), 3.96 (s, 2H), 0.09 (s, 9H).

^{13}C NMR (101 MHz, $CDCl_3$) δ 164.7, 155.8, 135.3, 132.6, 131.2, 130.2, 129.5, 129.5, 128.8, 128.7, 63.1, -2.7.

HRMS (ESI$^+$): $[M+Na]^+$ 350.1188; found 350.1179.

9*H*-Fluoren-9-one O-(2-((dimethyl(phenyl)silyl)oxy)acetyl) oxime (2c)

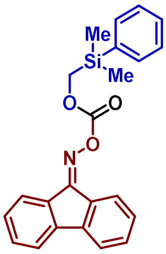

Was synthesized from 9*H*-fluoren-9-one oxime (1.9 g, 10 mmol, 1.0 equiv.) and (dimethyl(phenyl)silyl)methanol (1.6 g, 10 mmol., 1.0 equiv.) following **GP-A**. Purification by column chromatography on silica gel (pentane/EtOAc = 95/5 to 90/10) afforded the desired product (1.3 g, 3.5 mmol, 35%) as a yellow oil. $\mathbf{R_f}$ (pentane/EtOAc = 90/10) = 0.25.

^{1}H NMR (400 MHz, $CDCl_3$) δ 8.27 (dt, J = 7.7, 1.0 Hz, 1H), 7.92 (dt, J = 7.6, 1.0 Hz, 1H), 7.65 – 7.55 (m, 4H), 7.49 – 7.37 (m, 5H), 7.36 – 7.25 (m, 2H), 4.30 (s, 2H), 0.49 (s, 6H).

^{13}C NMR (101 MHz, $CDCl_3$) δ 158.1, 155.2, 142.5, 141.3, 135.8, 134.4, 134.1, 132.7, 131.7, 130.7, 129.9, 128.7, 128.5, 128.1, 123.4, 120.3, 120.2, 62.2, -4.4.

HRMS (ESI$^+$): $[M+Na]^+$ 410.1188; found 410.1181.

Bis(3-(trifluoromethyl)phenyl)methanone O-(((dimethyl(phenyl)silyl)methoxy)carbonyl) oxime (2d)

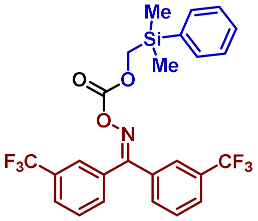

Was synthesized from bis(3-(trifluoromethyl)phenyl)methanone oxime (3.3 g, 10 mmol, 1.0 equiv.) and (dimethyl(phenyl)silyl)methanol (1.6 g, 10 mmol., 1.0 equiv.) following **GP-A**. Purification by column chromatography on silica gel (pentane/EtOAc = 95/5 to 90/10) afforded the desired product (2.1 g, 4.0 mmol, 40%) as a white solid. $\mathbf{R_f}$ (pentane/EtOAc = 95/5) = 0.4.

^{1}H NMR (400 MHz, $CDCl_3$) δ 7.85 (dt, J = 1.9, 1.1 Hz, 1H), 7.82 – 7.69 (m, 3H), 7.63 (dq, J = 2.5, 0.8 Hz, 2H), 7.52 (dt, J = 8.0, 1.6 Hz, 4H), 7.42 – 7.33 (m, 3H), 4.15 (s, 2H), 0.39 (s, 6H).

^{19}F NMR (376 MHz, $CDCl_3$) δ -62.8, -62.8.

^{13}C NMR (101 MHz, $CDCl_3$) δ 161.5, 154.9, 135.6, 135.1, 133.9, 132.5, 132.3, 132.1, 131.6, 131.5, 131.3, 131.2, 129.9, 129.4, 129.4, 128.1, 127.9, 127.2, 126.1, 125.7, 62.4, -4.5.

HRMS (ESI$^+$): $[M+Na]^+$ 548.1093; found 548.1090.

**Note: The quartets in the ^{13}C spectra cannot be distinguished well and hence all the peaks are separately picked.*

Bis(4-chlorophenyl)methanone O-(((dimethyl(phenyl)silyl)methoxy)carbonyl) oxime (2e)

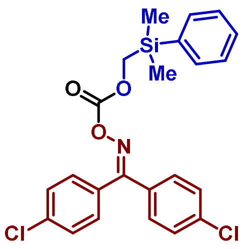

Was synthesized from bis(4-chlorophenyl)methanone oxime (2.6 g, 10 mmol, 1.0 equiv.) and (dimethyl(phenyl)silyl)methanol (1.6 g, 10 mmol., 1.0 equiv.) following **GP-A**. Purification by column chromatography on silica gel (pentane/EtOAc = 99/1 to 96/4) afforded the desired product (1.6 g, 3.5 mmol, 35%) as a yellow oil. **R_f** (pentane/EtOAc = 95/5) = 0.5.

^{1}H NMR (400 MHz, $CDCl_3$) δ 7.47 – 7.34 (m, 6H), 7.33 – 7.25 (m, 5H), 7.19 – 7.15 (m, 2H), 4.08 (s, 2H), 0.31 (s, 6H).

^{13}C NMR (101 MHz, $CDCl_3$) δ 162.2, 155.1, 137.5, 136.3, 135.7, 134.0, 133.0, 130.6, 130.3, 129.8, 128.9, 128.9, 128.1, 62.1, -4.4.

HRMS (ESI$^+$): $[M+Na]^+$ 480.0565; found 480.0561.

**Note: Trapped EtOAc appears in the ^{1}H spectra of 2e. Solvent does not go out after 4 days of high vac.*

Bis(4-methoxyphenyl)methanone O-(((dimethyl(phenyl)silyl)methoxy)carbonyl) oxime (2f)

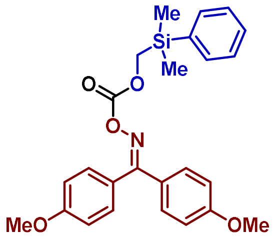

Was synthesized from bis(4-methoxyphenyl)methanone oxime (2.5 g, 10 mmol, 1.0 equiv.) and (dimethyl(phenyl)silyl)methanol (1.6 g, 10 mmol., 1.0 equiv.) following **GP-A**. Purification by column chromatography on silica gel (pentane/EtOAc = 99/1 to 96/4) afforded the desired product (1.6 g, 3.5 mmol, 35%) as a yellow solid. **R_f** (pentane/EtOAc = 95/5) = 0.5.

^{1}H NMR (400 MHz, $CDCl_3$) δ 7.58 – 7.46 (m, 4H), 7.41 – 7.34 (m, 3H), 7.31 – 7.26 (m, 2H), 6.98 – 6.92 (m, 2H), 6.90 – 6.84 (m, 2H), 4.15 (s, 2H), 3.87 (s, 3H), 3.83 (s, 3H), 0.39 (s, 6H).

^{13}C NMR (101 MHz, $CDCl_3$) δ 163.9, 161.9, 160.7, 155.6, 136.0, 134.0, 131.4, 131.0, 129.7, 128.1, 127.7, 124.6, 113.9, 113.6, 61.6, 55.5, 55.5, -4.3.

HRMS (ESI$^+$): $[M+Na]^+$ 472.1556; found 472.1550.

2.2 Alkene synthesis and library

Table T1. Library of successful alkene substrates, both commercially available and synthesized.

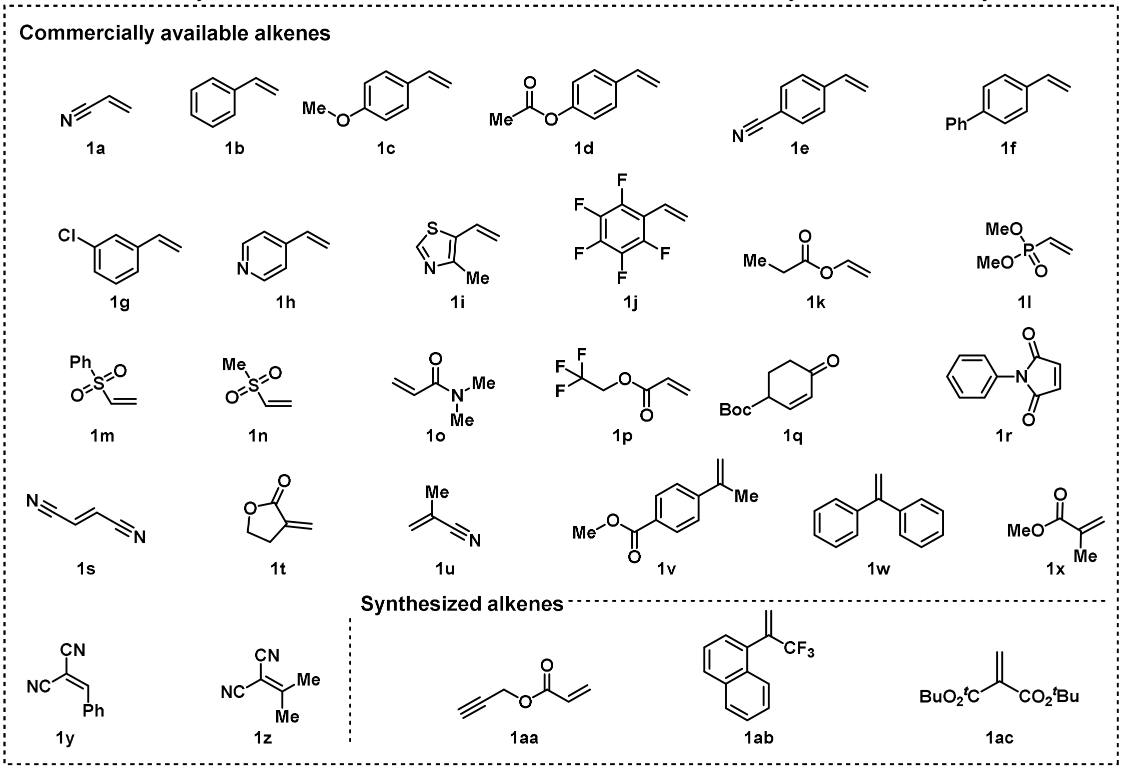

2.2.1 Synthesis of alkenes

Prop-2-yn-1-yl acrylate (1aa)

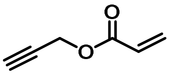

Was synthesized modifying a reported literature procedure. In a 100 mL Schlenk tube, propargyl alcohol (561 mg, 10.0 mmol, 1.0 equiv.) and Et_3N (1.7 mL, 12 mmol, 1.2 equiv.) was added and stirred at 0˚C for 5 minutes. To this acryloyl chloride (1.0 mL, 1.1 g, 12 mmol, 1.2 equiv.) was added dropwise, and the solution turned yellow slowly: this was left to stirred overnight. After completion of reaction, it was washed with saturated $NaHCO_3$ solution, and extracted using EtOAc. The organic layer was collected and dried over $MgSO_4$. Purification by flash column chromatography (pentane/EtOAc = 50/50) to obtain a clear yellow liquid product (359 mg, 3.3 mmol , 33%). **R_f** (pentane/EtOAc = 50/50) = 0.5.

^{1}H NMR (400 MHz, $CDCl_3$) δ 6.48 (dd, J = 17.3, 1.3 Hz, 1H), 6.16 (dd, J = 17.3, 10.5 Hz, 1H), 5.90 (dd, J = 10.5, 1.3 Hz, 1H), 4.77 (d, J = 2.5 Hz, 2H), 2.49 (t, J = 2.5 Hz, 1H).

^{13}C NMR (101 MHz, $CDCl_3$) δ 165.4, 132.1, 127.7, 75.1, 52.2.

* Alkenes **1ab**,[11] and **1ac**[12] were re-used directly from the previously synthesized batches (with a GC/MS check for purity) from our group, and synthesized and fully characterized in the cited references.

2.3 Optimization studies and control experiments

2.3.1 Deviation from standard conditions

Table T2. Optimization studies and control experiments for bifunctionalized products. All reactions were performed on 0.1 mmol scale following **GP I**. Yield determined by ^{1}H NMR using CH_2Br_2 internal standard.

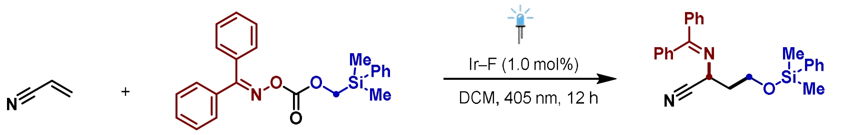

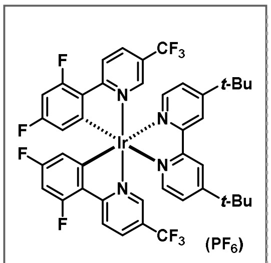

Entry	Variation from the standard conditions	Reaction yield (%) of 3a
1	none	78
2	**1** (1.0 equiv.), **2** (1.0 equiv.)	36
3	**1** (1.0 equiv.), **2** (2.0 equiv.)	62
4	PC (2 mol%)	70
5	EtOAc (0.1 M)	38
6	MeCN (0.1 M)	53
7	THF (0.1 M)	trace
8	DCM (0.1 M)	62
9	*fac*-Ir(ppy)$_3$ (2 mol%)	n.d.
10	[MesAcr]ClO$_4$	n.d.
11	4-CzIPN	48
12	TXT	65
13	without *hv*	n.d.
14	without PC	n.d.
15	only heat, no *hv* (60° C)	n.d.

As noted from Table **T2**, the reaction scheme works well with organic metal-free catalyst thioxanthone, however Ir–F proves to be the better catalyst. On conducting experiments without visible light, photocatalyst or only heat, no desired product formation was detected.

2.4 General procedures for catalytic reactions

2.4.1 General procedure for products isolated as silyl ethers

General Procedure I (GP I):

An oven-dried 10 mL Schlenk tube was equipped with a Teflon-coated stir bar, silyl-oxime carbonates **2** (0.3 mmol, 1.5 equiv.) and Ir–F (2.5 mg, 0.01 mmol, 1 mol%). The Schlenk tube was evacuated and backfilled with argon three times, before dry DCM (4 mL, 0.05 M) and alkene **1** (0.2 mmol, 1.0 equiv.) was added (if alkene was a solid, it was added prior to adding the solvent). The Schlenk tube was set under argon atmosphere and irradiated under 405 nm irradiation for 12 h at room temperature. Upon completion, the solvent was evaporated and ^{1}H NMR quantification was performed with CH_2Br_2 internal standard. The reaction crude was loaded onto silica. Purification by column chromatography on silica gel yielded the desired products.

2.4.2 General procedure for products isolated as amino alcohols

General Procedure II (GP II):

An oven-dried 10 mL Schlenk tube was equipped with a Teflon-coated stir bar, silyl-oxime carbonates **2** (0.3 mmol, 1.5 equiv.) and Ir–F (2.5 mg, 0.01 mmol, 1 mol%). The Schlenk tube was evacuated and backfilled with argon three times, before dry DCM (4 mL, 0.05 M) and alkene **1** (0.2 mmol, 1.0 equiv.) was added (if alkene was a solid, it was added prior to adding the solvent). The Schlenk tube was set under argon atmosphere and irradiated under 405 nm irradiation for 12 h at room temperature. Upon completion, the solvent was evaporated and ^{1}H NMR quantification was performed with CH_2Br_2 internal standard. The solvents were evaporated and the reaction crude was dissolved in 2.5 mL MeOH and stirred for 5 minutes. To this, 2.5 mL 1.0 M HCl was added and stirred at room temperature to get desired deprotection. Most of the solvent was evaporated and re-dissolved in MeOH to load on the column. Purification by column chromatography on silica gel yielded the desired amino alcohol as hydrochloride salts.

2.5 Synthesis of 1,3-amino alcohols

2.5.1 Silyl-oxime carbonate scope

4-((Dimethyl(phenyl)silyl)oxy)-2-((diphenylmethylene)amino)butanenitrile (3a)

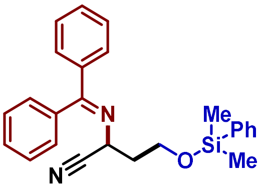

Was synthesized according to **GP I** using diphenylmethanone O-(((dimethyl(phenyl)silyl)methoxy)carbonyl) oxime **2a** (116.0 mg, 0.3 mmol, 1.5 equiv.) and acrylonitrile **1a** (10.6 mg, 0.2 mmol, 1.0 equiv.). Purification by column chromatography on silica gel (pentane/Et_2O = 95/5 to 90/10) afforded the desired product (62.1 mg, 0.16 mmol, 78%) as a colorless liquid. $\mathbf{R_f}$ (pentane/Et_2O = 95/5) = 0.3.

^{1}H NMR (400 MHz, $CDCl_3$) δ 7.64 – 7.58 (m, 2H), 7.50 – 7.42 (m, 6H), 7.39 – 7.31 (m, 5H), 7.23 – 7.17 (m, 2H), 4.49 (t, J = 7.0 Hz, 1H), 3.79 – 3.65 (m, 2H), 2.21 – 2.08 (m, 2H), 0.28 (s, 6H).

^{13}C NMR (101 MHz, CDCl3) δ 174.1, 139.0, 137.7, 135.7, 133.8, 131.6, 130.2, 129.7, 129.4, 129.3, 128.7, 128.3, 127.9, 120.1, 58.8, 50.1, 38.0, -1.6.

HRMS (ESI$^+$): $[M+Na]^+$ 421.1707; found 421.1707.

2-((Diphenylmethylene)amino)-4-((trimethylsilyl)oxy)butanenitrile (3b)

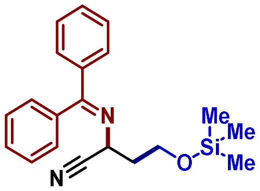

Was synthesized according to **GP I** using diphenylmethanone O-(((trimethylsilyl)methoxy)carbonyl) oxime **2b** (98.1 mg, 0.3 mmol, 1.5 equiv.) and acrylonitrile **1a** (10.6 mg, 0.2 mmol, 1.0 equiv.). Purification by column chromatography on silica gel (pentane/Et_2O = 96/4 to 90/10) afforded the desired product (47.0 mg, 0.14 mmol, 70%) as a colorless liquid. $\mathbf{R_f}$ (pentane/Et_2O = 95/5) = 0.35.

^{1}H NMR (500 MHz, $CDCl_3$) δ 7.67 (dddd, J = 8.3, 2.2, 1.4, 0.8 Hz, 2H), 7.53 – 7.44 (m, 4H), 7.38 (dddd, J = 8.2, 7.0, 1.4, 0.7 Hz, 2H), 7.25 (ddd, J = 6.8, 1.9, 0.7 Hz, 2H), 4.50 (td, J = 7.0, 0.8 Hz, 1H), 3.79 – 3.66 (m, 2H), 2.25 – 2.07 (m, 2H), 0.04 – 0.04 (m, 9H).

^{13}C NMR (126 MHz, $CDCl_3$) δ 173.9, 139.0, 135.6, 132.8, 131.5, 130.4, 129.7, 129.4, 129.3, 128.7, 128.6, 127.9, 120.1, 58.3, 50.1, 38.1, -0.5.

HRMS (ESI$^+$): $[M+Na]^+$ 359.1551; found 359.1550.

2-((Bis(3-(trifluoromethyl)phenyl)methylene)amino)-4-((dimethyl(phenyl)silyl)oxy)butanenitrile (3d)

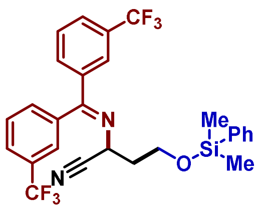

Was synthesized according to **GP I** using bis(3-(trifluoromethyl)phenyl)methanone O-(((dimethyl(phenyl)silyl)methoxy)carbonyl) oxime **2d** (157.5 mg, 0.3 mmol, 1.5 equiv.) and acrylonitrile **1a** (10.6 mg, 0.2 mmol, 1.0 equiv.). Purification by column chromatography on silica gel (pentane/Et_2O = 90/10 to 80/20) afforded the desired product (27.0 mg, 0.05 mmol, 25%) as a yellow liquid. **R_f** (pentane/Et_2O = 90/10) = 0.25.

^{1}H NMR (400 MHz, CD_2Cl_2) δ 8.02 – 7.90 (m, 2H), 7.81 (ddt, J = 7.8, 1.8, 1.0 Hz, 1H), 7.77 – 7.73 (m, 1H), 7.70 – 7.64 (m, 2H), 7.55 – 7.49 (m, 2H), 7.46 – 7.42 (m, 2H), 7.39 – 7.34 (m, 1H), 7.33 – 7.29 (m, 2H), 4.43 (t, J = 7.0 Hz, 1H), 3.78 – 3.67 (m, 2H), 2.22 – 2.08 (m, 2H), 0.27 (s, 6H).

^{13}C NMR (101 MHz, CD_2Cl_2) δ 170.8, 139.2, 137.7, 135.5, 133.7, 132.8, 131.5, 130.4, 130.1, 129.7, 129.5, 128.2, 127.0, 125.5, 124.6, 119.3, 58.7, 50.3, 37.9, 22.8, 14.2, -2.0.

^{19}F NMR (376 MHz, CD_2Cl_2) δ -61.14, -61.16.

HRMS (ESI$^+$): $[M]^+$ 534.1557; found 534.1550.

**Note: The quartets in the ^{13}C spectra cannot be distinguished well by the software, and hence all the peaks are separately picked.*

2-((Bis(4-chlorophenyl)methylene)amino)-4-((dimethyl(phenyl)silyl)oxy)butanenitrile (3e)

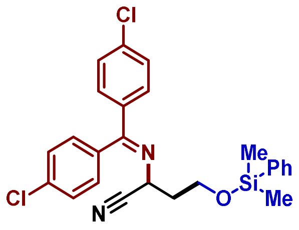

Was synthesized according to **GP I** using bis(4-chlorophenyl)methanone O-(((dimethyl(phenyl)silyl)methoxy)carbonyl) oxime **2e** (137.4 mg, 0.3 mmol, 1.5 equiv.) and acrylonitrile **1a** (10.6 mg, 0.2 mmol, 1.0 equiv.). Purification by column chromatography on silica gel (pentane/Et_2O = 90/10 to 80/20) afforded the desired product (51.3 mg, 0.11 mmol, 55%) as a brown liquid. **R_f** (pentane/Et_2O = 90/10) = 0.45.

^{1}H NMR (400 MHz, $CDCl_3$) δ 7.58 – 7.50 (m, 2H), 7.49 – 7.38 (m, 5H), 7.34 (td, J = 6.9, 1.4 Hz, 4H), 7.15 – 7.08 (m, 2H), 4.43 (dd, J = 7.6, 6.4 Hz, 1H), 3.80 – 3.63 (m, 2H), 2.22 – 2.05 (m, 2H), 0.30 (s, 3H), 0.29 (s, 3H).

^{13}C NMR (126 MHz, $CDCl_3$) δ 171.8, 138.1, 137.5, 137.0, 135.9, 133.7, 133.3, 131.7, 130.6, 130.2, 129.8, 129.3, 129.2, 129.0, 128.3, 119.7, 58.6, 49.9, 37.8, -1.7, -1.7.

HRMS (ESI$^+$): $[M+H]^+$ 467.1113; found 467.1103.

2.5.2 Alkene scope

N-(3-((Dimethyl(phenyl)silyl) oxy)-1-phenylpropyl)-1,1-diphenylmethanimine (3g)

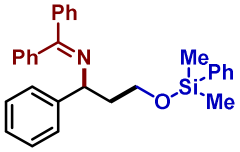

Was synthesized according to **GP I** using diphenylmethanone O-(((dimethyl(phenyl)silyl)methoxy)carbonyl) oxime **2a** (116.0 mg, 0.3 mmol, 1.5 equiv.) and styrene **1b** (20.8 mg, 0.2 mmol, 1.0 equiv.). Purification by column chromatography on silica gel (pentane/Et_2O = 98/2 to 95/5) afforded the desired product (55.5 mg, 0.12 mmol, 62%) as a colorless liquid. $\mathbf{R_f}$ (pentane/Et_2O = 95/5) = 0.5.

^{1}H NMR (400 MHz, $CDCl_3$) δ 7.81 (dd, J = 7.1, 1.7 Hz, 1H), 7.68 – 7.61 (m, 2H), 7.49 – 7.43 (m, 2H), 7.42 – 7.35 (m, 5H), 7.34 – 7.29 (m, 4H), 7.25 – 7.17 (m, 4H), 7.04 – 7.00 (m, 2H), 4.54 (dd, J = 7.9, 5.6 Hz, 1H), 3.64 – 3.48 (m, 2H), 2.22 – 2.12 (m, 1H), 2.04 (dtd, J = 12.6, 6.9, 5.6 Hz, 1H), 0.29 (s, 3H), 0.28 (s, 3H).

^{13}C NMR (126 MHz, $CDCl_3$) δ 167.5, 145.2, 140.6, 137.5, 133.9, 132.9, 130.5, 130.3, 129.9, 129.0, 128.7, 128.7, 128.4, 128.3, 128.2, 127.6, 127.0, 63.5, 60.6, 42.5, -1.2, -1.4.

HRMS (ESI^+): $[M+Na]^+$ 472.2073; found 472.2067.

3-Amino-3-phenylpropan-1-ol hydrochloride (3g')

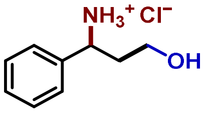

Was synthesized according to **GP II** using diphenylmethanone O-(((dimethyl(phenyl)silyl)methoxy)carbonyl) oxime **2a** (116.0 mg, 0.3 mmol, 1.5 equiv.) and styrene **1b** (20.8 mg, 0.2 mmol, 1.0 equiv.). Purification by column chromatography on silica gel (DCM/MeOH = 98/2 to 90/10) afforded the desired product (22.0 mg, 0.11 mmol, 57%) as a colourless solid. $\mathbf{R_f}$ (DCM/MeOH = 95/5) = 0.3.

^{1}H NMR (400 MHz, D_2O) δ 7.53 – 7.44 (m, 5H), 4.51 (dd, J = 8.8, 6.2 Hz, 1H), 3.67 – 3.57 (m, 1H), 3.48 (ddd, J = 11.4, 8.3, 5.4 Hz, 1H), 2.33 – 2.14 (m, 2H).

^{13}C NMR (101 MHz, D_2O) δ 135.8, 129.5, 129.4, 127.3, 58.0, 53.3, 35.5.

HRMS (ESI^+): $[M-Cl]^+$ 152.1075; found 152.1069.

**Note: The NH_2 and OH proton peaks cannot be seen possible due to proton exchange with solvent.*

N-(3-((Dimethyl(phenyl)silyl)oxy)-1-(4-methoxyphenyl)propyl)-1,1-diphenylmethanimine (3h)

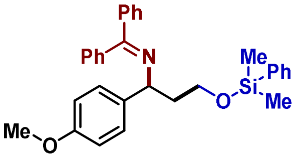

Was synthesized according to **GP I** using diphenylmethanone O-(((dimethyl(phenyl)silyl)methoxy)carbonyl) oxime **2a** (116.0 mg, 0.3 mmol, 1.5 equiv.) and 1-methoxy-4-vinylbenzene **1c** (26.8 mg, 0.2 mmol, 1.0 equiv.). Purification by column chromatography on silica gel (pentane/Et_2O = 97/3 to 94/6) afforded the desired product (65.0 mg, 0.14 mmol, 68%) as a yellow liquid. **R_f** (pentane/Et_2O = 95/5) = 0.4.

^{1}H NMR (400 MHz, $CDCl_3$) δ 7.85 – 7.61 (m, 3H), 7.51 – 7.45 (m, 2H), 7.43 – 7.37 (m, 4H), 7.35 – 7.29 (m, 4H), 7.15 – 7.10 (m, 2H), 7.07 – 7.01 (m, 2H), 6.85 – 6.76 (m, 2H), 4.50 (dd, J = 7.7, 5.8 Hz, 1H), 3.78 (s, 3H), 3.63 – 3.47 (m, 2H), 2.18 – 2.10 (m, 1H), 2.03 (dt, J = 13.3, 6.5 Hz, 1H), 0.29 (d, J = 2.7 Hz, 6H).

^{13}C NMR (126 MHz, $CDCl_3$) δ 166.5, 158.1, 140.0, 137.9, 136.9, 133.3, 132.2, 129.9, 129.6, 129.3, 128.3, 128.1, 128.0, 127.8, 127.7, 127.6, 113.4, 62.2, 60.0, 55.0, 41.9, -1.8, -2.0.

HRMS (ESI$^+$): $[M+Na]^+$ 502.2178; found 502.2174.

**Note: There are some unavoidable Si impurities in the ^{1}H spectra – which could not be separated even after rounds of washing the compound with pentane.*

4-(3-((Dimethyl(phenyl)silyl)oxy)-1-((diphenylmethylene)amino)propyl)phenyl acetate (3i)

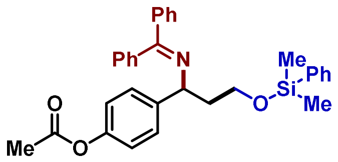

Was synthesized according to **GP I** using diphenylmethanone O-(((dimethyl(phenyl)silyl)methoxy)carbonyl) oxime **2a** (116.0 mg, 0.3 mmol, 1.5 equiv.) and 4-vinylphenyl acetate **1d** (32.4 mg, 0.2 mmol, 1.0 equiv.). Purification by column chromatography on silica gel (pentane/Et_2O = 97/3 to 90/10) afforded the desired product (76.0 mg, 0.15 mmol, 75%) as a colourless liquid. **R_f** (pentane/Et_2O = 95/5) = 0.25.

^{1}H NMR (400 MHz, $CDCl_3$) δ 7.67 – 7.61 (m, 2H), 7.49 – 7.43 (m, 2H), 7.42 – 7.36 (m, 5H), 7.35 – 7.30 (m, 4H), 7.23 – 7.19 (m, 2H), 7.06 – 7.01 (m, 2H), 7.00 – 6.94 (m, 2H), 4.55 (dd, J = 7.8, 5.6 Hz, 1H), 3.64 – 3.48 (m, 1H), 2.29 (s, 3H), 2.14 (ddt, J = 13.8, 8.3, 6.1 Hz, 1H), 2.01 (dtd, J = 13.5, 6.8, 5.6 Hz, 1H), 0.30 (s, 3H), 0.29 (s, 3H).

^{13}C NMR (101 MHz, $CDCl_3$) δ 169.7, 167.4, 149.4, 142.4, 140.1, 138.1, 137.0, 133.6, 130.0, 129.6, 128.7, 128.4, 128.2, 128.1, 128.0, 127.9, 121.3, 62.5, 60.1, 42.2, 21.3, -1.5, -1.8.

HRMS (ESI$^+$): $[M+Na]^+$ 530.2127; found 530.2122.

**Note: There are some unavoidable Si impurities in the ^{1}H spectra – which could not be separated even after rounds of washing the compound with pentane.*

4-(3-((Dimethyl(phenyl)silyl)oxy)-1-((diphenylmethylene)amino)propyl)benzonitrile (3j)

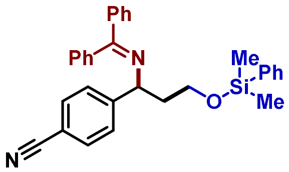

Was synthesized according to **GP I** using diphenylmethanone O-(((dimethyl(phenyl)silyl)methoxy)carbonyl) oxime **2a** (116.0 mg, 0.3 mmol, 1.5 equiv.) and 4-vinylbenzonitrile **1e** (25.8 mg, 0.2 mmol, 1.0 equiv.). Purification by column chromatography on silica gel (pentane/Et_2O = 95/5 to 92/8) afforded the desired product (62.0 mg, 0.13 mmol, 65%) as a colourless liquid. **R_f** (pentane/Et_2O = 95/5) = 0.25.

^{1}H NMR (400 MHz, $CDCl_3$) δ 7.69 – 7.62 (m, 2H), 7.54 – 7.50 (m, 2H), 7.46 – 7.39 (m, 7H), 7.36 – 7.27 (m, 6H), 6.99 (dt, J = 6.6, 1.6 Hz, 2H), 4.60 (dd, J = 7.8, 5.6 Hz, 1H), 3.51 (dt, J = 10.4, 6.0 Hz, 1H), 2.12 (ddt, J = 13.6, 7.8, 5.8 Hz, 1H), 2.04 – 1.91 (m, 1H), 0.30 (s, 3H), 0.28 (s, 3H).

^{13}C NMR (101 MHz, $CDCl_3$) δ 170.1, 152.1, 141.3, 139.3, 138.3, 135.1, 133.8, 131.9, 131.8, 131.3, 130.3, 130.2, 130.1, 130.0, 129.8, 129.6, 129.5, 129.3, 120.8, 112.0, 64.3, 61.3, 43.6, 0.0, -0.7.

HRMS (ESI$^+$): $[M+Na]^+$ 497.2025; found 497.2021.

**Note: There are some unavoidable Si impurities in the ^{1}H spectra – which could not be separated even after rounds of washing the compound with pentane.*

3-([1,1'-Biphenyl]-4-yl)-3-aminopropan-1-ol hydrochloride (3k)

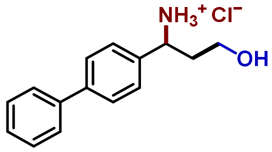

Was synthesized according to **GP II** using diphenylmethanone O-(((dimethyl(phenyl)silyl)methoxy)carbonyl) oxime **2a** (116.0 mg, 0.3 mmol, 1.5 equiv.) and 4-vinyl-1,1'-biphenyl **1f** (36.0 mg, 0.2 mmol, 1.0 equiv.). Purification by column chromatography on silica gel (DCM/MeOH = 95/5 to 90/10) afforded the desired product (25.0 mg, 0.11 mmol, 48%) as a colorless solid. **R_f** (DCM/MeOH = 95/5) = 0.3.

^{1}H NMR (400 MHz, MeOD) δ 7.73 – 7.68 (m, 2H), 7.65 – 7.60 (m, 2H), 7.57 – 7.52 (m, 2H), 7.48 – 7.42 (m, 2H), 7.38 – 7.33 (m, 1H), 4.47 (t, J = 7.2 Hz, 1H), 3.67 (dt, J = 10.8, 5.4 Hz, 1H), 3.54 (ddd, J = 10.9, 8.4, 4.8 Hz, 1H), 3.35 (s, 3H), 2.31 – 2.20 (m, 1H), 2.12 (ddt, J = 14.0, 7.8, 4.8 Hz, 1H).

^{13}C NMR (101 MHz, MeOD) δ 143.2, 141.5, 138.1, 130.0, 128.8, 128.7, 128.7, 128.0, 59.3, 54.6, 38.3.

HRMS (ESI$^+$): $[M-Cl]^+$ 228.1388; found 228.1389.

**Note: There is a quantitative amount of 'MeOH' hydrogen-bonded to the molecule and trapped which could not be removed after 2 days of high-vacuum drying. The NH_2 and OH proton peaks cannot be seen possible due to proton exchange with solvent.*

3-Amino-3-(3-chlorophenyl)propan-1-ol hydrochloride (3l)

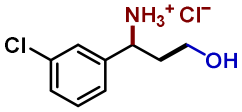

Was synthesized according to **GP II** using diphenylmethanone O-(((dimethyl(phenyl)silyl)methoxy)carbonyl) oxime **2a** (116.0 mg, 0.3 mmol, 1.5 equiv.) and 1-chloro-3-vinylbenzene **1g** (27.6 mg, 0.2 mmol, 1.0 equiv.). Purification by column chromatography on silica gel (DCM/MeOH = 95/5 to 90/10) afforded the desired product (18.0 mg, 0.08 mmol, 41%) as a colorless solid. **R_f** (DCM/MeOH = 95/5) = 0.4.

^{1}H NMR (400 MHz, MeOD) δ 7.49 (t, J = 1.9 Hz, 1H), 7.42 – 7.33 (m, 3H), 4.31 (t, J = 7.1 Hz, 1H), 3.63 (dt, J = 11.1, 5.6 Hz, 1H), 3.50 (ddd, J = 11.0, 8.0, 5.1 Hz, 1H), 2.13 (dddd, J = 13.7, 8.0, 6.9, 5.6 Hz, 1H), 1.99 (ddt, J = 14.0, 7.4, 5.3 Hz, 1H).

^{13}C NMR (101 MHz, MeOD) δ 143.8, 135.8, 131.0, 129.5, 128.7, 126.5, 59.5, 54.3, 39.4.

HRMS (ESI$^+$): $[M-Cl]^+$ 186.0686; found 186.0689.

**Note: The NH_2 and OH proton peaks cannot be seen possible due to proton exchange with solvent.*

***N*-(3-((Dimethyl(phenyl)silyl)oxy)-1-(pyridin-4-yl)propyl)-1,1-diphenylmethanimine (3m)**

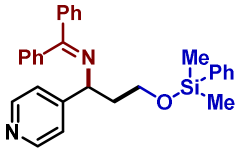

Was synthesized according to **GP I** using diphenylmethanone O-(((dimethyl(phenyl)silyl)methoxy)carbonyl) oxime **2a** (116.0 mg, 0.3 mmol, 1.5 equiv.) and 4-vinylpyridine **1h** (21.0 mg, 0.2 mmol, 1.0 equiv.). Purification by column chromatography on silica gel (pentane/EtOAc = 90/10 to 80/20) afforded the desired product (69.0 mg, 0.15 mmol, 77%) as an orange liquid. **R_f** (pentane/EtOAc = 90/10) = 0.25.

^{1}H NMR (400 MHz, $CDCl_3$) δ 8.49 – 8.42 (m, 2H), 7.71 – 7.60 (m, 2H), 7.47 – 7.43 (m, 2H), 7.43 – 7.35 (m, 6H), 7.35 – 7.30 (m, 3H), 7.14 – 7.10 (m, 2H), 7.00 (dt, J = 6.5, 1.6 Hz, 2H), 4.54 (dd, J = 8.0, 5.3 Hz, 1H), 3.67 – 3.58 (m, 1H), 3.54 (dt, J = 10.4, 6.0 Hz, 1H), 2.13 (ddt, J = 13.8, 8.0, 5.8 Hz, 1H), 2.05 – 1.93 (m, 1H), 0.31(s, 3H), 0.29(s, 3H).

^{13}C NMR (101 MHz, $CDCl_3$) δ 168.8, 153.9, 149.7, 139.7, 137.9, 136.7, 133.5, 130.3, 129.7, 128.7, 128.6, 128.5, 128.2, 128.0, 127.8, 122.5, 62.1, 59.8, 41.7, -1.6, -1.9.

HRMS (ESI$^+$): $[M+Na]^+$ 473.2025; found 473.2023.

***N*-(3-((Dimethyl(phenyl)silyl)oxy)-1-(4-methylthiazol-5-yl)propyl)-1,1-diphenylmethanimine (3n)**

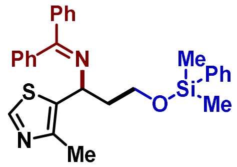

Was synthesized according to **GP I** using diphenylmethanone O-(((dimethyl(phenyl)silyl)methoxy)carbonyl) oxime **2a** (116.0 mg, 0.3 mmol, 1.5 equiv.) and 4-methyl-5-vinylthiazole **1i** (25.0 mg, 0.2 mmol, 1.0 equiv.). Purification by column chromatography on silica gel (pentane/EtOAc = 90/10 to 85/15) afforded the desired product (71.0 mg, 0.15 mmol, 75%) as a yellow liquid. **R_f** (pentane/EtOAc = 90/10) = 0.35.

^{1}H NMR (400 MHz, $CDCl_3$) δ 8.61 (s, 1H), 7.66 – 7.61 (m, 2H), 7.48 – 7.44 (m, 2H), 7.42 – 7.36 (m, 5H), 7.35 – 7.30 (m, 4H), 7.08 – 7.00 (m, 2H), 4.98 (dd, J = 7.5, 6.0 Hz, 1H), 3.62 (ddd, J = 10.4, 7.0, 5.5 Hz, 1H), 3.57 – 3.45 (m, 1H), 2.00 (s, 3H), 1.99 – 1.92 (m, 1H), 0.29 (d, J = 1.2 Hz, 6H).

^{13}C NMR (101 MHz, CDCl3) δ 168.4, 151.1, 147.1, 139.6, 138.0, 136.6, 135.5, 133.5, 130.4, 129.7, 128.8, 128.6, 128.6, 128.2, 127.9, 127.7, 59.6, 56.3, 42.7, 15.2, -1.6, -1.7.

HRMS (ESI$^+$): $[M+Na]^+$ 493.1746; found 493.1741.

***N*-(3-((Dimethyl(phenyl)silyl)oxy)-1-(perfluorophenyl)propyl)-1,1-diphenylmethanimine (3o)**

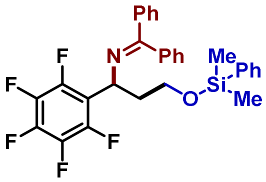

Was synthesized according to **GP I** using diphenylmethanone O-(((dimethyl(phenyl)silyl)methoxy)carbonyl) oxime **2a** (116.0 mg, 0.3 mmol, 1.5 equiv.) and 1,2,3,4,5-pentafluoro-6-vinylbenzene **1j** (39.8 mg, 0.2 mmol, 1.0 equiv.). Purification by column chromatography on silica gel (pentane/Et_2O = 95/5 to 90/10) afforded the desired product (57.0 mg, 0.10 mmol, 53%) as a colourless liquid. **R$_f$** (pentane/Et_2O = 95/5) = 0.45.

^{1}H NMR δ 7.84 – 7.61 (m, 3H), 7.43 (ddt, J = 7.3, 6.1, 1.0 Hz, 5H), 7.37 – 7.31 (m, 5H), 7.08 – 7.00 (m, 2H), 5.13 (t, J = 7.3 Hz, 1H), 3.57 (ddt, J = 38.2, 10.5, 6.1 Hz, 2H), 2.23 (q, J = 6.4 Hz, 2H), 0.26 (d, J = 1.7 Hz, 6H).

^{19}F NMR (376 MHz, $CDCl_3$) δ -140.38 – -140.52 (m), -156.95 (t, J = 21.0 Hz), -162.80 (td, J = 22.2, 7.6 Hz).

^{13}C NMR (101 MHz, $CDCl_3$) δ 170.4, 139.9, 138.1, 136.7, 133.8, 132.9, 130.9, 130.5, 130.0, 129.1, 129.0, 128.7, 128.5, 128.2, 127.7, 60.1, 54.6, 38.5, -1.6, -1.6.

HRMS (ESI$^+$): $[M+Na]^+$ 562.1602; found 562.1606.

3-((Dimethyl(phenyl)silyl)oxy)-1-((diphenylmethylene)amino)propyl propionate (3p)

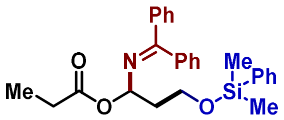

Was synthesized according to **GP I** using diphenylmethanone O-(((dimethyl(phenyl)silyl)methoxy)carbonyl) oxime **2a** (116.0 mg, 0.3 mmol, 1.5 equiv.) and vinyl propionate **1k** (20.0 mg, 0.2 mmol, 1.0 equiv.). Purification by column chromatography on silica gel (pentane/Et_2O = 95/5 to 90/10) afforded the desired product (36.0 mg, 0.10 mmol, 40%) as a pale-yellow liquid. **R$_f$** (pentane/Et_2O = 90/10) = 0.25.

^{1}H NMR (400 MHz, $CDCl_3$) δ 7.63 – 7.59 (m, 2H), 7.49 – 7.44 (m, 2H), 7.42 – 7.29 (m, 9H), 7.18 – 7.15 (m, 2H), 4.26 – 4.21 (m, 1H), 4.12 (dd, J = 7.1, 4.4 Hz, 2H), 3.65 (qdd, J = 10.3, 7.1, 5.6 Hz, 2H), 2.26 – 2.03 (m, 2H), 1.22 (t, J = 7.1 Hz, 3H), 0.29 (d, J = 2.8 Hz, 6H).

^{13}C NMR (101 MHz, $CDCl_3$) δ 172.2, 170.8, 139.6, 137.7, 136.3, 133.3, 130.2, 129.4, 128.7, 128.5, 128.3, 127.9, 127.8, 127.7, 62.0, 61.0, 59.4, 36.3, 14.1, -1.9, -2.0.

HRMS (ESI$^+$): $[M+Na]^+$ 468.1971; found 468.1965.

Dimethyl-(3-((dimethyl(phenyl)silyl)oxy)-1-((diphenylmethylene)amino)propyl)phosphonate (3q)

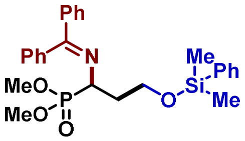

Was synthesized according to **GP I** using diphenylmethanone O-(((dimethyl(phenyl)silyl)methoxy)carbonyl) oxime **2a** (116.0 mg, 0.3 mmol, 1.5 equiv.) and dimethyl vinylphosphonate **1l** (33.2 mg, 0.2 mmol, 1.0 equiv.). Purification by column chromatography on silica gel (pentane/Et_2O = 95/5 to 85/15) afforded the desired product (42.0 mg, 0.09 mmol, 44%) as a colourless liquid. **R$_f$** (pentane/Et_2O = 90/10) = 0.30.

^{1}H NMR (400 MHz, $CDCl_3$) δ 7.57 – 7.52 (m, 2H), 7.41 – 7.36 (m, 2H), 7.33 – 7.27 (m, 5H), 7.27 – 7.21 (m, 4H), 7.19 – 7.14 (m, 2H), 4.08 (ddd, J = 12.4, 9.5, 3.8 Hz, 1H), 3.65 (dd, J = 10.5, 8.8 Hz, 6H), 3.58 – 3.51 (m, 1H), 3.46 – 3.35 (m, 1H), 2.18 – 1.99 (m, 2H), 0.22 (s, 3H), 0.21(s, 3H).

^{31}P NMR (162 MHz, $CDCl_3$) δ 17.28.

^{13}C NMR (101 MHz, $CDCl_3$) δ 171.5 (d, J = 16.0 Hz), 139.7 (d, J = 3.8 Hz), 137.9, 135.8 (d, J = 2.7 Hz), 133.5, 130.4, 129.7, 128.9 (d, J = 1.7 Hz), 128.7, 128.4, 128.4, 128.1, 127.9, 59.7 (d, J = 16.4 Hz), 58.7, 57.1, 53.3 (t, J = 6.7 Hz), 34.1 (d, J = 4.9 Hz), -1.6, -1.8 (d, J = 2.1 Hz).

HRMS (ESI$^+$): [M+Na]$^+$ 504.1736; found 504.1730.

Prop-2-yn-1-yl 4-((dimethyl(phenyl)silyl)oxy)-2-((diphenylmethylene)amino)butanoate (3r)

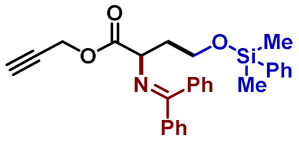

Was synthesized according to **GP I** using diphenylmethanone O-(((dimethyl(phenyl)silyl)methoxy)carbonyl) oxime **2a** (116.0 mg, 0.3 mmol, 1.5 equiv.) and prop-2-yn-1-yl acrylate **1aa** (22.0 mg, 0.2 mmol, 1.0 equiv.). Purification by column chromatography on silica gel (pentane/Et_2O = 95/5 to 90/10) afforded the desired product (25.0 mg, 0.06 mmol, 28%) as a yellow liquid. **R$_f$** (pentane/Et_2O = 95/5) = 0.20.

^{1}H NMR (400 MHz, CD_2Cl_2) δ 7.81 – 7.77 (m, 1H), 7.64 – 7.58 (m, 2H), 7.51 – 7.47 (m, 2H), 7.43 (dd, J = 5.1, 2.0 Hz, 3H), 7.38 – 7.30 (m, 5H), 7.19 – 7.13 (m, 2H), 4.65 (dd, J = 2.5, 1.1 Hz, 2H), 4.27 (dd, J = 7.7, 5.2 Hz, 1H), 3.76 – 3.56 (m, 2H), 2.52 (t, J = 2.5 Hz, 1H), 2.22 – 2.02 (m, 2H), 0.30 (s, 3H), 0.29 (s, 3H).

^{13}C NMR (101 MHz, CD_2Cl_2) δ 171.8, 171.6, 139.9, 138.3, 136.6, 133.9, 132.8, 130.8, 130.3, 129.9, 129.1, 128.9, 128.7, 128.4, 128.3, 128.1, 78.1, 75.1, 62.3, 59.6, 52.6, 36.9, -1.7, -1.8.

HRMS (ESI$^+$): [M+Na]$^+$ 478.1814; found 478.1808.

***N*-(3-((Dimethyl(phenyl)silyl)oxy)-1-(phenylsulfonyl)propyl)-1,1-diphenylmethanimine (3s)**

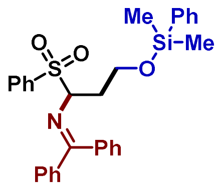

Was synthesized according to **GP I** using diphenylmethanone O-(((dimethyl(phenyl)silyl)methoxy)carbonyl) oxime **2a** (116.0 mg, 0.3 mmol, 1.5 equiv.) and (vinylsulfonyl)benzene **1m** (35.6 mg, 0.2 mmol, 1.0 equiv.). Purification by column chromatography on silica gel (pentane/Et_2O = 95/5 to 85/15) afforded the desired product (26.0 mg, 0.05 mmol, 24%) as a yellow liquid. **R$_f$** (pentane/Et_2O = 90/10) = 0.30.

^{1}H NMR (400 MHz, CD_2Cl_2) δ 7.80 – 7.77 (m, 2H), 7.66 (dt, J = 7.1, 1.4 Hz, 2H), 7.57 – 7.51 (m, 4H), 7.47 – 7.42 (m, 3H), 7.40 – 7.35 (m, 3H), 7.32 (ddd, J = 8.1, 3.4, 2.0 Hz, 4H), 6.91 (dt, J = 7.2, 1.4 Hz, 2H), 4.84 (dd, J = 9.4, 3.4 Hz, 1H), 3.70 – 3.58 (m, 1H), 3.48 (ddd, J = 10.5, 8.7, 5.2 Hz, 1H), 2.38 – 2.26 (m, 1H), 1.95 (ddt, J = 14.2, 9.8, 5.0 Hz, 1H), 0.28 (s, 3H), 0.26 (s, 3H).

^{13}C NMR (101 MHz, CD_2Cl_2) δ 174.9, 139.3, 138.0, 137.5, 135.1, 134.1, 132.8, 131.4, 130.3, 130.3, 130.0, 129.5, 129.3, 129.2, 128.7, 128.5, 128.5, 128.2, 80.6, 59.2, 34.0, -1.7, -1.9.

HRMS (ESI$^+$): [M+Na]$^+$ 536.1692; found 536.1695.

**Note: There are some aliphatic impurities in the ^{1}H and ^{13}C spectra – which could not be separated by washing the compound with pentane.*

N-(3-((Dimethyl(phenyl)silyl)oxy)-1-(methylsulfonyl)propyl)-1,1-diphenylmethanimine (3t)

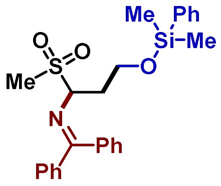

Was synthesized according to **GP I** using diphenylmethanone O-(((dimethyl(phenyl)silyl)methoxy)carbonyl) oxime **2a** (116.0 mg, 0.3 mmol, 1.5 equiv.) and (methylsulfonyl)ethene **1n** (21.2 mg, 0.2 mmol, 1.0 equiv.). Purification by column chromatography on silica gel (pentane/Et_2O = 95/5 to 85/15) afforded the desired product (43.0 mg, 0.09 mmol, 48%) as a colourless liquid. **R_f** (pentane/Et_2O = 95/5) = 0.25.

^{1}H NMR (400 MHz, CD_2Cl_2) δ 7.67 – 7.63 (m, 2H), 7.49 – 7.46 (m, 3H), 7.45 – 7.41 (m, 4H), 7.39 (d, J = 2.8 Hz, 1H), 7.37 (d, J = 1.8 Hz, 1H), 7.35 (d, J = 0.9 Hz, 1H), 7.33 (d, J = 1.6 Hz, 1H), 7.29 – 7.23 (m, 2H), 4.69 (dd, J = 9.5, 3.3 Hz, 1H), 3.67 (ddd, J = 10.8, 6.2, 4.8 Hz, 1H), 3.50 (ddd, J = 10.4, 8.6, 5.2 Hz, 1H), 2.88 (s, 3H), 2.36 (dddd, J = 13.5, 8.6, 6.2, 3.4 Hz, 1H), 2.11 (ddt, J = 13.5, 9.9, 5.0 Hz, 1H), 0.29 (s, 3H), 0.28 (s, 3H).

^{13}C NMR (101 MHz, CD_2Cl_2) δ 174.7, 139.0, 137.5, 134.8, 133.4, 131.1, 129.6, 129.1, 129.1, 128.5, 128.4, 128.2, 128.1, 127.8, 79.3, 58.7, 36.0, 34.4, -2.1, -2.3.

HRMS (ESI^+): $[M+Na]^+$ 474.1535; found 474.1529.

4-((Dimethyl(phenyl)silyl)oxy)-2-((diphenylmethylene)amino)-N,N-dimethylbutanamide (3u)

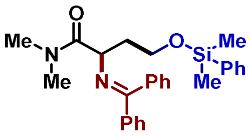

Was synthesized according to **GP I** using diphenylmethanone O-(((dimethyl(phenyl)silyl)methoxy)carbonyl) oxime **2a** (116.0 mg, 0.3 mmol, 1.5 equiv.) and *N*,*N*-dimethylacrylamide **1o** (19.8 mg, 0.2 mmol, 1.0 equiv.). Purification by column chromatography on silica gel (pentane/Et_2O = 90/10 to 65/35) afforded the desired product (49.0 mg, 0.11 mmol, 55%) as a yellow liquid. **R_f** (pentane/Et_2O = 85/15) = 0.25.

^{1}H NMR (400 MHz, CD_2Cl_2) δ 7.65 – 7.59 (m, 2H), 7.50 – 7.41 (m, 5H), 7.39 – 7.29 (m, 6H), 7.14 – 7.10 (m, 2H), 4.45 (dd, J = 7.6, 5.8 Hz, 1H), 3.73 – 3.58 (m, 2H), 2.80 (s, 3H), 2.60 (s, 3H), 2.16 – 2.05 (m, 1H), 2.05 – 1.94 (m, 1H), 0.29 (s, 3H), 0.28 (s, 3H).

^{13}C NMR (101 MHz, CD_2Cl_2) 172.6, 169.9, 140.0, 138.4, 137.4, 133.7, 130.6, 129.9, 128.9, 128.9, 128.4, 128.1, 128.1, 60.2, 37.5, 36.8, 35.9, -1.7, -1.8.

HRMS (ESI^+): $[M+Na]^+$ 467.2131; found 467.2127.

2,2,2-Trifluoroethyl -4-((dimethyl(phenyl)silyl)oxy)-2-((diphenylmethylene)amino)butanoate (3v)

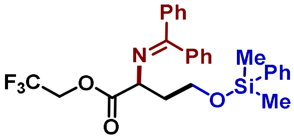

Was synthesized according to **GP I** using diphenylmethanone O-(((dimethyl(phenyl)silyl)methoxy)carbonyl) oxime **2a** (116.0 mg, 0.3 mmol, 1.5 equiv.) and 2,2,2-trifluoroethyl acrylate **1p** (30.8 mg, 0.2 mmol, 1.0

equiv.). Purification by column chromatography on silica gel (pentane/Et_2O = 90/10 to 85/15) afforded the desired product (48.0 mg, 0.09 mmol, 48%) as a colourless liquid. **R$_f$** (pentane/Et_2O = 90/10) = 0.35.

^{1}H NMR (400 MHz, CD_2Cl_2) δ 7.81 – 7.77 (m, 1H), 7.64 – 7.58 (m, 2H), 7.51 – 7.45 (m, 2H), 7.45 – 7.40 (m, 4H), 7.38 – 7.31 (m, 4H), 7.17 – 7.12 (m, 2H), 4.43 (td, J = 8.5, 2.3 Hz, 2H), 4.34 (dd, J = 7.6, 5.3 Hz, 1H), 3.69 (d, J = 5.7 Hz, 1H), 3.66 – 3.58 (m, 1H), 2.13 (ddt, J = 18.1, 7.9, 5.7 Hz, 2H), 0.30 (s, 3H), 0.29 (s, 3H).

^{19}F NMR (376 MHz, CD_2Cl_2) δ -73.85.

^{13}C NMR (101 MHz, CD_2Cl_2) δ 172.3, 171.5, 140.1, 138.4, 136.7, 134.1, 133.0, 131.2, 130.6, 130.2, 129.4, 129.2, 128.9, 128.7, 128.4, 62.3, 61.0 (q, J = 36.3 Hz), 59.7, 37.0, -1.5, -1.7.

HRMS (ESI$^+$): $[M+Na]^+$ 522.1688; found 522.1682.

3-(((Dimethyl(phenyl)silyl)oxy)methyl)-2-((diphenylmethylene)amino)cyclohexan-1-one (3w)

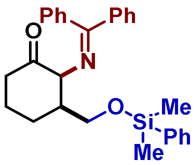

Was synthesized according to **GP I** using diphenylmethanone O-(((dimethyl(phenyl)silyl)methoxy)carbonyl) oxime **2a** (116.0 mg, 0.3 mmol, 1.5 equiv.) and cyclohex-2-en-1-one **1q** (19.2 mg, 0.2 mmol, 1.0 equiv.). Purification by column chromatography on silica gel (pentane/Et_2O = 97/3 to 90/10) afforded the desired product (58.0 mg, 0.13 mmol, 66%, d.r. >95:5) as a colourless oil. **R$_f$** (pentane/Et_2O = 95/5) = 0.35.

^{1}H NMR (400 MHz, $CDCl_3$) δ 7.61 (dt, J = 6.9, 1.5 Hz, 2H), 7.52 – 7.47 (m, 2H), 7.37 (dq, J = 7.1, 2.1 Hz, 3H), 7.32 (dddd, J = 8.1, 6.7, 4.8, 2.4 Hz, 6H), 7.08 – 7.04 (m, 2H), 3.90 (d, J = 10.9 Hz, 1H), 3.64 – 3.52 (m, 2H), 2.49 – 2.37 (m, 2H), 2.14 – 1.99 (m, 2H), 1.97 – 1.87 (m, 1H), 1.74 (dt, J = 13.0, 3.7 Hz, 1H), 1.63 (td, J = 12.5, 3.4 Hz, 1H), 0.33 (d, J = 2.3 Hz, 6H).

^{13}C NMR (101 MHz, $CDCl_3$) δ 209.7, 172.7, 141.3, 139.5, 138.5, 135.2, 131.9, 131.3, 130.5, 130.2, 130.0, 129.7, 129.6, 129.3, 73.3, 66.0, 49.7, 42.6, 28.7, 26.9, 0.0, -0.1.

HRMS (ESI$^+$): $[M+Na]^+$ 464.2022; found 464.2018.

3-(((Dimethyl(phenyl)silyl)oxy)methyl)-4-((diphenylmethylene)amino)-1-phenylpyrrolidine-2,5-dione (3x)

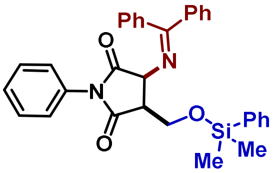

Was synthesized according to **GP I** using diphenylmethanone O-(((dimethyl(phenyl)silyl)methoxy)carbonyl) oxime **2a** (116.0 mg, 0.3 mmol, 1.5 equiv.) and 1-phenyl-1*H*-pyrrole-2,5-dione **1r** (34.6 mg, 0.2 mmol, 1.0 equiv.). Purification by column chromatography on silica gel (pentane/Et_2O = 90/10) afforded the desired product (77.0 mg, 0.15 mmol, 75%, d.r. = 50:50) as a colourless oil. **R$_f$** (pentane/Et_2O = 90/10) = 0.25.

^{1}H NMR (400 MHz, CD_2Cl_2, *mixture of diastereomers - 50:50*) δ 7.71 – 7.64 (m, 4H), 7.53 – 7.47 (m, 8H), 7.47 – 7.43 (m, 4H), 7.42 – 7.35 (m, 16H), 7.34 – 7.30 (m, 4H), 7.29 – 7.25 (m, 4H), 4.83 (d, J = 4.7 Hz,

1H), 4.14 (d, J = 3.4 Hz, 1H), 4.11 (d, J = 3.4 Hz, 2H), 3.65 (dd, J = 10.2, 3.0 Hz, 2H), 3.28 (dt, J = 4.6, 3.2 Hz, 2H), 0.21 (s, 6H), 0.19 (s, 6H).

^{13}C NMR (101 MHz, CD_2Cl_2) δ 175.9, 175.1, 173.9, 139.5, 137.5, 136.0, 133.7, 132.8, 131.2, 130.1, 129.5, 129.4, 129.3, 129.1, 128.9, 128.9, 128.5, 128.2, 127.0, 63.7, 60.0, 52.1, -1.9, -2.1.

HRMS (ESI$^+$): $[M+Na]^+$ 541.1923; found 541.1920.

2-(((Dimethyl(phenyl)silyl)oxy)methyl)-3-((diphenylmethylene)amino)succinonitrile (3y)

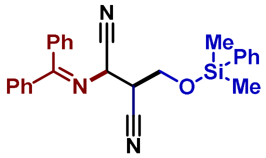

Was synthesized according to **GP I** using diphenylmethanone O-(((dimethyl(phenyl)silyl)methoxy)carbonyl) oxime **2a** (116.0 mg, 0.3 mmol, 1.5 equiv.) and fumaronitrile **1s** (15.6 mg, 0.2 mmol, 1.0 equiv.). Purification by column chromatography on silica gel (pentane/Et_2O = 95/5) afforded the desired product (46.0 mg, 0.11 mmol, 55%, d.r. = 50:50) as a yellow gum. **R_f** (pentane/Et_2O = 96/4) = 0.35.

^{1}H NMR (599 MHz, CD_2Cl_2, *mixture of diastereomers - 50:50*) δ 7.80 – 7.66 (m, 2H), 7.64 (d, J = 1.6 Hz, 2H), 7.56 – 7.50 (m, 8H), 7.48 – 7.44 (m, 4H), 7.43 – 7.38 (m, 6H), 7.35 (dt, J = 7.5, 1.2 Hz, 4H), 7.23 (ddd, J = 21.0, 6.4, 1.8 Hz, 4H), 4.65 (d, J = 3.3 Hz, 1H), 4.64 (d, J = 2.5 Hz, 1H), 3.96 – 3.85 (m, 4H), 3.33 – 3.24 (m, 2H), 0.34 (t, J = 7.9 Hz, 12H).

^{13}C NMR (151 MHz, CD_2Cl_2) δ 177.1, 176.8, 138.3, 136.6, 135.0, 133.7, 132.8, 132.2, 130.4, 130.4, 130.3, 130.2, 130.1, 129.7, 129.6, 129.5, 129.5, 128.7, 128.7, 128.4, 128.4, 127.9, 127.7, 117.4, 117.0, 59.9, 59.8, 51.5, 50.8, 40.4, 40.2, -2.0, -2.1, -2.1, -2.2.

HRMS (ESI$^+$): $[M+Na]^+$ 446.1665; found 446.1661.

3-Amino-3-(2-hydroxyethyl)dihydrofuran-2(3*H*)-one hydrochloride (3z)

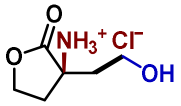

Was synthesized according to **GP II** using diphenylmethanone O-(((dimethyl(phenyl)silyl)methoxy)carbonyl) oxime **2a** (116.0 mg, 0.3 mmol, 1.5 equiv.) and 3-methylenedihydrofuran-2(3*H*)-one **1t** (19.6 mg, 0.2 mmol, 1.0 equiv.). Purification by column chromatography on silica gel (DCM/MeOH = 95/5) afforded the desired product (18.0 mg, 0.10 mmol, 51%) as a yellow solid. **R_f** (DCM/MeOH = 95/5) = 0.25.

^{1}H NMR (400 MHz, MeOD) δ 4.37 (ddd, J = 9.1, 8.4, 4.4 Hz, 1H), 4.28 (ddd, J = 9.2, 7.9, 7.2 Hz, 1H), 3.76 (t, J = 6.4 Hz, 2H), 2.44 (ddd, J = 13.1, 7.2, 4.4 Hz, 1H), 2.20 (dt, J = 13.1, 8.2 Hz, 1H), 1.89 (td, J = 6.5, 3.8 Hz, 2H).

^{13}C NMR (101 MHz, MeOD) δ 182.1, 66.0, 58.5, 57.7, 39.6, 35.7.

HRMS (ESI$^+$): $[M-Cl]^+$ 146.0817; found 146.0814.

**Note: The NH_2 and OH proton peaks cannot be seen possible due to proton exchange with solvent.*

4-((Dimethyl(phenyl)silyl)oxy)-2-((diphenylmethylene)amino)-2-methylbutanenitrile (3aa)

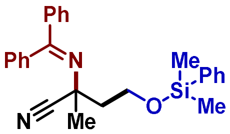

Was synthesized according to **GP I** using diphenylmethanone O-(((dimethyl(phenyl)silyl)methoxy)carbonyl) oxime **2a** (116.0 mg, 0.3 mmol, 1.5 equiv.) and methacrylonitrile **1u** (16.6 mg, 0.2 mmol, 1.0 equiv.). Purification by column chromatography on silica gel (pentane/Et_2O = 98/2 to 95/5) afforded the desired product (59.0 mg, 0.14 mmol, 72%) as a colourless gum. **R_f** (pentane/Et_2O = 96/4) = 0.3.

^{1}H NMR (400 MHz, $CDCl_3$) δ 7.61 – 7.57 (m, 2H), 7.55 – 7.43 (m, 5H), 7.41 – 7.35 (m, 4H), 7.35 – 7.30 (m, 2H), 7.22 – 7.18 (m, 2H), 3.88 (ddd, J = 8.0, 6.2, 3.6 Hz, 2H), 2.30 (dd, J = 7.7, 5.9 Hz, 1H), 2.25 – 2.17 (m, 1H), 0.40 (d, J = 1.5 Hz, 6H).

^{13}C NMR (101 MHz, $CDCl_3$) δ 168.4, 140.0, 137.7, 135.3, 133.6, 133.2, 130.8, 130.2, 129.8, 129.7, 128.7, 128.6, 128.3, 128.2, 128.0, 120.3, 59.3, 55.5, 46.4, 29.4, -1.6, -1.8.

HRMS (ESI$^+$): $[M+Na]^+$ 435.1869; found 435.1863.

**Note: There are some aliphatic Si impurities in the ^{1}H and ^{13}C spectra – which could not be separated even after rounds of washing the compound with pentane.*

_N_-(4-((Dimethyl(phenyl)silyl)oxy)-1,1,1-trifluoro-2-(naphthalen-1-yl)butan-2-yl)-1,1-diphenylmethanimine (3ab)

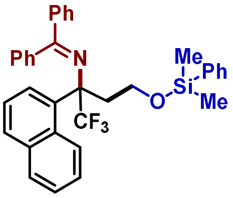

Was synthesized according to **GP I** using diphenylmethanone O-(((dimethyl(phenyl)silyl)methoxy)carbonyl) oxime **2a** (116.0 mg, 0.3 mmol, 1.5 equiv.) and 1-(3,3,3-trifluoroprop-1-en-2-yl)naphthalene **1ab** (47.6 mg, 0.2 mmol, 1.0 equiv.). Purification by column chromatography on silica gel (pentane/Et_2O = 98/2 to 96/4) afforded the desired product (61.0 mg, 0.10 mmol, 54%) as a colourless gum. **R_f** (pentane/Et_2O = 96/4) = 0.45.

^{1}H NMR (400 MHz, CD_2Cl_2) δ 8.36 (dd, J = 8.6, 2.5 Hz, 1H), 7.83 (dd, J = 8.1, 1.5 Hz, 1H), 7.76 (dt, J = 7.9, 1.1 Hz, 1H), 7.57 (ddt, J = 7.0, 5.1, 1.6 Hz, 4H), 7.45 – 7.39 (m, 2H), 7.34 (dddd, J = 7.6, 6.6, 5.0, 1.9 Hz, 6H), 7.24 – 7.11 (m, 2H), 7.03 (tt, J = 7.5, 1.3 Hz, 1H), 6.78 (td, J = 7.4, 1.2 Hz, 2H), 5.96 (d, J = 7.5 Hz, 2H), 4.14 (tdd, J = 10.5, 5.9, 1.5 Hz, 1H), 3.91 (td, J = 10.5, 4.3 Hz, 1H), 2.84 (ddd, J = 13.8, 10.6, 5.8 Hz, 1H), 2.37 (dddd, J = 15.4, 10.7, 4.3, 1.9 Hz, 1H), 0.40 (s, 3H), 0.39 (s, 3H).

^{19}F NMR (376 MHz, CD_2Cl_2) δ -66.80.

^{13}C NMR (101 MHz, CD_2Cl_2) δ 169.1, 140.5, 138.2, 137.1, 136.9, 135.1, 133.8, 132.7, 130.9, 130.4, 130.1, 130.0, 129.4, 128.9, 128.5, 128.2, 128.2, 127.2, 127.0, 127.0, 125.8, 125.6, 124.8, 70.7 (q, J = 26.8 Hz), 59.4, 39.6, -1.8.

HRMS (ESI$^+$): $[M+Na]^+$ 590.2103; found 590.2109.

**Note: The quartets in the aromatic ^{13}C spectra were not observed even after long scans.*

Methyl 4-(4-((dimethyl(phenyl)silyl)oxy)-2-((diphenylmethylene)amino)butan-2-yl)benzoate (3ac)

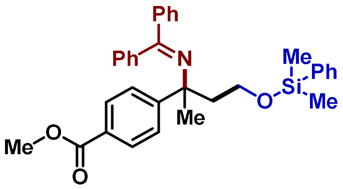

Was synthesized according to **GP I** using diphenylmethanone O-(((dimethyl(phenyl)silyl)methoxy)carbonyl) oxime **2a** (116.0 mg, 0.3 mmol, 1.5 equiv.) and methyl 4-(prop-1-en-2-yl)benzoate **1v** (35.2 mg, 0.2 mmol, 1.0 equiv.). Purification by column chromatography on silica gel (pentane/Et_2O = 98/2 to 94/6) afforded the desired product (74.0 mg, 0.14 mmol, 71%) as a yellow solid. **R_f** (pentane/Et_2O = 94/6) = 0.45.

^{1}H NMR (400 MHz, CD_2Cl_2) δ 8.04 – 7.97 (m, 2H), 7.89 – 7.81 (m, 2H), 7.77 – 7.69 (m, 2H), 7.68 – 7.59 (m, 4H), 7.40 – 7.27 (m, 5H), 7.26 – 7.21 (m, 3H), 7.14 – 7.09 (m, 1H), 4.30 (d, J = 2.8 Hz, 1H), 4.24 (dd, J = 5.0, 3.2 Hz, 1H), 3.89 (s, 3H), 2.28 – 2.16 (m, 1H), 1.99 – 1.93 (m, 1H), 1.17 (s, 3H), 0.39 (s, 6H).

^{13}C NMR (101 MHz, CD_2Cl_2) δ 167.2, 156.2, 148.3, 145.2, 133.4, 129.9, 129.0, 128.5, 128.4, 128.2, 127.5, 127.4, 127.3, 125.5, 125.3, 88.8, 59.3, 52.3, 36.0, 30.1, 0.1.

HRMS (ESI$^+$): $[M+Na]^+$ 544.2284; found 544.2279.

3-Amino-3,3-diphenylpropan-1-ol hydrochloride (3ad)

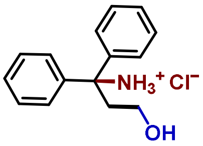

Was synthesized according to **GP II** using diphenylmethanone O-(((dimethyl(phenyl)silyl)methoxy)carbonyl) oxime **2a** (116.0 mg, 0.3 mmol, 1.5 equiv.) and ethene-1,1-diyldibenzene **1w** (36.0 mg, 0.2 mmol, 1.0 equiv.). Purification by column chromatography on silica gel (DCM/MeOH = 90/10) afforded the desired product (47.0 mg, 0.17 mmol, 89%) as an orange solid. **R_f** (DCM/MeOH = 90/10) = 0.2.

^{1}H NMR (400 MHz, D_2O) δ 7.44 (q, J = 4.2 Hz, 6H), 7.32 (dd, J = 7.6, 2.0 Hz, 4H), 3.55 (t, J = 5.8 Hz, 2H), 2.74 (t, J = 5.8 Hz, 2H).

^{13}C NMR (101 MHz, D_2O) δ 140.0, 128.9, 128.8, 126.4, 64.6, 57.6, 38.2.

HRMS (ESI$^+$): $[M-Cl]^+$ 228.1388; found 228.1382.

**Note: The NH_2 and OH proton peaks cannot be seen possible due to proton exchange with solvent.*

Methyl 4-methyl-2,2-diphenyl-1,3-oxazinane-4-carboxylate (3ae)

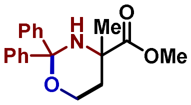

Was synthesized according to **GP I** using diphenylmethanone O-(((dimethyl(phenyl)silyl)methoxy)carbonyl) oxime **2a** (116.0 mg, 0.3 mmol, 1.5 equiv.) and methyl methacrylate **1x** (20.0 mg, 0.2 mmol, 1.0 equiv.). Purification by column chromatography on silica gel (pentane/Et_2O = 95/5 to 90/10) afforded the cyclized product (31.0 mg, 0.1 mmol, 50%) as a yellow liquid. **R_f** (pentane/Et_2O = 95/5) = 0.35.

^{1}H NMR (400 MHz, $CDCl_3$) δ 7.83 – 7.67 (m, 2H), 7.60 – 7.56 (m, 2H), 7.52 – 7.48 (m, 1H), 7.44 – 7.36 (m, 1H), 7.31 – 7.27 (m, 2H), 7.22 – 7.14 (m, 2H), 4.15 (ddd, J = 12.0, 7.6, 4.4 Hz, 1H), 4.07 – 4.00 (m, 1H), 3.62 (s, 3H), 2.87 – 2.76 (m, 1H), 2.05 (ddd, J = 13.3, 6.5, 4.4 Hz, 1H), 1.66 – 1.58 (m, 1H), 1.33 (s, 3H).

^{13}C NMR (101 MHz, $CDCl_3$) δ 176.3, 145.7, 145.3, 137.8, 133.9, 132.6, 130.2, 128.4, 128.2, 127.3, 126.7, 126.2, 88.5, 59.0, 55.7, 52.3, 33.6, 27.0.

HRMS (ESI$^+$): $[M+Na]^+$ 334.1419; found 334.1417.

Di-*tert*-butyl 2-(2-((dimethyl(phenyl)silyl)oxy)ethyl)-2-((diphenylmethylene)amino)malonate (3af)

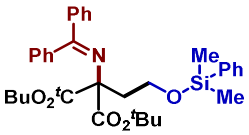

Was synthesized according to **GP I** using diphenylmethanone O-(((dimethyl(phenyl)silyl)methoxy)carbonyl) oxime **2a** (116.0 mg, 0.3 mmol, 1.5 equiv.) and di-*tert*-butyl 2-methylenemalonate **1ac** (45.6 mg, 0.2 mmol, 1.0 equiv.). Purification by column chromatography on silica gel (pentane/Et_2O = 98/2 to 94/6) afforded the desired product (74.0 mg, 0.14 mmol, 71%) as a yellow solid. **R_f** (pentane/Et_2O = 94/6) = 0.45.

^{1}H NMR (400 MHz, CD_2Cl_2) δ 7.58 – 7.55 (m, 2H), 7.54 – 7.49 (m, 2H), 7.41 – 7.28 (m, 9H), 7.19 – 7.16 (m, 2H), 3.82 – 3.73 (m, 2H), 2.35 – 2.26 (m, 2H), 1.33 (s, 18H), 0.35 (s, 6H).

^{13}C NMR (101 MHz, CD_2Cl_2) δ 168.7, 168.2, 141.7, 138.1, 137.1, 133.6, 132.5, 130.2, 129.6, 128.9, 128.8, 128.4, 128.0, 127.9, 127.9, 81.8, 74.2, 59.1, 40.6, 27.5, -2.2.

HRMS (ESI$^+$): $[M+Na]^+$ 596.2808; found 596.2809.

2-(2-((Dimethyl(phenyl)silyl)oxy)-1-phenylethyl)-2-((diphenylmethylene)amino)malononitrile (3ag)

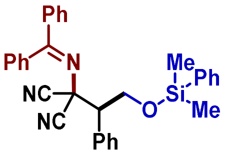

Was synthesized according to **GP I** using diphenylmethanone O-(((dimethyl(phenyl)silyl)methoxy)carbonyl) oxime **2a** (116.0 mg, 0.3 mmol, 1.5 equiv.) and 2-benzylidenemalononitrile **1y** (30.8 mg, 0.2 mmol, 1.0 equiv.). Purification by column chromatography on silica gel (pentane/Et_2O = 90/10 to 85/15) afforded the desired product (71.0 mg, 0.14 mmol, 71%) as a yellow liquid. **R_f** (pentane/Et_2O = 90/10) = 0.3.

^{1}H NMR (400 MHz, $CDCl_3$) δ 7.46 – 7.42 (m, 4H), 7.42 – 7.33 (m, 6H), 7.30 – 7.22 (m, 8H), 7.17 (dt, J = 8.3, 1.4 Hz, 2H), 4.36 (dd, J = 10.7, 6.9 Hz, 1H), 4.11 (dd, J = 10.7, 6.6 Hz, 1H), 3.73 (t, J = 6.7 Hz, 1H), 0.28 (d, J = 0.7 Hz, 6H).

^{13}C NMR (101 MHz, $CDCl_3$) δ 176.4, 138.9, 137.1, 135.1, 133.8, 133.7, 132.2, 130.7, 130.0, 129.9, 129.5, 128.8, 128.7, 128.6, 128.4, 128.0, 114.0, 113.4, 62.9, 58.2, 55.6, -2.0, -2.0.

HRMS (ESI$^+$): $[M+Na]^+$ 522.1978; found 522.1972.

2-(1-((Dimethyl(phenyl)silyl)oxy)-2-methylpropan-2-yl)-2-((diphenylmethylene)amino)malononitrile (3ah)

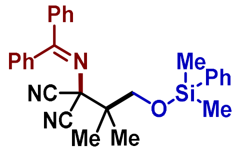

Was synthesized according to **GP I** using diphenylmethanone O-(((dimethyl(phenyl)silyl)methoxy)carbonyl) oxime **2a** (116.0 mg, 0.3 mmol, 1.5 equiv.) and 2-(propan-2-ylidene)malononitrile **1z** (21.2 mg, 0.2 mmol, 1.0 equiv.). Purification by column chromatography on silica gel (pentane/Et_2O = 90/10 to 85/15) afforded the desired product (54.0 mg, 0.12 mmol, 60%) as a yellow oil. $\mathbf{R_f}$ (pentane/Et_2O = 90/10) = 0.3.

^{1}**H NMR** (400 MHz, CD_2Cl_2) δ 7.60 – 7.48 (m, 8H), 7.44 – 7.29 (m, 7H), 3.80 (s, 2H), 1.30 (s, 6H), 0.39 (s, 6H).

^{13}**C NMR** (101 MHz, CD_2Cl_2) δ 177.1, 139.6, 137.8, 134.3, 134.0, 132.4, 130.7, 130.0, 129.8, 129.2, 128.8, 128.7, 128.2, 113.9, 67.8, 59.5, 47.1, 20.4, -2.0.

HRMS (ESI$^+$): [M+Na]$^+$ 474.1978; found 474.1973.

2.5.3 Limitations

Table T3. Unsuccessful attempts with following substrates.

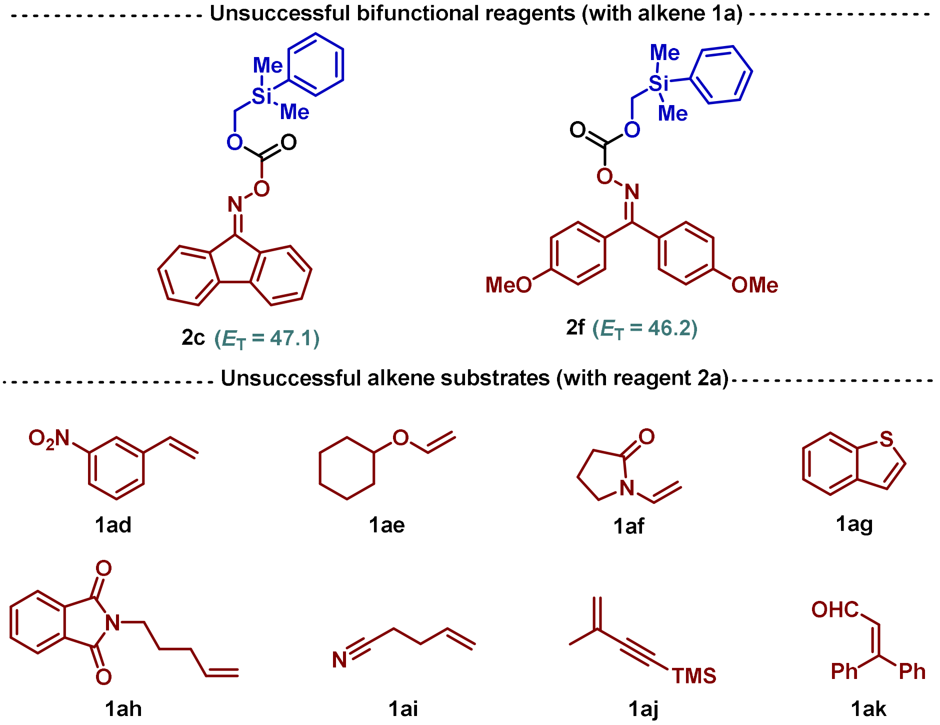

As reported in the manuscript, the unsuccessful alkenes were mostly limited to electron-rich alkenes and unactivated ones. Additionally, we saw no product using reagent **2a** with styrenic alkene with the *m*-nitro-group(**1ad**), and there was no reactivity with the aldehyde-bearing tri-substituted alkene (**1ak**).

2.6 Sensitivity assessment

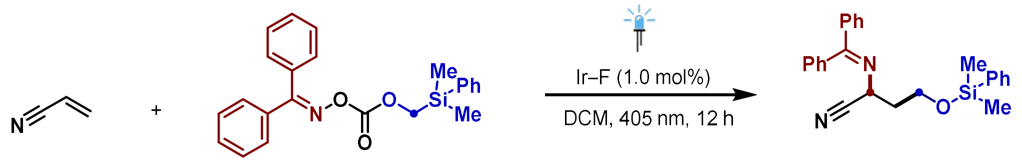

1a **2a** **3a**

1.0 quiv. **1.5 equiv.** **78%**

Table T4. Results of the sensitivity screen for the bifunctionalization. All reactions were performed on 0.1 mmol scale**.** Only entry *6*, was conducted at 1.0 mmol scale; where *n* signifies 0.1 mmol.

Entry	Parameter	Variation		Deviation
1	H_2O		$+H_2O$, $V_{H_2O} = 1\%\ V_{rxn}$	+6%
2	Concentration	Low<br>High	$V_{rxn\,-\,10\%}\,V_{rxn}$<br>$V_{rxn\,+\,10\%}\,V_{rxn}$	–3%<br>0%
3	Oxygen	Medium<br>High	Inert atmosphere<br>+air, $V_{air} = 10 * V_{rxn}$	–3%<br>+7%
4	Temperature	Low<br>High	*T* – 10 °C<br>*T* + 10 °C	–3%<br>+1%
5	Intensity	Low<br>High	32 cm<br>2 cm	–11%<br>0 %
6	Big Scale		*n* *10	–3%

The sensitivity screening, as established priorly by our group, is a test of the reaction's tolerance and generality to unoptimized conditions.[13]

As apparent from the results of Table **T3** and the radar diagram Figure. **F2**, the bifunctionalization reaction proved to be rather a broad and tolerating reaction. Irrespective of addition of water, high-oxygen infestation, increasing the reaction temperature, or lowering the solvent dilution, the yields stayed put around the optimized conditions (78%). Only for entry 5, on reducing the intensity of the light, there is a substantial decrease by 11% in the yield.

Big-scale reaction (20 times):

An oven-dried 100 mL Schlenk tube was equipped with a Teflon-coated stir bar, diphenylmethanone O-(((dimethyl(phenyl)silyl)methoxy) carbonyl) oxime **2a** (1.16 g, 3.0 mmol, 1.5 equiv.) and Ir–F (25.0 mg, 0.2 mmol, 1 mol%). The Schlenk tube was evacuated and backfilled with argon three times, before dry DCM (40 mL, 0.05 M) and acrylonitrile **1a** (107.2 mg, 2.0 mmol, 1.0 equiv.) was added. The Schlenk tube was set under argon atmosphere and irradiated under 405 nm irradiation for 12 h at rt. Upon completion, the solvent was evaporated and the reaction crude was loaded onto silica. ^{1}H NMR quantification was performed with CH_2Br_2 internal standard (^{1}H NMR yield 75%). This is 3% decrease as compared to the standard NMR yield (78%) (Fig. **F2**). The isolated yield was 70%. Characterization data given as **3a** in *section 2.4*.

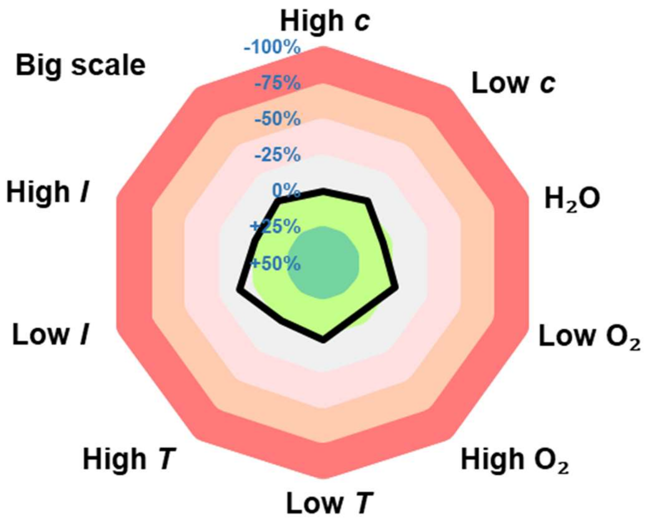

Figure F2. Radar diagram of the sensitivity screen for the bifunctionalization.

2.7 Product diversifications

2.7.1 Complete reduction of the standard product

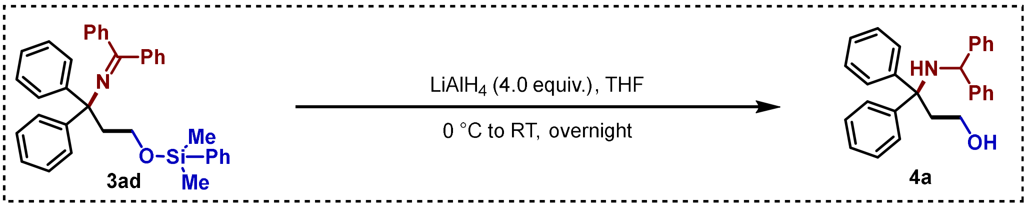

A 10 mL tube was charged with reaction crude of **3ad** (0.2 mmol), after removing solvent and re-dissolving in DCM (1mL).[10] To this, THF (2.0 mL) was added, and the solution was stirred at 0˚C. LiAlH4 (15.2 mg, 0.4 mmol) was added portion-wise to the reaction mixture over a period of 10 min, and the reaction mixture was allowed to warm to room temperature. The resulting mixture was stirred overnight. After consumption of the starting material was confirmed by TLC analysis, the reaction was quenched by H_2O (100 µL). The resulting homogenous solution was transferred to a 25 mL round bottom flask with aid of DCM (2 x 3 mL). The volatiles were removed under reduced pressure, affording a powder which was loaded on column. Purification by column chromatography (pentane/EtOAc = 90/10 to 80/20) yielded the pure corresponding γ-amino alcohol as a white solid (43 mg, yield – 55%). $\mathbf{R_f}$ (pentane/EtOAc = 90/10) = 0.2.

3-(Benzhydrylamino)-3,3-diphenylpropan-1-ol (4a)

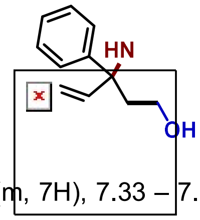

^{1}H NMR (400 MHz, $CDCl_3$) δ 7.39 – 7.34 (m, 7H), 7.33 – 7.18 (m, 13H), 4.70 (s, 1H), 3.31 (t, J = 6.6 Hz, 2H), 2.31 (t, J = 6.6 Hz, 2H).

^{13}C NMR (101 MHz, $CDCl_3$) δ 147.0, 145.4, 128.5, 128.1, 127.7, 127.4, 126.9, 126.9, 66.3, 62.0, 59.8, 40.0.

HRMS (ESI$^+$): [M+Na]$^+$ 416.1990; found 416.1999.

**Note: The NH and OH proton peaks cannot be seen possible due to proton exchange with solvent.*

2.7.2 One-step transformation of 1,3-amino alcohol to carbamate

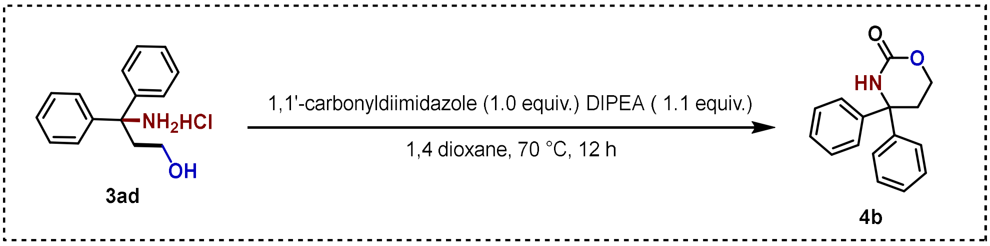

This procedure is derived from a reported procedure and optimized in this case.[14] In a 10 mL Schlenk tube, 3-amino-3,3-diphenylpropan-1-ol hydrochloride (36 mg, 0.14 mmol, 1.0 equiv.) and DIPEA (23 µL, 0.14 mmol, 1.0 equiv.) was added in dry 1,4 dioxane (1mL). To this 1,1'-carbonyldiimidazole (23 mg, 0.14 mmol, 1.1 equiv.) was added by parts. The reaction mixture was stirred with a loose cap at 70˚C overnight, and administered by TLC for reaction completion. The resultant mixture was washed with water (5mL) and the organic layer was extracted with DCM, and dried over $MgSO_4$. Purification by

column chromatography (DCM/MeOH = 95/5 to 90/10) yielded the pure carbamate as a yellow solid (13 mg, yield 50%). **R$_f$** (DCM/MeOH = 90/10) = 0.3.

4,4-Diphenyl-1,3-oxazinan-2-one (4b)

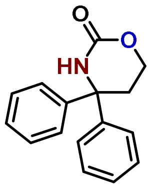

^{1}H NMR (400 MHz, MeOD) δ 7.46 – 7.27 (m, 10H), 4.14 (t, 2H), 2.73 (t, 2H).

^{13}C NMR (101 MHz, MeOD) δ 145.9, 129.6, 128.7, 127.9, 65.6, 64.0, 35.0.

HRMS (ESI$^+$): $[M+Na]^+$ 276.1000; found 276.0994.

**Note: The NH_2 and OH proton peaks cannot be seen possible due to proton exchange with solvent.*

2.7.3 One-step orthogonal functionalization of 1,3-amino alcohol to phthalimide

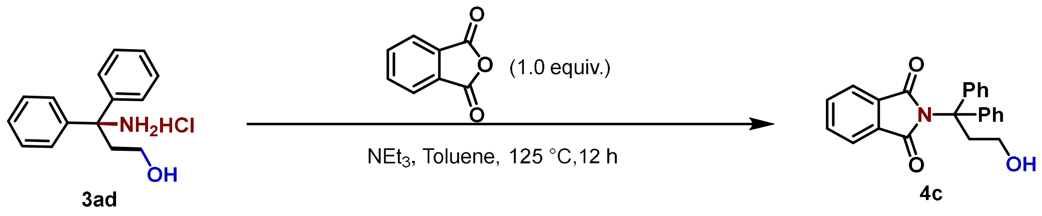

Modifying a reported literature procedure, in a 10 mL Schlenk tube, 3-amino-3,3-diphenylpropan-1-ol hydrochloride (52 mg, 0.2 mmol, 1.0 equiv.) (dissolved in MeOH) and isobenzofuran-1,3-dione (30 mg, 0.2 mmol, 1.0 equiv.) was added in dry toluene (1mL).[15] To this Et_3N (27 μL, 0.2 mmol, 1.0 equiv.) was added dropwise. The reaction mixture was stirred with a tightened cap at 125˚C overnight, and administered by TLC for reaction completion. The resultant mixture was washed with water (5mL) and the organic layer was extracted with DCM, and dried over $MgSO_4$. Purification by column chromatography (DCM/MeOH = 90/10 to 60/40) yielded the phthalimidic alcohol as a yellow oil (42 mg, yield 60%). **R$_f$** (DCM/MeOH = 80/20) = 0.25.

2-(3-Hydroxy-1,1-diphenylpropyl) isoindoline-1,3-dione (4c)

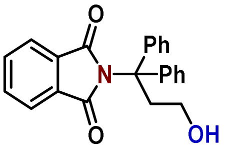

^{1}H NMR (400 MHz, MeOD) δ 7.66 (dd, J = 7.7, 1.3 Hz, 1H), 7.60 (dd, J = 7.7, 1.4 Hz, 1H), 7.50 (td, J = 7.5, 1.4 Hz, 1H), 7.44 – 7.39 (m, 5H), 7.38 – 7.33 (m, 6H), 3.55 (t, J = 5.9 Hz, 2H), 2.67 (t, J = 5.9 Hz, 2H).

^{13}C NMR (126 MHz, MeOD) δ 170.7, 143.5, 132.0, 129.6, 129.3, 129.1, 129.1, 128.7, 127.6, 65.1, 59.1, 40.6.

HRMS (ESI$^+$): $[M+Na]^+$ 380.1263; found 380.1258.

**Note: There is a quantitative amount of 'MeOH' hydrogen-bonded to the molecule and trapped which could not be removed after 2 days of high-vacuum drying. *Note: The OH proton peak cannot be seen possible due to proton exchange with solvent.*

2.7.4 One-step synthesis of non-essential amino acid homoserine

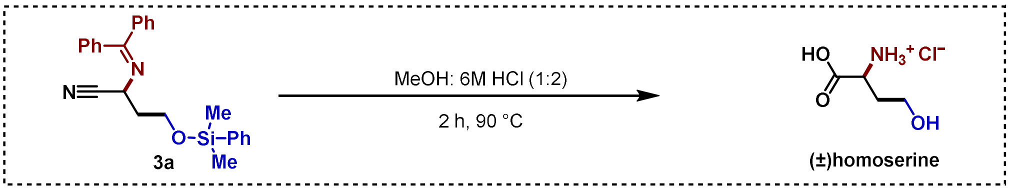

A 10 mL tube was charged with reaction crude of **3a** (0.2 mmol), after removing solvent and re-dissolving in MeOH (2.5 mL). To this, 6M HCl (5mL) was added to this, and then it was stirred for 2 h at 90 ˚C. After letting it stir for 2 h, it was cooled to room temperature and the solvents were removed by evaporation.[10] This was then dissolved in minor amounts of MeOH and to this 10 mL of DCM was added and the resultant mixture was kept in an ice-bath for white powder (10 mg, 0.06 mmol, 32%) to precipitate out. This was washed multiple times with DCM to reduce impurities. This yields the desired amino acid in decent purity (98%, with minor impurities in the aliphatic region that remain, after multiple rounds of washing).

(±)Homoserine hydrochloride (4d)

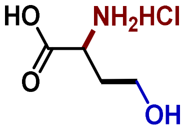

^{1}H NMR (400 MHz, MeOD) δ 4.53 (td, J = 9.1, 1.2 Hz, 1H), 4.46 – 4.32 (m, 2H), 2.75 (dddd, J = 12.5, 8.9, 6.0, 1.2 Hz, 1H), 2.36 (dtd, J = 12.5, 11.2, 9.1 Hz, 1H).

^{13}C NMR (101 MHz, MeOD) δ 172.7, 66.0, 58.1, 27.0.

HRMS (ESI$^+$): [M-Cl]$^+$ 120.0661; found 120.0663.

**Note: The NH_2, COOH and OH proton peaks cannot be seen possible due to proton exchange with solvent.*

2.8 Crystallographic data

X-Ray diffraction: Data sets for compounds **2a** and **3ad** were collected with a Bruker D8 Venture Photon III Diffractometer. Programs used: data collection: *APEX4* Version 2021.4-0 [1] (Bruker AXS Inc., **2021**); cell refinement: *SAINT* Version 8.40B (Bruker AXS Inc., **2021**); data reduction: *SAINT* Version 8.40B (Bruker AXS Inc., **2021**); absorption correction, *SADABS* Version 2016/2 (Bruker AXS Inc., **2021**); structure solution *SHELXT*-Version 2018-3 [2] (Sheldrick, G. M. *Acta Cryst.,* **2015**, *A71*, 3-8); structure refinement *SHELXL*-Version 2018-3 [3] (Sheldrick, G. M. *Acta Cryst.,* **2015**, *C71* (1), 3-8) and graphics, *XP* [4] (Version 5.1, Bruker AXS Inc., Madison, Wisconsin, USA, **1998**). *R*-values are given for observed reflections, and wR^2 values are given for all reflections.

X-ray crystal structure analysis of 2a (glo10464): A colorless, prism-like specimen of $C_{23}H_{23}NO_3Si$, approximate dimensions 0.062 mm x 0.130 mm x 0.144 mm, was used for the X-ray crystallographic analysis. The X-ray intensity data were measured (λ = 1.54178 Å). A total of 1118 frames were collected. The total exposure time was 14.49 hours. The frames were integrated with the Bruker SAINT software package using a wide-frame algorithm. The integration of the data using an orthorhombic unit cell yielded a total of 18666 reflections to a maximum θ angle of 66.67° (0.84 Å resolution), of which 3555 were independent (average redundancy 5.251, completeness = 99.0%, R_{int} = 3.45%, R_{sig} = 2.76%) and 3462 (97.38%) were greater than 2σ(F^2). The final cell constants of a = 23.7866(4) Å, b = 8.8165(2) Å, c = 9.9416(2) Å, volume = 2084.90(7) Å^3, are based upon the refinement of the XYZ-centroids of 9915 reflections above 20 σ(I) with 7.433° < 2θ < 132.9°. Data were corrected for absorption effects using the Multi-Scan method (SADABS). The ratio of minimum to maximum apparent transmission was 0.913. The calculated minimum and maximum transmission coefficients (based on crystal size) are 0.8490 and 0.9310. The structure was solved and refined using the Bruker SHELXTL Software Package, using the space group *Pca*2$_1$, with Z = 4 for the formula unit, $C_{23}H_{23}NO_3Si$. The final anisotropic full-matrix least-squares refinement on F^2 with 255 variables converged at R1 = 2.49%, for the observed data and wR2 = 6.34% for all data. The goodness-of-fit was 1.037. The largest peak in the final difference electron density synthesis was 0.236 e$^-$/Å^3 and the largest hole was -0.158 e$^-$/Å^3 with an RMS deviation of 0.034 e$^-$/Å^3. On the basis of the final model, the calculated density was 1.241 g/cm^3 and F(000), 824 e$^-$. CCDC Nr.: 2312966.

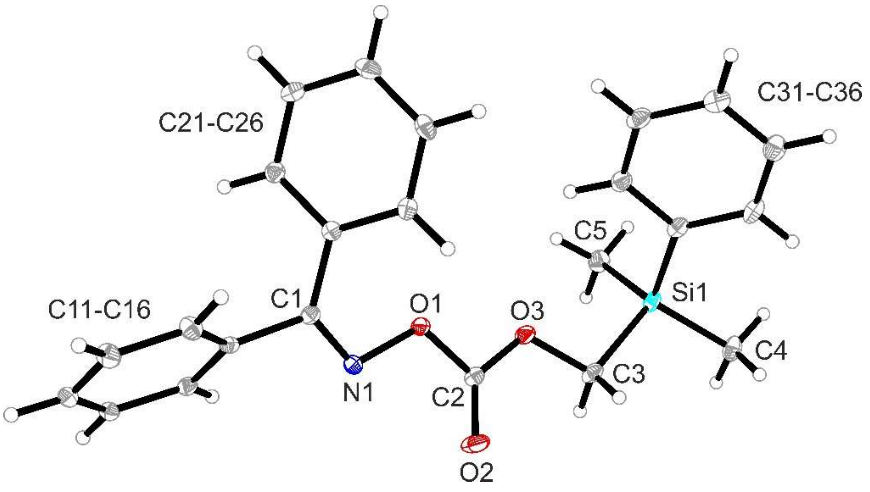

Figure S1: Crystal structure of compound **2a**.
Thermal ellipsoids are shown at 30% probability.

X-ray crystal structure analysis of 3ad (glo10515): A pale orange, plate-like specimen of $C_{15}H_{18}ClNO$, approximate dimensions 0.034 mm x 0.095 mm x 0.174 mm, was used for the X-ray crystallographic

analysis. The X-ray intensity data were measured on a single crystal diffractometer Bruker D8 Venture Photon III system equipped with a micro focus tube Cu ImS (CuKα, λ = 1.54178 Å) and a MX mirror monochromator. A total of 994 frames were collected. The total exposure time was 8.37 hours. The frames were integrated with the Bruker SAINT software package using a wide-frame algorithm. The integration of the data using a monoclinic unit cell yielded a total of 13023 reflections to a maximum θ angle of 66.61° (0.84 Å resolution), of which 2462 were independent (average redundancy 5.290, completeness = 99.1%, R_{int} = 8.04%, R_{sig} = 5.36%) and 1916 (77.82%) were greater than $2\sigma(F^2)$. The final cell constants of a = 12.7849(14) Å, b = 11.7655(13) Å, c = 9.7138(11) Å, β = 106.006(8)°, volume = 1404.5(3) $Å^3$, are based upon the refinement of the XYZ-centroids of 4538 reflections above 20 σ(I) with 7.193° < 2θ < 132.6°. Data were corrected for absorption effects using the multi-scan method (SADABS). The ratio of minimum to maximum apparent transmission was 0.852. The calculated minimum and maximum transmission coefficients (based on crystal size) are 0.6900 and 0.9260. The structure was solved and refined using the Bruker SHELXTL Software Package, using the space group $P2_1/c$, with Z = 4 for the formula unit, $C_{15}H_{18}ClNO$. The final anisotropic full-matrix least-squares refinement on F^2 with 179 variables converged at R1 = 4.27%, for the observed data and wR2 = 10.77% for all data. The goodness-of-fit was 1.051. The largest peak in the final difference electron density synthesis was 0.319 $e^-/Å^3$ and the largest hole was -0.281 $e^-/Å^3$ with an RMS deviation of 0.059 $e^-/Å^3$. On the basis of the final model, the calculated density was 1.247 g/cm^3 and F(000), 560 e^-. The hydrogen atoms at N1 and O1 were refined freely. CCDC Nr.: 2312967.

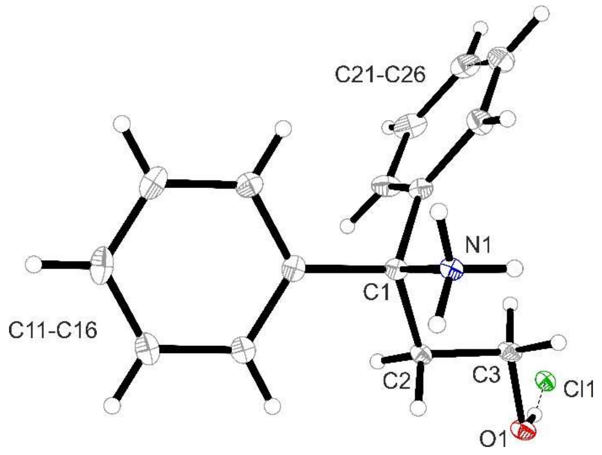

Figure S2: Crystal structure of compound **3ad**.
Thermal ellipsoids are shown at 30% probability.

References X-Ray Part:

1. Bruker AXS (**2021**) *APEX4 Version 2021.4-0*, *SAINT Version 8.40B* and *SADABS Bruker AXS area detector scaling and absorption correction Version 2016/2*, Bruker AXS Inc., Madison, Wisconsin, USA.
2. Sheldrick, G. M., *SHELXT – Integrated space-group and crystal-structure determination*, *Acta Cryst.*, **2015**, *A71*, 3-8.
3. Sheldrick, G.M., *Crystal structure refinement with SHELXL*, *Acta Cryst.*, **2015**, *C71 (1)*, 3-8.
4. Bruker AXS (**1998**) *XP – Interactive molecular graphics, Version 5.1,* Bruker AXS Inc., Madison, Wisconsin, USA.

3 Mechanistic investigations

3.1 Cyclic voltammetry studies

Linear scan voltammetry measurements were performed on a CHI600E electrochemical workstation (CH Instruments, Austin, Texas, USA) using a three-electrode cell set-up: 3 mm glassy carbon disc electrode as working electrode, Ag/AgCl (sat. KCl) as reference electrode, and a platinum wire as counter electrode.

The following parameters were set:

scan rate: 0.1 V s^{-1}, *sweep segments*: 20, *sensitivity*: 10^{-3}, V_{min}: −3.0 V, V_{max}: +3.0 V. All the measurements were taken at room temperature.

All the samples were prepared in degassed MeCN (50 mM) along with tetrabutylammonium-hexafluorophosphate (*n*-Bu_4NPF_6) as electrolyte. Prior to the CV measurement, argon was bubbled through the solution (approx. for 2 min). First, a blank run of 50 mM *n*-Bu_4NPF_6 in MeCN was performed. Next, the linear scan voltammogram for the sample with n-Bu_4NPF_6 (both 50 mM) in MeCN was recorded under the above-mentioned conditions.

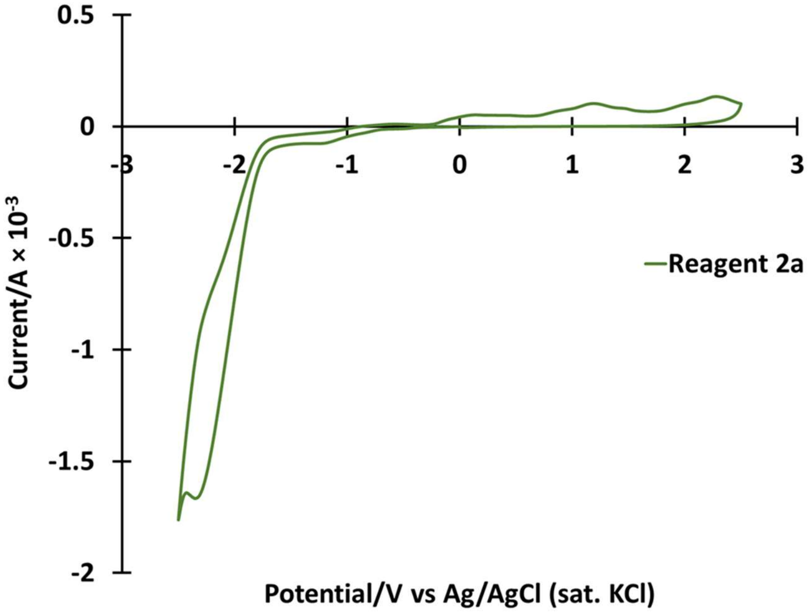

Figure F3. Linear scan voltammogram of diphenylmethanone *O*-(((dimethyl(phenyl)silyl) methoxy) carbonyl) oxime (50 mM) in MeCN (50 mM n-Bu_4NPF_6).

The linear scan voltammogram measurement shows one distinct reduction event of diphenylmethanone *O*-(((dimethyl(phenyl)silyl) methoxy) carbonyl) oxime **2a**. The reduction takes place at approx. −2.25 V vs Ag/AgCl (sat. KCl). This is clearly outside the redox window of the catalyst in operation—Ir–F photocatalyst ($E_{1/2}$(M*/M+) / V = -0.89 vs SCE).[16] Thioxanthone, a slightly more reducing photocatalyst ($E_{1/2}$(M*/M+) / V = -1.11) gives a lower yield than Ir–F,[17] these non-linear findings indicate that the pathway does not proceed by a photo-redox mechanism.

3.2 UV/Visible absorption spectroscopy

UV/Vis absorption spectra were recorded on a Jasco V-730 spectrophotometer, equipped with a temperature control unit for 25 °C. The samples were measured in Hellma fluorescence QS quarts cuvettes (volume = 1.4 mL, height = 46.0 mm, width = 12.5 cm, depth = 12.5 cm, pathlength = 1.0 cm) fitted with a OTFE stopper. The following parameters were set prior to the measurements: response time = 0.06 sec, data interval = 0.5 nm, scan speed = 1000 nm/min.

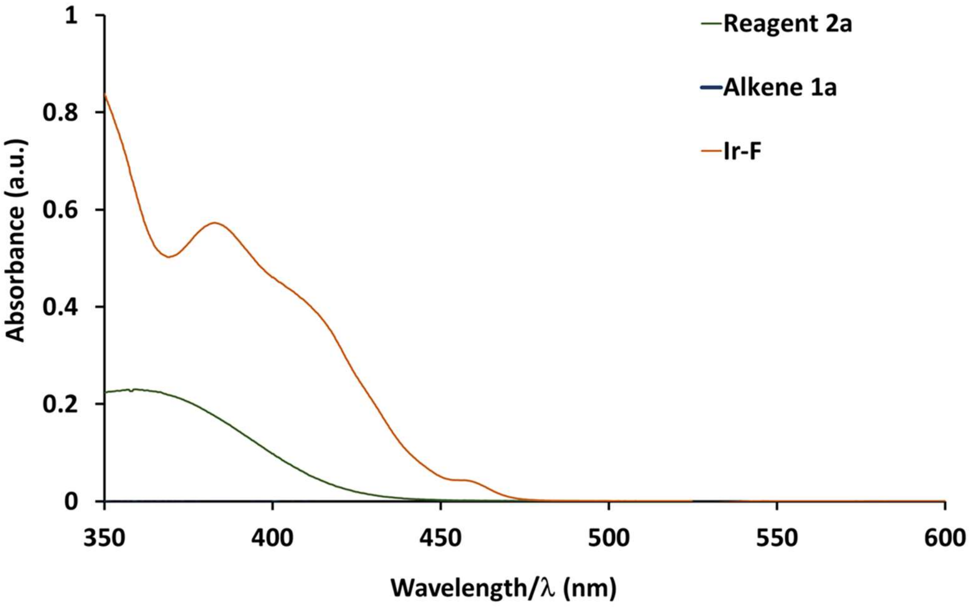

Figure F4. UV/Vis absorption spectroscopy.

The UV/vis studies as depicted in **F4** shows that in the range of 405 nm (operational wavelength), the photocatalyst Ir–F (denoted by the red line) absorbs as it is well expected. Notably, the bifunctional reagent **2a** also absorbs around the operational wavelength similar to the Ir–F, but with an evidently lower absorption coefficient. This is reconfirmed by a direct excitation experiment, without the photocatalyst in *section 3.3*.

3.3 Direct excitation

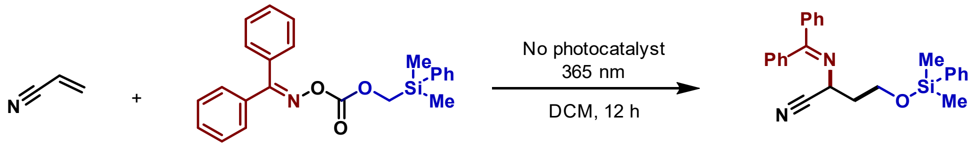

1a **2a** **3a**

1.0 equiv. **1.5 equiv.** **20% ^{1}H NMR yield**

As anticipated from Figure **F5,** we observed the formation of product (20% ^{1}H NMR yield) in the absence of photocatalyst at 365 nm, which clearly denotes the capacity of the reagent to absorb directly.

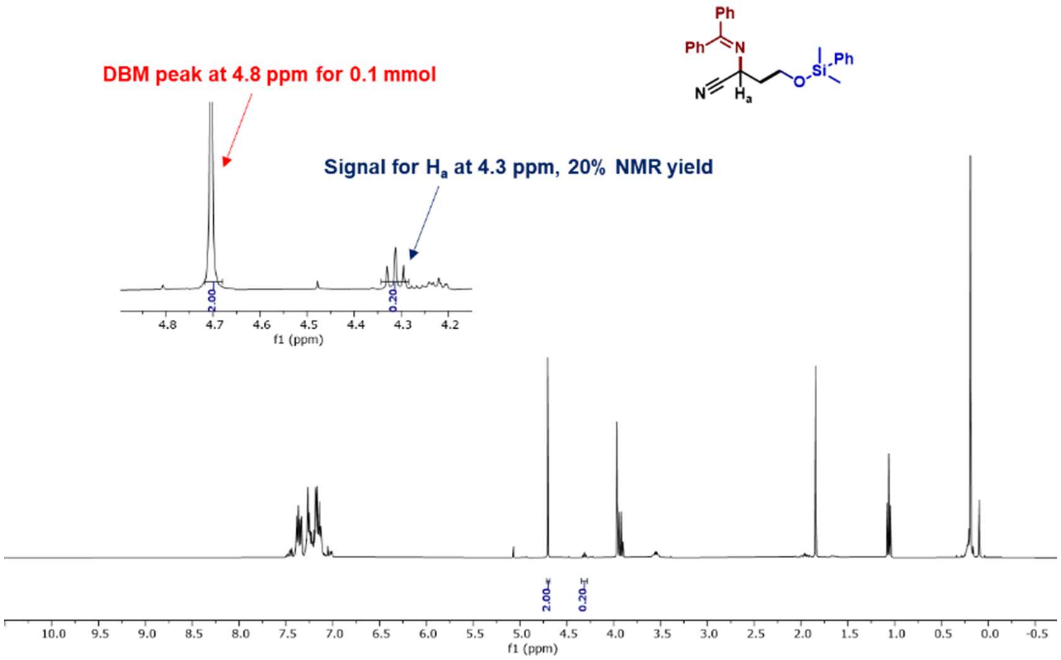

Figure F5. NMR yield for direct excitation experiment.

3.4 Cross-relationship between yield and photocatalyst triplet energies

To investigate the mode of reactivity and substrate activation, photocatalysts with different triplet energy (and redox potentials) were evaluated in a cross-relationship with the product yield for the bifunctionalization reaction.[18]

Table T5. Evaluation of various triplet photosensitizers. [a]Yield determined by ^{1}H NMR using dibromomethane as internal standard. [b]10 mol% PC used, 425 nm. [c]2 mol% PC used, 450 nm. [d]5 mol% PC used, 405 nm. Triplet energies and redox potentials (vs SCE) were adopted from the following sources.[19,17,16]

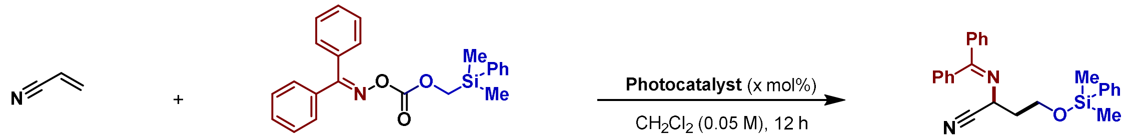

1a 1.0 equiv. **2a** 1.5 equiv. **3a**

Entry	Photocatalyst	$E_{1/2}$ (M^{-}/M*) / V	$E_{1/2}$(M*/M^{+}) / V	E_T / kcal mol^{-1}	Yield[a] / %
1[b]	[MesAcr]ClO_4	+2.08	–	44.7	n.d.
2[c]	[Ir(ppy)$_2$(dtbpy)]PF_6	+0.66	−0.96	49.2	n.d.
3[c]	*fac*-Ir(ppy)$_3$	+0.31	−1.73	58.1	n.d.
4[c]	thioxanthone	+1.18	−1.11	65.5	65
5[d]	[Ir(*d*F(CF$_3$)ppy)$_2$(dtbbpy)]PF_6	+1.21	−0.89	61.8	**78**

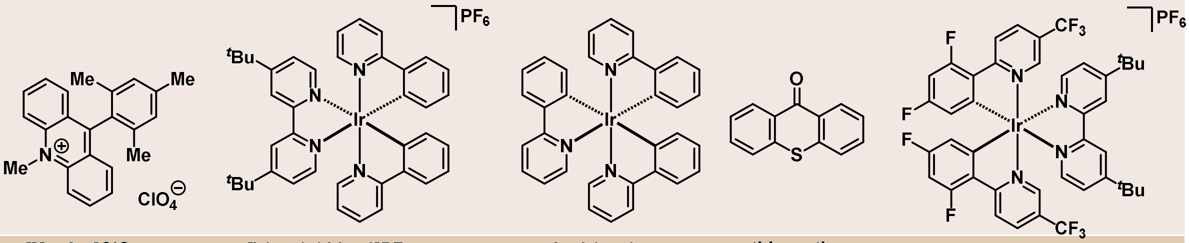

[MesAcr]ClO$_4$ **[Ir(ppy)$_2$(dtbpy)]PF$_6$** ***fac*-Ir(ppy)$_3$** **thioxanthone** **[Ir(*d*F(CF$_3$)ppy)$_2$(dtbbpy)]PF$_6$**

The above reactions were conducted in the same procedure as that in optimization with special conditions for wavelength as stated in the Table header. Table **T5** lists the correlation between triplet energy and yield of the catalytic product. Furthermore, variation of the photocatalyst´s redox potential has a negligible effect on the reaction outcome (in line with the CV studies).

3.5 Stern Volmer quenching

Stern-Volmer luminescence quenching studies were performed using a Jasco FP-8300 spectrofluorometer using Starna® fluorescence quartz cuvettes (type: 29-F, chamber volume = 1.400 mL, H × W × D = 48 mm × 12.5 mm × 12.5 mm, path length = 10 mm). The following parameters were set: data interval = 0.5 nm, scan-speed = 500 nm/min, excitation wavelength λ_{ex} = 405 nm, measured luminescence wavelength λ = 472 nm. All samples used in the luminescence quenching-based screening studies were prepared in an argon-filled glovebox with degassed and dry MeCN. Stock solutions of potential quenchers (0.025 M each) and the photocatalyst (Ir[*d*F(CF$_3$)ppy]$_2$(dtbbpy))PF$_6$ ($1\cdot10^{-4}$ M) were initially prepared. Stern-Volmer luminescence quenching studies were performed using a stock solution of the photocatalyst and variable concentrations of the potential quenchers at rt under an argon atmosphere. The samples were prepared by dilution in the 1.4 mL quartz cuvettes inside the argon-filled glovebox. The solutions were irradiated at 405 nm and the luminescence was measured at 472 nm. The ratio of I_0/I was plotted as a

function of the quencher concentration [Quencher] (I_0 = emission intensity of the photocatalyst in isolation at the specified wavelength 472 nm; I = observed emission intensity of the photocatalyst with added quencher). The following solutions in MeCN were prepared: Ir–F (1 x 10^{-4} M), diphenylmethanone O-(((dimethyl(phenyl)silyl) methoxy) carbonyl) oxime (2.5 x 10^{-2} M), acrylonitrile (2.5 x 10^{-2} M).

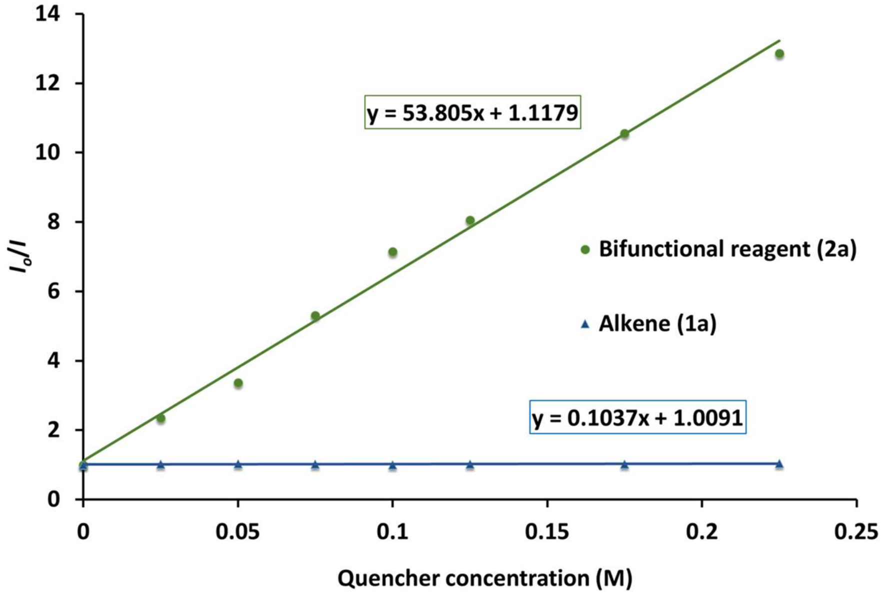

Figure F6: Stern-Volmer luminescence quenching studies using (Ir[*d*F(CF_3)ppy]$_2$(dtbbpy))PF_6 as photocatalyst.

Stern-Volmer luminescence quenching studies show that only diphenylmethanone O-(((dimethyl(phenyl)silyl) methoxy) carbonyl) oxime **2a** (green line) quenches the fluorescence of the excited photocatalyst, while acrylonitrile **1a** (blue line) is unable to quench the photocatalyst.

3.6 TEMPO-trapping experiment

To investigate the intermediacy of radicals which are involved in the presented methodology, a trapping experiment using 2,2,6,6-tetramethylpiperidine-*N*-oxyl (TEMPO) in over-stoichiometric quantities was performed.

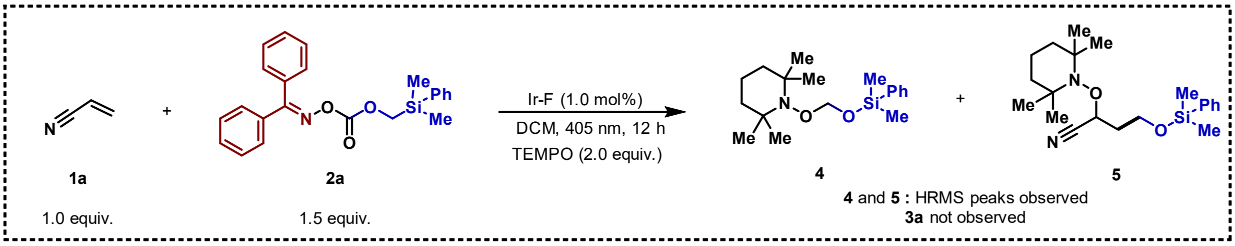

Both, the adduct of the transient C-centered methoxydimethyl (phenyl)silane radical with TEMPO, and that of the transient radical added to the alkene with TEMPO (**4** and **5** respectively), could be observed in HRMS (ESI^+): **4**: $[M+H]^+$ 322.2202, found 322.2220; **5**: $[M+H]^+$ 375.2468, found 375.2462.

No standard product formation could be observed by NMR analysis or HRMS.

Therefore, the TEMPO trapping experiment supports the intermediacy of the C-centered methoxydimethyl (phenyl)silane radical, and the absence of the adduct with the persistent iminyl radical denotes most of the iminyl radical must be involved in the bisimine formation (also detected in HRMS).

3.7 Radical initiators

To evaluate the possibility of reaction initiation by exogenous radical initiators, the addition of different (thermal) radical initiators (AIBN, DTBP) in the absence of light under heating was investigated.

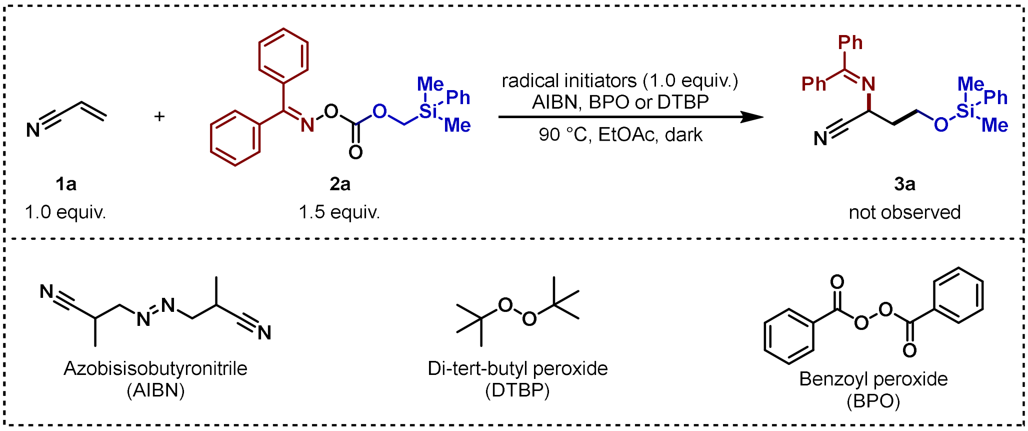

In all the 3 cases of addition of AIBN, BPO and DTBP, in dark, and without photocatalyst no product formation was detected. This rule out the possibility of reaction initiation by external radical species, in the absence of light or the photocatalyst.

3.8 Concept of transient-radical shift and Brook rearrangement

In the light of discussions of the path and progress of the reaction, and the “Energy Transfer Enabled Brook Rearrangement” mentioned in the title, we like to present a hypothesis of the shift of the initially-formed O-centered radical by the energy transfer mediated photosensitization of the N−O bifunctional reagent, to the C-centered radical by a rapid Brook rearrangement, as illustrated in Fig **F7**.[20] This kinetically-driven radical shift makes the access to the 1,3-amino alcohol products possible through a formal 1,2 addition across the alkenes.

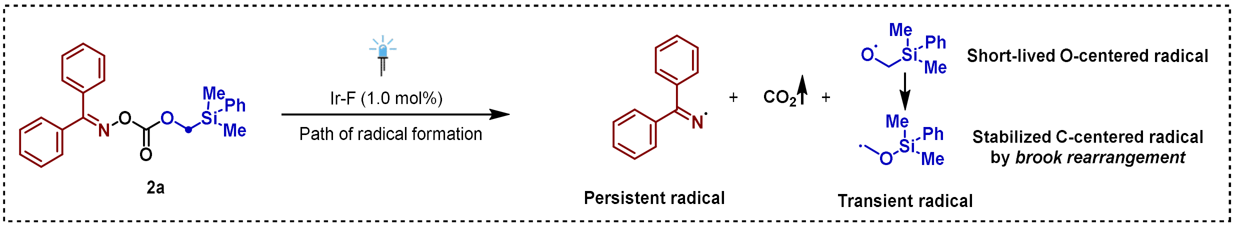

Figure F7: Radical shift hypothesis (Brook rearrangement)

This is further elaborated and explained by the calculations run for the reaction pathway in *section 4*.

3.9 Quantum yield calculations

3.9.1 Determination of the photon flux

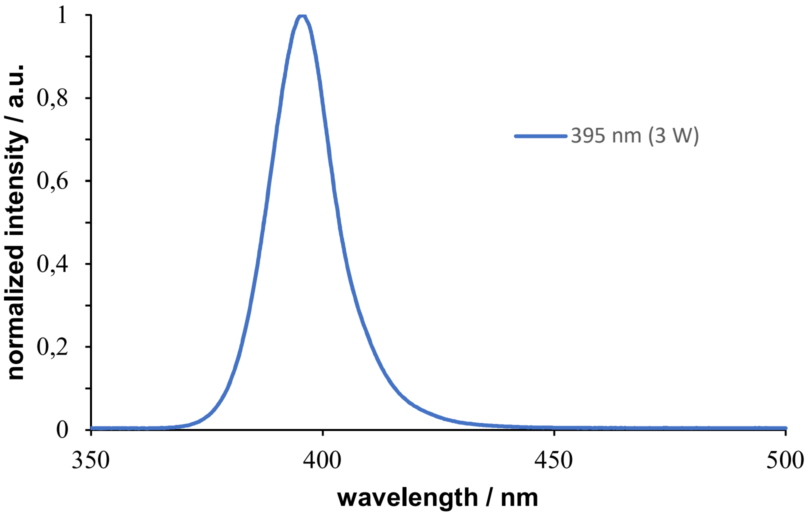

The photon flux of a single blue LED (3 W, λmax = 395 nm) was determined by ferrioxalate actinometry following a literature procedure.[21,22,23] For this purpose, the following two solutions were prepared:

Solution A:

Potassium ferrioxalate hydrate (737.0 mg, 1.5 mmol) was dissolved in aq. H_2SO_4 (50.0 mM, 10 mL) to afford a 150.0 mM ferrioxalate solution.

Solution B:

1,10-Phenanthroline monohydrate (20.0 mg, 0.1 mmol), NaOAc (2.7 g, 41.3 mmol) were dissolved in aq. H_2SO_4 (0.5 M, 20 mL).

Note: Solution A was prepared in the dark and both solutions were stored in the dark to avoid external irradiation prior to the actinometry. The following procedure was performed in a darkened lab.

First, the photon flux of the 395 nm LED was determined. For this, solution A (1.0 mL) was filled in a 10 mL Schlenk tube and irradiated for 60 s, at λ_{max} = 395 nm (3 W). After irradiation, solution B (175 µL) was added to the Schlenk tube and the mixture was stirred in the dark for 1 h to ensure coordination of $Fe^{(II)}$-ions by 1,10-phenanthroline. The solution was poured into a quartz cuvette and the absorption of the solution was measured at 510 nm. Sample preparation and measurement were repeated two more times. In a similar way a non-irradiated control sample was prepared, measured for absorbance at 510 nm, which was repeated twice. The average of the absorption of the both irradiated and radiated samples were calculated and were used to calculate the conversion factor n (2.07×10^{-7} mol) applying eq. 1.

$$n(\mathrm{Fe}^{2+}) = \frac{V\cdot\Delta A(510\ \mathrm{nm})}{l\cdot\varepsilon} \qquad (1)$$

V refers to the total volume (0.001175 L) of the solution (after addition of solution B), ΔA is the average difference in absorption of irradiated and non-irradiated samples between at 510 nm (ΔA = 1.95), l is the path length (1.0 cm) of the cuvette, and ε is the molar extinction coefficient of the ferrioxalate actinometer at 510 nm (11100 L mol^{-1} cm^{-1}). The photon flux (Φq) is calculated using eq. 2

$$\Phi_q = \frac{n(\mathrm{Fe}^{2+})}{\Phi_F \cdot t \cdot f} \qquad (2)$$

Φ_F refers to the quantum yield for the ferrioxalate actinometer (1.13, at λ_{ex} = 395 nm),[24] t is the irradiation time for solution A (60 s), and f is the fraction of light absorbed at λ_{ex} = 395 nm by the ferrioxalate actinometer. This value is calculated using eq. 3, where A(395 nm) is the absorption of the ferrioxalate solution at 395 nm. A measured absorbance value of >3 at 395 nm indicates the fraction of absorbed light (*f*) to be >0.999.

$$f = 1 - 10^{-A(395\ \mathrm{nm})} \qquad (3)$$

Thus, the average photon flux was calculated to be 3.05×10^{-9} einsteins s^{-1}.

3.9.2 Determination of the quantum yield

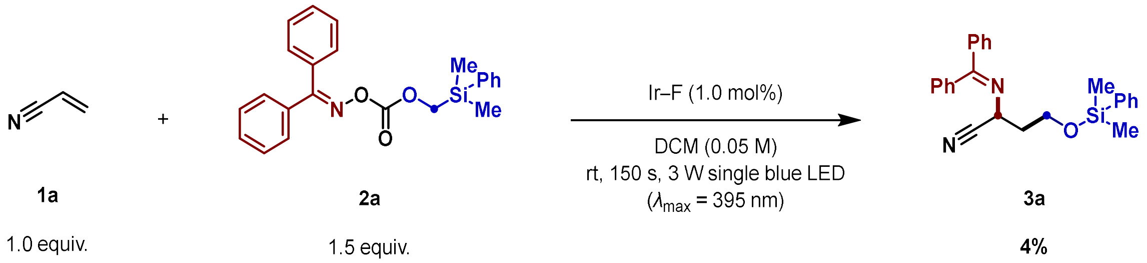

First, a stock solution was prepared: To an oven-dried 10 mL Schlenk tube was added acrylonitrile (26.5 mg, 0.50 mmol, 1.0 equiv.), diphenylmethanone O-(((dimethyl(phenyl)silyl) methoxy) carbonyl) oxime (291.8 mg, 0.75 mmol, 1.5 equiv.), Ir–F (5.0 mg, 0.005 mmol, 1 mol%) and DCM (10 mL). *Note: Concentration was increased to keep the volume constant referring to irradiation of Solution A.* Upon homogenization, 1 mL of 0.05 M stock solution was added to two 10 mL Schlenk tubes each equipped with Teflon coated stir bars under argon. Both reaction tubes were carefully degassed by two freeze/pump/thaw cycles and irradiated with the single blue LED (3 W, λ_{max} = 395 nm) for 150 s. Followed by an addition of 1 mL 0.1 M dibromomethane internal standard in $CDCl_3$ and yield determination by ^{1}H NMR, the average yield at 150 s was 4%. The quantum yield (Φ) of the reaction was determined using eq. 4, where the photon flux (Φ_q) is 3.05×10^{-9} einsteins s^{-1} (see above), *t* is the reaction time (2.5 min = 150 s) and f_R is the fraction of light absorbed by the reaction mixture (indicated in eq. 3; A_{Rct} (395 nm) = 1.60) determined using eq. 4.

$$\Phi = \frac{n(\mathrm{product})}{\Phi_q \cdot t \cdot f_R} \qquad (4)$$

Thus, the quantum yield (Φ) of the imino-fluorosulfonylation was determined to be: Φ = 4.48, which indicates a plausible **radical chain-induced reaction**. (*section 4)*

4 Computational studies

All geometry optimizations of intermediates and transition states were achieved using spin-unrestricted uB3LYP[25]-D3[26]/def2-SVP[27,28] method, in DCM solvent using the CPCM solvent model[29] with "opt=noeigen" and "guess=mix" keywords as implemented in Gaussian16.[30] Frequency calculations were also conducted at the same level of theory to obtain vibrational frequencies to determine the identity of stationary points as intermediates (no imaginary frequencies) or transition states (only one imaginary frequency), as well as obtaining the thermochemistry: enthalpy (ΔH) and free energy (ΔG) at the temperature of 298 K. Also, extensive conformational analysis was performed and only the lowest-energy species were shown and discussed. All structural figures were generated with CYLview.[31] Distances in structural figures are shown in Å and energies are in kcal.mol^{-1}. To compare energetics, we also carried out single-point energy calculations for the lowest energy structures using uB3LYP-d3/def2-TZVPP.[27]

4.1 Proposed mechanism

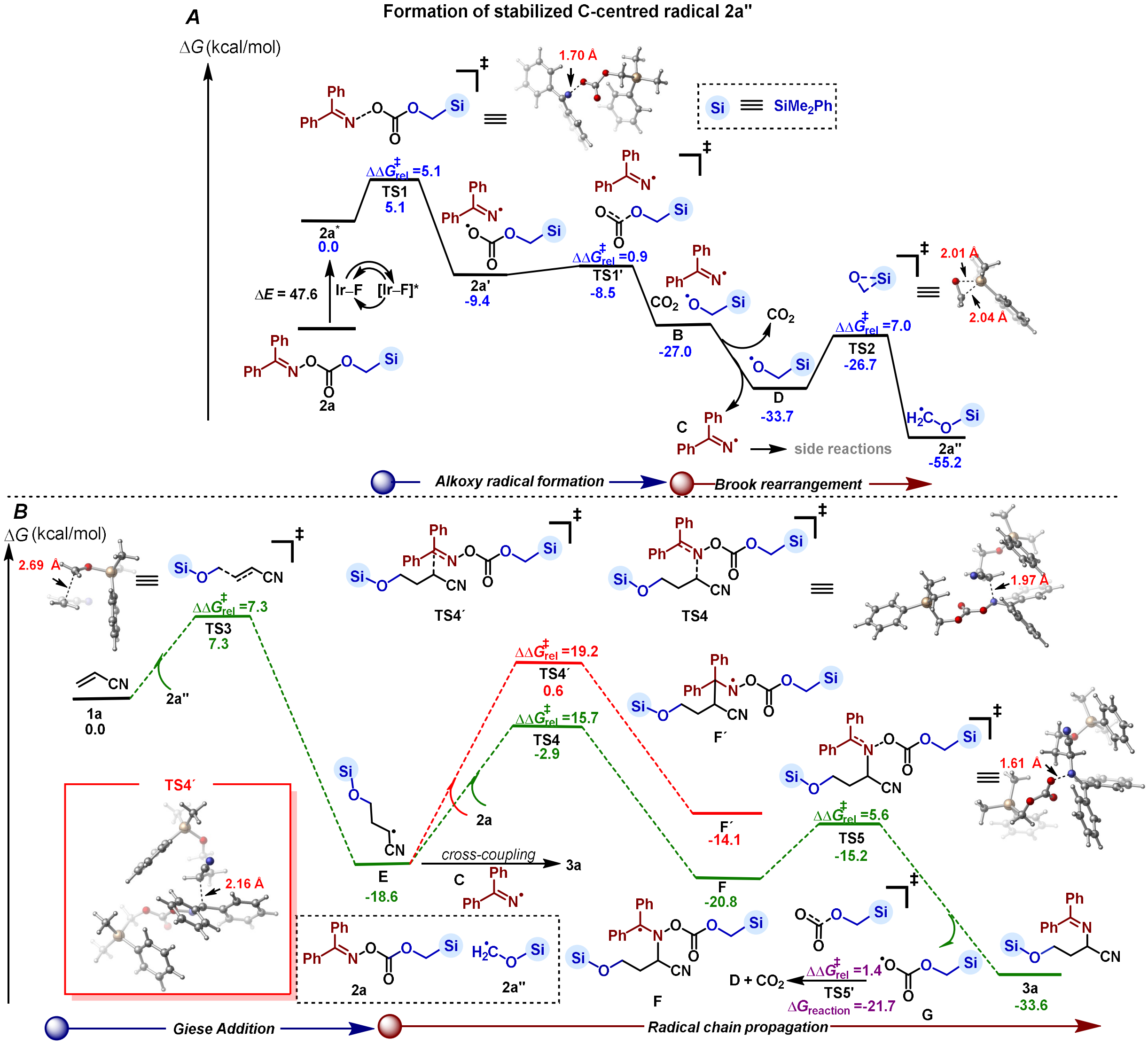

Figure F8: Proposed Mechanism supported by computational studies. Calculated free Gibbs energies [CPCM(DCM) uB3LYP-D3/def2-svp] are given in kcal.mol^{-1}.

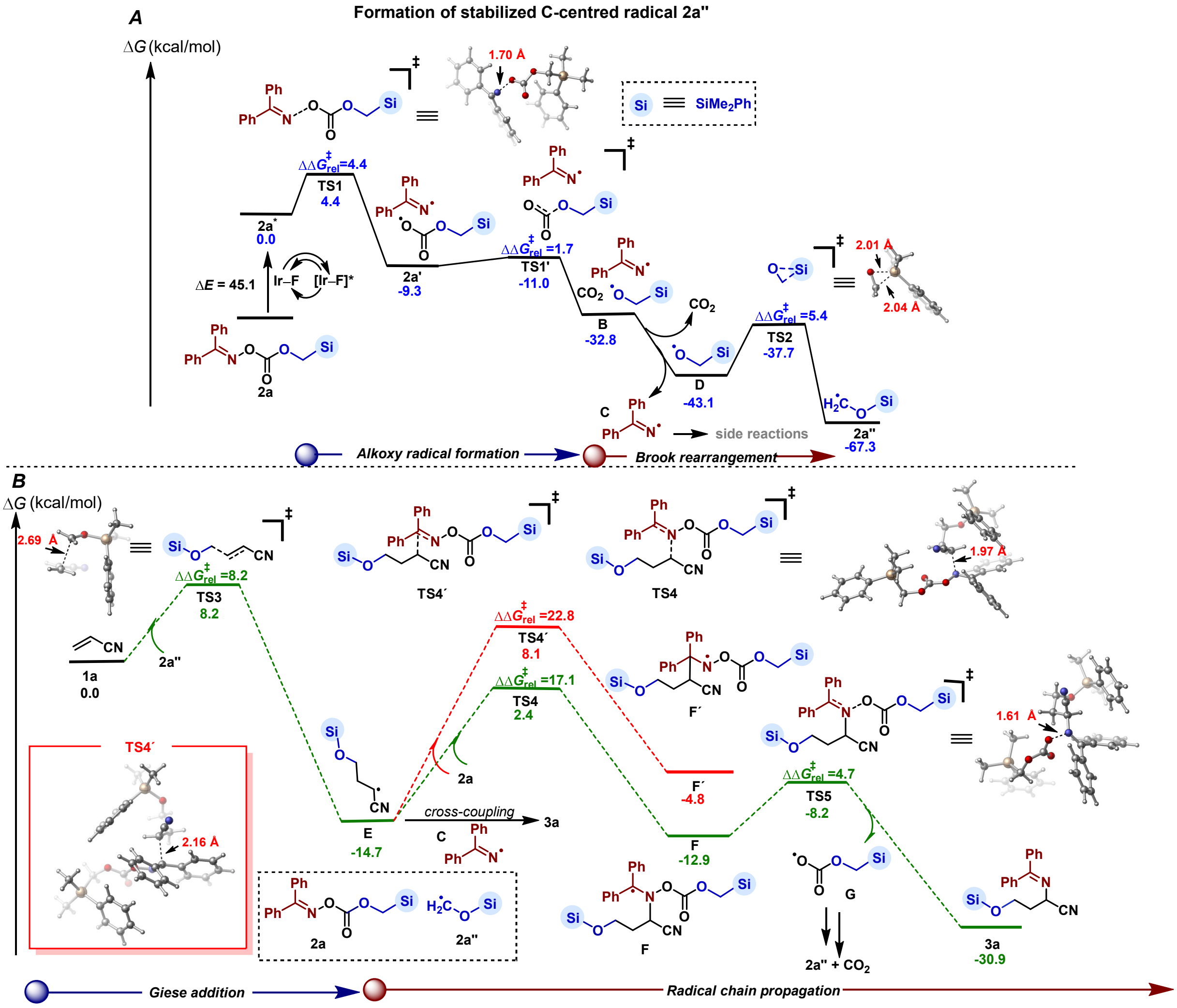

Figure F9. Proposed Mechanism supported by computational studies. Calculated free Gibbs energies [uB3LYP-D3/def2-tzvpp//CPCM(DCM) uB3LYP-D3/def2-svp] are given in kcal.mol^{-1}.

4.2 Addition of alternative radicals with alkene 1a

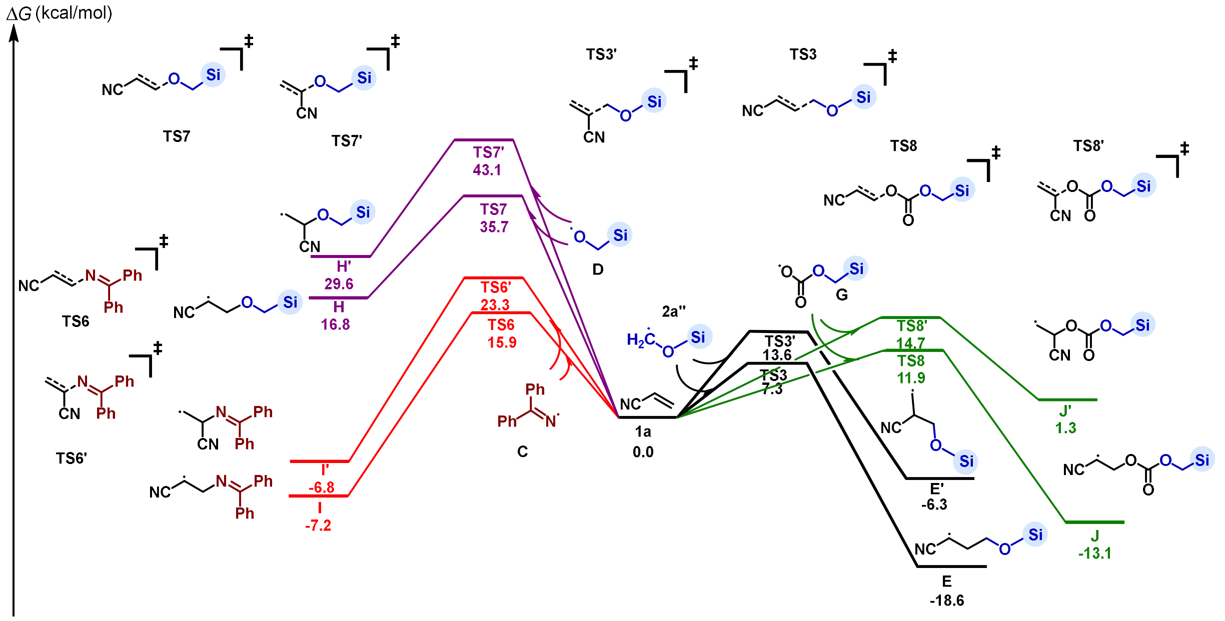

Figure F10: Addition of alternative radicals explored by computational studies. Calculated free Gibbs energies [CPCM(DCM) uB3LYP-D3/def2-svp] are given in kcal.mol^{-1}.

4.3 Cartesian coordinates

Table T6. Cartesian coordinates (xyz format) of all the structures involved in each reaction mechanism studied calculated at the CPCM(DCM) uB3LYP-d3/def2-SVP and single-point energies calculated at a larger basis set (def2-TZVPP in gas phase)

1a
E(scf) = -170.712307104 a.u.
ν_{min} = 234.0150 cm^{-1}

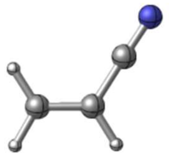

C	2.787661	-2.081227	-0.000005
C	4.129425	-2.069716	0.000003
H	2.201840	-1.158306	0.000010
H	2.243808	-3.028884	-0.000029
H	4.707368	-2.999781	-0.000012
C	4.897744	-0.859700	0.000034
N	5.536529	0.110852	0.000059

Zero-point correction= 0.050556 (Hartree/Particle)
Thermal correction to Energy= 0.054708
Thermal correction to Enthalpy= 0.055652
Thermal correction to Gibbs Free Energy= 0.024660
Sum of electronic and zero-point Energies= -170.661751
Sum of electronic and thermal Energies= -170.657599
Sum of electronic and thermal Enthalpies= -170.656655
Sum of electronic and thermal Free Energies= -170.687647

UB3LYP-D3/def2-TZVPP
E(scf) = -170.904235994 a.u.

2a
E(scf) = --1459.35249739 a.u.
ν_{min} = 7.6559 cm^{-1}

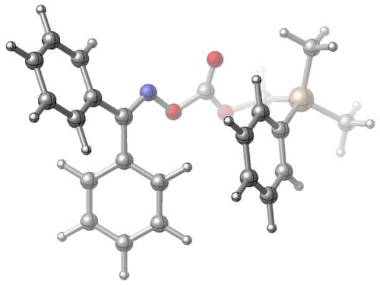

C	2.301867	-1.837570	0.701360	H	-5.777119	-2.572067	-0.921689
O	2.259466	-2.992027	0.362572	O	3.376964	-1.151733	1.061793
O	1.263107	-0.970949	0.765334	C	4.654231	-1.820161	0.908563
N	0.029105	-1.544901	0.421909	H	4.651986	-2.753597	1.496048
C	-0.847371	-0.645111	0.125483	H	5.373860	-1.129611	1.369720
C	-0.585872	0.819995	0.029102	Si	5.139767	-2.164541	-0.934324
C	-1.448614	1.729356	0.665125	C	6.925337	-1.600214	-1.147864
C	0.501953	1.311466	-0.714283	H	7.043276	-0.528458	-0.920522
C	-1.218322	3.104646	0.572754	H	7.586476	-2.163430	-0.468244
H	-2.301485	1.356227	1.236479	H	7.271365	-1.773488	-2.179872
C	0.716713	2.685923	-0.821939	C	4.967885	-3.998477	-1.312750
H	1.173450	0.617963	-1.217967	H	3.948476	-4.339261	-1.079422
C	-0.137729	3.586102	-0.174096	H	5.185455	-4.209882	-2.372282
H	-1.889272	3.801752	1.080542	H	5.673190	-4.579951	-0.696076
H	1.558598	3.051893	-1.414771	C	3.983166	-1.135229	-2.027814
H	0.036541	4.662071	-0.254077	C	4.257880	0.219553	-2.303261
C	-2.216749	-1.164504	-0.144452	C	2.819473	-1.694807	-2.593469
C	-3.072352	-0.491901	-1.036431	C	3.414575	0.981758	-3.119009
C	-2.665265	-2.352463	0.464909	H	5.147947	0.694931	-1.880630
C	-4.343442	-0.999496	-1.317169	C	1.973064	-0.938592	-3.411864
H	-2.737125	0.426659	-1.521377	H	2.559947	-2.735716	-2.385970
C	-3.936807	-2.853695	0.185594	C	2.271918	0.401395	-3.680663
H	-2.007965	-2.870815	1.164698	H	3.650182	2.030568	-3.318513
C	-4.780029	-2.179663	-0.706738	H	1.075431	-1.395434	-3.836760
H	-4.994590	-0.470043	-2.016951	H	1.609912	0.995661	-4.315631
H	-4.275780	-3.771851	0.671732				

Zero-point correction= 0.405264 (Hartree/Particle)
Thermal correction to Energy= 0.432069
Thermal correction to Enthalpy= 0.433013
Thermal correction to Gibbs Free Energy= 0.344590
Sum of electronic and zero-point Energies= -1458.947234
Sum of electronic and thermal Energies= -1458.920428
Sum of electronic and thermal Enthalpies= -1458.919484
Sum of electronic and thermal Free Energies= -1459.007908

UB3LYP-D3/def2-TZVPP
E(scf) = -1460.74147449 a.u.

2a*
E(scf) = -1459.27669873 a.u.
ν_{min}= 10.3665 cm^{-1}

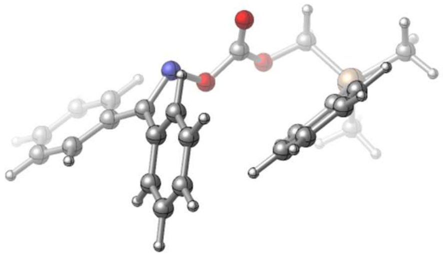

C	2.098687	-1.855376	-0.862317
O	2.307963	-2.091472	-2.017459
O	1.040700	-1.111004	-0.376786
N	0.045975	-0.876001	-1.314172
C	-0.790426	0.143988	-0.822019
C	-0.454911	1.483698	-1.297481
C	-0.694997	2.639133	-0.513179
C	0.195310	1.651745	-2.546440
C	-0.318984	3.901689	-0.969771
H	-1.143978	2.536747	0.475821
C	0.555776	2.918144	-2.999963
H	0.407852	0.773173	-3.157711
C	0.301006	4.051416	-2.217516
H	-0.501484	4.776955	-0.341222
H	1.047676	3.022541	-3.970000
H	0.591971	5.043030	-2.572096
C	-1.896895	-0.225294	0.034025
C	-3.041023	0.604658	0.186524
C	-1.894734	-1.475462	0.712936
C	-4.103675	0.216717	0.997890
H	-3.103478	1.544987	-0.362296
C	-2.963623	-1.853560	1.520642
H	-1.035333	-2.139809	0.615560
C	-4.072902	-1.010431	1.675514
H	-4.973070	0.872134	1.093912
H	-2.931972	-2.815307	2.038960
H	-4.909597	-1.310536	2.310808
O	2.804060	-2.201747	0.197925
C	4.137250	-2.748800	-0.015805
H	4.216250	-3.128150	-1.045493
H	4.228472	-3.599004	0.676678
Si	5.429182	-1.374006	0.358352
C	5.397870	-1.042713	2.211172
H	4.382775	-0.771477	2.543067
H	5.711101	-1.938959	2.771454
H	6.076587	-0.215340	2.474533
C	7.112978	-1.971329	-0.228588
H	7.102987	-2.211928	-1.303824
H	7.882562	-1.201405	-0.055810
H	7.411021	-2.879112	0.320935
C	4.856301	0.158037	-0.588345
C	3.714163	0.872298	-0.166633
C	5.517403	0.604255	-1.749243
C	3.242964	1.976745	-0.882349
H	3.166266	0.554142	0.724365
C	5.057397	1.716983	-2.463069
H	6.406804	0.078792	-2.108920
C	3.917129	2.402410	-2.032204
H	2.342105	2.500222	-0.553343
H	5.587801	2.045990	-3.360821
H	3.545136	3.262830	-2.593471

Zero-point correction= 0.402496 (Hartree/Particle)
Thermal correction to Energy= 0.429582
Thermal correction to Enthalpy= 0.430526

Thermal correction to Gibbs Free Energy= 0.340901
Sum of electronic and zero-point Energies= -1458.874203
Sum of electronic and thermal Energies= -1458.847117
Sum of electronic and thermal Enthalpies= -1458.846173
Sum of electronic and thermal Free Energies= -1458.935798

UB3LYP-D3/def2-TZVPP
E(scf) = -1460.665847 a.u

2a’
E(scf) = -1459.28792826 a.u.
ν_{min}= 13.2543 cm^{-1}

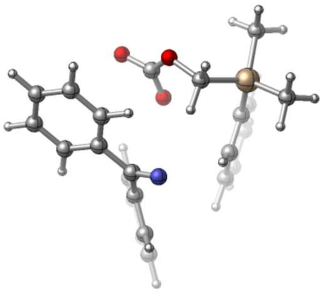

C	-0.233284	-0.736921	2.186528
O	0.171977	0.257596	1.523112
O	-1.124168	-0.519224	3.032323
N	-0.781698	-0.943388	-1.927915
C	-1.524608	-0.125043	-1.310126
C	-1.330408	1.340310	-1.583891
C	-1.239387	2.267417	-0.532421
C	-1.173168	1.778371	-2.909664
C	-1.002717	3.615250	-0.808946
H	-1.317535	1.922771	0.498620
C	-0.939283	3.128987	-3.180883
H	-1.242284	1.054963	-3.724470
C	-0.853969	4.049981	-2.130887
H	-0.922083	4.328797	0.014564
H	-0.824578	3.462735	-4.215030
H	-0.669068	5.106069	-2.342454
C	-2.562499	-0.600942	-0.338687
C	-3.633666	0.223206	0.046225
C	-2.484531	-1.906695	0.182453
C	-4.593587	-0.242832	0.949117
H	-3.723488	1.230089	-0.363444
C	-3.440024	-2.365677	1.087368
H	-1.661812	-2.557791	-0.116097
C	-4.496879	-1.533938	1.476396
H	-5.420207	0.409036	1.241795
H	-3.356074	-3.375022	1.496975
H	-5.243042	-1.892431	2.189717
O	0.232155	-1.957785	2.027830
C	1.095051	-2.278173	0.894179
H	0.596984	-1.967857	-0.038799
H	1.137741	-3.376481	0.900916
Si	2.871064	-1.556219	0.980788
C	3.519709	-1.740170	2.734706
H	2.890813	-1.188160	3.451316
H	3.521682	-2.802223	3.028721
H	4.550892	-1.361073	2.820217
C	3.883342	-2.533014	-0.271597
H	3.459256	-2.433956	-1.283959
H	4.919745	-2.159243	-0.300261
H	3.909459	-3.603640	-0.010719
C	2.788677	0.260676	0.474432
C	3.340546	1.285349	1.271255
C	2.170228	0.629098	-0.743547
C	3.285759	2.620653	0.866752
H	3.817984	1.039783	2.223837
C	2.112031	1.962898	-1.150494
H	1.704281	-0.131590	-1.376386
C	2.669510	2.961735	-0.345903
H	3.719281	3.401256	1.497547
H	1.607491	2.227644	-2.082015
H	2.613786	4.008039	-0.657093

Zero-point correction= 0.400253 (Hartree/Particle)
Thermal correction to Energy= 0.428581
Thermal correction to Enthalpy= 0.429525
Thermal correction to Gibbs Free Energy= 0.337222
Sum of electronic and zero-point Energies= -1458.887675
Sum of electronic and thermal Energies= -1458.859347
Sum of electronic and thermal Enthalpies= -1458.858403
Sum of electronic and thermal Free Energies= -1458.950707

UB3LYP-D3/def2-TZVPP
E(scf) = -1460.67976803 a.u

B
E(scf) = -1459.30877150 a.u.
ν_{min}= 9.7699 cm^{-1}

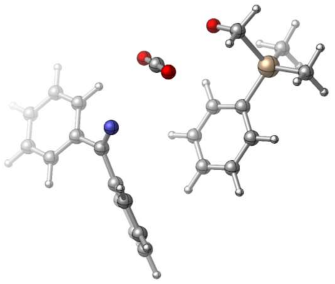

C	1.660009	-2.506535	-0.591745	C	-4.248526	-0.221394	1.343556
O	2.266643	-2.582596	-1.580738	H	-5.300378	1.274823	0.184014
O	1.020993	-2.432688	0.378276	H	-2.958698	-1.671768	2.302377
N	-0.356031	-0.674047	-1.825827	H	-5.071443	-0.368755	2.047451
C	-1.011525	0.336528	-1.440552	O	3.894623	-3.202176	0.861981
C	-0.655927	1.699444	-1.955645	C	4.932782	-3.198816	0.056604
C	-0.778675	2.830985	-1.131598	H	4.718578	-3.196156	-1.037246
C	-0.128128	1.842634	-3.250904	H	5.770226	-3.891103	0.315339
C	-0.373779	4.085284	-1.596384	Si	5.811510	-1.421171	0.328705
H	-1.177155	2.727373	-0.120675	C	6.167172	-1.307513	2.166370
C	0.269219	3.098016	-3.713567	H	5.230240	-1.370143	2.740625
H	-0.034751	0.962739	-3.890308	H	6.825704	-2.130588	2.487672
C	0.149695	4.221885	-2.887004	H	6.662009	-0.352057	2.405468
H	-0.464647	4.958451	-0.946009	C	7.362391	-1.446437	-0.729556
H	0.674564	3.200837	-4.722963	H	7.123384	-1.600028	-1.793518
H	0.463307	5.203955	-3.249166	H	7.899833	-0.488577	-0.631023
C	-2.132967	0.164696	-0.457558	H	8.038632	-2.251934	-0.402933
C	-3.323228	0.899336	-0.596158	C	4.585643	-0.115401	-0.236945
C	-2.010100	-0.767204	0.587995	C	3.334651	0.018498	0.400525
C	-4.376560	0.702839	0.300212	C	4.884372	0.757708	-1.300993
H	-3.425109	1.621519	-1.408902	C	2.416654	0.988453	-0.010657
C	-3.064095	-0.953327	1.485716	H	3.062901	-0.660201	1.213483
H	-1.082645	-1.331224	0.696344	C	3.970355	1.735868	-1.709127

H	5.842095	0.680549	-1.823311	H	4.220585	2.405720	-2.536075
C	2.735916	1.853926	-1.064015	H	2.020449	2.612541	-1.384623
H	1.445156	1.066763	0.483695				

Zero-point correction= 0.397695 (Hartree/Particle)
Thermal correction to Energy= 0.427731
Thermal correction to Enthalpy= 0.428675
Thermal correction to Gibbs Free Energy= 0.329986
Sum of electronic and zero-point Energies= -1458.911077
Sum of electronic and thermal Energies= -1458.881040
Sum of electronic and thermal Enthalpies= -1458.880096
Sum of electronic and thermal Free Energies= -1458.978785

UB3LYP-D3/def2-TZVPP
E(scf) = -1460.707122 a.u

CO_2
E(scf) = -188.445556181 a.u.
ν_{min}= 649.6564 cm^{-1}

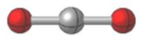

C	-3.222873	0.344942	0.000000
O	-2.059596	0.344942	0.000000
O	-4.386151	0.344942	0.000000

Zero-point correction= 0.011688 (Hartree/Particle)
Thermal correction to Energy= 0.014326
Thermal correction to Enthalpy= 0.015270
Thermal correction to Gibbs Free Energy= -0.009017
Sum of electronic and zero-point Energies= -188.433868
Sum of electronic and thermal Energies= -188.431230
Sum of electronic and thermal Enthalpies= -188.430286
Sum of electronic and thermal Free Energies= -188.454573

UB3LYP-D3/def2-TZVPP
E(scf) = -188.6700688 a.u

C
E(scf) = -555.724116622 a.u.
ν_{min}= 34.3486cm^{-1}

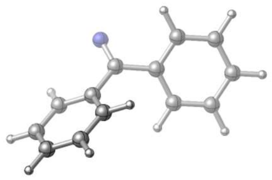

N	-0.478293	-1.035338	-0.398847	H	-0.268839	5.028466	0.331594
C	-1.423477	-0.222080	-0.185933	C	-2.830646	-0.730374	-0.060107
C	-1.131338	1.245285	-0.060469	C	-3.909202	0.006324	-0.579184
C	-1.836565	2.041592	0.858225	C	-3.072737	-1.978761	0.540067
C	-0.109264	1.824117	-0.833567	C	-5.209046	-0.499848	-0.497820
C	-1.522713	3.395825	0.999549	H	-3.729205	0.973905	-1.052014
H	-2.627695	1.598677	1.466561	C	-4.373991	-2.477867	0.624486
C	0.196890	3.179132	-0.693244	H	-2.234805	-2.546981	0.948965
H	0.433296	1.205959	-1.551704	C	-5.444578	-1.740437	0.105198
C	-0.508095	3.967603	0.223824	H	-6.041367	0.078057	-0.906885
H	-2.073871	4.006778	1.718596	H	-4.554222	-3.444541	1.101113
H	0.986184	3.623007	-1.304881	H	-6.462883	-2.131193	0.173347

Zero-point correction= 0.190819 (Hartree/Particle)
Thermal correction to Energy= 0.201659
Thermal correction to Enthalpy= 0.202603
Thermal correction to Gibbs Free Energy= 0.152172
Sum of electronic and zero-point Energies= -555.533298
Sum of electronic and thermal Energies= -555.522458
Sum of electronic and thermal Enthalpies= -555.521514
Sum of electronic and thermal Free Energies= -555.571945

UB3LYP-D3/def2-TZVPP
E(scf) = -556.3237612 a.u

D
E(scf) = -715.114503843 a.u.
ν_{min}= 16.3498 cm^{-1}

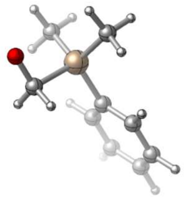

O	3.338917	-0.847903	-0.622079	C	6.216433	0.572531	-1.216724
C	4.278252	-1.765519	-0.608602	H	6.382138	0.165427	-2.226723
H	4.617920	-2.167739	-1.594340	H	5.338780	1.235964	-1.258110
H	4.184174	-2.586068	0.144260	H	7.098289	1.170674	-0.935228
Si	5.903250	-0.790309	0.031058	C	5.476351	-0.136878	1.735339

H	5.245190	-0.954320	2.436424	H	8.041836	-1.610353	-1.915054
H	6.311517	0.449683	2.151426	C	8.447892	-3.898359	1.201970
H	4.589315	0.511586	1.664883	H	6.790406	-2.823771	2.051562
C	7.274251	-2.078663	0.065489	C	9.301800	-4.047614	0.103222
C	8.143224	-2.247152	-1.031721	H	9.813491	-3.331544	-1.874491
C	7.445201	-2.923997	1.181030	H	8.564694	-4.541937	2.077874
C	9.148142	-3.219769	-1.014335	H	10.086629	-4.808417	0.118256

Zero-point correction= 0.192805 (Hartree/Particle)
Thermal correction to Energy= 0.206001
Thermal correction to Enthalpy= 0.206946
Thermal correction to Gibbs Free Energy= 0.151585
Sum of electronic and zero-point Energies= -714.921699
Sum of electronic and thermal Energies= -714.908502
Sum of electronic and thermal Enthalpies= -714.907558
Sum of electronic and thermal Free Energies= -714.962919

UB3LYP-D3/def2-TZVPP
E(scf) = -715.6945441 a.u

2a''
E(scf) = -715.148224856 a.u.
ν_{min}= 18.2724

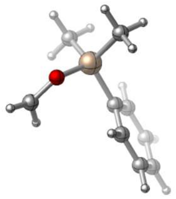

O	4.614279	-1.239212	0.957207	C	8.405466	-3.849548	0.985941
C	3.937628	-2.354696	0.589950	H	6.818458	-2.759976	1.952712
H	3.097611	-2.628164	1.235049	C	9.022938	-3.098552	-1.231497
Si	5.952349	-0.578422	0.127953	H	7.922484	-1.422193	-2.012359
C	6.530599	0.819133	1.228222	C	9.201856	-3.977778	-0.158219
H	5.733337	1.567848	1.364438	H	8.538507	-4.537535	1.825228
H	6.826235	0.439519	2.219245	H	9.639592	-3.197236	-2.128912
H	7.402873	1.321533	0.779224	H	9.959137	-4.764327	-0.213838
H	4.453900	-3.119307	-0.003754	C	5.403746	0.016263	-1.562873
C	7.243680	-1.943559	-0.014439	H	6.237007	0.491171	-2.106342
C	7.437879	-2.842526	1.054243	H	5.027485	-0.820405	-2.172894
C	8.050530	-2.094534	-1.158793	H	4.597861	0.760878	-1.460332

Zero-point correction= 0.192286 (Hartree/Particle)

Thermal correction to Energy= 0.205830
Thermal correction to Enthalpy= 0.206774
Thermal correction to Gibbs Free Energy= 0.150996
Sum of electronic and zero-point Energies= -714.955939
Sum of electronic and thermal Energies= -714.942395
Sum of electronic and thermal Enthalpies= -714.941451
Sum of electronic and thermal Free Energies= -714.997229

UB3LYP-D3/def2-TZVPP
E(scf) = -715.7324565 a.u

E
E(scf) = -885.916951564 a.u.
ν_{min}= 19.1052

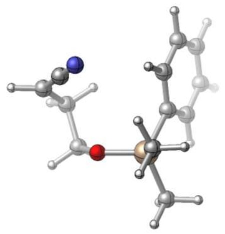

C	2.399141	-1.481169	-0.033000	C	4.589568	-4.698949	2.901698
C	1.545293	-2.015613	-1.138822	H	5.199229	-5.400474	2.309433
H	1.805921	-1.394871	0.891755	H	5.163374	-3.764420	3.013407
H	1.668167	-1.653271	-2.164116	H	4.445227	-5.130926	3.905598
C	0.638182	-3.053784	-0.934409	C	1.874294	-3.227333	3.066064
N	-0.135610	-3.914388	-0.738321	C	0.498723	-3.085502	2.785245
H	2.766637	-0.478257	-0.300270	C	2.418666	-2.434925	4.095693
C	3.621307	-2.383535	0.265943	C	-0.297959	-2.185768	3.499873
H	4.179493	-1.933577	1.109431	H	0.040989	-3.674961	1.986013
H	4.295086	-2.386316	-0.607934	C	1.626325	-1.531670	4.814054
O	3.233481	-3.710363	0.527442	H	3.479889	-2.517597	4.347226
Si	2.944633	-4.403320	2.044866	C	0.265690	-1.405026	4.516275
C	2.040601	-5.994213	1.656772	H	-1.361233	-2.091625	3.263182
H	1.762733	-6.526852	2.580569	H	2.072005	-0.925985	5.607761
H	1.124445	-5.784837	1.081840	H	-0.355427	-0.700238	5.075530
H	2.678274	-6.659597	1.052258				

Zero-point correction= 0.249475 (Hartree/Particle)
Thermal correction to Energy= 0.266705
Thermal correction to Enthalpy= 0.267650
Thermal correction to Gibbs Free Energy= 0.202509
Sum of electronic and zero-point Energies= -885.667476
Sum of electronic and thermal Energies= -885.650246
Sum of electronic and thermal Enthalpies= -885.649302
Sum of electronic and thermal Free Energies= -885.714442

UB3LYP-D3/def2-TZVPP
E(scf) = -886.6869093 a.u

E'
E(scf) = -885.894144989 a.u.
ν_{min}= 12.7595

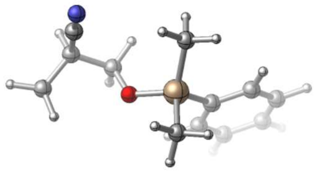

C	0.747836	0.799252	-0.408028	H	-2.178197	-0.597121	5.191130
C	2.024656	1.587756	-0.484411	C	-2.175293	3.255288	4.663923
H	0.126607	0.993357	-1.300246	H	-0.462399	2.941835	3.390795
H	2.572755	1.784818	0.438809	C	-3.138763	2.658965	5.485335
H	2.485450	1.798413	-1.451051	H	-3.883661	0.801058	6.309475
C	1.053383	-0.646405	-0.409131	H	-2.179445	4.338216	4.512691
N	1.315301	-1.775939	-0.380293	H	-3.896553	3.274025	5.978028
C	-0.088410	1.151939	0.844847	H	0.232327	-2.187059	2.264630
H	-0.356319	2.221357	0.778540	H	-0.773867	-2.238265	3.740908
H	-1.029828	0.572933	0.824509	H	-1.429677	-1.538389	2.240733
O	0.666409	0.911998	2.004872	H	1.449174	-0.627736	5.371963
Si	0.156137	0.039053	3.363337	H	2.471527	-0.687729	3.907456
C	-1.180775	1.066713	4.209380	H	2.090870	0.883517	4.672866
C	-2.162182	0.486389	5.036891	C	1.688428	-0.112312	4.427509
C	-1.207971	2.464791	4.034326	C	-0.518793	-1.636625	2.852858
C	-3.130860	1.271799	5.671338				

Zero-point correction= 0.247568 (Hartree/Particle)
Thermal correction to Energy= 0.265386
Thermal correction to Enthalpy= 0.266330
Thermal correction to Gibbs Free Energy= 0.199303
Sum of electronic and zero-point Energies= -885.646577
Sum of electronic and thermal Energies= -885.628759
Sum of electronic and thermal Enthalpies= -885.627815
Sum of electronic and thermal Free Energies= -885.694842

H
E(scf) = -885.856238616 a.u.
ν_{min}= 18.1022

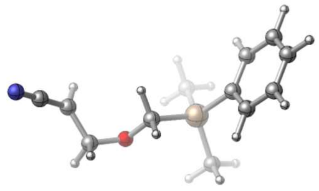

C	1.105166	-1.962339	-0.712271	H	5.595211	-5.065943	2.797859
C	1.968090	-2.257774	-1.909281	C	5.750286	-1.691571	4.719939
H	0.050775	-2.173467	-0.977262	H	3.715998	-1.387080	4.094103
H	2.309155	-3.287580	-2.057571	C	6.866365	-2.536215	4.692583
C	2.286057	-1.305639	-2.870910	H	7.668320	-4.413570	3.974602
N	2.548716	-0.487881	-3.671080	H	5.794925	-0.742758	5.261512
H	1.161958	-0.886121	-0.451347	H	7.784557	-2.249543	5.212175
Si	2.892388	-3.740907	2.442552	C	3.013151	-5.457001	1.678714
C	1.406198	-3.607988	3.593159	H	2.102518	-5.661036	1.092948
H	1.379127	-2.625386	4.091800	H	3.876496	-5.531379	0.997514
H	0.476545	-3.719861	3.012205	H	3.112059	-6.240192	2.447681
H	1.428907	-4.387193	4.372180	C	2.649804	-2.476789	1.034064
C	4.488069	-3.279278	3.347643	H	2.636086	-1.439634	1.428264
C	5.623523	-4.112848	3.333839	H	3.500089	-2.531362	0.323762
C	4.576996	-2.062576	4.055636	O	1.414668	-2.782982	0.384373
C	6.800559	-3.748749	3.998514				

Zero-point correction= 0.247500 (Hartree/Particle)
Thermal correction to Energy= 0.265165
Thermal correction to Enthalpy= 0.266109
Thermal correction to Gibbs Free Energy= 0.198168
Sum of electronic and zero-point Energies= -885.608738
Sum of electronic and thermal Energies= -885.591073
Sum of electronic and thermal Enthalpies= -885.590129
Sum of electronic and thermal Free Energies= -885.658070

H'
E(scf) = -885.833815812 a.u.
ν_{min}= 16.2470

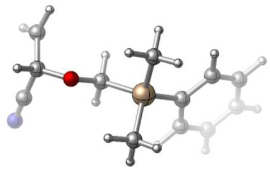

C	0.165578	0.353182	0.048746	C	0.943154	1.526152	-0.462311

H	-0.802924	0.293836	-0.488767	C	2.871545	1.936995	7.004720
H	1.780426	1.413127	-1.152500	H	1.156925	2.782645	6.017390
H	0.578602	2.521295	-0.198246	C	3.466569	-0.322750	6.375725
C	0.863658	-0.933674	-0.222827	H	2.219353	-1.262476	4.897570
N	1.404750	-1.943553	-0.393254	C	3.708769	0.824755	7.140151
Si	0.104132	0.690337	4.092798	H	3.054512	2.835615	7.600177
H	-0.327737	-1.752561	4.397282	H	4.116767	-1.195877	6.477641
H	-1.505881	-0.783227	5.323581	H	4.547893	0.850691	7.840466
H	-1.677208	-0.952662	3.550604	C	0.876893	0.549709	2.353914
H	-1.707532	2.221013	3.377872	H	1.532044	-0.342457	2.295864
H	-1.433397	2.373448	5.135784	H	1.512916	1.429129	2.136406
H	-0.315963	3.154758	3.982179	O	-0.201156	0.449733	1.413601
C	1.535239	0.757556	5.327402	C	-0.951752	-0.844391	4.372954
C	1.798201	1.901039	6.106725	C	-0.933009	2.259081	4.160567
C	2.391137	-0.352782	5.482376				

Zero-point correction= 0.245662 (Hartree/Particle)
Thermal correction to Energy= 0.263875
Thermal correction to Enthalpy= 0.264819
Thermal correction to Gibbs Free Energy= 0.196083
Sum of electronic and zero-point Energies= -885.588154
Sum of electronic and thermal Energies= -885.569941
Sum of electronic and thermal Enthalpies= -885.568996
Sum of electronic and thermal Free Energies= -885.637733

I
E(scf) = -726.469511785 a.u.
ν_{min}= 21.4841

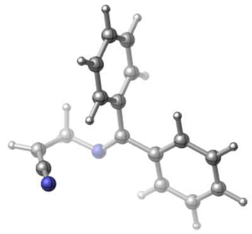

C	0.259834	0.683433	-0.036211	C	3.520403	2.255527	0.867311
C	1.621219	0.750597	-0.662065	C	5.431994	0.681652	2.423517
H	-0.618044	0.885609	-0.656861	C	4.640489	-1.390789	1.449730
H	1.558039	0.257216	-1.649552	C	2.630085	2.933422	1.714819
H	1.840389	1.820491	-0.874036	C	4.418725	2.991746	0.078969
C	0.066283	0.456498	1.321692	C	6.396989	-0.028140	3.145205
N	-0.078003	0.270925	2.472036	H	5.368912	1.765623	2.532178
N	2.639070	0.085840	0.120044	C	5.603511	-2.096381	2.168095
C	3.500248	0.753177	0.796420	H	3.946225	-1.907336	0.785264
C	4.541494	0.010098	1.566468	C	2.636344	4.330881	1.768198

H	1.935567	2.363638	2.336484	C	3.527542	5.060790	0.974454
C	4.417811	4.388904	0.129187	H	1.941475	4.850414	2.432699
H	5.118547	2.466952	-0.576311	H	5.116371	4.954544	-0.492459
C	6.486702	-1.417121	3.019494	H	7.241883	-1.971800	3.582214
H	7.079856	0.508268	3.808612	H	3.529805	6.152854	1.015369
H	5.669977	-3.182563	2.065846				

Zero-point correction= 0.244681 (Hartree/Particle)
Thermal correction to Energy= 0.260183
Thermal correction to Enthalpy= 0.261127
Thermal correction to Gibbs Free Energy= 0.198436
Sum of electronic and zero-point Energies= -726.224831
Sum of electronic and thermal Energies= -726.209329
Sum of electronic and thermal Enthalpies= -726.208384
Sum of electronic and thermal Free Energies= -726.271076

I'
E(scf) = -726.445805168 a.u.
ν_{min}= 22.6839

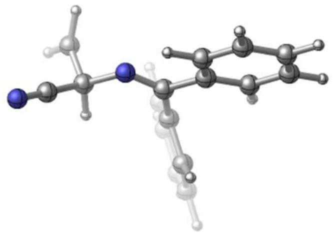

C	-0.042130	1.037812	0.626799	H	-1.709298	-1.977951	3.020405
C	1.079177	1.194894	-0.364062	C	0.156123	-3.199729	-0.167239
H	0.893371	1.021719	-1.425491	H	1.523368	-1.561449	0.192094
H	2.015071	1.647305	-0.029721	C	1.606203	0.277403	5.983227
C	-0.819998	2.302321	0.696158	H	0.885746	1.453841	4.313788
N	-1.421856	3.292493	0.726313	C	1.750099	-2.109681	5.603329
H	-0.747093	0.269222	0.252953	H	1.144076	-2.798153	3.652098
N	0.469917	0.758286	1.962191	C	-0.993696	-3.867096	0.267425
C	0.452445	-0.435119	2.435890	H	-2.566967	-3.940016	1.753358
C	-0.042288	-1.636193	1.683220	H	0.688573	-3.545616	-1.056550
C	0.951721	-0.645365	3.827755	C	1.918416	-1.008099	6.447112
C	-1.190179	-2.317987	2.121035	H	1.732804	1.141529	6.640294
C	0.627944	-2.085985	0.534040	H	1.995825	-3.114197	5.956335
C	1.130388	0.457840	4.685829	H	-1.364218	-4.735144	-0.283405
C	1.267068	-1.931290	4.303405	H	2.290988	-1.148320	7.464990
C	-1.667709	-3.422280	1.410757				

Zero-point correction= 0.243165 (Hartree/Particle)
Thermal correction to Energy= 0.259120
Thermal correction to Enthalpy= 0.260064
Thermal correction to Gibbs Free Energy= 0.197090
Sum of electronic and zero-point Energies= -726.202640
Sum of electronic and thermal Energies= -726.186685
Sum of electronic and thermal Enthalpies= -726.185741
Sum of electronic and thermal Free Energies= -726.248715

J

E(scf) = -1074.30300658 a.u.

ν_{min}= 11.6716

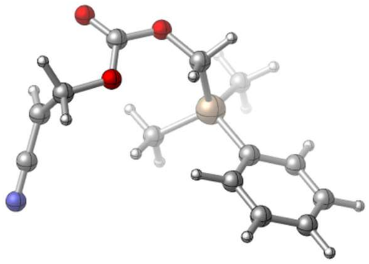

C	3.091549	0.092153	0.503819	C	5.087861	-4.573993	1.603368
O	3.529653	-0.422434	1.670023	C	4.797990	-5.833117	-0.436081
O	2.354483	1.044445	0.411804	C	5.970800	-5.545719	2.083819
O	3.557700	-0.557511	-0.551059	H	4.867378	-3.705255	2.232797
C	4.368229	-1.760968	-0.419908	C	5.680394	-6.810105	0.039979
H	4.966252	-1.795269	-1.342792	H	4.348818	-5.964269	-1.424775
H	5.063974	-1.658519	0.425192	C	6.268659	-6.667622	1.300953
Si	3.303690	-3.355307	-0.275417	H	6.427314	-5.429528	3.070456
C	1.918573	-3.079456	0.971894	H	5.909137	-7.684634	-0.575068
H	2.317651	-2.878653	1.977025	H	6.958300	-7.429330	1.674249
H	1.280662	-2.229511	0.679034	C	3.053236	0.209977	2.873766
H	1.279989	-3.976324	1.024816	C	1.625493	-0.155822	3.147739
C	2.599570	-3.770605	-1.969125	H	3.725451	-0.135069	3.667844
H	3.400016	-3.935876	-2.708355	H	0.858536	0.175980	2.442522
H	1.974213	-4.677254	-1.929065	C	1.245552	-0.934126	4.234594
H	1.971160	-2.939319	-2.328412	N	0.933694	-1.589151	5.156940
C	4.483993	-4.697307	0.334641	H	3.138490	1.302917	2.763964

Zero-point correction= 0.264068 (Hartree/Particle)
Thermal correction to Energy= 0.283967
Thermal correction to Enthalpy= 0.284911

Thermal correction to Gibbs Free Energy= 0.211617
Sum of electronic and zero-point Energies= -1074.038938
Sum of electronic and thermal Energies= -1074.019039
Sum of electronic and thermal Enthalpies= -1074.018095
Sum of electronic and thermal Free Energies= -1074.091390

J'

E(scf) = -1074.27954948 a.u.

ν_{min}= 21.3259

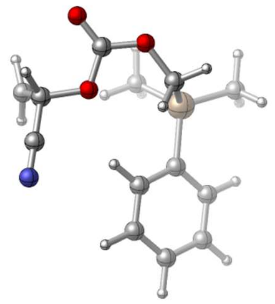

C	2.912316	-0.135844	0.857717
O	3.461980	-0.958778	1.784369
O	2.175700	0.782151	1.119183
O	3.273881	-0.449935	-0.372296
C	4.154309	-1.576085	-0.656412
H	4.562138	-1.350004	-1.652177
H	4.993121	-1.578027	0.054924
Si	3.247952	-3.285031	-0.688409
C	1.561615	-3.085607	0.129206
H	1.663928	-2.862047	1.201742
H	0.988819	-2.269733	-0.340850
H	0.981452	-4.016877	0.026868
C	3.060987	-3.833945	-2.478648
H	4.042316	-3.936426	-2.969919
H	2.540474	-4.803069	-2.546932
H	2.471507	-3.092582	-3.042643
C	4.318708	-4.504755	0.279891
C	4.725726	-4.188721	1.592561
C	4.722375	-5.744500	-0.251867
C	5.501597	-5.072809	2.347040
H	4.437054	-3.232482	2.033838
C	5.499311	-6.635489	0.498694
H	4.430584	-6.026220	-1.267806
C	5.890191	-6.301546	1.799833
H	5.803485	-4.798445	3.361488
H	5.801703	-7.592815	0.065534
H	6.497981	-6.996283	2.385644
C	3.096851	-0.690445	3.171184
C	1.770768	-1.274417	3.522009
H	1.694587	-2.297010	3.894597
H	3.082347	0.403417	3.307357
H	0.873846	-0.712234	3.259896
C	4.192404	-1.257972	3.975081
N	5.034145	-1.723787	4.618776

Zero-point correction= 0.262151 (Hartree/Particle)
Thermal correction to Energy= 0.282452
Thermal correction to Enthalpy= 0.283396
Thermal correction to Gibbs Free Energy= 0.211215
Sum of electronic and zero-point Energies= -1074.017398
Sum of electronic and thermal Energies= -1073.997097
Sum of electronic and thermal Enthalpies= -1073.996153

Sum of electronic and thermal Free Energies= -1074.068334

G
E(scf) = -903.544362897 a.u.
ν_{min}= 8.8280

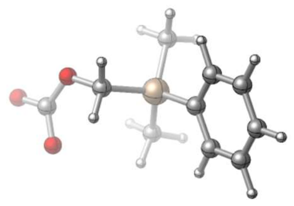

C	2.065081	-0.444896	0.356226
O	1.936289	-0.811038	1.546071
O	1.260163	0.457745	-0.002197
O	2.938881	-0.916038	-0.493831
C	3.870827	-1.957548	-0.044967
H	4.791419	-1.770054	-0.615348
H	4.098446	-1.812188	1.022912
Si	3.190111	-3.722089	-0.386665
C	1.578838	-3.934614	0.559937
H	1.702798	-3.692365	1.626765
H	0.801349	-3.268364	0.153008
H	1.214104	-4.971249	0.475072
C	2.925177	-3.889912	-2.241276
H	3.871007	-3.785600	-2.796670
H	2.488012	-4.871064	-2.487737
H	2.235518	-3.106461	-2.595514
C	4.522615	-4.903270	0.239978
C	4.404937	-5.544131	1.489551
C	5.685543	-5.148691	-0.518654
C	5.407819	-6.396691	1.964142
H	3.516664	-5.381224	2.106631
C	6.691758	-5.998793	-0.048651
H	5.813902	-4.672815	-1.495820
C	6.553977	-6.625287	1.195254
H	5.293744	-6.884751	2.935775
H	7.584546	-6.174965	-0.654601
H	7.338323	-7.291684	1.563802

Zero-point correction= 0.208299 (Hartree/Particle)
Thermal correction to Energy= 0.223976
Thermal correction to Enthalpy= 0.224920
Thermal correction to Gibbs Free Energy= 0.161528
Sum of electronic and zero-point Energies= -903.336064
Sum of electronic and thermal Energies= -903.320387
Sum of electronic and thermal Enthalpies= -903.319443
Sum of electronic and thermal Free Energies= -903.382835

UB3LYP-D3/def2-TZVPP
E(scf) = -904.3413817 a.u

F
E(scf) = -2345.30062858 a.u.
ν_{min}= 9.0507

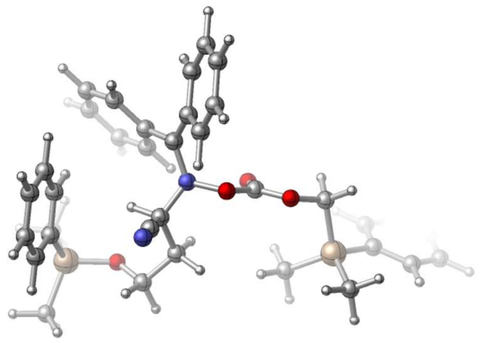

C	2.135363	0.935573	1.470999	H	0.005638	-4.084932	-3.328143
C	1.740341	1.582348	2.809789	O	-1.836474	-0.588726	2.171858
H	0.884536	2.243699	2.611319	C	-3.166444	-0.194816	2.617386
H	1.417262	0.814192	3.528547	H	-3.772898	-1.109644	2.550390
C	3.025289	-0.234678	1.667460	H	-3.580688	0.544995	1.915443
N	3.684257	-1.176555	1.814757	Si	-3.156680	0.482957	4.414612
H	2.706032	1.676366	0.893662	C	-4.899931	1.146672	4.708144
N	0.990244	0.601334	0.641065	C	-5.786577	0.547338	5.623651
O	0.104791	-0.266021	1.299314	C	-5.355992	2.271215	3.989270
C	1.033097	0.528191	-0.755729	C	-7.078887	1.049777	5.815652
C	-1.104467	0.326600	1.568392	H	-5.469117	-0.325516	6.201333
C	1.378341	1.783279	-1.408694	C	-6.646289	2.776611	4.175494
C	0.757051	-0.720285	-1.444518	H	-4.695294	2.766888	3.270433
O	-1.405122	1.456974	1.293085	C	-7.511188	2.165338	5.090997
C	2.099777	1.817537	-2.629114	H	-7.749776	0.569268	6.532881
C	1.059550	3.021349	-0.792241	H	-6.978425	3.649410	3.606951
C	1.026206	-1.976531	-0.839861	H	-8.520255	2.558998	5.239204
C	0.188804	-0.724175	-2.744796	C	-1.884694	1.866936	4.531445
C	2.483877	3.029289	-3.198813	H	-0.867880	1.479642	4.360323
H	2.386197	0.880327	-3.109206	H	-2.077279	2.649015	3.781662
C	1.447454	4.228402	-1.369531	H	-1.908883	2.323535	5.534207
H	0.479481	3.008910	0.131775	C	-2.738795	-0.922267	5.591975
C	0.761619	-3.165845	-1.514224	H	-3.473273	-1.740711	5.521120
H	1.463651	-2.013700	0.157818	H	-1.746868	-1.333934	5.343976
C	-0.072835	-1.918765	-3.412140	H	-2.711531	-0.571977	6.636610
H	-0.064170	0.224916	-3.220834	C	2.877179	2.415216	3.393283
C	2.165587	4.243325	-2.573311	H	3.778199	1.784181	3.525108
H	3.053717	3.028549	-4.131413	H	2.585298	2.770059	4.397392
H	1.182388	5.168620	-0.879585	O	3.128300	3.522182	2.549504
C	0.212979	-3.148738	-2.804219	Si	4.650694	4.020887	1.998115
H	0.991180	-4.119213	-1.031194	C	5.366682	2.624002	0.948487
H	-0.514073	-1.891791	-4.411853	C	4.865649	2.384358	-0.348820
H	2.476166	5.191209	-3.018877	C	6.356745	1.751124	1.440795

C	5.325558	1.310395	-1.116495	C	4.301038	5.537689	0.961819
H	4.094713	3.034624	-0.771397	H	3.629946	5.294888	0.123310
C	6.824741	0.677747	0.674404	H	3.819614	6.319176	1.571623
H	6.773003	1.904159	2.440681	H	5.236216	5.948146	0.547708
C	6.307413	0.454100	-0.605280	C	5.759875	4.387705	3.467569
H	4.912187	1.143321	-2.113868	H	5.864992	3.511359	4.127633
H	7.590197	0.010719	1.079740	H	6.768407	4.687260	3.138532
H	6.666362	-0.387752	-1.203178	H	5.333780	5.210778	4.064004

Zero-point correction= 0.659502 (Hartree/Particle)
Thermal correction to Energy= 0.704121
Thermal correction to Enthalpy= 0.705065
Thermal correction to Gibbs Free Energy= 0.574777
Sum of electronic and zero-point Energies= -2344.641127
Sum of electronic and thermal Energies= -2344.596508
Sum of electronic and thermal Enthalpies= -2344.595564
Sum of electronic and thermal Free Energies= -2344.725852

UB3LYP-D3/def2-TZVPP
E(scf) = -2347.453306 a.u

F'
E(scf) = -2345.29476233 a.u.
ν_{min}= 16.2624

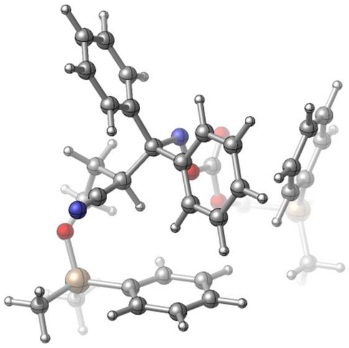

C	1.969186	1.726709	-1.615802	H	2.269002	1.452540	-0.595370
C	1.831959	3.268984	-1.669038	N	-0.386898	1.394232	-0.894168
H	1.996145	3.628003	-2.695920	O	0.223341	1.370736	0.326007
H	0.804035	3.550201	-1.397069	C	0.608683	0.981016	-1.901125
C	3.053736	1.253626	-2.478577	C	-0.566438	1.845780	1.356375
N	3.914991	0.870839	-3.153544	C	0.062654	1.413546	-3.270324

C	0.722888	-0.554241	-1.777846	Si	-1.176778	0.537802	4.526953
O	-1.694494	2.228935	1.248082	C	-1.821831	-0.510650	3.097377
C	0.630252	0.889147	-4.444006	C	-0.915260	-1.193204	2.261124
C	-0.957875	2.368818	-3.384621	C	-3.186966	-0.545072	2.753271
C	1.738193	-1.170753	-1.033347	C	-1.349542	-1.865685	1.115512
C	-0.265670	-1.363159	-2.364259	H	0.154991	-1.185727	2.484345
C	0.195510	1.317771	-5.699823	C	-3.629562	-1.226043	1.614336
H	1.413892	0.133351	-4.377236	H	-3.920995	-0.023738	3.374347
C	-1.397138	2.794160	-4.643691	C	-2.709196	-1.881417	0.788845
H	-1.417565	2.776521	-2.484328	H	-0.625545	-2.366574	0.472363
C	1.793552	-2.563290	-0.919342	H	-4.694051	-1.236908	1.364948
H	2.505532	-0.584869	-0.531492	H	-3.047572	-2.400917	-0.111539
C	-0.212640	-2.753957	-2.250301	C	-2.552297	1.085931	5.685419
H	-1.079771	-0.900274	-2.924160	H	-3.027838	0.216031	6.166945
C	-0.821353	2.273576	-5.805200	H	-2.150439	1.736335	6.479543
H	0.650915	0.898250	-6.600315	H	-3.330233	1.649541	5.145876
H	-2.195590	3.537354	-4.711989	C	0.187711	-0.368627	5.456078
C	0.825193	-3.361653	-1.534525	H	0.630052	0.283022	6.227373
H	2.601416	-3.017974	-0.341488	H	-0.217392	-1.264631	5.954003
H	-0.987476	-3.364468	-2.720191	H	0.997024	-0.695606	4.784513
H	-1.164378	2.606508	-6.787997	C	4.569264	1.317661	0.883155
H	0.871004	-4.449825	-1.447163	C	3.498105	1.311193	1.801136
C	2.788201	3.957143	-0.700933	C	5.133652	0.073048	0.534499
H	2.693123	5.052738	-0.809342	C	3.009997	0.118552	2.343468
H	2.481512	3.711962	0.333538	H	3.016080	2.245470	2.101349
O	4.122103	3.568671	-0.957452	C	4.662984	-1.122301	1.089808
Si	5.228587	2.936716	0.152232	H	5.957747	0.029155	-0.182479
C	5.478523	4.181546	1.536620	C	3.597952	-1.101743	1.997147
H	6.221851	3.815347	2.263723	H	2.162265	0.151478	3.028386
H	5.832377	5.143841	1.131932	H	5.122045	-2.072758	0.805382
H	4.538447	4.366156	2.082166	H	3.218387	-2.034351	2.422414
O	0.195182	1.808145	2.434231	C	6.772099	2.635642	-0.858746
C	-0.436831	2.123115	3.706885	H	7.568768	2.176758	-0.251696
H	0.361965	2.563828	4.320205	H	6.548399	1.968399	-1.706559
H	-1.213757	2.885768	3.545137	H	7.151380	3.587647	-1.263304

Zero-point correction= 0.659894 (Hartree/Particle)
Thermal correction to Energy= 0.704192
Thermal correction to Enthalpy= 0.705136
Thermal correction to Gibbs Free Energy= 0.579471
Sum of electronic and zero-point Energies= -2344.634869
Sum of electronic and thermal Energies= -2344.590570
Sum of electronic and thermal Enthalpies= -2344.589626
Sum of electronic and thermal Free Energies= -2344.715291

UB3LYP-D3/def2-TZVPP
E(scf) = -2347.445036 a.u

3a
E(scf) = -1441.75031526 a.u.
ν_{min}= 15.5042

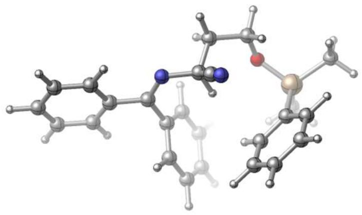

C	2.569019	0.335555	1.388190	C	1.127377	3.873748	-1.878012
C	2.083244	0.634272	2.824998	H	2.173853	2.984273	-3.551884
H	1.036617	0.967713	2.753358	H	0.072510	4.467568	-0.083425
H	2.096226	-0.290612	3.422475	C	-1.720967	-2.926926	-2.198526
C	3.889897	-0.324660	1.413789	H	-0.696275	-4.438960	-1.033685
N	4.926904	-0.840551	1.443845	H	-2.547354	-1.208015	-3.220507
H	2.717248	1.300900	0.875747	H	1.177570	4.888011	-2.281028
N	1.638841	-0.550110	0.707156	H	-2.395523	-3.639615	-2.679794
C	0.946154	-0.132019	-0.288739	C	2.895913	1.726632	3.509102
C	1.006352	1.268076	-0.835579	H	3.968298	1.449100	3.517224
C	0.021972	-1.093221	-0.961609	H	2.579201	1.803475	4.564886
C	1.619791	1.509007	-2.075870	O	2.678424	2.964191	2.856607
C	0.452897	2.342848	-0.123129	Si	3.883036	4.076759	2.441311
C	0.093553	-2.467743	-0.659322	C	4.974692	3.297509	1.112773
C	-0.937783	-0.654255	-1.891813	C	4.435822	3.034111	-0.163916
C	1.685031	2.806207	-2.591097	C	6.313189	2.929778	1.348524
H	2.049539	0.675308	-2.636324	C	5.197742	2.420225	-1.161652
C	0.510059	3.638994	-0.644920	H	3.401598	3.306496	-0.389308
H	-0.024808	2.165490	0.842639	C	7.084144	2.320185	0.351472
C	-0.767452	-3.375012	-1.273462	H	6.768312	3.117226	2.325357
H	0.838279	-2.803962	0.063547	C	6.526583	2.062150	-0.904992
C	-1.804455	-1.565483	-2.503294	H	4.750887	2.220922	-2.139032
H	-1.014756	0.406560	-2.135578				

Zero-point correction= 0.447545 (Hartree/Particle)
Thermal correction to Energy= 0.475745
Thermal correction to Enthalpy= 0.476690
Thermal correction to Gibbs Free Energy= 0.386855

Sum of electronic and zero-point Energies= -1441.302770
Sum of electronic and thermal Energies= -1441.274570
Sum of electronic and thermal Enthalpies= -1441.273626
Sum of electronic and thermal Free Energies= -1441.363460

UB3LYP-D3/def2-TZVPP
E(scf) = -1443.114162 a.u

TS1
E(scf) = -1459.26662245 a.u.
ν_{min}= -434.0604

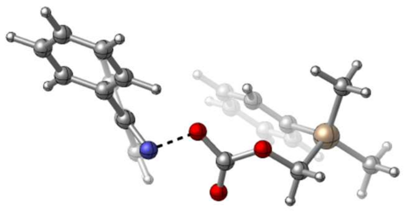

C	1.931409	-1.918267	-1.030193
O	2.122904	-1.835493	-2.221832
O	0.869754	-1.480111	-0.354545
N	-0.396448	-1.159845	-1.441772
C	-1.014864	-0.043220	-1.053292
C	-0.552978	1.217507	-1.646984
C	-0.859523	2.469890	-1.066797
C	0.280929	1.186226	-2.792376
C	-0.372906	3.648255	-1.632511
H	-1.452954	2.518674	-0.153548
C	0.757316	2.367477	-3.351155
H	0.560189	0.223230	-3.223597
C	0.426944	3.604004	-2.780478
H	-0.611624	4.607496	-1.167347
H	1.400511	2.326898	-4.233025
H	0.805267	4.529665	-3.220626
C	-2.103989	-0.171807	-0.068992
C	-3.273561	0.615032	-0.161689
C	-2.012301	-1.142584	0.954037
C	-4.307698	0.450594	0.759208
H	-3.380092	1.336947	-0.972919
C	-3.047957	-1.293905	1.875134
H	-1.113715	-1.756556	1.029174
C	-4.196896	-0.498328	1.783348
H	-5.209396	1.061561	0.673178
H	-2.958887	-2.037383	2.670799
H	-5.008329	-0.622265	2.504566
O	2.783769	-2.442880	-0.135650
C	4.143453	-2.720023	-0.561711
H	4.183817	-2.771342	-1.660247
H	4.395421	-3.715115	-0.161579
Si	5.308602	-1.370496	0.142504
C	5.194768	-1.438354	2.022070
H	4.151441	-1.300471	2.348513
H	5.544763	-2.412719	2.401132
H	5.806583	-0.646705	2.484234
C	7.055799	-1.710129	-0.470344
H	7.103147	-1.714285	-1.571334
H	7.757773	-0.945151	-0.100124
H	7.403899	-2.692069	-0.110456
C	4.680561	0.304210	-0.473997
C	3.423839	0.797010	-0.060859
C	5.432435	1.094740	-1.364797
C	2.935616	2.021287	-0.525719

H	2.801373	0.211320	0.620872	H	1.948595	2.371030	-0.213510
C	4.952896	2.326909	-1.825560	H	5.556305	2.923009	-2.515678
H	6.411362	0.749730	-1.709984	H	3.316599	3.745266	-1.775020
C	3.701999	2.791066	-1.407841				

Zero-point correction= 0.400772 (Hartree/Particle)
Thermal correction to Energy= 0.427848
Thermal correction to Enthalpy= 0.428793
Thermal correction to Gibbs Free Energy= 0.338895
Sum of electronic and zero-point Energies= -1458.865850
Sum of electronic and thermal Energies= -1458.838774
Sum of electronic and thermal Enthalpies= -1458.837830
Sum of electronic and thermal Free Energies= -1458.927728

UB3LYP-D3/def2-TZVPP
E(scf) = -1460.656895 a.u

TS1'
E(scf) = -1459.28327601 a.u.
ν_{min}= -336.9482

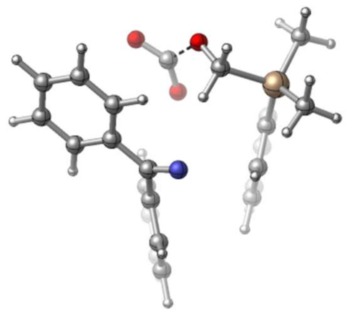

C	-0.343910	-0.592824	2.528370	H	-0.684962	3.322884	-4.232284
O	0.011016	0.417746	1.919711	H	-0.574230	5.030421	-2.415127
O	-1.340937	-0.807031	3.216553	C	-2.460905	-0.639991	-0.323690
N	-0.527188	-0.993378	-1.727516	C	-3.530787	0.209082	0.006152
C	-1.346204	-0.176732	-1.212303	C	-2.461631	-1.959273	0.170026
C	-1.165022	1.281613	-1.534337	C	-4.567420	-0.246295	0.826074
C	-1.094259	2.242559	-0.512338	H	-3.560820	1.227506	-0.382625
C	-1.007264	1.676075	-2.872914	C	-3.495552	-2.409217	0.988746
C	-0.883254	3.584752	-0.833792	H	-1.645157	-2.634348	-0.091798
H	-1.161814	1.925544	0.528798	C	-4.551490	-1.552723	1.322695
C	-0.799394	3.021659	-3.188112	H	-5.391772	0.425905	1.076091
H	-1.056512	0.924170	-3.663120	H	-3.475483	-3.431654	1.373268
C	-0.738896	3.978388	-2.169182	H	-5.359821	-1.904194	1.968589
H	-0.817778	4.326742	-0.034387	O	0.520614	-1.706048	2.479361

C	1.021507	-2.143569	1.248351	C	2.723195	0.311121	0.452126
H	0.414430	-1.810997	0.392567	C	3.020475	1.336147	1.377386
H	1.104770	-3.241992	1.287121	C	2.333593	0.684955	-0.853689
Si	2.853740	-1.496749	0.922486	C	2.957487	2.680486	1.006097
C	3.813919	-1.757349	2.511376	H	3.308067	1.081683	2.400742
H	3.323553	-1.241983	3.351962	C	2.263235	2.026643	-1.224764
H	3.866400	-2.830973	2.752875	H	2.059254	-0.079170	-1.585201
H	4.842143	-1.372526	2.417675	C	2.577603	3.027191	-0.295875
C	3.449664	-2.553614	-0.513557	H	3.195713	3.460487	1.733706
H	2.777774	-2.464655	-1.381674	H	1.937709	2.298883	-2.230911
H	4.458508	-2.236925	-0.824292	H	2.509658	4.078742	-0.585410
H	3.493830	-3.613953	-0.218008				

Zero-point correction= 0.397717 (Hartree/Particle)
Thermal correction to Energy= 0.426118
Thermal correction to Enthalpy= 0.427063
Thermal correction to Gibbs Free Energy= 0.333942
Sum of electronic and zero-point Energies= -1458.885559
Sum of electronic and thermal Energies= -1458.857158
Sum of electronic and thermal Enthalpies= -1458.856213
Sum of electronic and thermal Free Energies= -1458.949334

UB3LYP-D3/def2-TZVPP
E(scf) = -1460.67376137 a.u

TS2
E(scf) = -715.103526286 a.u.
ν_{min}= -291.1474

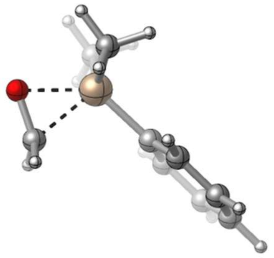

O	3.817047	-0.717381	0.320764	C	7.212462	-1.991967	-0.031307
C	4.250274	-1.980827	0.326086	C	7.895795	-2.415045	1.126332
H	4.300965	-2.532549	1.285510	C	7.566910	-2.584337	-1.260090
Si	5.815469	-0.691542	0.066800	C	8.905095	-3.381379	1.058269
C	6.127125	0.379253	1.573007	H	7.644946	-1.983192	2.099633
H	5.469409	1.260853	1.559347	C	8.575369	-3.550913	-1.333311
H	5.924346	-0.181658	2.499643	H	7.054864	-2.286681	-2.179926
H	7.176770	0.713690	1.599261	C	9.247598	-3.951856	-0.172966
H	4.076305	-2.617256	-0.564079	H	9.426791	-3.689598	1.968487

H	8.838559	-3.992112	-2.298574	H	6.742677	0.511227	-1.900208
H	10.035638	-4.707530	-0.228054	H	5.321680	-0.462665	-2.360789
C	5.735702	0.197187	-1.581312	H	5.088938	1.084052	-1.508861

Zero-point correction= 0.192224 (Hartree/Particle)
Thermal correction to Energy= 0.204887
Thermal correction to Enthalpy= 0.205831
Thermal correction to Gibbs Free Energy= 0.151769
Sum of electronic and zero-point Energies= -714.911303
Sum of electronic and thermal Energies= -714.898639
Sum of electronic and thermal Enthalpies= -714.897695
Sum of electronic and thermal Free Energies= -714.951757

UB3LYP-D3/def2-TZVPP
E(scf) = -715.6861944 a.u

TS3
E(scf) = -885.871422476 a.u.
ν_{min}= - 52.6002

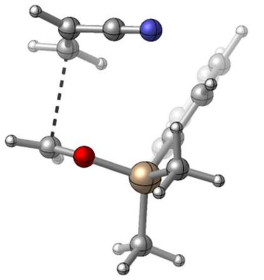

C	1.844983	-1.136636	0.002488	C	4.536453	-4.962211	3.087012
C	1.243182	-1.859910	-0.969624	H	5.176063	-5.654250	2.515725
H	1.664233	-1.335426	1.059804	H	5.138961	-4.075897	3.342392
H	1.401642	-1.630799	-2.028166	H	4.243943	-5.457877	4.027142
C	0.427605	-2.996082	-0.696105	C	1.937025	-3.275139	3.009664
N	-0.238620	-3.928823	-0.488873	C	0.536830	-3.268501	2.847910
H	2.447837	-0.264231	-0.255350	C	2.509644	-2.282425	3.831254
C	4.097427	-2.497175	0.564282	C	-0.259033	-2.302268	3.472168
H	4.355166	-1.943649	1.474282	H	0.056401	-4.012723	2.209192
H	4.565243	-2.240285	-0.388955	C	1.718999	-1.310488	4.453173
O	3.577823	-3.731424	0.650720	H	3.591425	-2.258546	3.992761
Si	3.020962	-4.505624	2.082252	C	0.331396	-1.318133	4.272431
C	2.071103	-5.987955	1.467262	H	-1.342695	-2.314426	3.328788
H	1.651478	-6.556851	2.312671	H	2.185663	-0.546875	5.081005
H	1.246138	-5.678889	0.806694	H	-0.288865	-0.559261	4.756641
H	2.738732	-6.656583	0.900415				

Zero-point correction= 0.244994 (Hartree/Particle)
Thermal correction to Energy= 0.262969
Thermal correction to Enthalpy= 0.263913
Thermal correction to Gibbs Free Energy= 0.198117
Sum of electronic and zero-point Energies= -885.626428
Sum of electronic and thermal Energies= -885.608453
Sum of electronic and thermal Enthalpies= -885.607509
Sum of electronic and thermal Free Energies= -885.673306

UB3LYP-D3/def2-TZVPP
E(scf) = -886.6460647 a.u

TS3'
E(scf) = -885.860562128 a.u.
ν_{min}= -389.6942

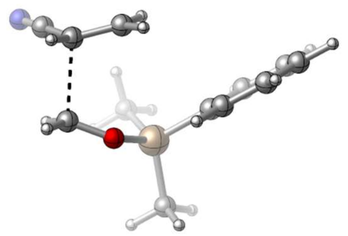

C	1.192855	0.716060	0.387198	H	-1.494763	2.408211	5.415098
C	2.186796	0.693780	1.352442	H	-2.636348	1.213358	4.728381
H	1.163795	1.539461	-0.332584	C	1.411497	1.043678	4.713983
H	2.369702	-0.188266	1.968646	C	2.096717	2.252996	4.482744
H	2.743513	1.601285	1.589706	C	2.037372	0.069304	5.515207
C	0.610827	-0.509348	-0.081863	C	3.361159	2.481831	5.033412
N	0.116495	-1.500561	-0.437458	H	1.639875	3.018951	3.850198
C	-0.696771	1.503205	1.367946	C	3.304019	0.293141	6.066936
H	-0.829986	2.376419	0.720170	H	1.538689	-0.884723	5.712797
H	-1.348315	0.639523	1.180241	C	3.967993	1.500784	5.826771
O	-0.385793	1.799056	2.632174	H	3.877892	3.425760	4.840003
Si	-0.300571	0.757589	3.994642	H	3.774687	-0.477816	6.682836
H	0.157558	-1.327273	2.687406	H	4.958315	1.676708	6.255063
H	-0.470335	-1.691145	4.316987	C	-0.569455	-1.014969	3.452135
H	-1.582860	-1.149182	3.040509	C	-1.635148	1.343193	5.170469
H	-1.593228	0.767910	6.110037				

Zero-point correction= 0.245225 (Hartree/Particle)
Thermal correction to Energy= 0.262887

Thermal correction to Enthalpy= 0.263831
Thermal correction to Gibbs Free Energy= 0.197417
Sum of electronic and zero-point Energies= -885.615337
Sum of electronic and thermal Energies= -885.597676
Sum of electronic and thermal Enthalpies= -885.596731
Sum of electronic and thermal Free Energies= -885.663145

TS4
E(scf) = -2345.27051754 a.u.
ν_{min}= -514.8189

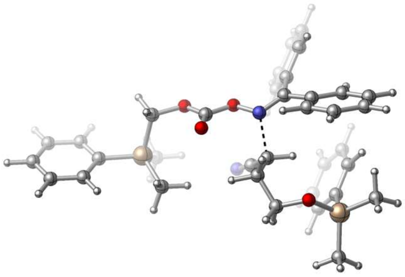

C	1.409876	2.076425	1.306607	H	4.417531	2.483754	-3.909182
C	1.089769	3.463422	1.815910	H	1.875051	5.591084	-2.318247
H	0.749080	4.077308	0.970797	C	0.894274	-2.920984	-2.512321
H	0.268652	3.429313	2.544578	H	1.188845	-3.393997	-0.419609
C	1.262361	0.989470	2.208770	H	0.589992	-2.137996	-4.508495
N	1.037817	0.080297	2.905454	H	3.872328	4.908351	-3.655992
H	2.322857	2.017611	0.710187	H	0.927401	-3.963881	-2.837158
N	0.168347	1.646219	-0.164431	O	-2.450253	0.209705	1.520471
O	-0.740437	0.695207	0.322458	C	-3.512990	0.486781	2.473805
C	0.810123	1.184189	-1.252298	H	-4.261767	-0.297635	2.291607
C	-1.692119	1.222961	1.141447	H	-3.972803	1.460342	2.241380
C	1.621665	2.191845	-1.948517	Si	-2.921269	0.406128	4.301359
C	0.812909	-0.230607	-1.671325	C	-4.509339	0.471295	5.325212
O	-1.797046	2.379898	1.452190	C	-5.374966	-0.640825	5.376849
C	2.748698	1.823152	-2.717887	C	-4.883136	1.627287	6.038477
C	1.321770	3.568798	-1.824726	C	-6.565981	-0.600823	6.108731
C	0.990411	-1.272258	-0.734920	H	-5.118848	-1.559796	4.839759
C	0.672403	-0.565577	-3.033986	C	-6.073184	1.673394	6.773766
C	3.544949	2.791749	-3.328075	H	-4.237274	2.510003	6.025165
H	3.015307	0.770281	-2.814305	C	-6.917496	0.55867	6.809812
C	2.125291	4.533226	-2.428982	H	-7.22087	-1.475991	6.134529
H	0.437759	3.866662	-1.260172	H	-6.341553	2.581351	7.320647
C	1.037292	-2.601125	-1.156337	H	-7.847261	0.592197	7.383948
H	1.105353	-1.038200	0.323409	C	-1.802214	1.866099	4.693586
C	0.708199	-1.897713	-3.448996	H	-0.853421	1.763456	4.146031
H	0.528228	0.229530	-3.768704	H	-2.264703	2.820761	4.399812
C	3.242730	4.151234	-3.182673	H	-1.573428	1.900927	5.771424

C	-2.00763	-1.221677	4.536539
H	-2.629736	-2.084506	4.249239
H	-1.097473	-1.220936	3.915621
H	-1.705031	-1.349705	5.588781
C	2.319650	4.140508	2.43311
H	2.723569	3.511071	3.249271
H	2.000103	5.093285	2.892757
O	3.290814	4.408195	1.441256
Si	4.898164	3.877931	1.429828
C	4.877963	1.994291	1.289883
C	4.796547	1.376709	0.025087
C	4.790902	1.168498	2.428534
C	4.607413	-0.003057	-0.099337
H	4.851929	1.982037	-0.882284
C	4.607375	-0.213618	2.310746
H	4.852083	1.605631	3.429508
C	4.506795	-0.801272	1.045603
H	4.526661	-0.456323	-1.090566
H	4.524497	-0.830999	3.208755
H	4.344493	-1.877974	0.950657
C	5.636144	4.667651	-0.096263
H	5.044933	4.417029	-0.991359
H	5.645181	5.764536	0.008321
H	6.671748	4.326732	-0.256117
C	5.749103	4.417969	3.014198
H	5.249648	4.010842	3.908302
H	6.798030	4.079014	3.030456
H	5.740089	5.517253	3.094008

Zero-point correction= 0.656855 (Hartree/Particle)
Thermal correction to Energy= 0.701398
Thermal correction to Enthalpy= 0.702342
Thermal correction to Gibbs Free Energy= 0.573143
Sum of electronic and zero-point Energies= -2344.613663
Sum of electronic and thermal Energies= -2344.569119
Sum of electronic and thermal Enthalpies= -2344.568175
Sum of electronic and thermal Free Energies= -2344.697375

UB3LYP-D3/def2-TZVPP
E(scf) = -2347.427201 a.u

TS4'
E(scf) = -2345.26548609 a.u.
ν_{min}= -546.8136

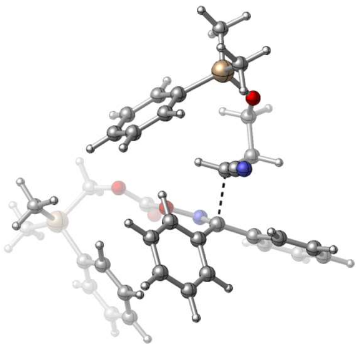

C	2.304507	1.782064	-1.421835
C	2.196549	3.217998	-1.877756
H	2.301705	3.278246	-2.971079
H	1.197966	3.601795	-1.616734

C	3.223540	0.941485	-2.104505
N	3.946508	0.236660	-2.688016
H	2.320086	1.633752	-0.337679
N	-0.229662	1.586902	-0.687643
O	0.085903	1.051684	0.537548
C	0.358964	0.887525	-1.683780
C	-0.595016	1.616069	1.586175
C	-0.011915	1.420245	-3.042390
C	0.618074	-0.598486	-1.547709
O	-1.567564	2.310733	1.497319
C	0.690322	0.999633	-4.186600
C	-1.033975	2.373497	-3.191323
C	1.435226	-1.145125	-0.542532
C	-0.059341	-1.481816	-2.408447
C	0.382872	1.522740	-5.443629
H	1.489179	0.263008	-4.092512
C	-1.343287	2.892477	-4.451778
H	-1.587813	2.703135	-2.312190
C	1.574438	-2.527100	-0.407413
H	1.970959	-0.497026	0.146131
C	0.082174	-2.866063	-2.273591
H	-0.716261	-1.088919	-3.184928
C	-0.635887	2.472429	-5.582186
H	0.943553	1.186516	-6.319201
H	-2.144990	3.628862	-4.548812
C	0.900200	-3.395733	-1.272002
H	2.217146	-2.925257	0.381632
H	-0.458112	-3.530372	-2.952283
H	-0.878045	2.879958	-6.566834
H	1.010780	-4.477435	-1.164320
C	3.245429	4.111475	-1.200000
H	3.142834	5.137846	-1.596008
H	3.023725	4.159665	-0.118092
O	4.552923	3.640750	-1.447436
Si	5.677417	3.134973	-0.287674
C	6.376407	4.640628	0.590569
H	7.130590	4.345432	1.338711
H	6.856217	5.316407	-0.136198
H	5.587972	5.208647	1.110865

O	0.039783	1.216970	2.674831
C	-0.616932	1.399231	3.959111
H	0.170083	1.727098	4.65466
H	-1.367177	2.199975	3.87853
Si	-1.391353	-0.286523	4.46846
C	-2.268918	-0.896521	2.911132
C	-1.532064	-1.505778	1.875305
C	-3.642219	-0.668141	2.693329
C	-2.136278	-1.862986	0.666015
H	-0.461289	-1.690604	1.992849
C	-4.254759	-1.029947	1.48886
H	-4.248556	-0.194615	3.471169
C	-3.500567	-1.624815	0.470744
H	-1.536261	-2.317468	-0.124309
H	-5.321823	-0.84225	1.341772
H	-3.975002	-1.900099	-0.475003
C	-2.582505	-0.000782	5.895126
H	-3.069811	-0.943953	6.191352
H	-2.044484	0.394599	6.772181
H	-3.368779	0.722265	5.625068
C	0.011604	-1.452009	4.935087
H	0.559238	-1.065895	5.810307
H	-0.371276	-2.455376	5.182075
H	0.728808	-1.556331	4.105302
C	4.797465	2.009011	0.95311
C	3.992432	2.545643	1.980605
C	4.852616	0.605994	0.834243
C	3.248167	1.721037	2.829855
H	3.933377	3.628996	2.122053
C	4.125408	-0.225376	1.693897
H	5.456807	0.1474	0.047601
C	3.311885	0.331193	2.686127
H	2.610288	2.160983	3.599728
H	4.183499	-1.310853	1.578717
H	2.722485	-0.313654	3.341759
C	6.970087	2.202778	-1.265778
H	7.738965	1.772179	-0.604288
H	6.50161	1.387366	-1.839636
H	7.468728	2.879781	-1.978043

Zero-point correction= 0.656767 (Hartree/Particle)
Thermal correction to Energy= 0.701329
Thermal correction to Enthalpy= 0.702273

Thermal correction to Gibbs Free Energy= 0.573594
Sum of electronic and zero-point Energies= -2344.608719
Sum of electronic and thermal Energies= -2344.564157
Sum of electronic and thermal Enthalpies= -2344.563213
Sum of electronic and thermal Free Energies= -2344.691892

UB3LYP-D3/def2-TZVPP
E(scf) = - 2347.418684 a.u

TS5
E(scf) = -2345.29250613 a.u.
ν_{min}= -573.5253

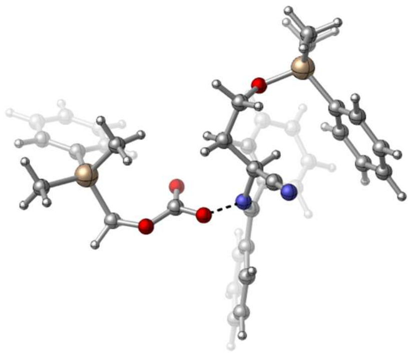

C	2.405417	0.522071	1.723339	C	0.641977	3.887769	-0.371109
C	1.850731	1.106234	3.038463	H	0.202284	2.363560	1.091947
H	0.873016	1.555052	2.826205	C	0.193288	-3.343652	-1.641458
H	1.691446	0.299309	3.767168	H	1.545545	-2.540225	-0.162173
C	3.591337	-0.323086	1.994129	C	-1.228154	-1.738089	-2.767129
N	4.516578	-0.979009	2.228042	H	-1.006652	0.317839	-2.158712
H	2.753047	1.359400	1.098640	C	1.078418	4.158869	-1.672799
N	1.481760	-0.249480	0.910957	H	1.791777	3.301002	-3.530138
O	0.585713	-1.148334	1.902897	H	0.343953	4.705279	0.289386
C	0.938068	0.100699	-0.269452	C	-0.806188	-3.060680	-2.582733
C	-0.670501	-0.715312	2.044902	H	0.529750	-4.373099	-1.494520
C	0.950552	1.498927	-0.739257	H	-2.014240	-1.509932	-3.491233
C	0.358541	-0.976157	-1.076120	H	1.134016	5.189588	-2.030880
O	-1.105587	0.361918	1.695247	H	-1.255336	-3.867581	-3.166948
C	1.380722	1.784630	-2.054572	O	-1.373065	-1.674360	2.640332
C	0.578477	2.573493	0.093743	C	-2.764844	-1.379762	2.933063
C	0.766815	-2.318357	-0.893195	H	-3.221957	-2.365962	3.098139
C	-0.656800	-0.706922	-2.022242	H	-3.233369	-0.931582	2.043069
C	1.444238	3.099379	-2.514063	Si	-3.027957	-0.256543	4.485090
H	1.681869	0.963571	-2.708414	C	-4.186649	1.151359	3.986279

C	-5.337732	1.474596	4.730241	O	2.800740	3.307422	2.782546
C	-3.895877	1.929777	2.845443	Si	4.192435	4.087537	2.213950
C	-6.168148	2.537659	4.355119	C	5.024242	2.946936	0.959889
H	-5.598019	0.891064	5.618204	C	4.409320	2.715446	-0.288866
C	-4.722714	2.992434	2.467681	C	6.216989	2.253978	1.242413
H	-3.012971	1.695088	2.243880	C	4.954781	1.818279	-1.211382
C	-5.861177	3.299386	3.222858	H	3.480159	3.229838	-0.546527
H	-7.057236	2.770735	4.947464	C	6.772670	1.360563	0.319389
H	-4.479864	3.583045	1.579934	H	6.723501	2.404109	2.200091
H	-6.508682	4.129516	2.927786	C	6.140200	1.138226	-0.907869
C	-1.358522	0.427363	5.032862	H	4.449849	1.649425	-2.165794
H	-0.614934	-0.379458	5.136627	H	7.696561	0.829348	0.562909
H	-0.980335	1.155382	4.300825	H	6.568576	0.434565	-1.626508
H	-1.457532	0.934233	6.006208	C	3.561877	5.643058	1.388418
C	-3.798941	-1.298584	5.851327	H	2.889976	5.399801	0.550986
H	-4.768434	-1.718247	5.536635	H	3.003922	6.264288	2.107562
H	-3.131024	-2.136986	6.109058	H	4.402176	6.237496	0.994483
H	-3.963255	-0.704253	6.764913	C	5.345161	4.451699	3.650384
C	2.767995	2.172922	3.626179	H	5.642869	3.533277	4.181714
H	3.778521	1.752555	3.794614	H	6.263128	4.951590	3.300236
H	2.377020	2.462302	4.617639	H	4.847182	5.116326	4.374948

Zero-point correction= 0.658013 (Hartree/Particle)
Thermal correction to Energy= 0.702242
Thermal correction to Enthalpy= 0.703186
Thermal correction to Gibbs Free Energy= 0.575514
Sum of electronic and zero-point Energies= -2344.634493
Sum of electronic and thermal Energies= -2344.590264
Sum of electronic and thermal Enthalpies= -2344.589320
Sum of electronic and thermal Free Energies= -2344.716992

UB3LYP-D3/def2-TZVPP
E(scf) = -2347.446579 a.u

TS5’
E(scf) = -903.544482194 a.u.
ν_{min}= -146.0455

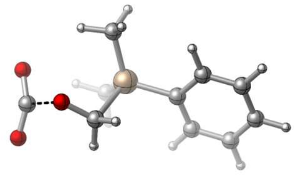

C	1.736871	-0.554523	-0.013734	O	1.039487	-0.951083	-0.939729

O	1.767796	-0.021800	1.081221	H	2.174140	-2.804668	-2.261637
O	3.335476	-0.957590	-0.476195	C	4.540579	-4.905926	0.274845
C	3.951330	-1.859486	0.282380	C	4.660062	-5.307031	1.629553
H	5.024524	-1.979380	0.051985	C	5.486540	-5.401532	-0.674768
H	3.700755	-1.839532	1.369065	C	5.684514	-6.183465	2.027464
Si	3.181812	-3.731914	-0.244727	H	3.948619	-4.936376	2.389759
C	1.607601	-3.911309	0.748559	C	6.501008	-6.278168	-0.288280
H	1.780173	-3.773937	1.828016	H	5.423214	-5.099377	-1.729267
H	0.859867	-3.178406	0.402015	C	6.601011	-6.669520	1.058866
H	1.201758	-4.925051	0.590405	H	5.752554	-6.484659	3.077888
C	2.908626	-3.605919	-2.090245	H	7.218748	-6.660814	-1.035953
H	3.841486	-3.360387	-2.623006	H	7.390958	-7.352712	1.363530
H	2.512334	-4.553470	-2.484816				

Zero-point correction= 0.206093 (Hartree/Particle)
Thermal correction to Energy= 0.221099
Thermal correction to Enthalpy= 0.222044
Thermal correction to Gibbs Free Energy= 0.163867
Sum of electronic and zero-point Energies= -903.338389
Sum of electronic and thermal Energies= -903.323383
Sum of electronic and thermal Enthalpies= -903.322439
Sum of electronic and thermal Free Energies= -903.380615

TS6
E(scf) = -726.430713898 a.u.
ν_{min}= -409.5782

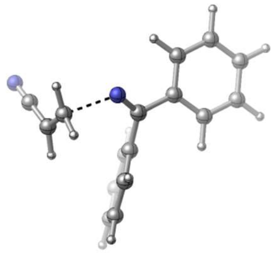

C	0.746098	1.540868	-0.346205	C	5.794203	0.631938	2.185327
C	1.523997	0.767995	-1.187378	C	4.558099	-1.438280	1.906190
H	0.972774	2.598015	-0.185160	C	3.079929	2.623257	2.154124
H	1.196245	-0.233149	-1.465785	C	3.799862	2.996686	-0.133353
H	2.194301	1.269118	-1.886823	C	6.724711	-0.035710	2.986489
C	-0.293267	0.993010	0.445900	H	5.917462	1.697258	1.981482
N	-1.151842	0.555214	1.102635	C	5.486573	-2.099525	2.710538
N	3.093050	-0.052202	-0.115271	H	3.704543	-1.975236	1.488276
C	3.716977	0.639354	0.747830	C	2.845322	3.992720	2.301629
C	4.704047	-0.064853	1.635560	H	2.890986	1.939602	2.984984
C	3.539191	2.118125	0.928302	C	3.579694	4.368153	0.023082

H	4.176160	2.604007	-1.080462	H	2.469711	4.379105	3.252208
C	6.573279	-1.400691	3.251641	H	3.786614	5.048689	-0.806350
H	7.570382	0.514307	3.406474	H	7.298204	-1.919519	3.883798
H	5.359731	-3.164061	2.922375	H	2.912207	5.938078	1.354922
C	3.092699	4.866895	1.236631				

Zero-point correction= 0.242680 (Hartree/Particle)
Thermal correction to Energy= 0.258239
Thermal correction to Enthalpy= 0.259183
Thermal correction to Gibbs Free Energy= 0.196392
Sum of electronic and zero-point Energies= -726.188033
Sum of electronic and thermal Energies= -726.172475
Sum of electronic and thermal Enthalpies= -726.171531
Sum of electronic and thermal Free Energies= -726.234322

TS6’
E(scf) = -726.418677792 a.u.
ν_{min}= -528.3991

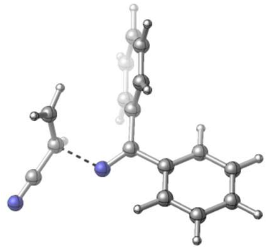

C	-0.869715	1.188665	0.590706	H	-2.143775	-1.452012	1.893594
C	-0.065730	0.690086	-0.433789	C	0.873742	-3.337977	0.321099
H	-0.174490	-0.345180	-0.757891	H	2.117119	-1.965268	1.440133
H	0.749895	1.284695	-0.850460	C	2.292454	0.184046	5.798475
C	-0.939925	2.621137	0.778795	H	1.696195	1.387856	4.100940
N	-0.998681	3.770956	0.917656	C	1.552885	-2.122133	5.769219
H	-1.808040	0.672071	0.810780	H	0.383389	-2.722266	4.056330
N	-0.092234	0.809442	2.376415	C	-0.433900	-3.730979	0.011735
C	0.256897	-0.387197	2.632123	H	-2.540315	-3.367319	0.355356
C	0.011376	-1.570722	1.742526	H	1.723365	-3.867487	-0.116876
C	0.962326	-0.636977	3.936160	C	2.241384	-1.080569	6.399193
C	-1.297422	-1.976812	1.444442	H	2.836093	0.998816	6.283153
C	1.097324	-2.269857	1.193313	H	1.509142	-3.110626	6.233017
C	1.656410	0.406539	4.577162	H	-0.607381	-4.568985	-0.667732
C	0.919190	-1.904807	4.542339	H	2.742122	-1.253848	7.355029
C	-1.518009	-3.055092	0.581988				

Zero-point correction= 0.241881 (Hartree/Particle)

Thermal correction to Energy= 0.257473
Thermal correction to Enthalpy= 0.258417
Thermal correction to Gibbs Free Energy= 0.196236
Sum of electronic and zero-point Energies= -726.176797
Sum of electronic and thermal Energies= -726.161205
Sum of electronic and thermal Enthalpies= -726.160261
Sum of electronic and thermal Free Energies= -726.222441

TS7
E(scf) = -885.822871064 a.u.
ν_{min}= -440.6990

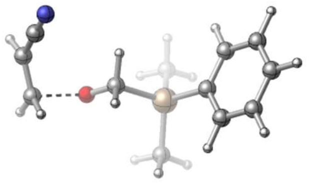

C	0.834876	-1.849181	-1.073531
C	1.665113	-2.101414	-2.146345
H	-0.160357	-2.293910	-1.059403
H	1.430344	-2.893550	-2.863109
C	2.926283	-1.471953	-2.280975
N	3.968295	-0.958051	-2.385591
H	0.983875	-0.962607	-0.454071
Si	2.863972	-3.792186	2.490803
C	1.359195	-3.450706	3.561648
H	1.302022	-2.389005	3.850372
H	0.446501	-3.695927	2.995912
H	1.376628	-4.058173	4.480931
C	4.466765	-3.250266	3.325295
C	5.696895	-3.858639	3.002702
C	4.477689	-2.200076	4.265785
C	6.890956	-3.437494	3.597033
H	5.730261	-4.677216	2.277901
C	5.668981	-1.774928	4.862776
H	3.543580	-1.703015	4.543263
C	6.878907	-2.393558	4.528807
H	7.833079	-3.925450	3.333344
H	5.653364	-0.959854	5.591244
H	7.811065	-2.062983	4.994520
C	2.933241	-5.578836	1.916183
H	2.018903	-5.814648	1.349277
H	3.795731	-5.756850	1.254151
H	3.005107	-6.270713	2.770918
C	2.695374	-2.681392	0.890447
H	2.709609	-1.623208	1.232805
H	3.615804	-2.881336	0.298562
O	1.537003	-3.085520	0.309746

Zero-point correction= 0.245144 (Hartree/Particle)
Thermal correction to Energy= 0.263035
Thermal correction to Enthalpy= 0.263979
Thermal correction to Gibbs Free Energy= 0.194816
Sum of electronic and zero-point Energies= -885.577727
Sum of electronic and thermal Energies= -885.559836
Sum of electronic and thermal Enthalpies= -885.558892
Sum of electronic and thermal Free Energies= -885.628055

TS7’
E(scf) = -885.812113971 a.u.
ν_{min}= -584.1642

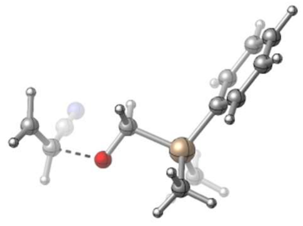

C	0.173952	0.334682	-0.289926	C	2.368787	-0.364485	5.505775
C	0.956326	1.379794	-0.780203	C	2.918033	1.921335	7.012439
H	-0.889975	0.316687	-0.540161	H	1.209710	2.800356	6.044091
H	2.046362	1.347436	-0.725565	C	3.455586	-0.353841	6.385582
H	0.476376	2.299890	-1.118356	H	2.170110	-1.271903	4.926656
C	0.762588	-0.972158	-0.115606	C	3.732175	0.791223	7.141892
N	1.251473	-2.010213	0.057735	H	3.128073	2.817953	7.601845
Si	0.091181	0.733481	4.140850	H	4.087728	-1.240530	6.483265
H	-0.218872	-1.745744	4.175213	H	4.580527	0.801641	7.831360
H	-1.414699	-0.943009	5.230214	C	0.874103	0.775259	2.370948
H	-1.620187	-0.917907	3.451825	H	1.542916	-0.110672	2.307302
H	-1.802639	2.218577	3.568174	H	1.494207	1.690913	2.282367
H	-1.493126	2.264976	5.327905	O	-0.201089	0.727237	1.509266
H	-0.443029	3.175732	4.206303	C	-0.882127	-0.870639	4.268228
C	1.535317	0.763654	5.358789	C	-1.013283	2.241694	4.336075
C	1.833000	1.905567	6.128507				

Zero-point correction= 0.244611 (Hartree/Particle)
Thermal correction to Energy= 0.262438
Thermal correction to Enthalpy= 0.263382
Thermal correction to Gibbs Free Energy= 0.195888
Sum of electronic and zero-point Energies= -885.567503
Sum of electronic and thermal Energies= -885.549676
Sum of electronic and thermal Enthalpies= -885.548732
Sum of electronic and thermal Free Energies= -885.616226

TS8
E(scf) = -1074.25646564 a.u.
ν_{min}= -191.0994

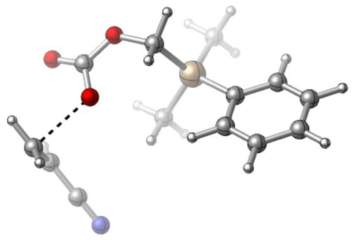

C	3.180877	0.043090	0.455924	C	5.080933	-4.604336	1.589509
O	3.635078	-0.399730	1.596836	C	4.782925	-5.879605	-0.438855
O	2.407214	0.983748	0.384658	C	5.950784	-5.581752	2.082139
O	3.610436	-0.592367	-0.628988	H	4.868239	-3.727629	2.210419
C	4.411470	-1.808939	-0.497485	C	5.652087	-6.862309	0.049587
H	4.996308	-1.860289	-1.427513	H	4.335292	-6.015198	-1.427637
H	5.127797	-1.704952	0.332457	C	6.237959	-6.714525	1.311065
Si	3.320152	-3.381805	-0.308643	H	6.405143	-5.461525	3.069273
C	1.957844	-3.039550	0.946256	H	5.872298	-7.745475	-0.556157
H	2.371992	-2.805973	1.937791	H	6.917273	-7.480698	1.694034
H	1.329167	-2.191066	0.629695	C	2.681962	0.775025	3.465126
H	1.307073	-3.924115	1.040837	C	1.543993	0.036573	3.426886
C	2.592654	-3.817272	-1.986917	H	3.511604	0.518208	4.126384
H	3.382367	-4.014041	-2.729918	H	0.727207	0.304828	2.749948
H	1.949854	-4.710156	-1.921721	C	1.371021	-1.150931	4.195145
H	1.977455	-2.981113	-2.357474	N	1.222569	-2.129061	4.806745
C	4.480080	-4.732699	0.319856	H	2.759355	1.679058	2.861919

Zero-point correction= 0.259832 (Hartree/Particle)
Thermal correction to Energy= 0.280569
Thermal correction to Enthalpy= 0.281513
Thermal correction to Gibbs Free Energy= 0.204923
Sum of electronic and zero-point Energies= -1073.996633
Sum of electronic and thermal Energies= -1073.975897
Sum of electronic and thermal Enthalpies= -1073.974952
Sum of electronic and thermal Free Energies= -1074.051542

TS8'
E(scf) = -1074.25456685 a.u.
ν_{min}= -171.6205

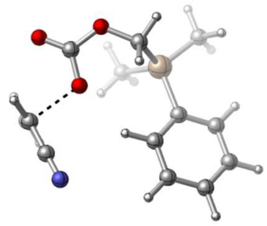

C	2.962370	-0.214762	0.844268
O	3.533514	-1.065634	1.675076
O	2.223759	0.695086	1.158043
O	3.280476	-0.447421	-0.436197
C	4.130082	-1.573768	-0.799662
H	4.439879	-1.351677	-1.831338
H	5.037926	-1.571955	-0.176226
Si	3.244786	-3.295167	-0.746859
C	1.559232	-3.090419	0.073890
H	1.670140	-2.877002	1.147280
H	0.994169	-2.263751	-0.386401
H	0.970724	-4.015493	-0.036552
C	3.049367	-3.906892	-2.517363
H	4.027005	-4.008039	-3.016445
H	2.544897	-4.886268	-2.550390
H	2.442102	-3.193125	-3.098007
C	4.326079	-4.486840	0.248502
C	4.716153	-4.157995	1.563899
C	4.753627	-5.723845	-0.271805
C	5.498154	-5.027397	2.329549
H	4.409924	-3.203940	1.997380
C	5.536367	-6.599731	0.490599
H	4.475814	-6.016355	-1.288576
C	5.910009	-6.253481	1.793492
H	5.785517	-4.742046	3.345340
H	5.856353	-7.554891	0.065196
H	6.521983	-6.936338	2.388973
C	3.037594	-0.627073	3.724135
C	1.773275	-1.156348	3.648380
H	1.563289	-2.185261	3.948353
H	3.203724	0.433649	3.521338
H	0.974380	-0.552128	3.215399
C	4.126245	-1.356231	4.308692
N	5.007245	-1.955809	4.767509

Zero-point correction= 0.260545 (Hartree/Particle)
Thermal correction to Energy= 0.280929
Thermal correction to Enthalpy= 0.281873
Thermal correction to Gibbs Free Energy= 0.207544
Sum of electronic and zero-point Energies= -1073.994022
Sum of electronic and thermal Energies= -1073.973638
Sum of electronic and thermal Enthalpies= -1073.972693
Sum of electronic and thermal Free Energies= -1074.047022

5 Spectra

Diphenylmethanone O-(((dimethyl(phenyl)silyl)methoxy)carbonyl) oxime (2a)

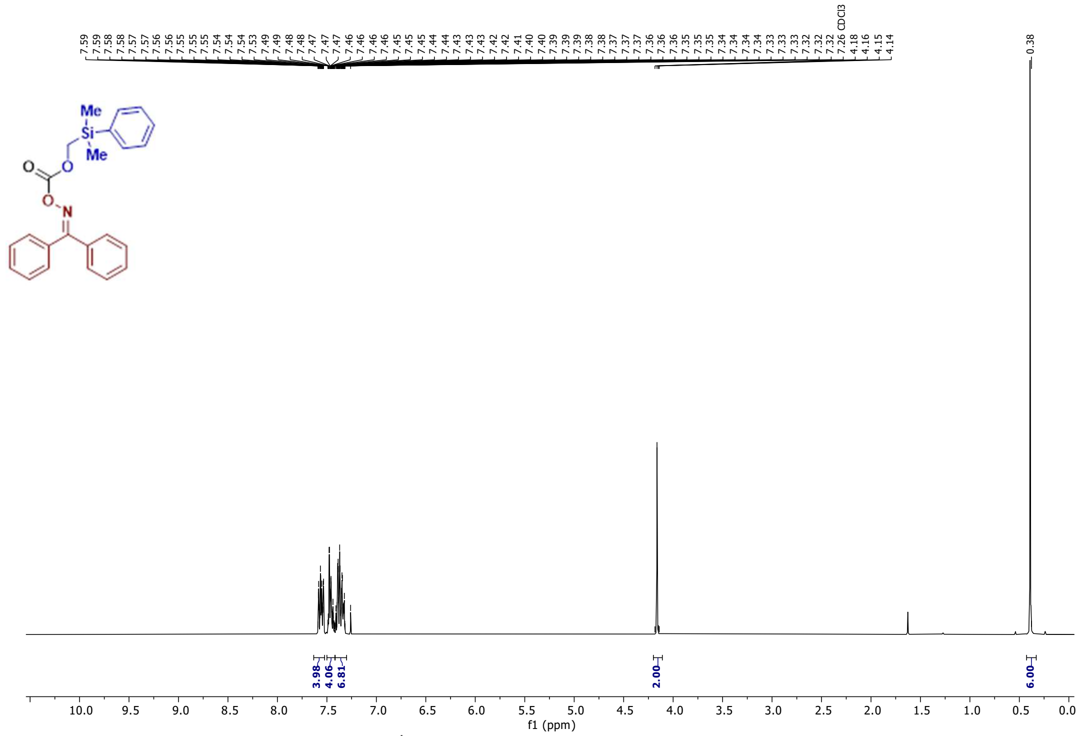

^{1}H NMR (400 MHz, $CDCl_3$)

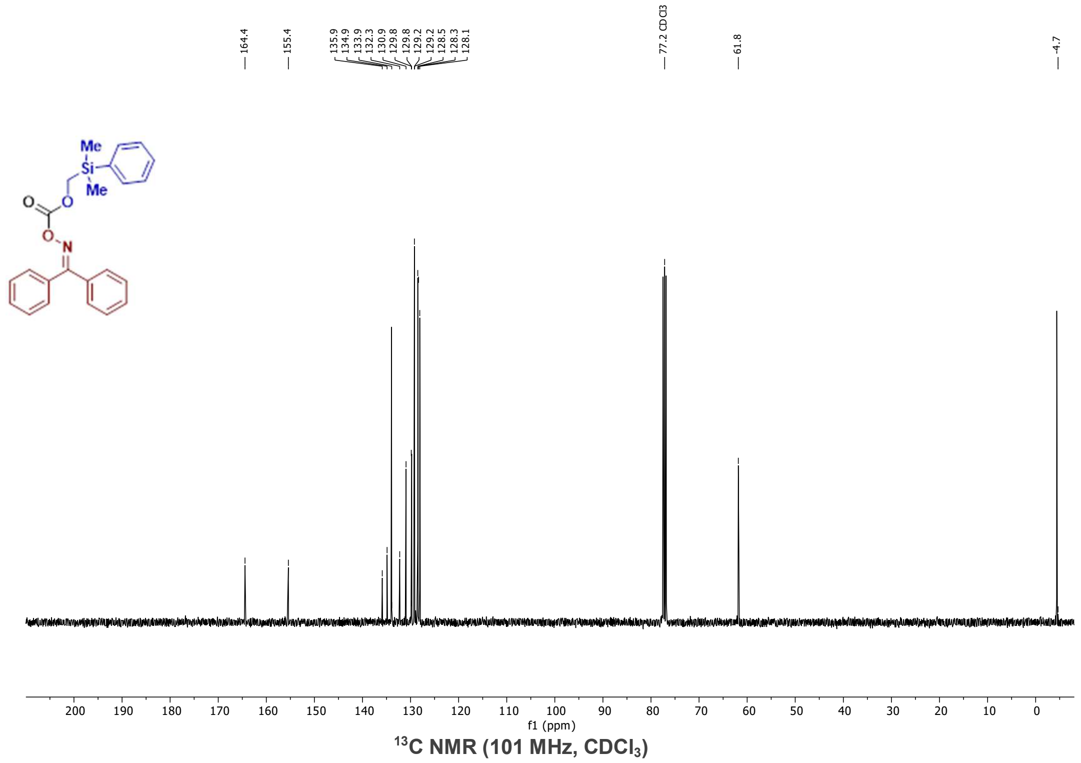

^{13}C NMR (101 MHz, $CDCl_3$)

Diphenylmethanone O-(((trimethylsilyl)methoxy)carbonyl) oxime (2b)

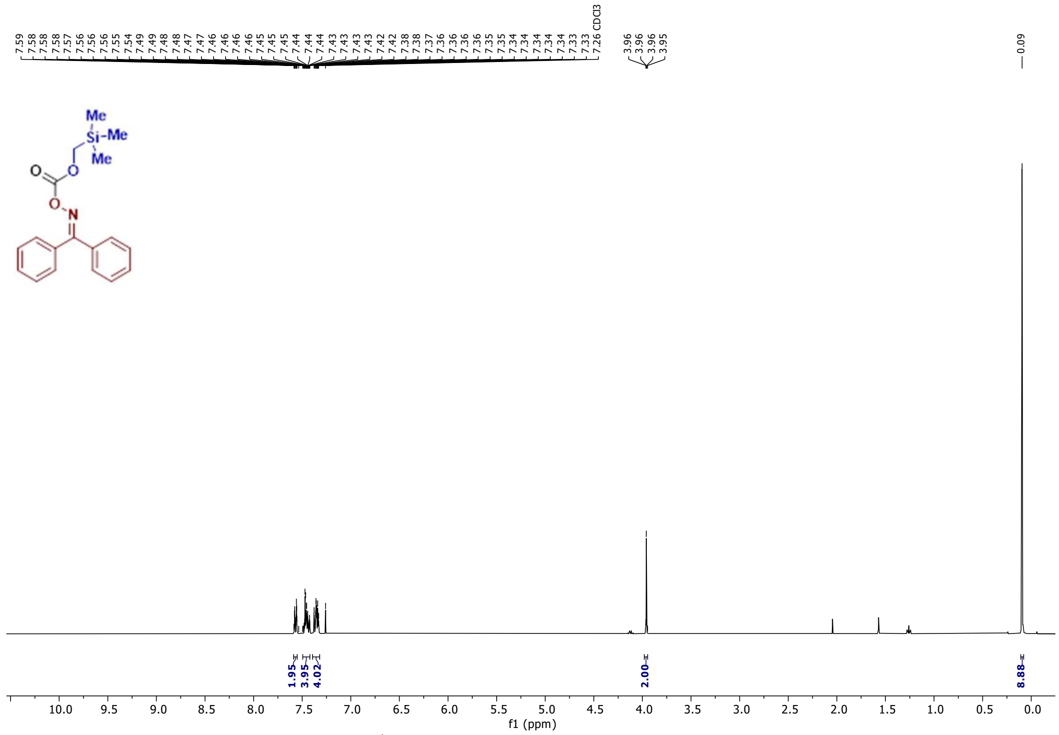

^{1}H NMR (400 MHz, $CDCl_3$)

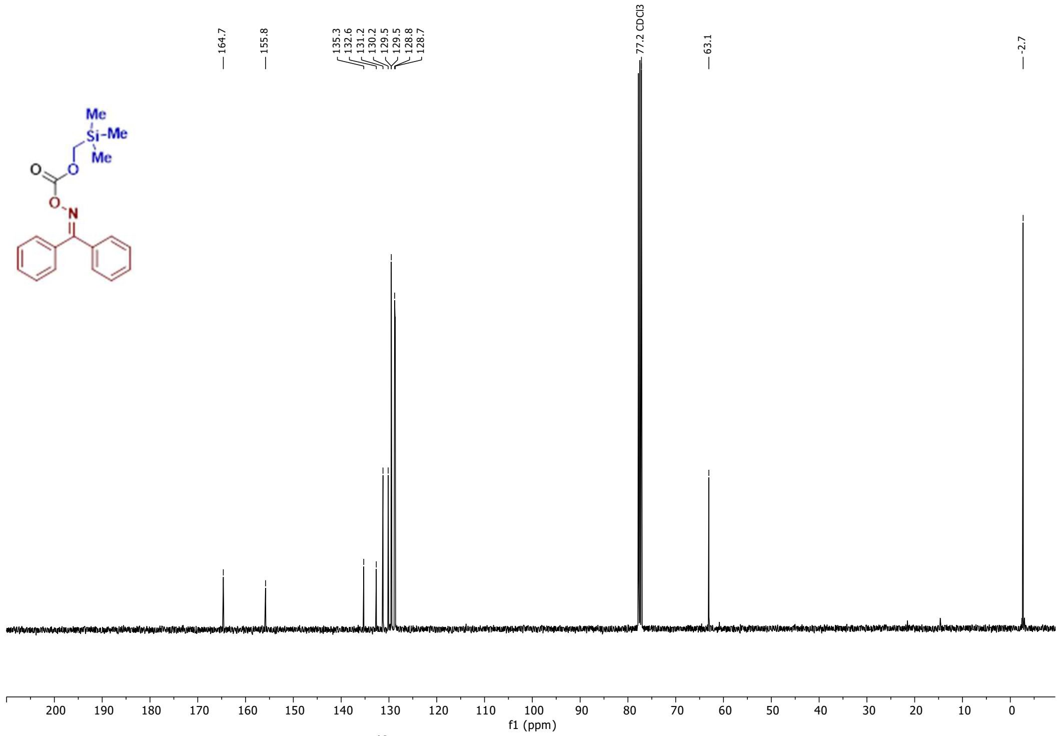

^{13}C NMR (101 MHz, $CDCl_3$)

9*H*-Fluoren-9-one O-(2-((dimethyl(phenyl)silyl)oxy)acetyl) oxime (2c)

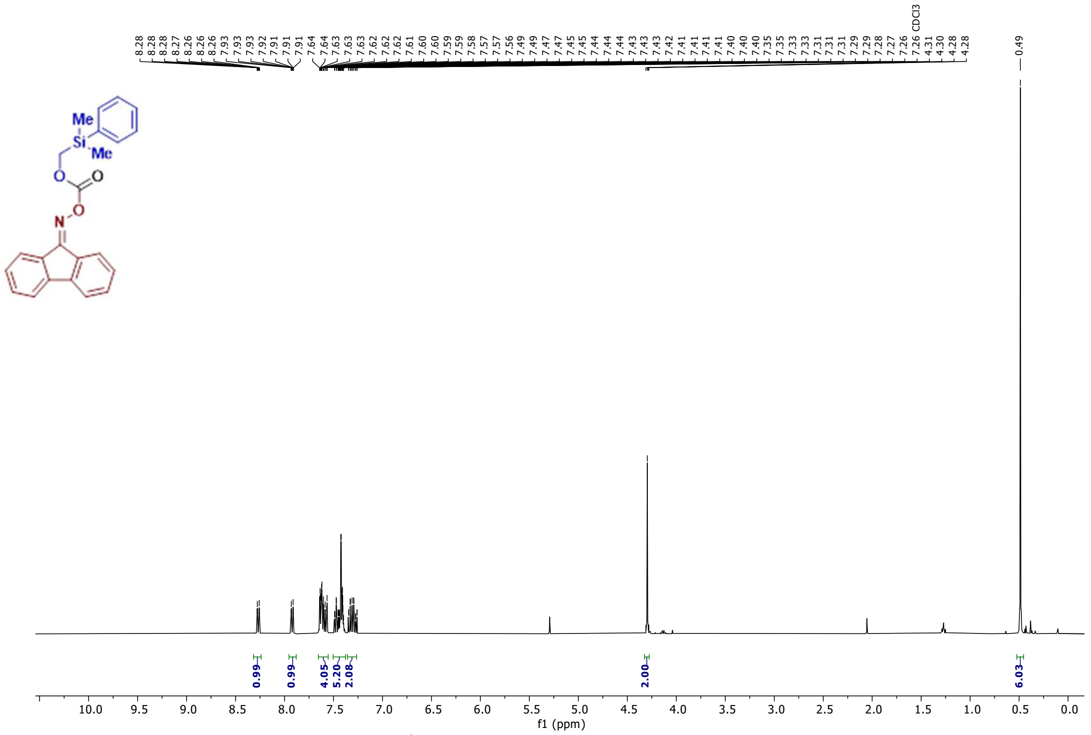

^{1}H NMR (400 MHz, $CDCl_3$)

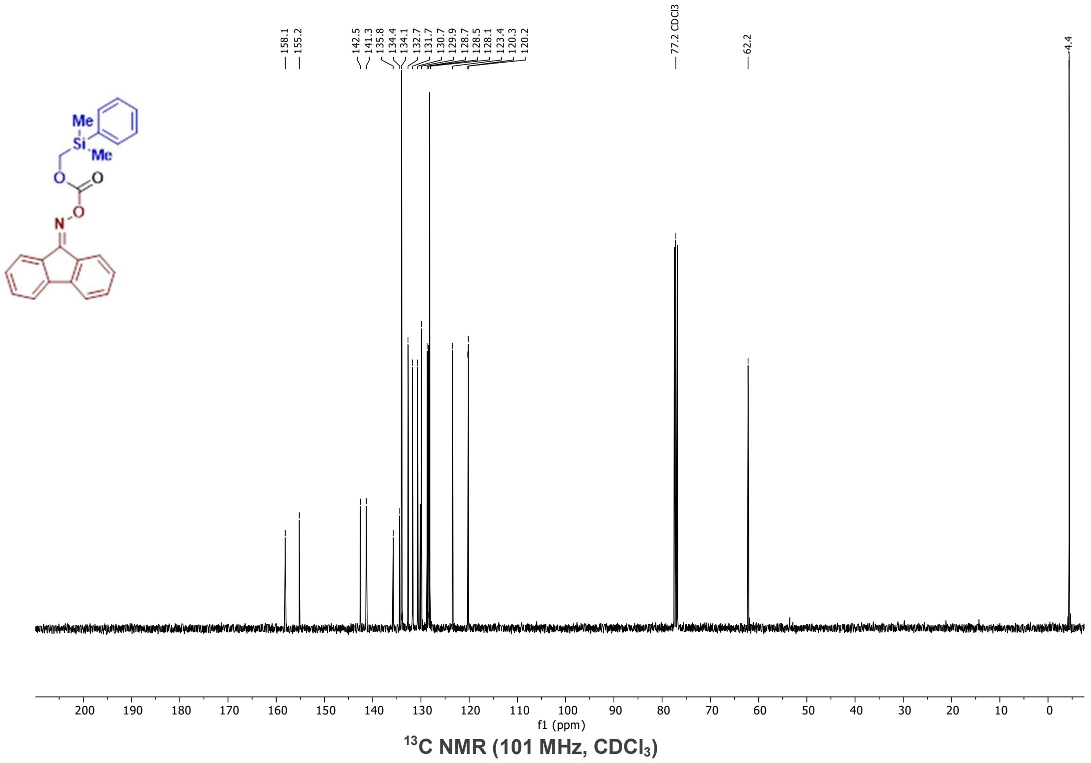

^{13}C NMR (101 MHz, $CDCl_3$)

Bis(3-(trifluoromethyl)phenyl)methanone O-(((dimethyl(phenyl)silyl)methoxy)carbonyl) oxime (2d)

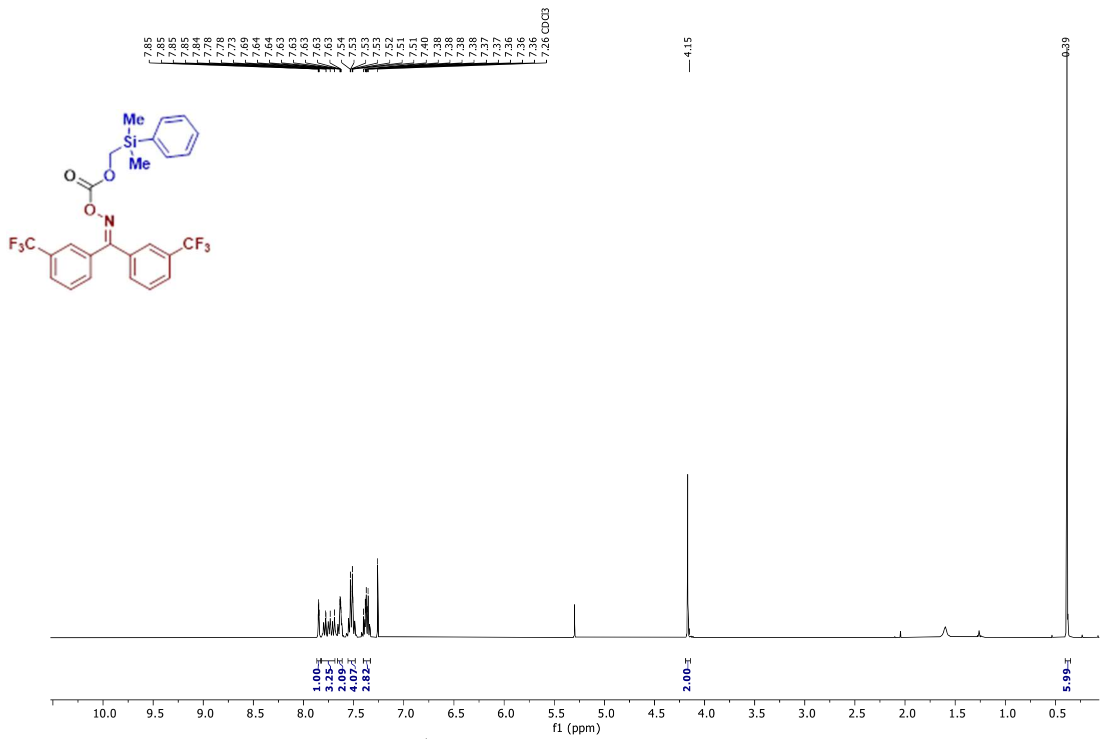

^{1}H NMR (400 MHz, CDCl$_3$)

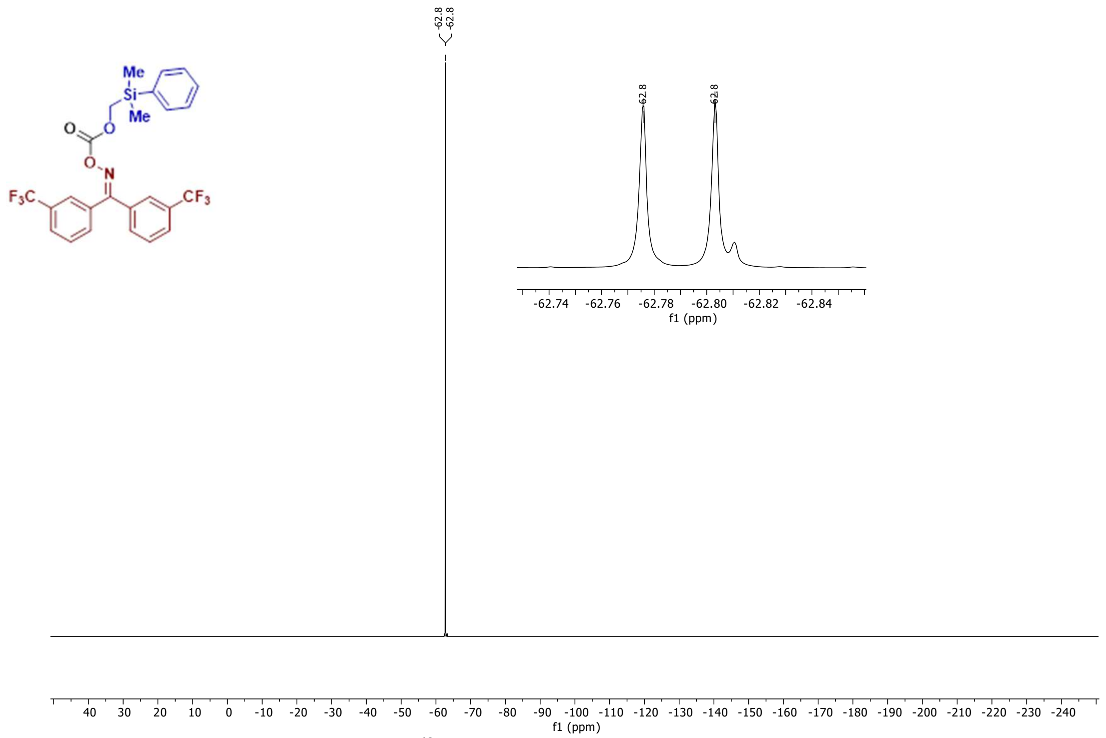

^{19}F NMR (376 MHz, CDCl$_3$)

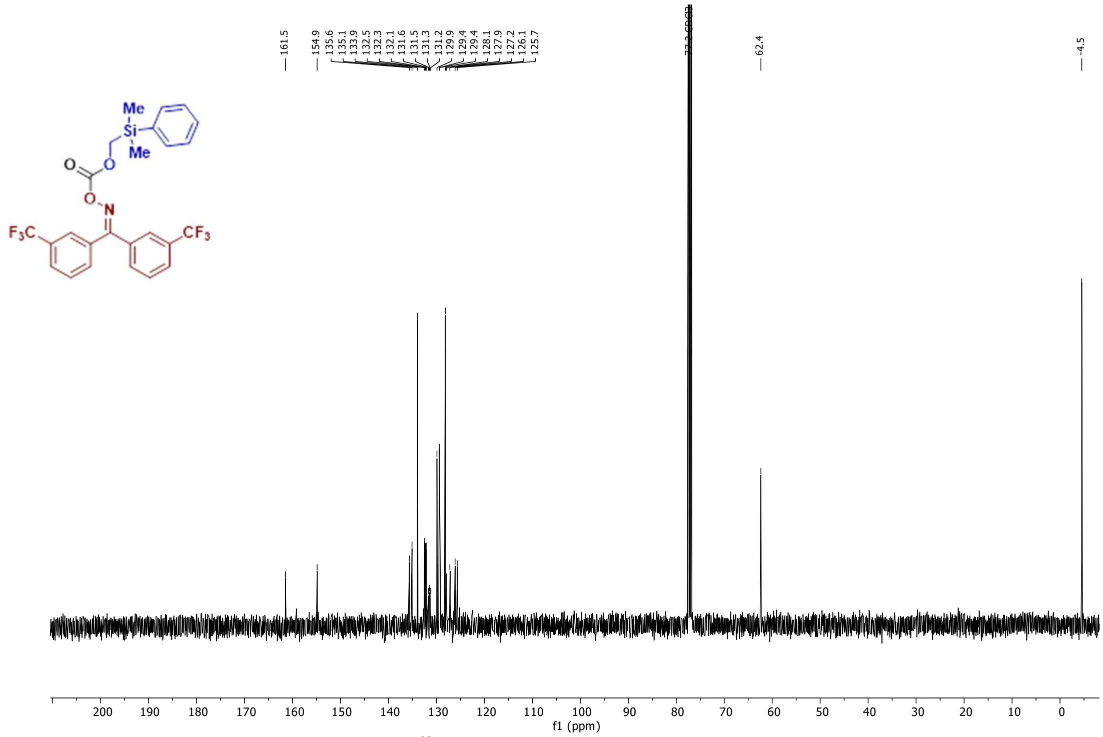

^{13}C NMR (101 MHz, $CDCl_3$)

Bis(4-chlorophenyl)methanone O-(((dimethyl(phenyl)silyl)methoxy)carbonyl) oxime (2e)

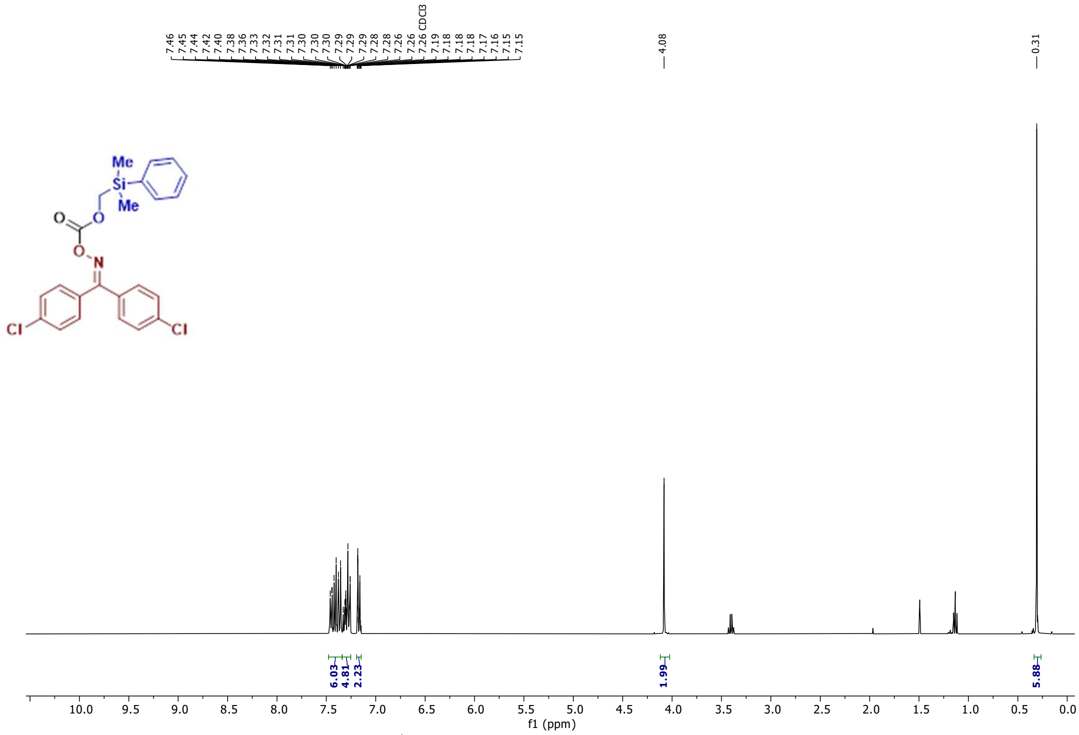

^{1}H NMR (400 MHz, $CDCl_3$)

Note: Small peaks of trapped EtOAc in ^{1}H spectra. Spectra produced after 2 nights of high vacuum drying.

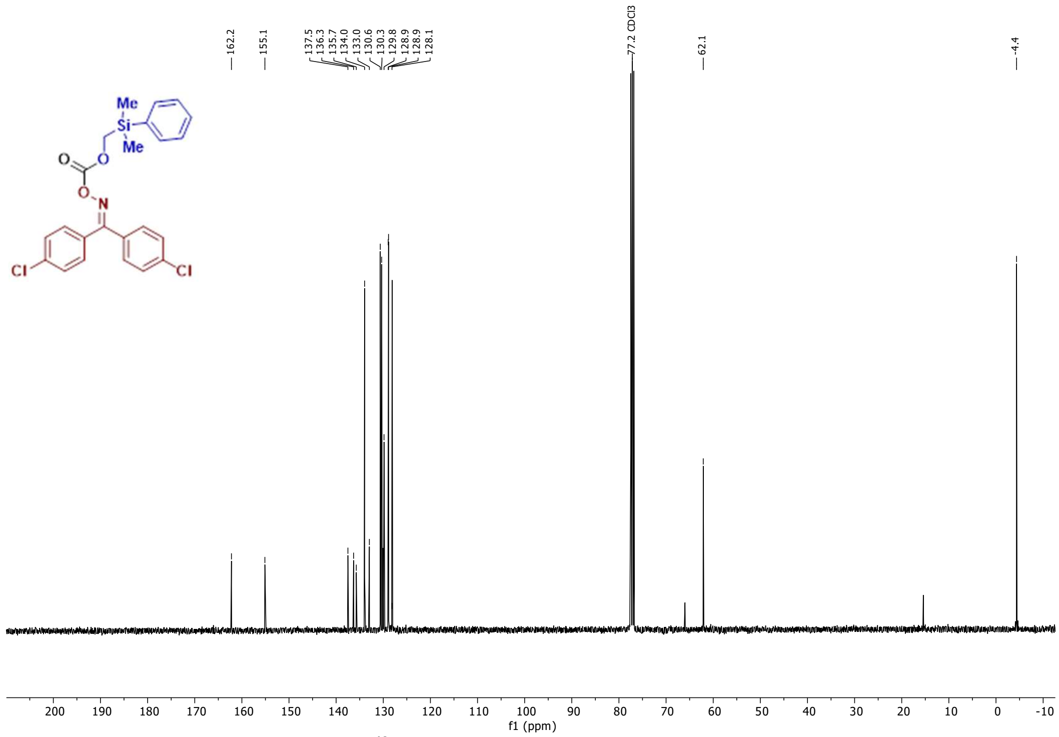

^{13}C NMR (101 MHz, $CDCl_3$)

Bis(4-methoxyphenyl)methanone O-(((dimethyl(phenyl)silyl)methoxy)carbonyl) oxime (2f)

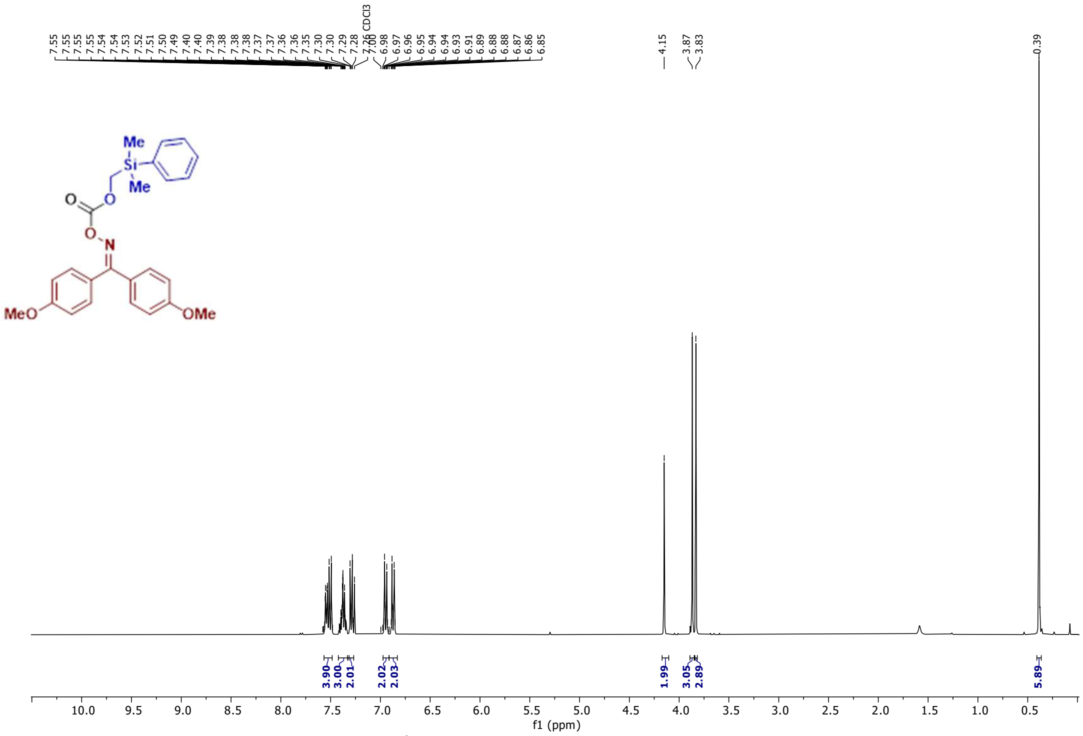

^{1}H NMR (400 MHz, $CDCl_3$)

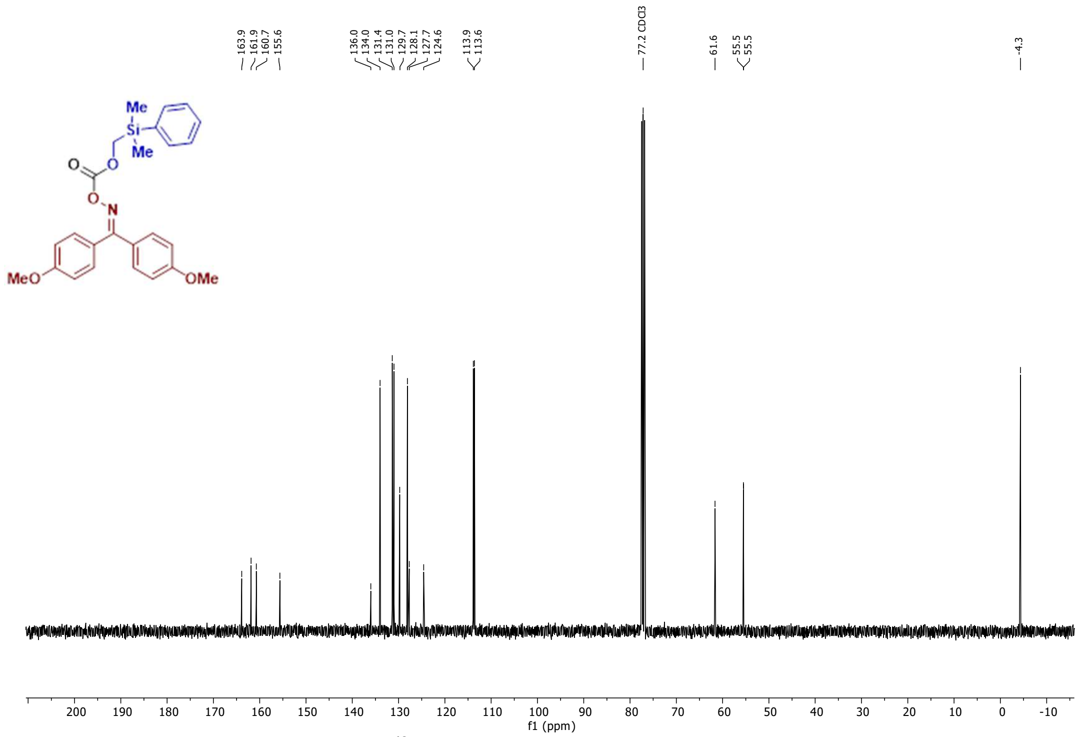

^{13}C NMR (101 MHz, $CDCl_3$)

4-((Dimethyl(phenyl)silyl)oxy)-2-((diphenylmethylene)amino)butanenitrile (3a)

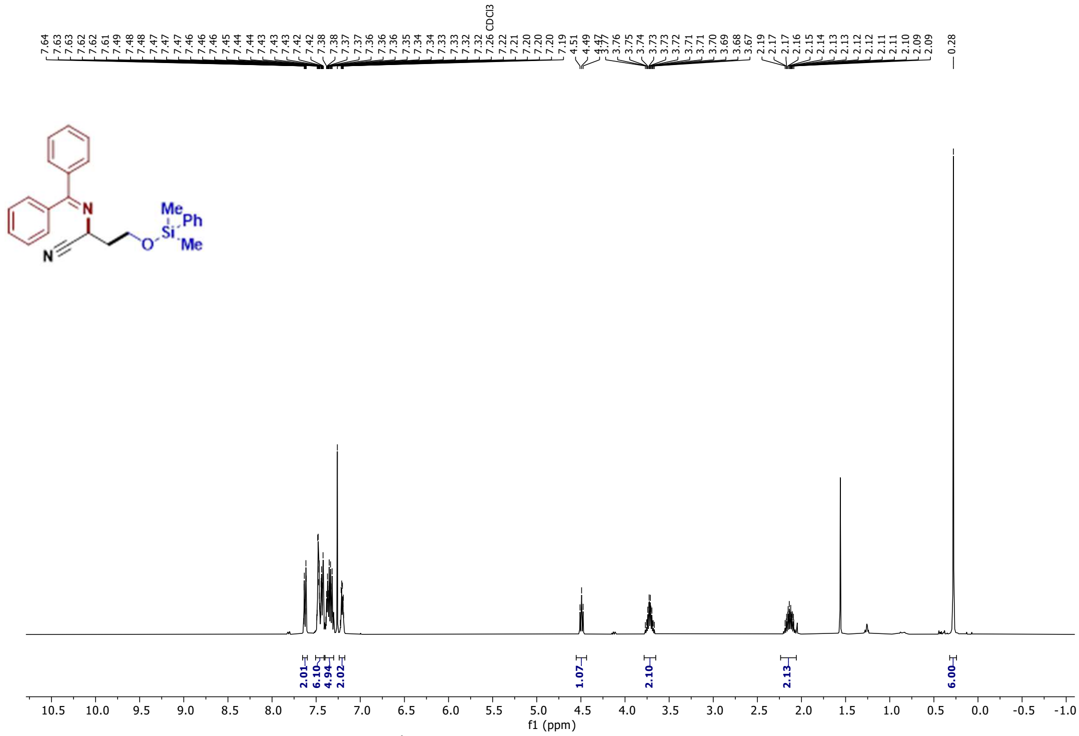

^{1}H NMR (400 MHz, $CDCl_3$)

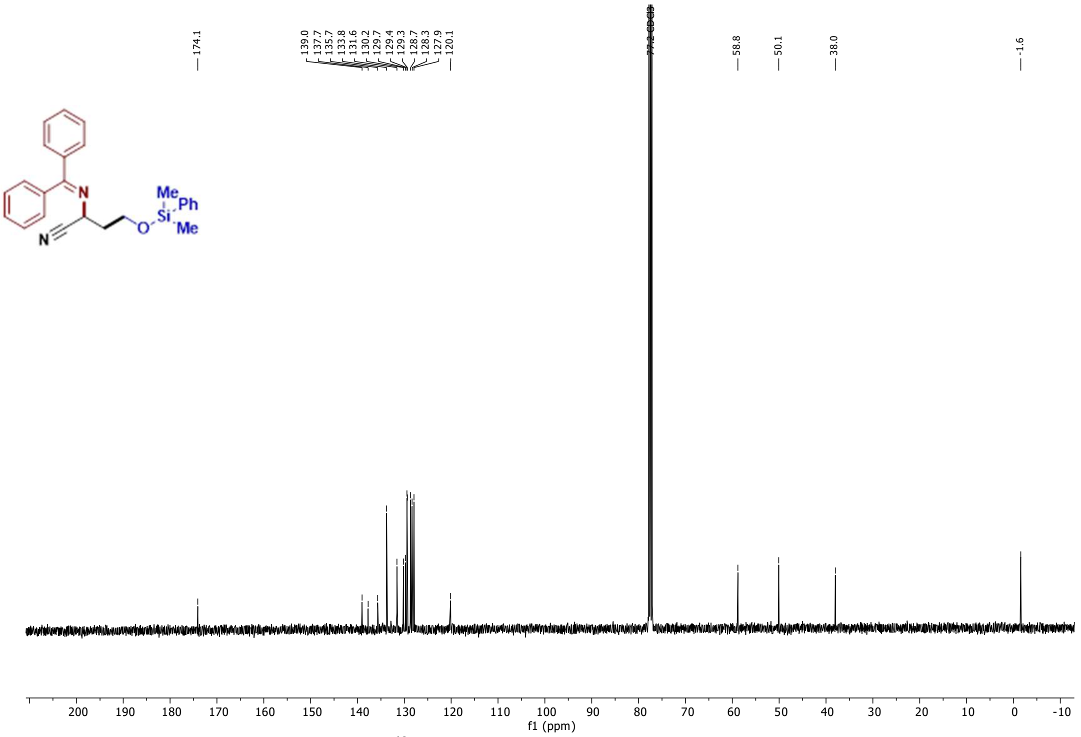

^{13}C NMR (101 MHz, $CDCl_3$)

2-((Diphenylmethylene)amino)-4-((trimethylsilyl)oxy)butanenitrile (3b)

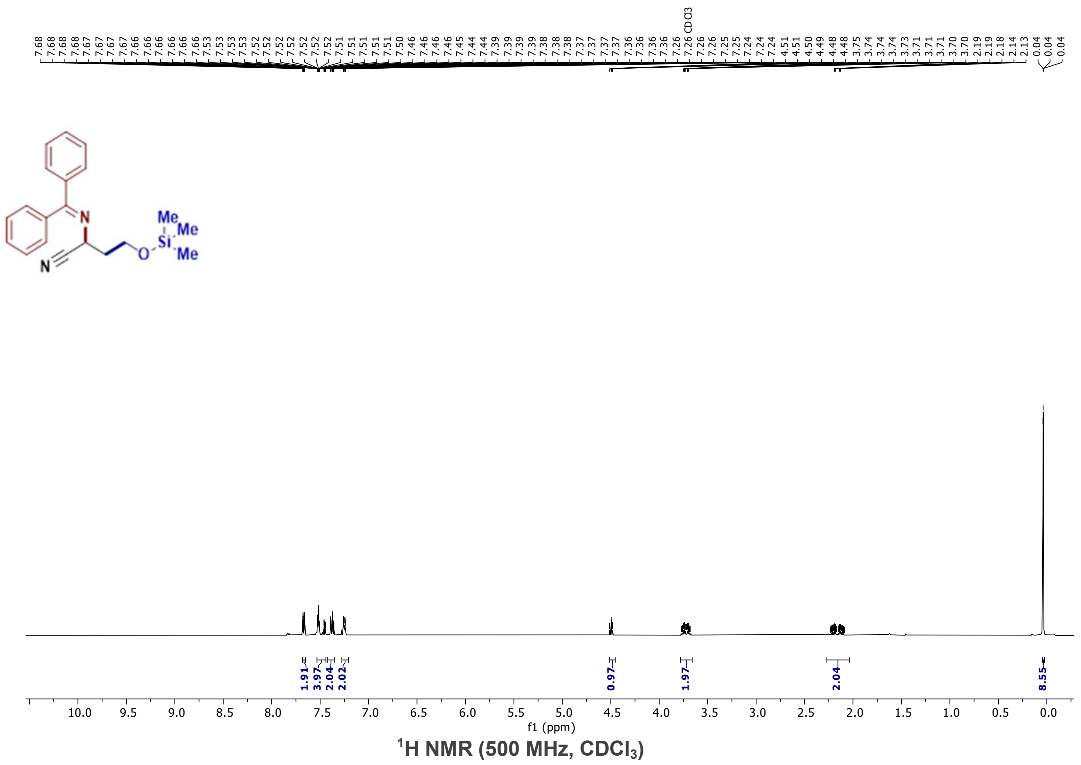

^{1}H NMR (500 MHz, $CDCl_3$)

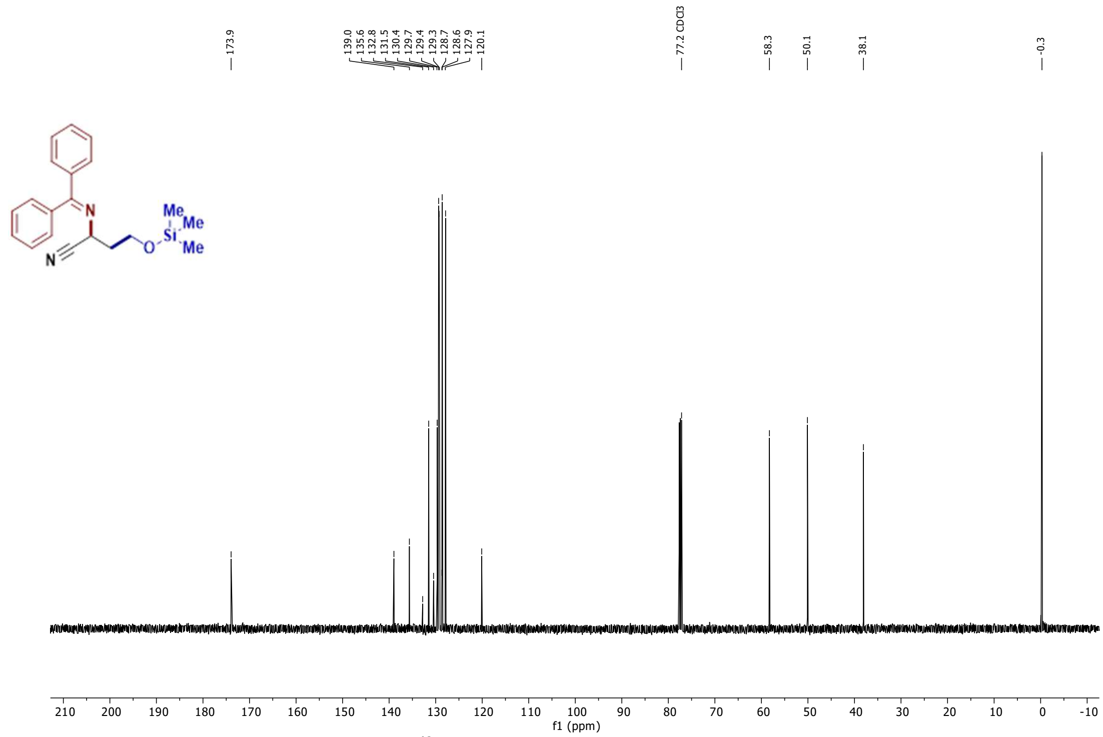

^{13}C NMR (126 MHz, $CDCl_3$)

2-((Bis(3-(trifluoromethyl)phenyl)methylene)amino)-4-((dimethyl(phenyl)silyl)oxy)butanenitrile (3d)

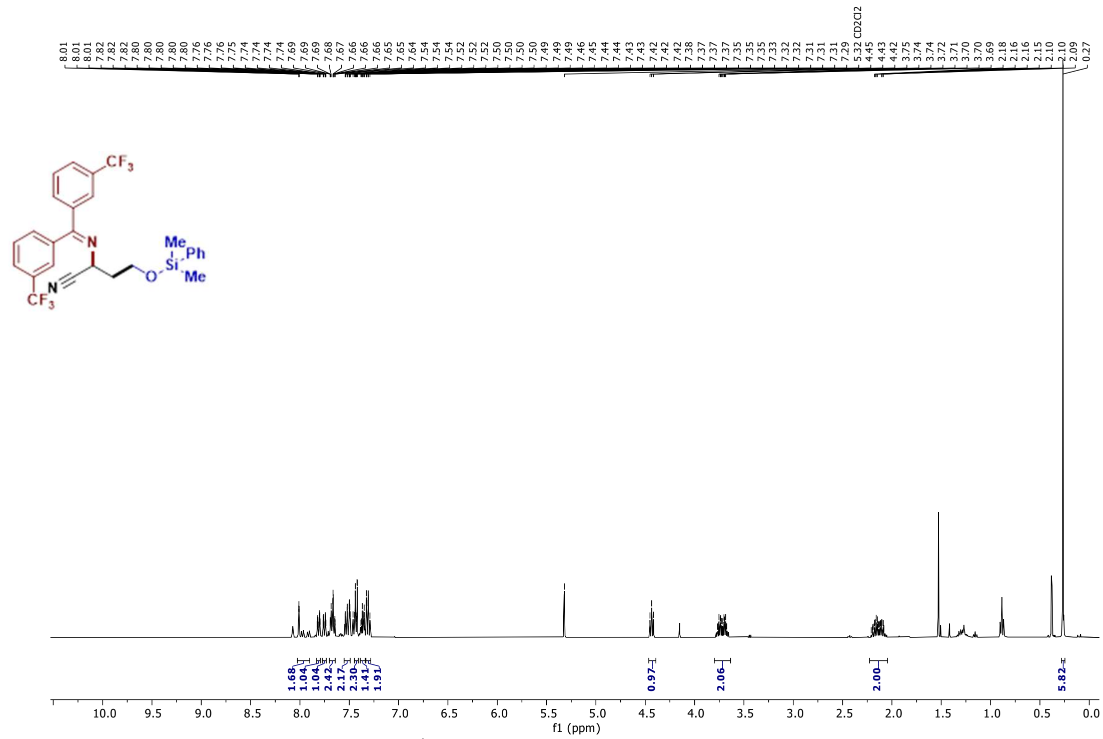

^{1}H NMR (400 MHz, CD_2Cl_2)

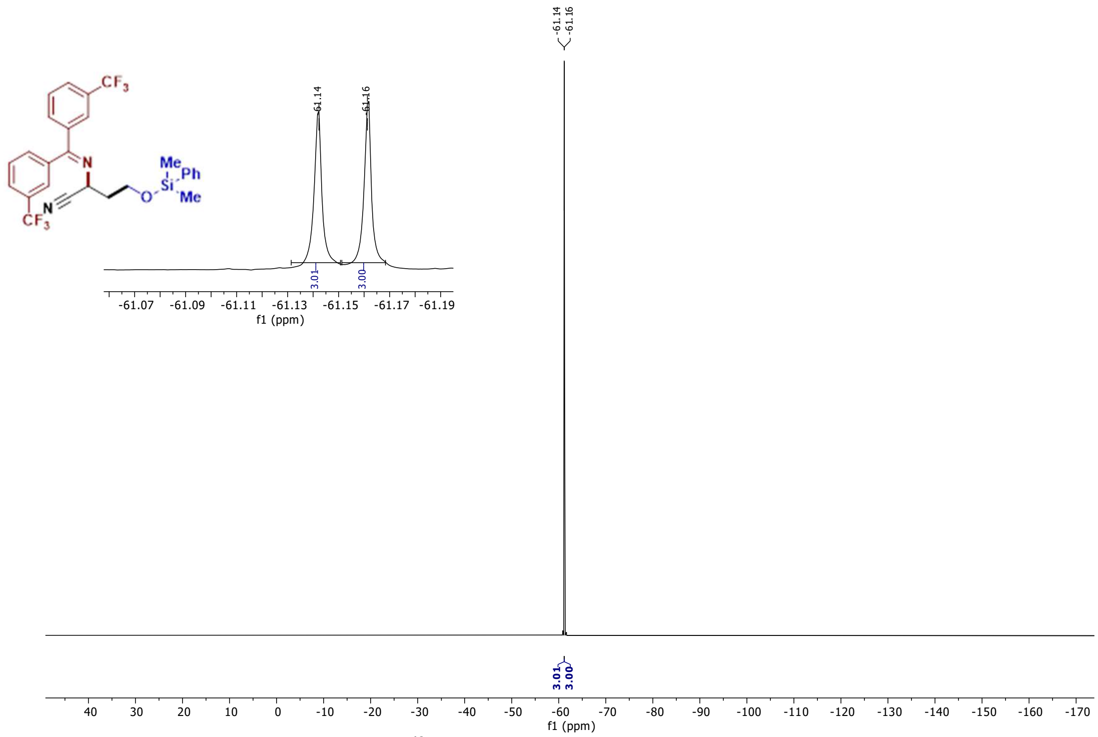

^{19}F NMR (400 MHz, CD_2Cl_2)

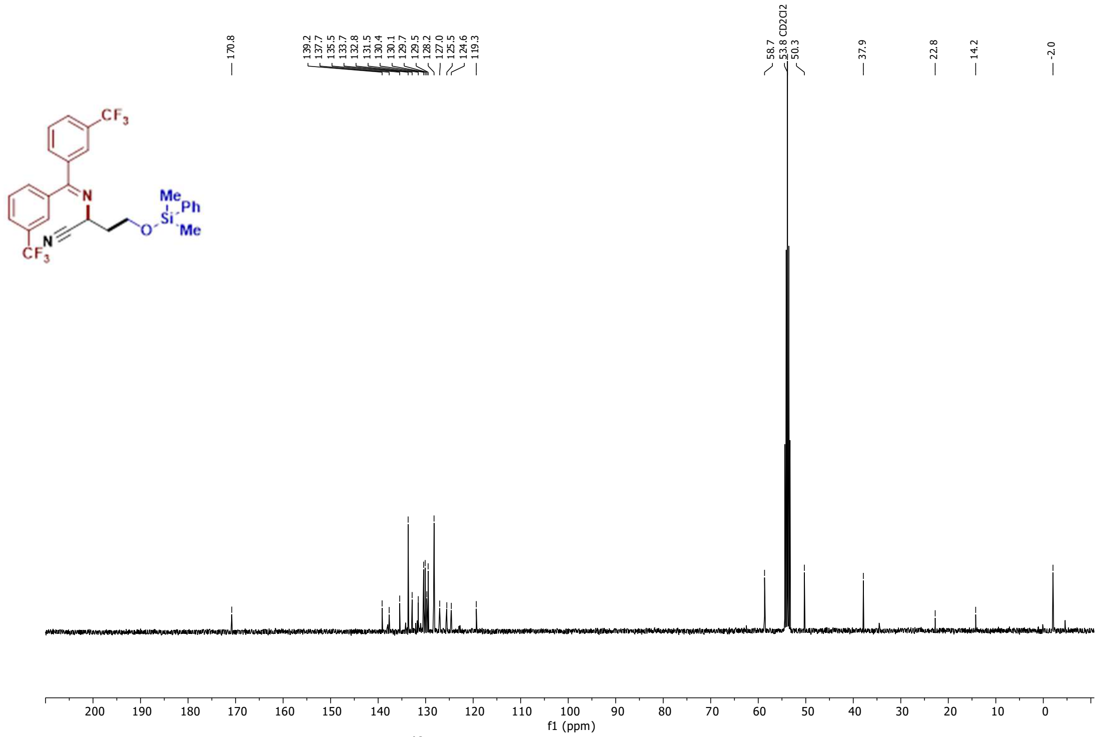

^{13}C NMR (101 MHz, CD_2Cl_2)

2-((Bis(4-chlorophenyl)methylene)amino)-4-((dimethyl(phenyl)silyl)oxy)butanenitrile (3e)

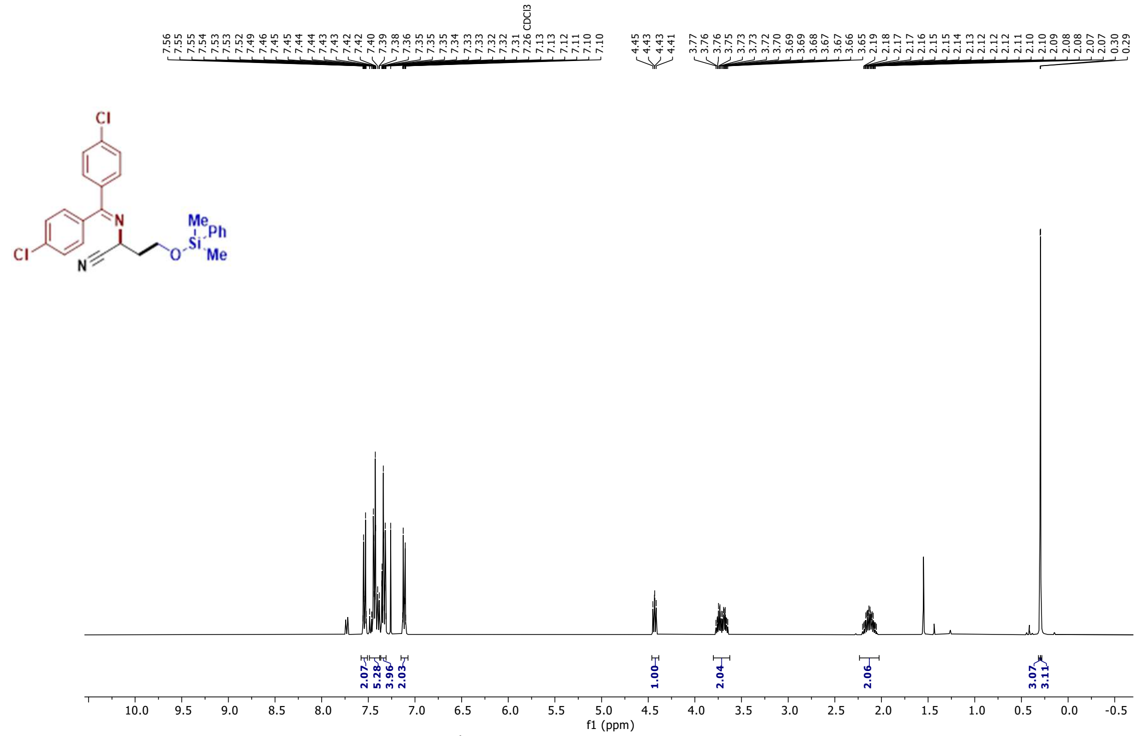

^{1}H NMR (400 MHz, $CDCl_3$)

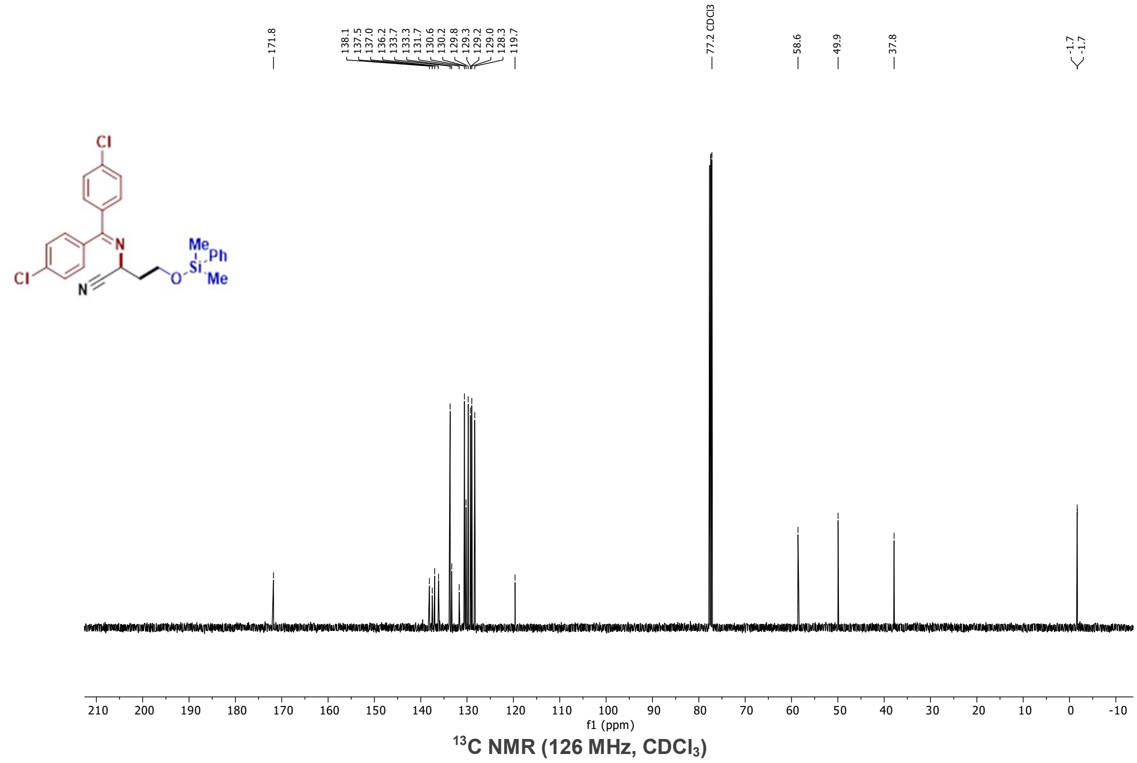

^{13}C NMR (126 MHz, $CDCl_3$)

***N*-(3-((Dimethyl(phenyl)silyl)oxy)-1-phenylpropyl)-1,1-diphenylmethanimine(3g)**

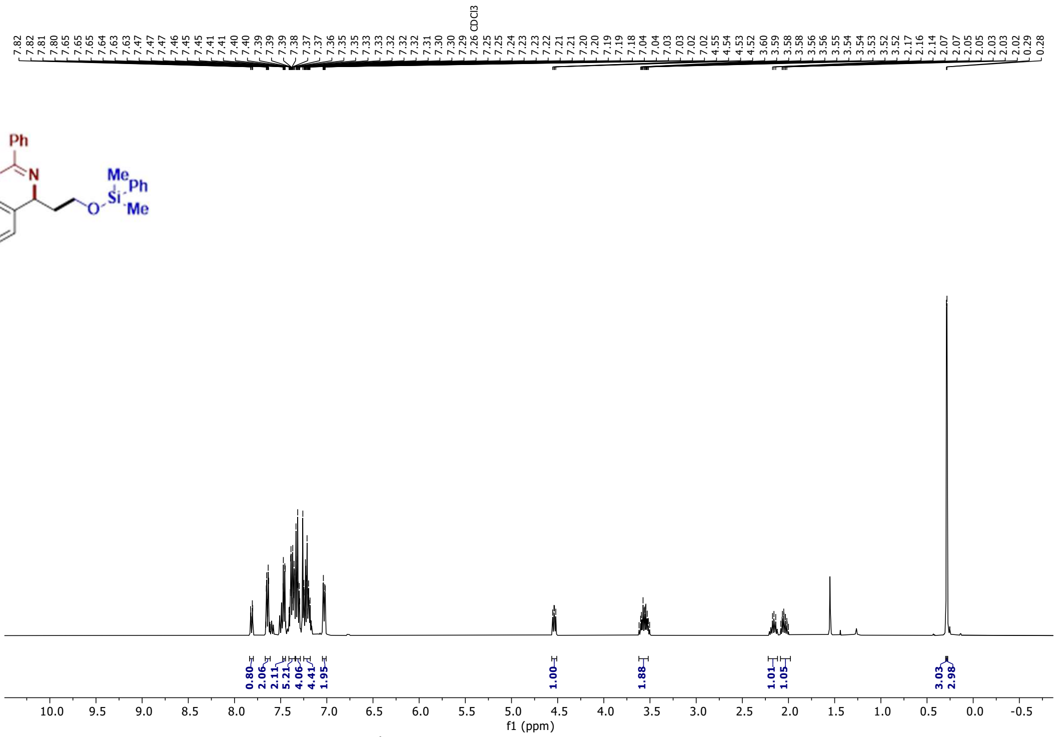

^{1}H NMR (400 MHz, $CDCl_3$)

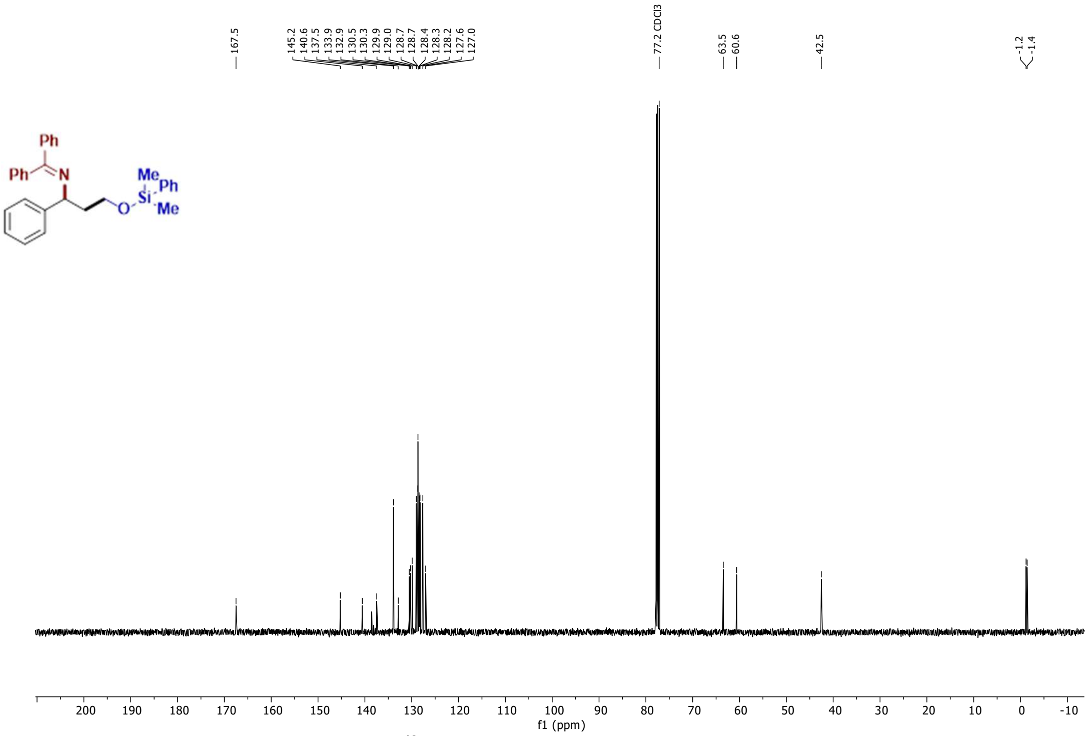

^{13}C NMR (126 MHz, $CDCl_3$)

3-Amino-3-phenylpropan-1-ol hydrochloride (3g')

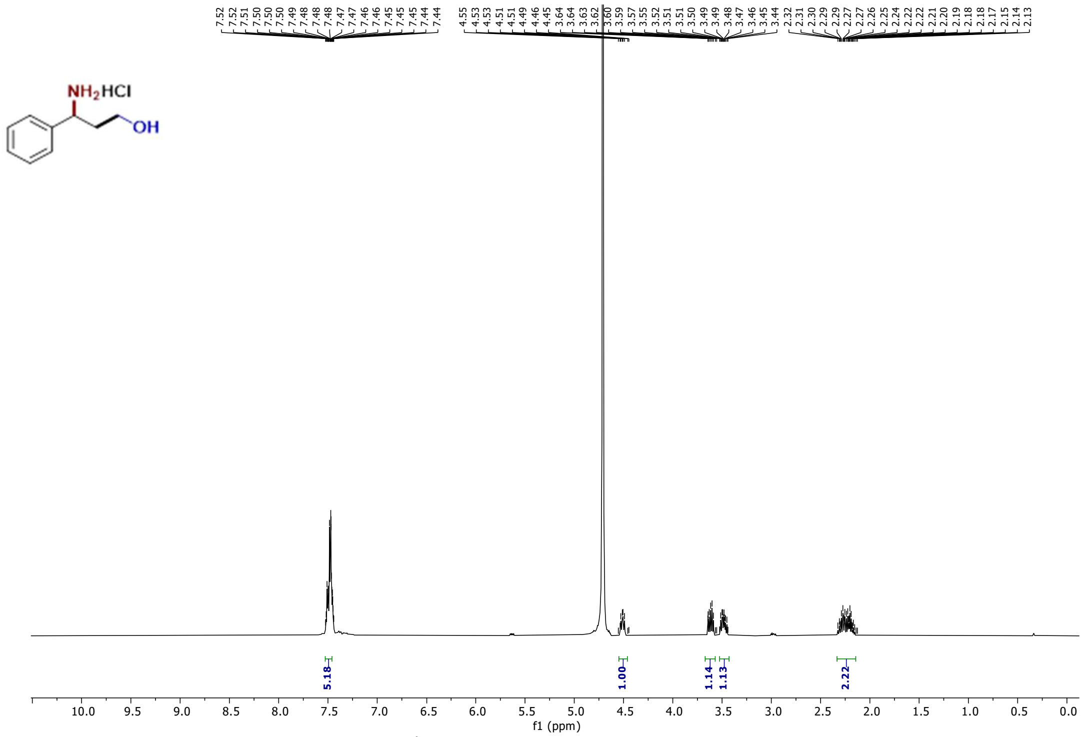

^{1}H NMR (400 MHz, D_2O)

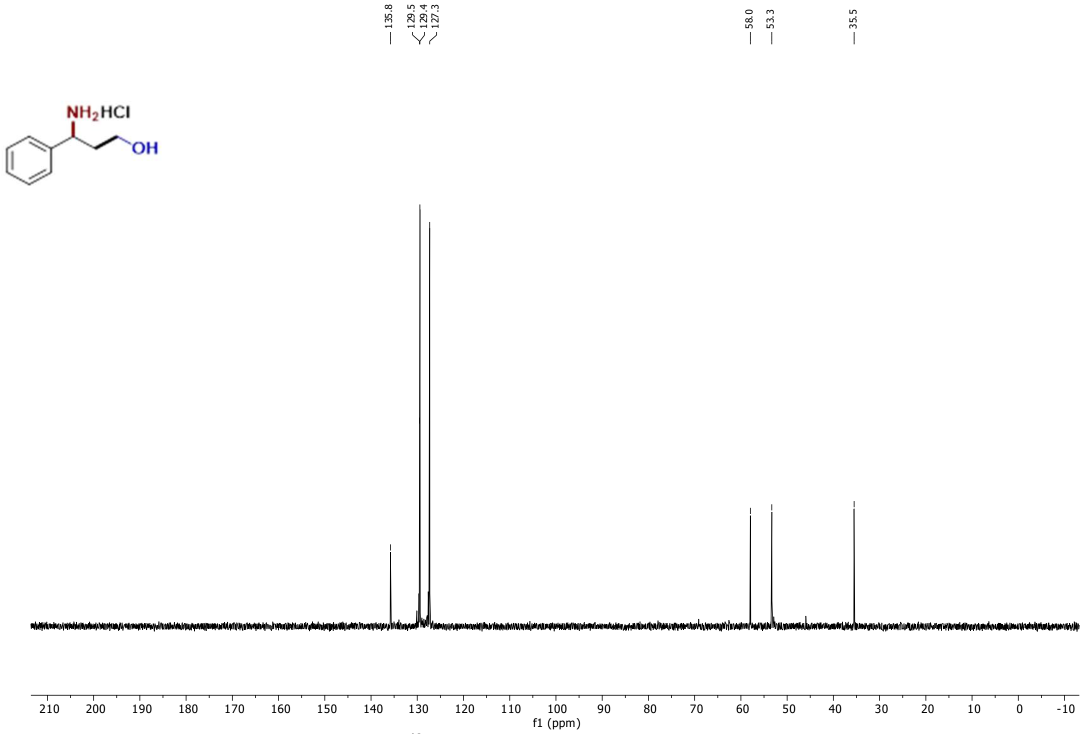

^{13}C NMR (101 MHz, D_2O)

N-(3-((Dimethyl(phenyl)silyl)oxy)-1-(4-methoxyphenyl)propyl)-1,1-diphenylmethanimine (3h)

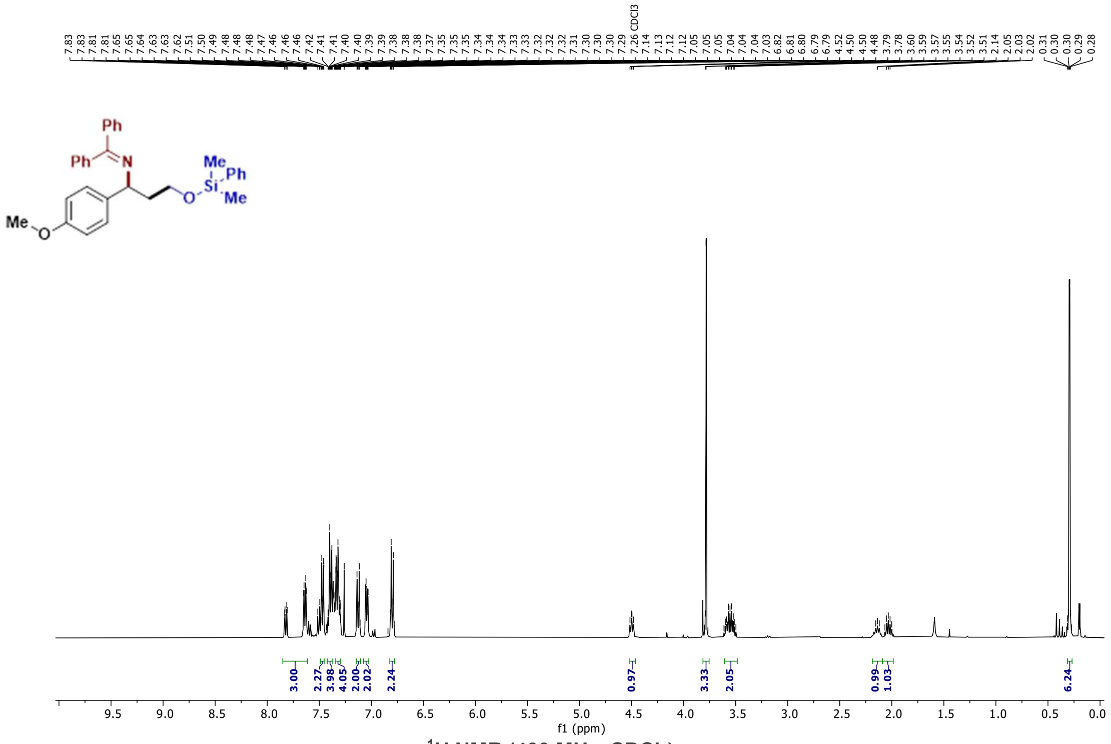

^{1}H NMR (400 MHz, $CDCl_3$)

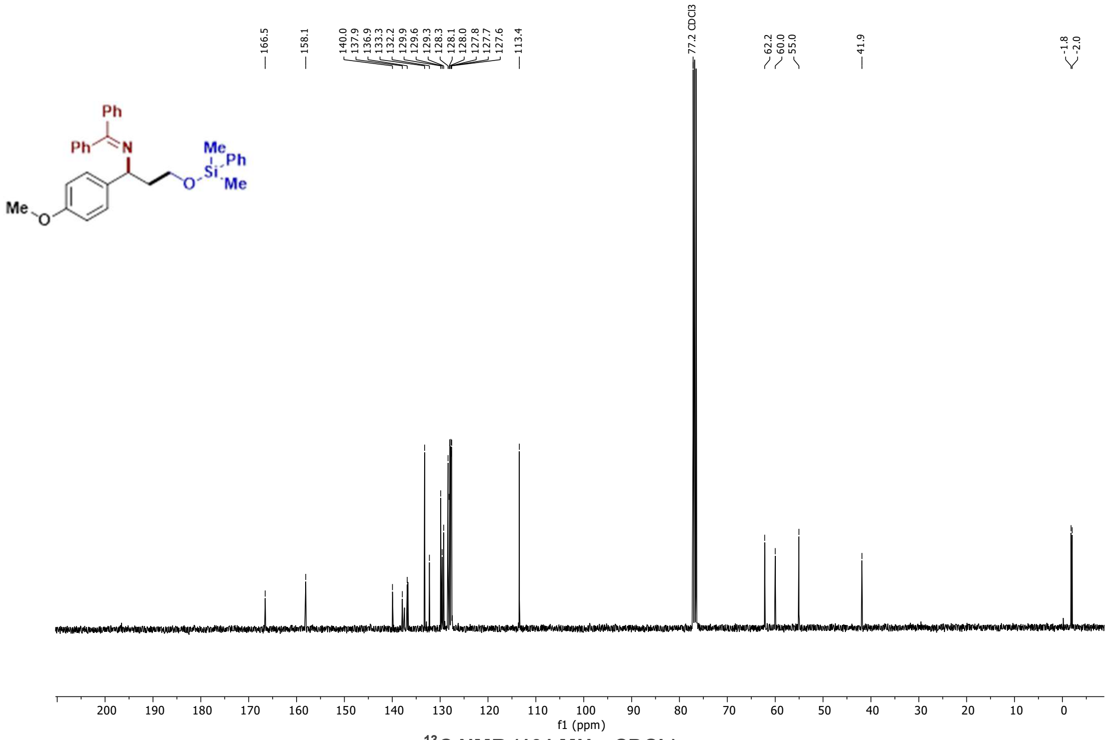

^{13}C NMR (101 MHz, $CDCl_3$)

4-(3-((Dimethyl(phenyl)silyl)oxy)-1-((diphenylmethylene)amino)propyl)phenyl acetate (3i)

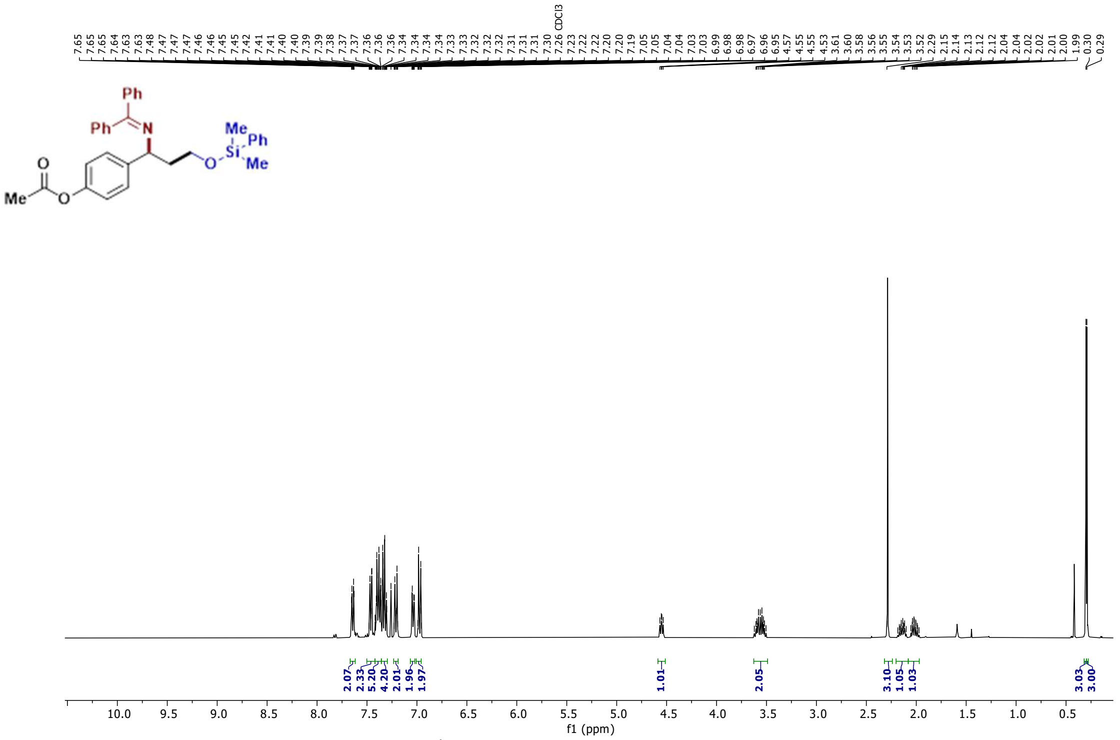

^{1}H NMR (400 MHz, $CDCl_3$)

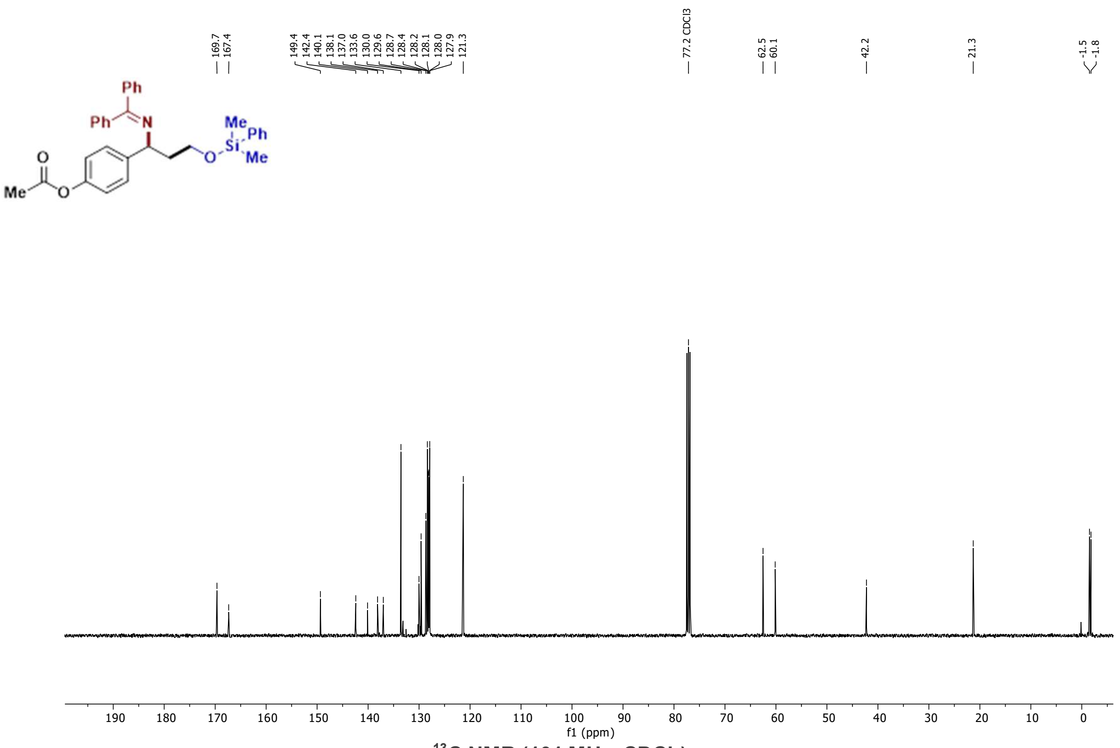

^{13}C NMR (101 MHz, $CDCl_3$)

4-(3-((Dimethyl(phenyl)silyl)oxy)-1-((diphenylmethylene)amino)propyl)benzonitrile (3j)

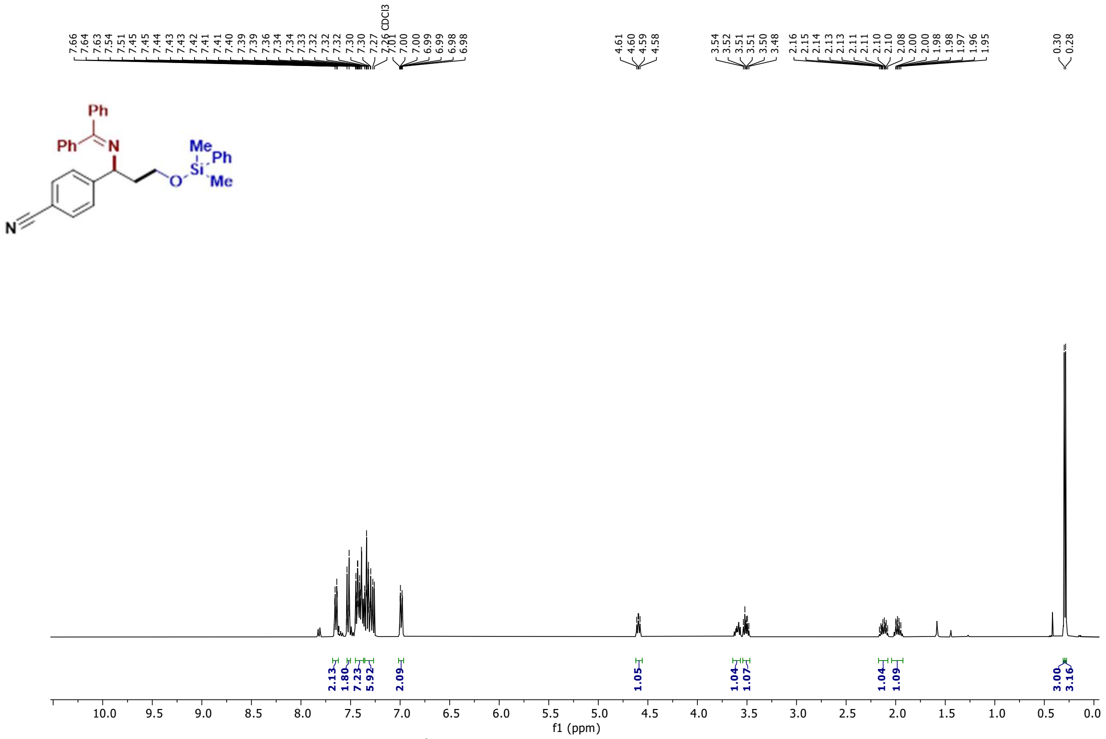

^{1}H NMR (400 MHz, CDCl$_3$)

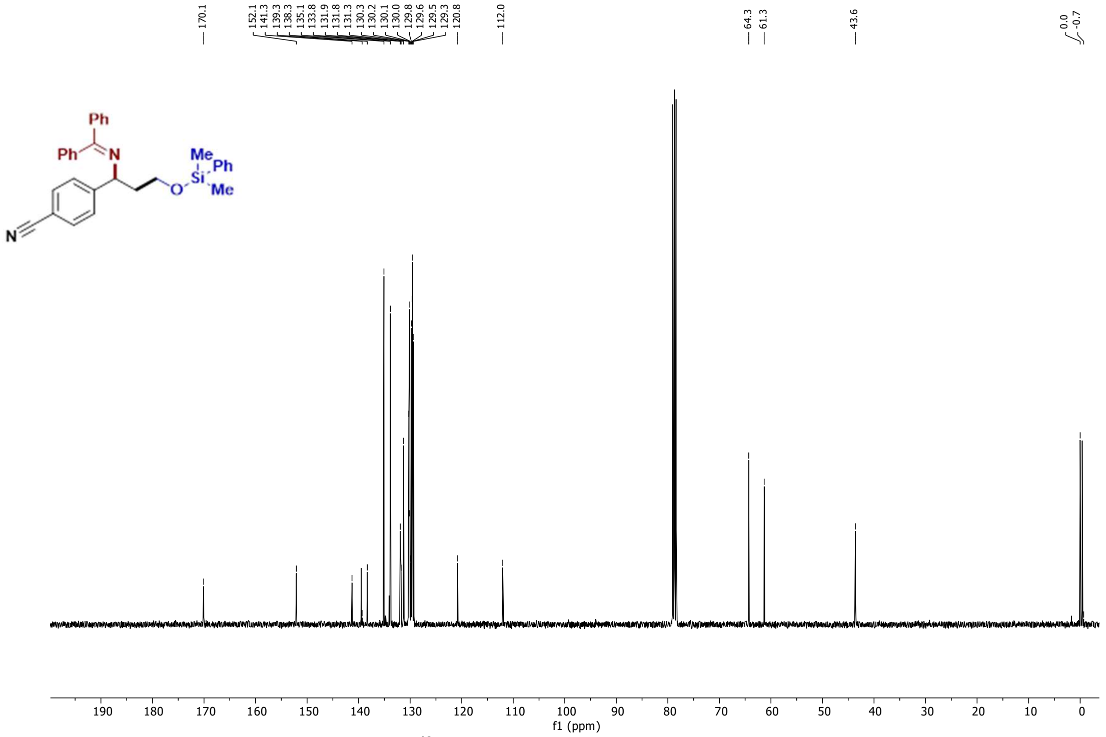

^{13}C NMR (101 MHz, CDCl$_3$)

3-([1,1'-Biphenyl]-4-yl)-3-aminopropan-1-ol hydrochloride (3k)

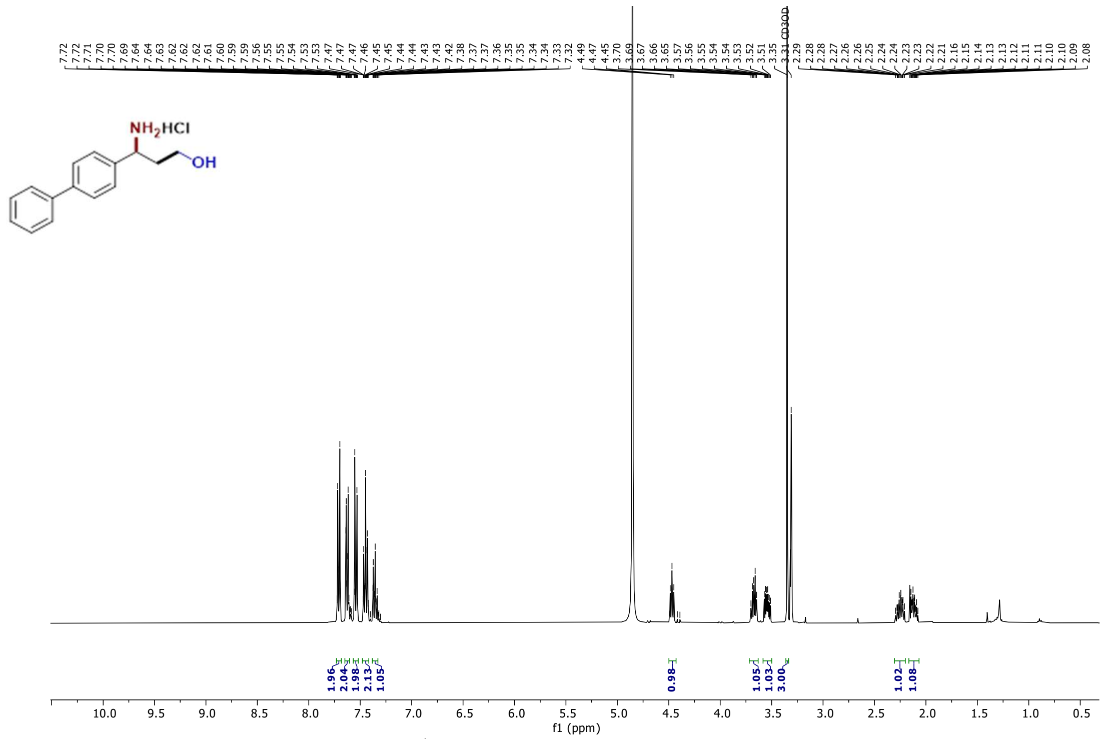

^{1}H NMR (400 MHz, MeOD)

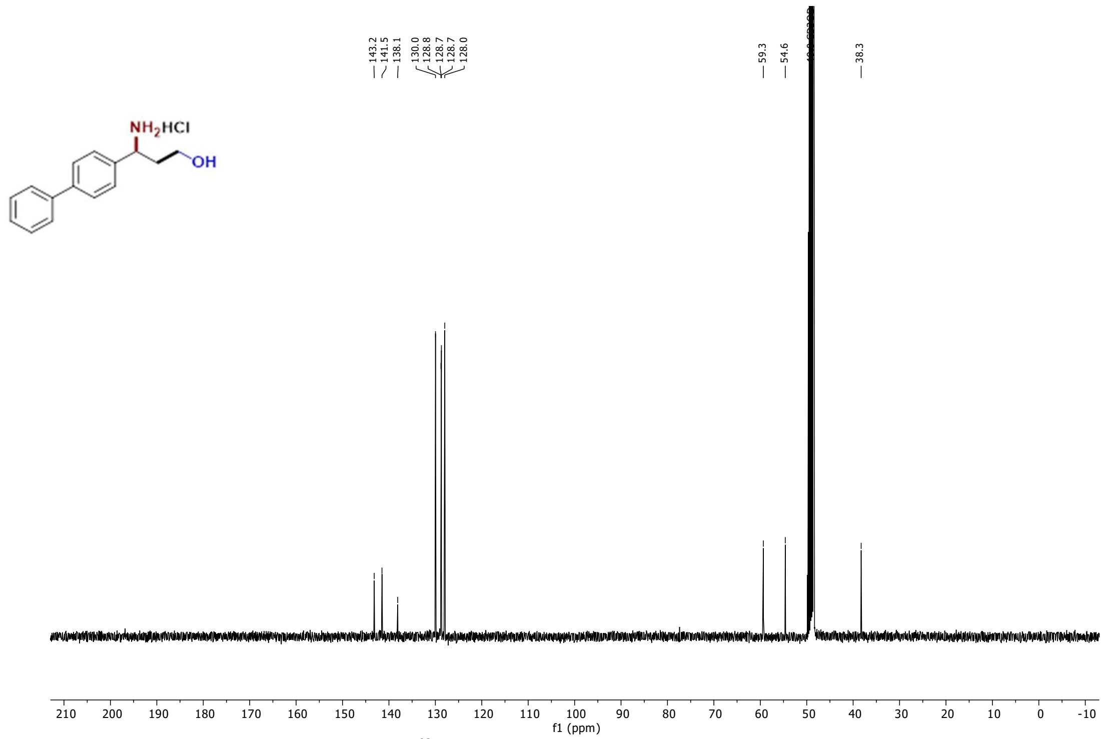

^{13}C NMR (101 MHz, MeOD)

3-Amino-3-(3-chlorophenyl)propan-1-ol hydrochloride (3l)

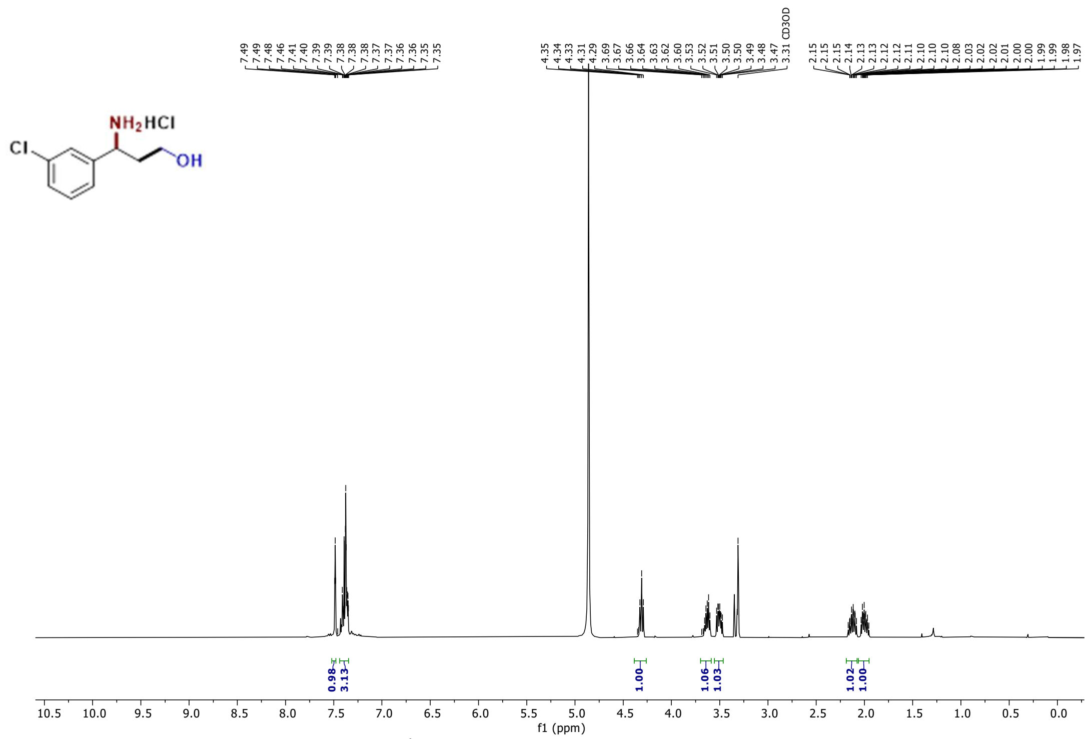

^{1}H NMR (400 MHz, MeOD)

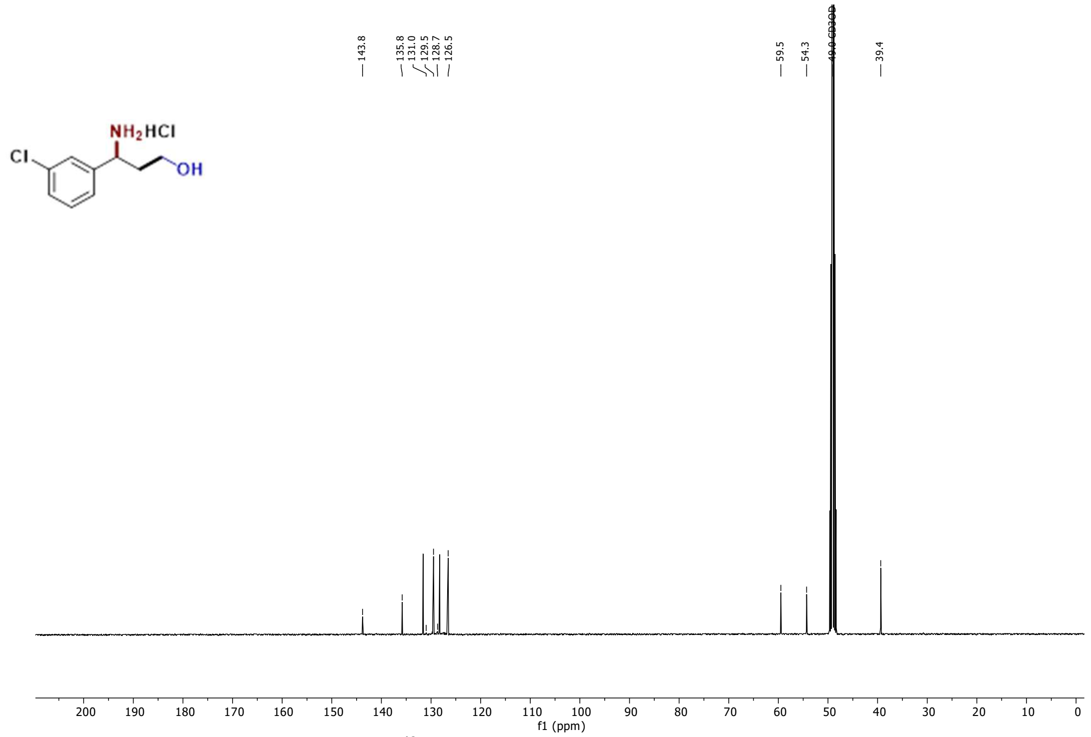

^{13}C NMR (101 MHz, MeOD)

N-(3-((Dimethyl(phenyl)silyl)oxy)-1-(pyridin-4-yl)propyl)-1,1-diphenylmethanimine (3m)

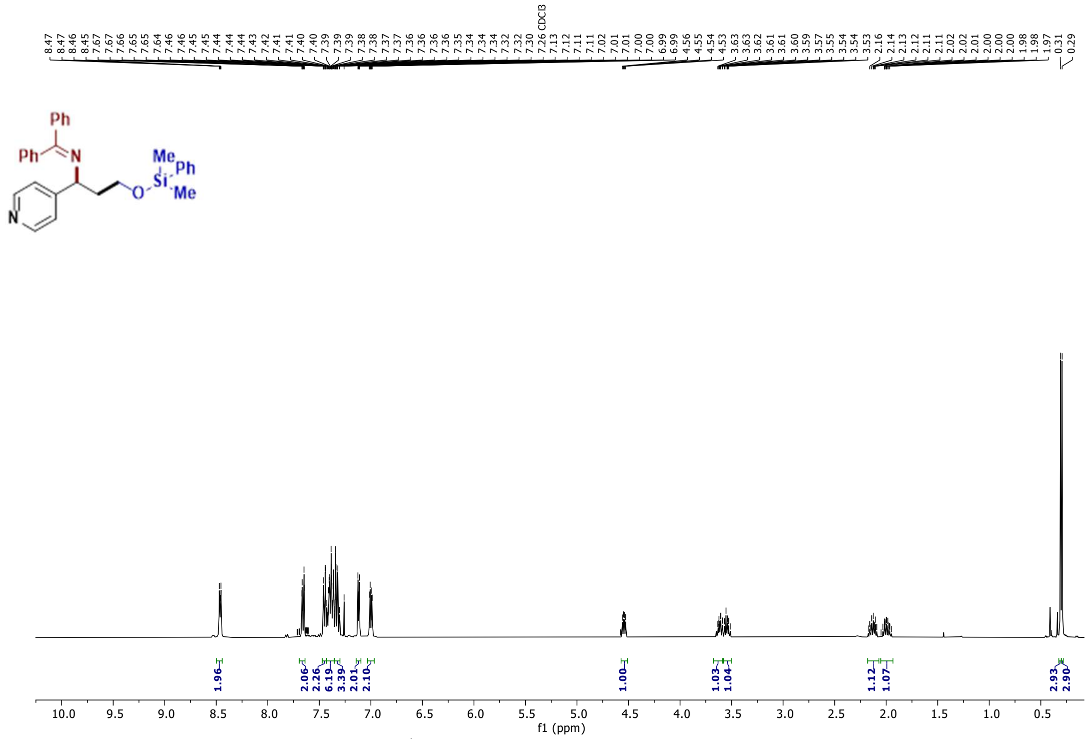

^{1}H NMR (400 MHz, CDCl$_3$)

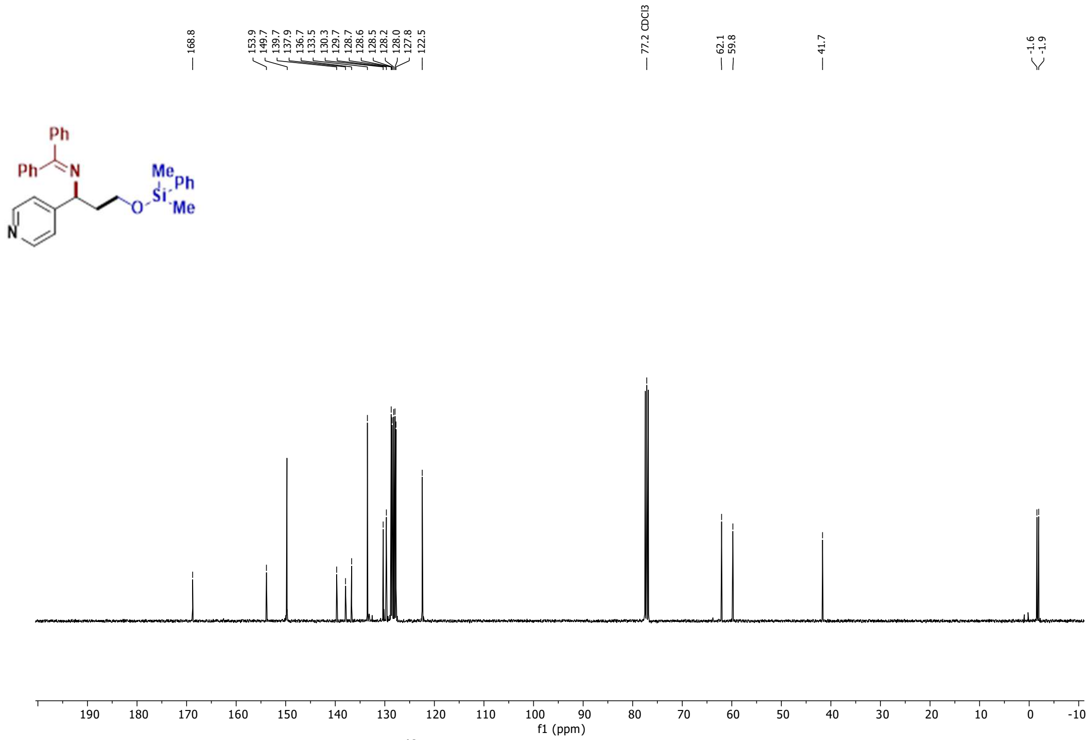

^{13}C NMR (101 MHz, CDCl$_3$)

N-(3-((Dimethyl(phenyl)silyl)oxy)-1-(4-methylthiazol-5-yl)propyl)-1,1-diphenylmethanimine (3n)

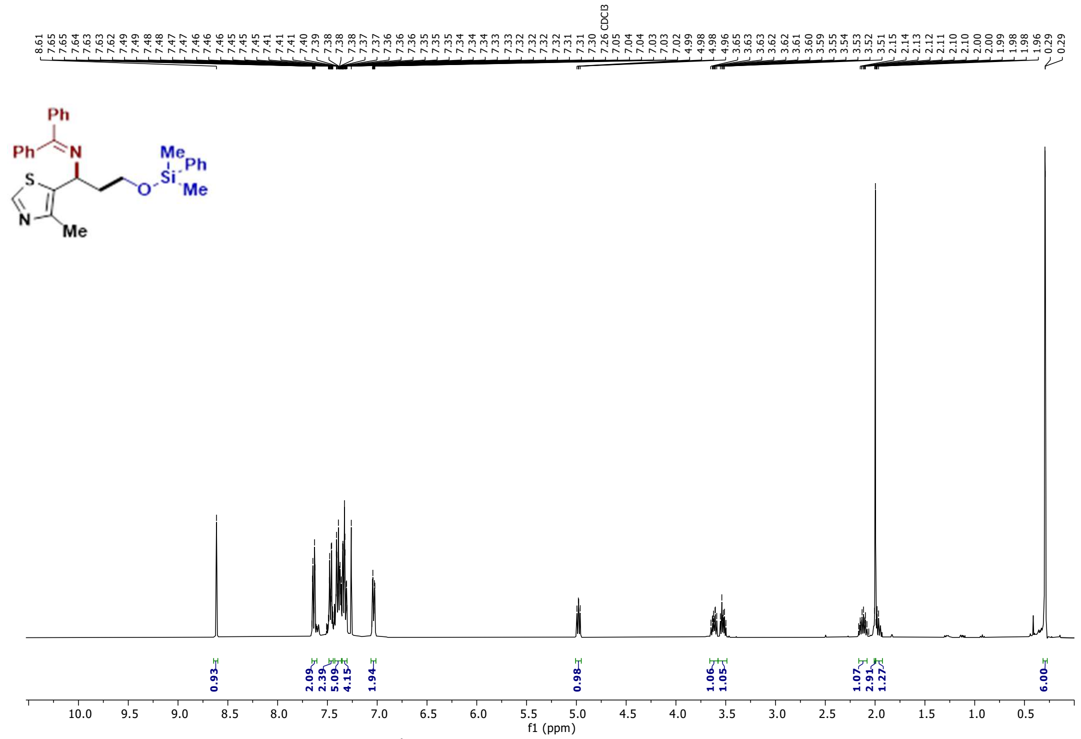

^{1}H NMR (400 MHz, CDCl$_3$)

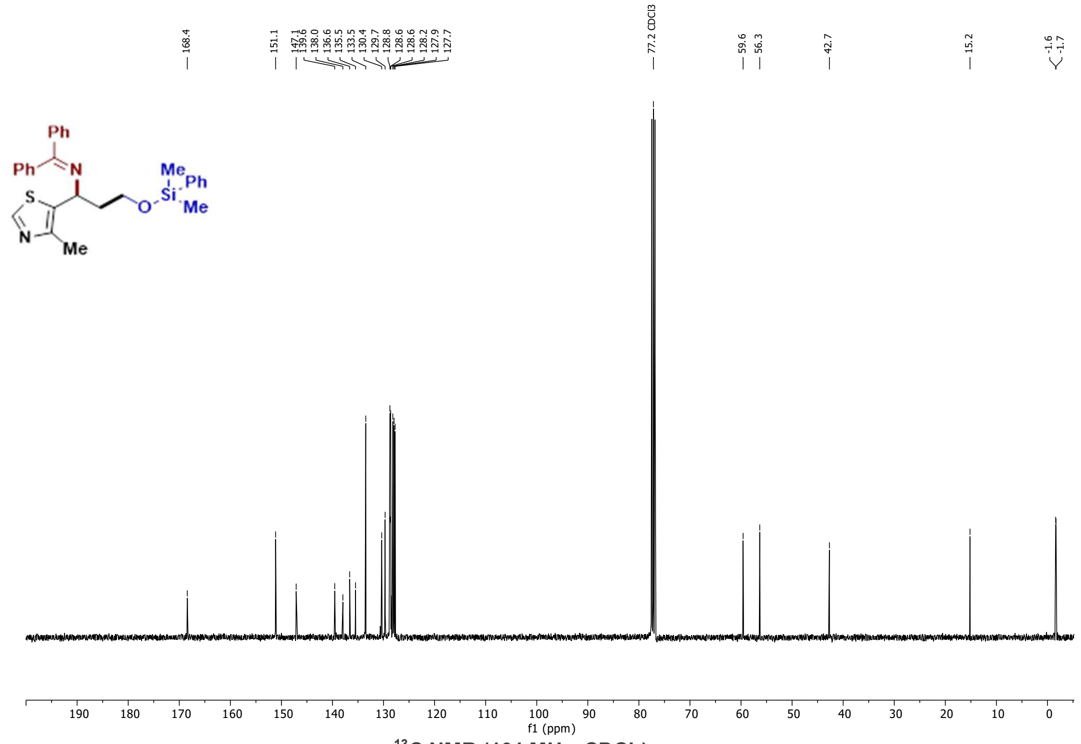

^{13}C NMR (101 MHz, CDCl$_3$)

***N*-(3-((Dimethyl(phenyl)silyl)oxy)-1-(perfluorophenyl)propyl)-1,1-diphenylmethanimine (3o)**

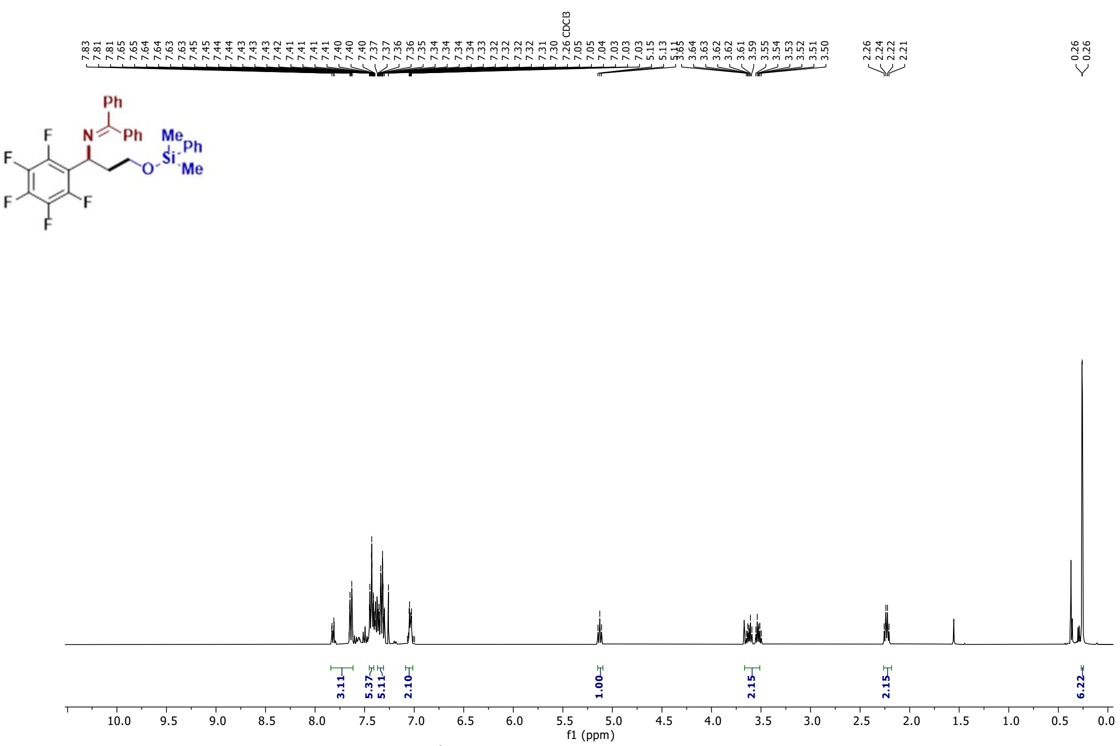

^{1}H NMR (400 MHz, CDCl$_3$)

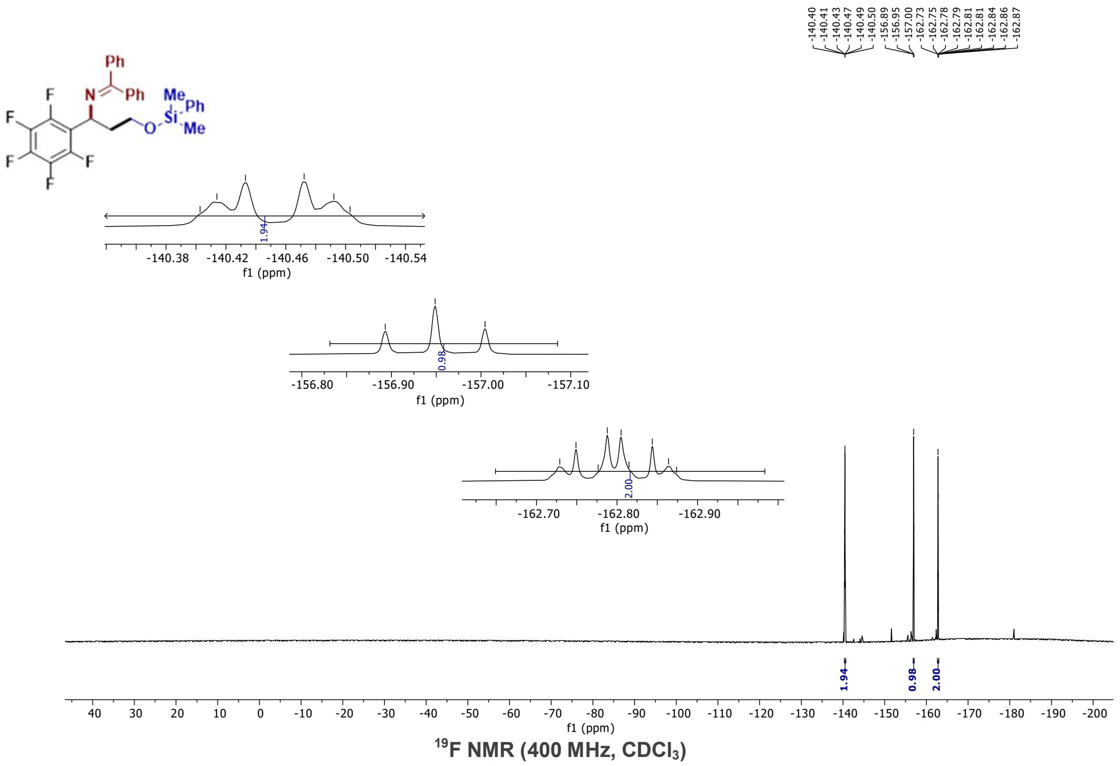

^{19}F NMR (400 MHz, CDCl$_3$)

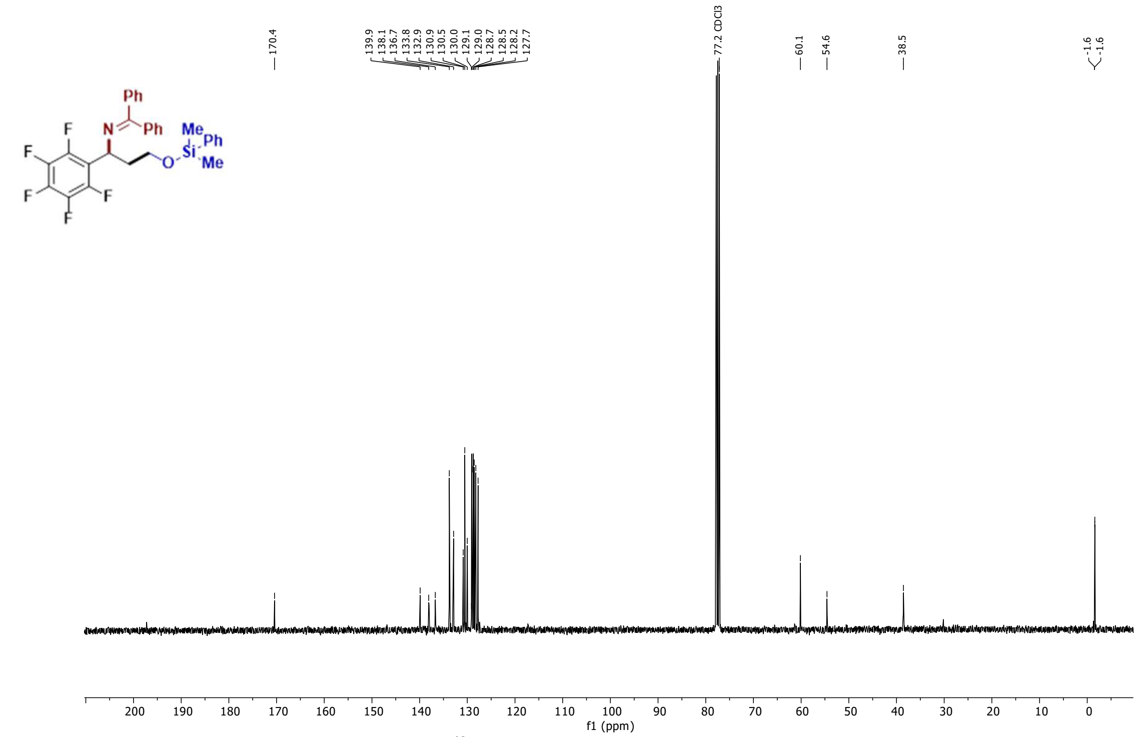

^{13}C NMR (101 MHz, $CDCl_3$)

3-((Dimethyl(phenyl)silyl)oxy)-1-((diphenylmethylene)amino)propyl propionate (3p)

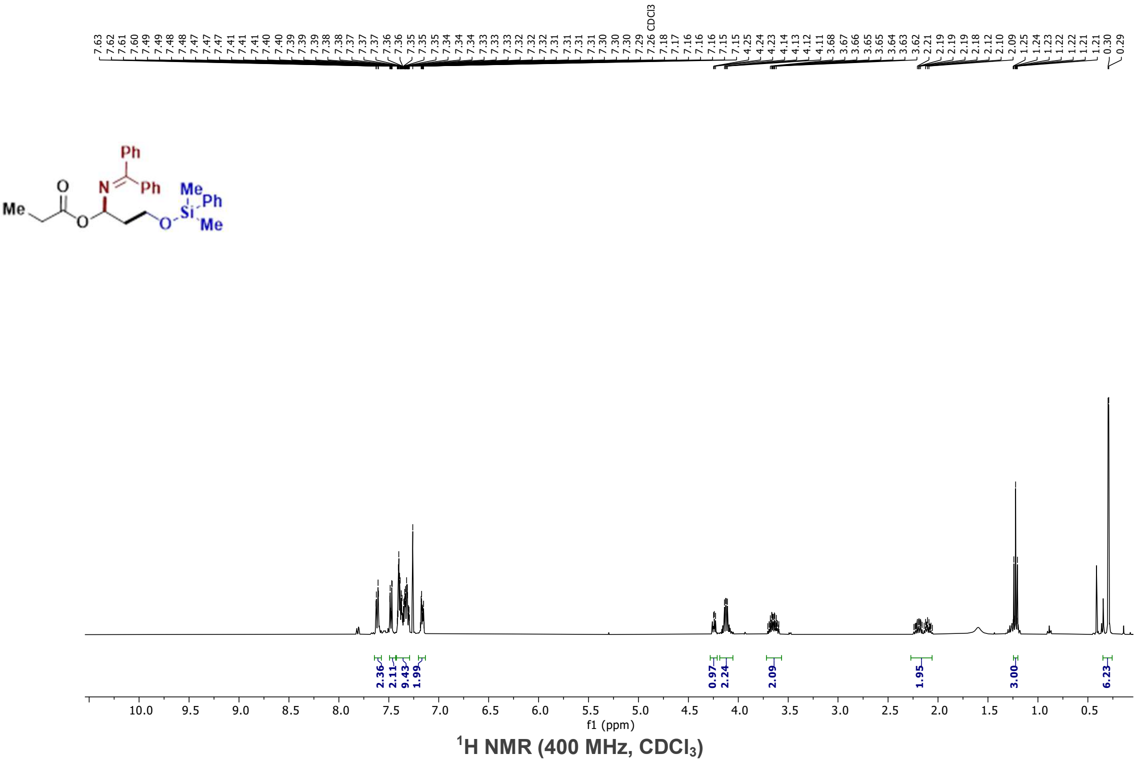

^{1}H NMR (400 MHz, $CDCl_3$)

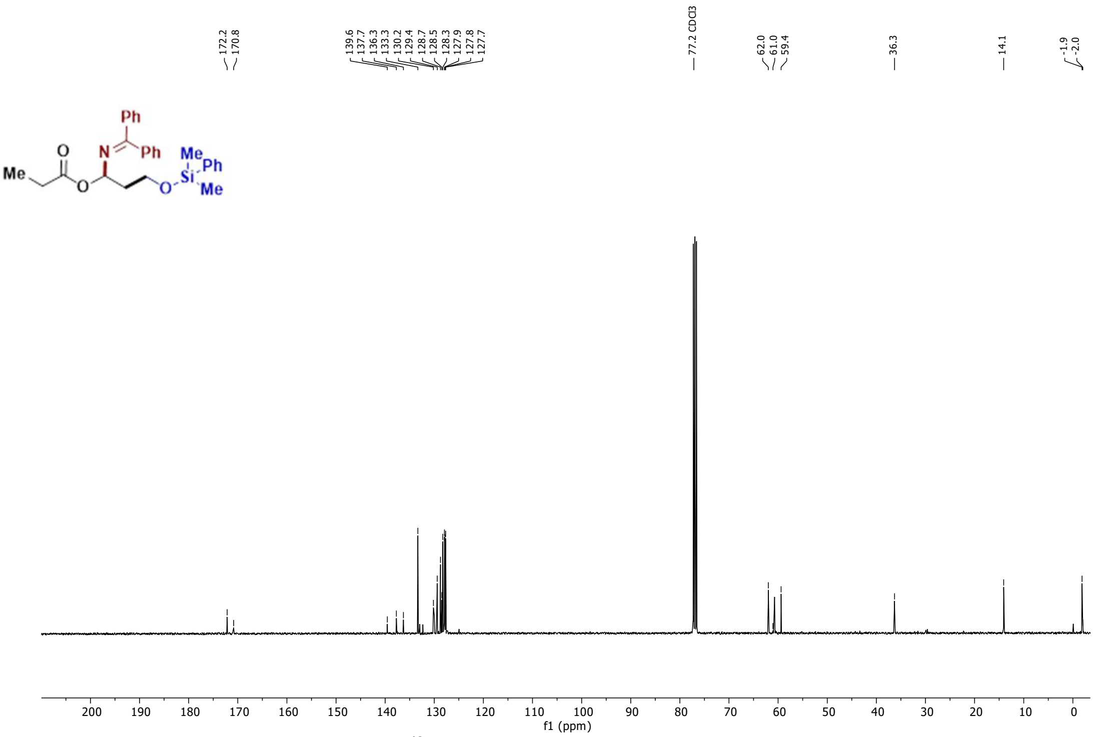

^{13}C NMR (101 MHz, $CDCl_3$)

Dimethyl-(3-((dimethyl(phenyl)silyl)oxy)-1-((diphenylmethylene)amino)propyl)phosphonate (3q)

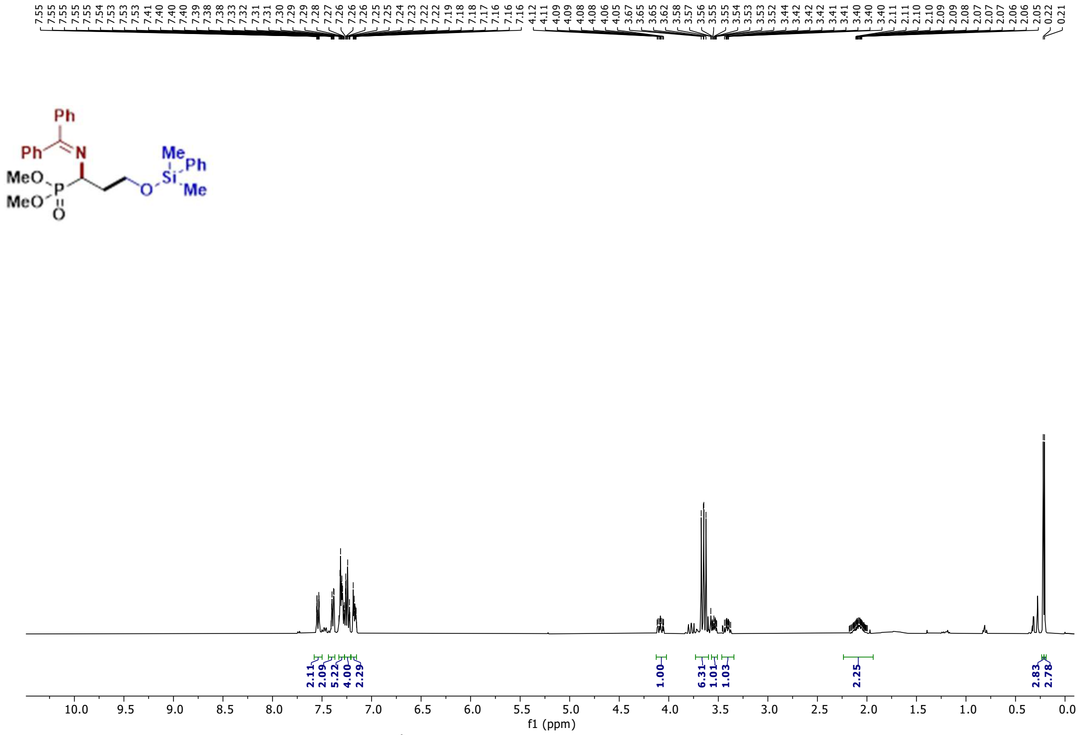

^{1}H NMR (400 MHz, $CDCl_3$)

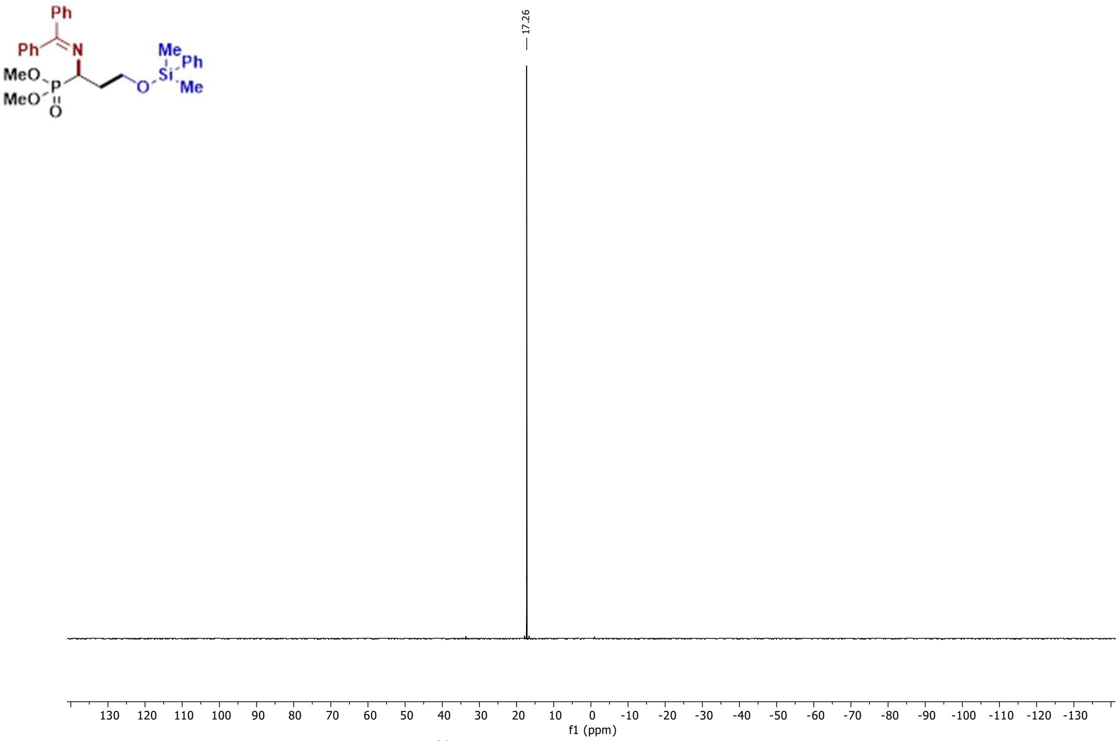

^{31}P NMR (162 MHz, $CDCl_3$)

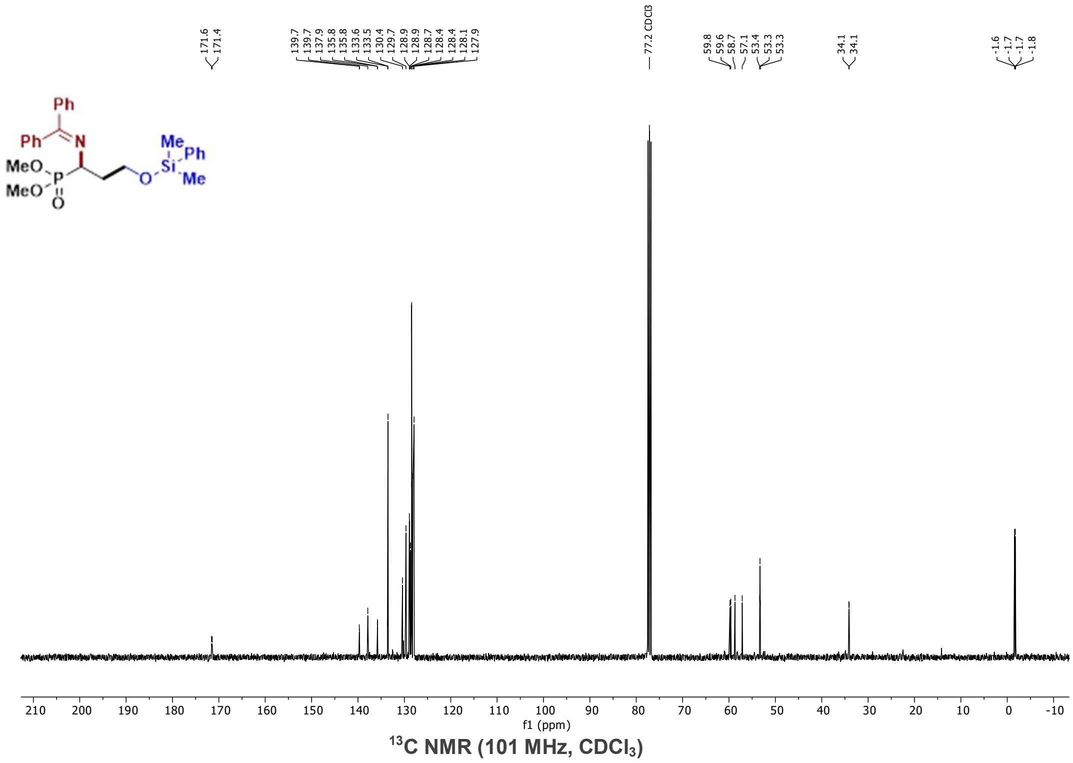

^{13}C NMR (101 MHz, $CDCl_3$)

Prop-2-yn-1-yl 4-((dimethyl(phenyl)silyl)oxy)-2-((diphenylmethylene)amino)butanoate (3r)

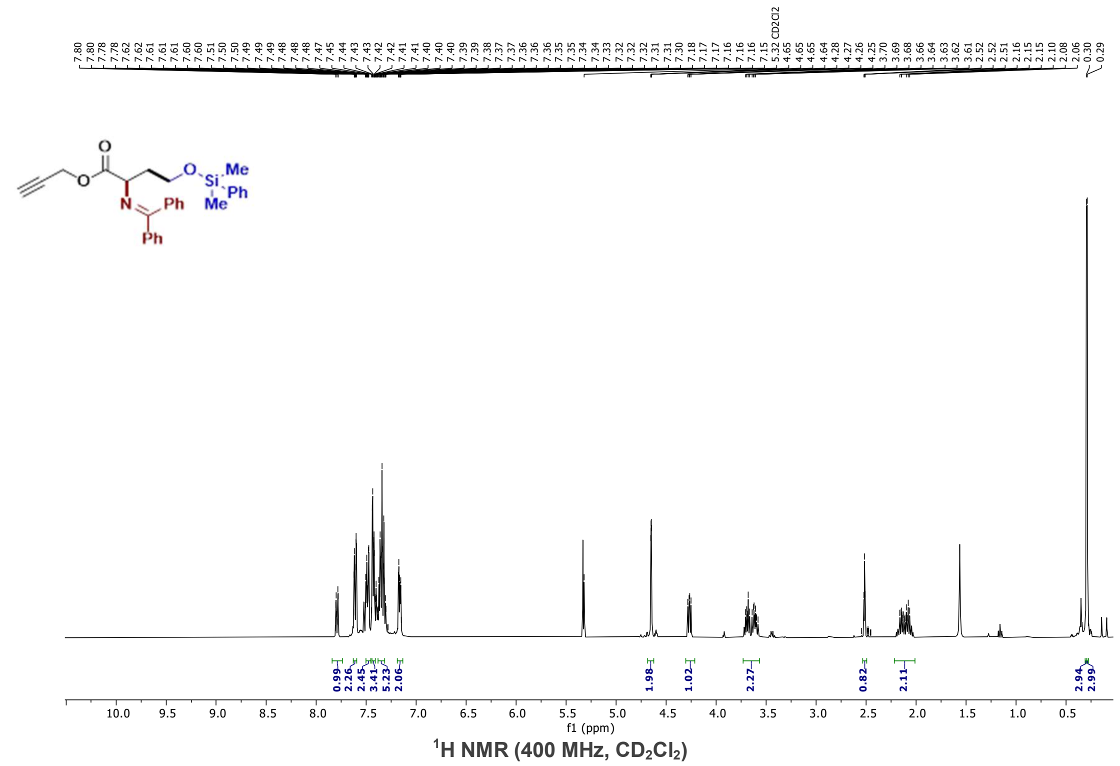

^{1}H NMR (400 MHz, CD_2Cl_2)

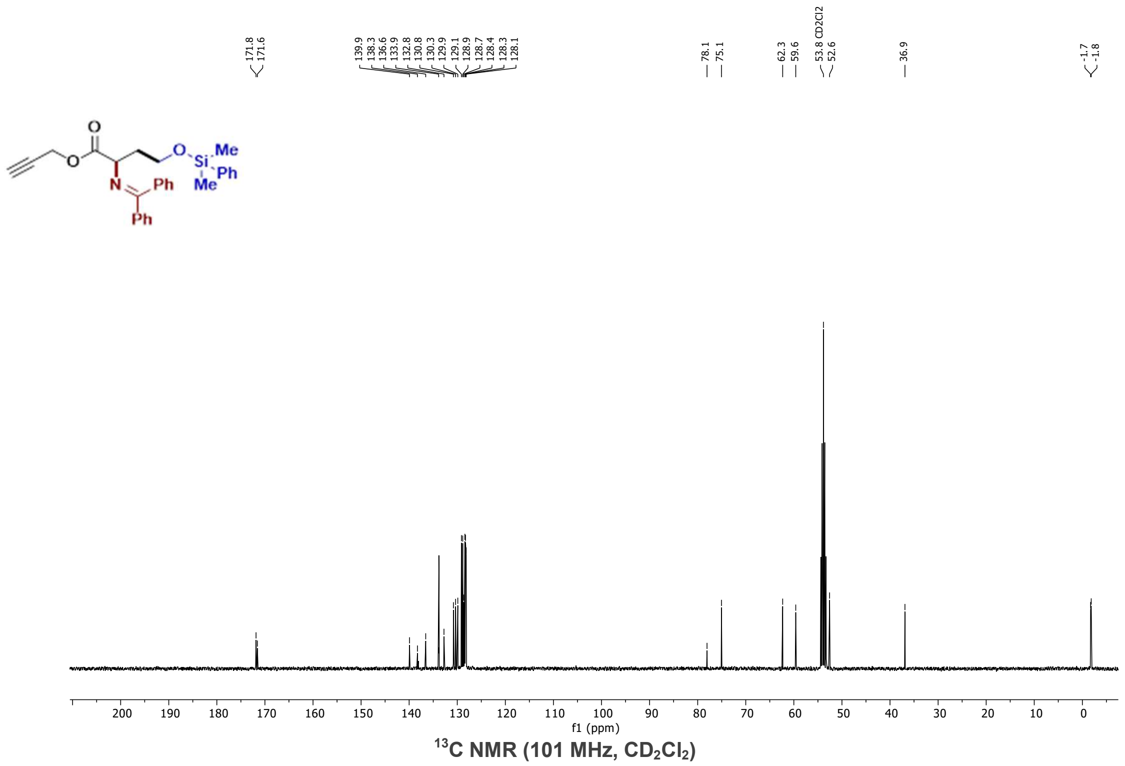

^{13}C NMR (101 MHz, CD_2Cl_2)

***N*-(3-((Dimethyl(phenyl)silyl)oxy)-1-(phenylsulfonyl)propyl)-1,1-diphenylmethanimine (3s)**

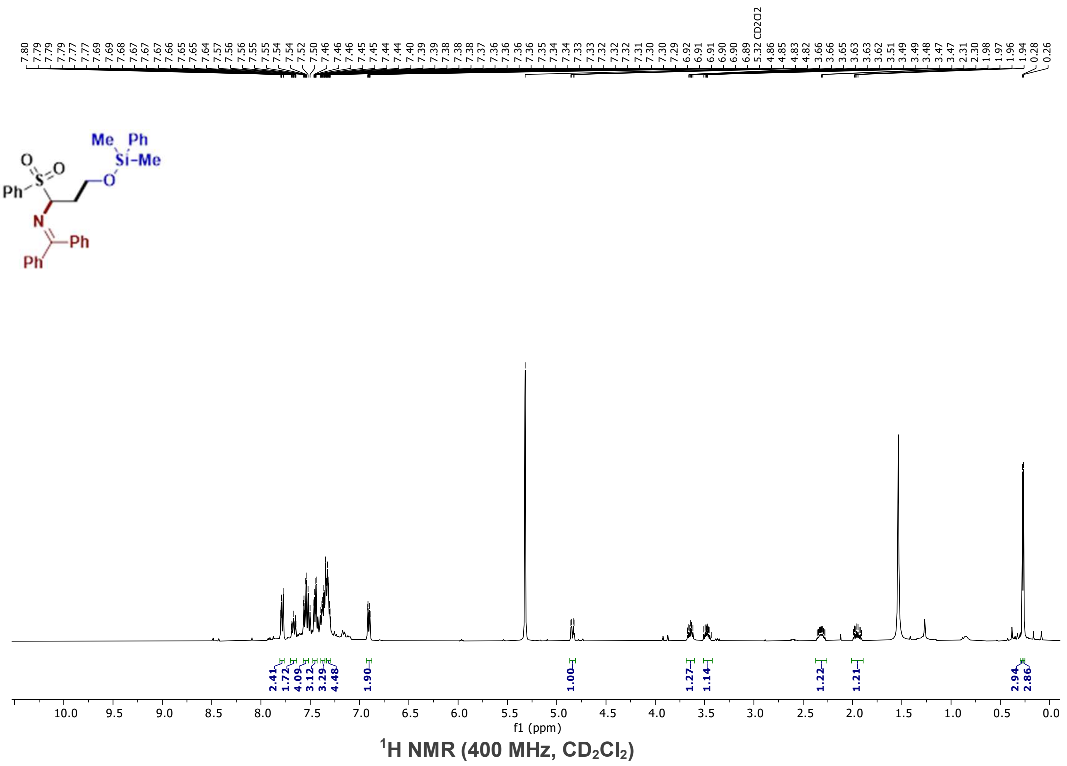

^{1}H NMR (400 MHz, CD_2Cl_2)

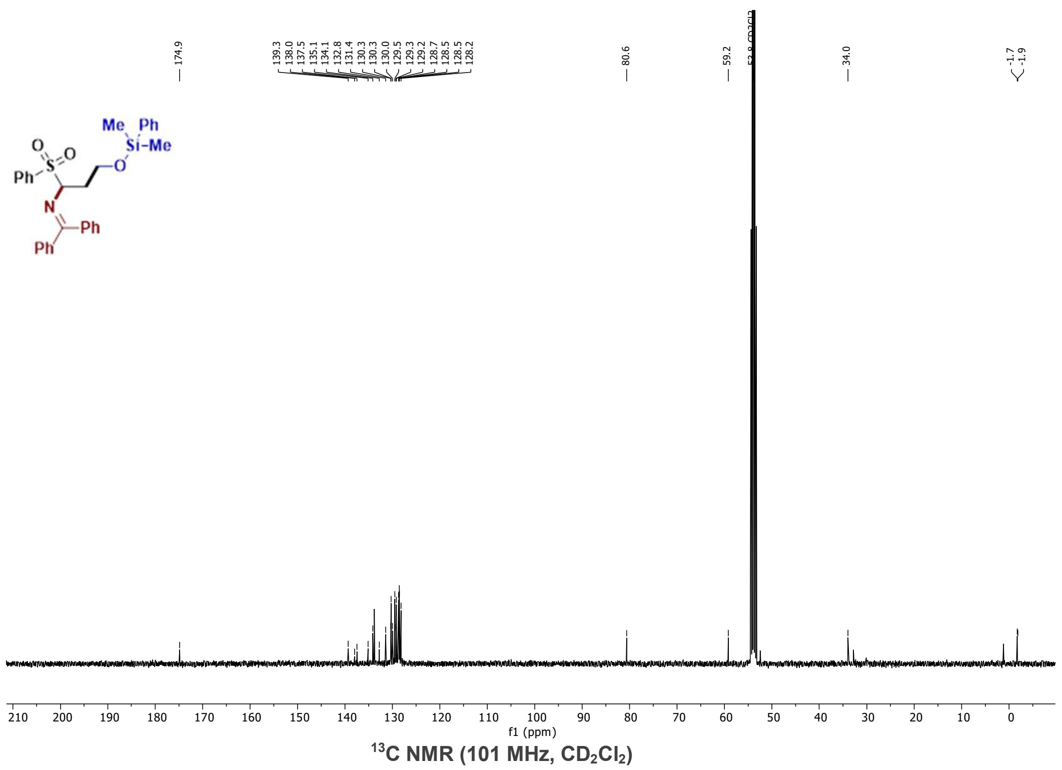

^{13}C NMR (101 MHz, CD_2Cl_2)

N-(3-((Dimethyl(phenyl)silyl)oxy)-1-(methylsulfonyl)propyl)-1,1-diphenylmethanimine (3t)

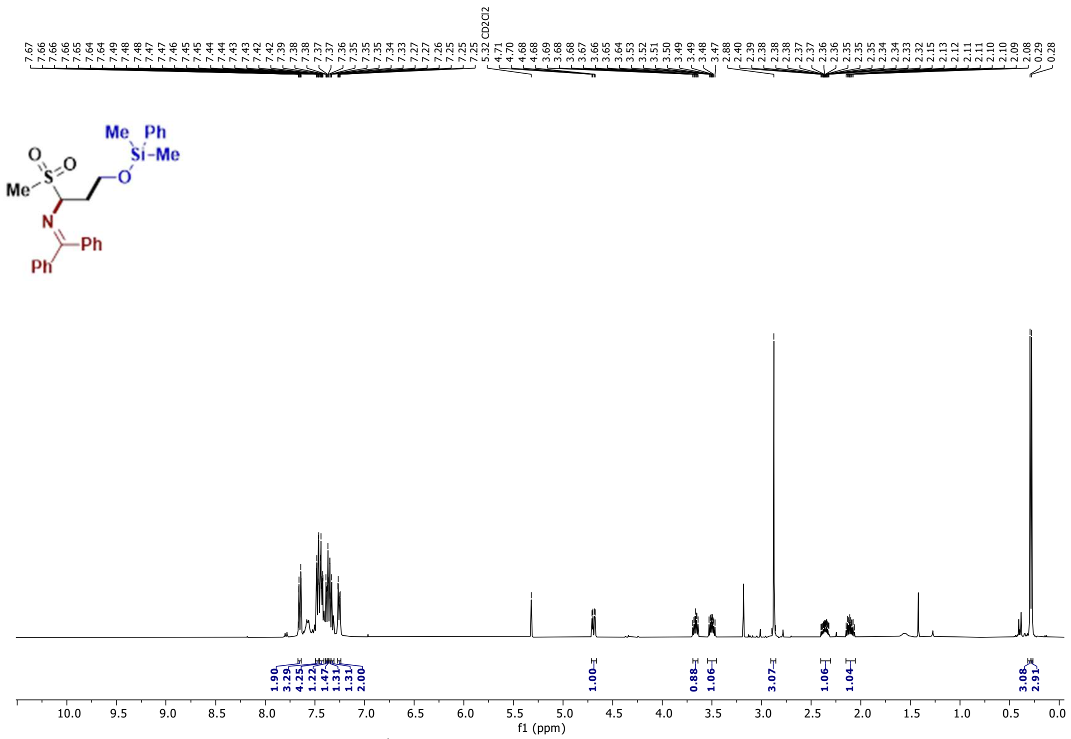

^{1}H NMR (400 MHz, CD_2Cl_2)

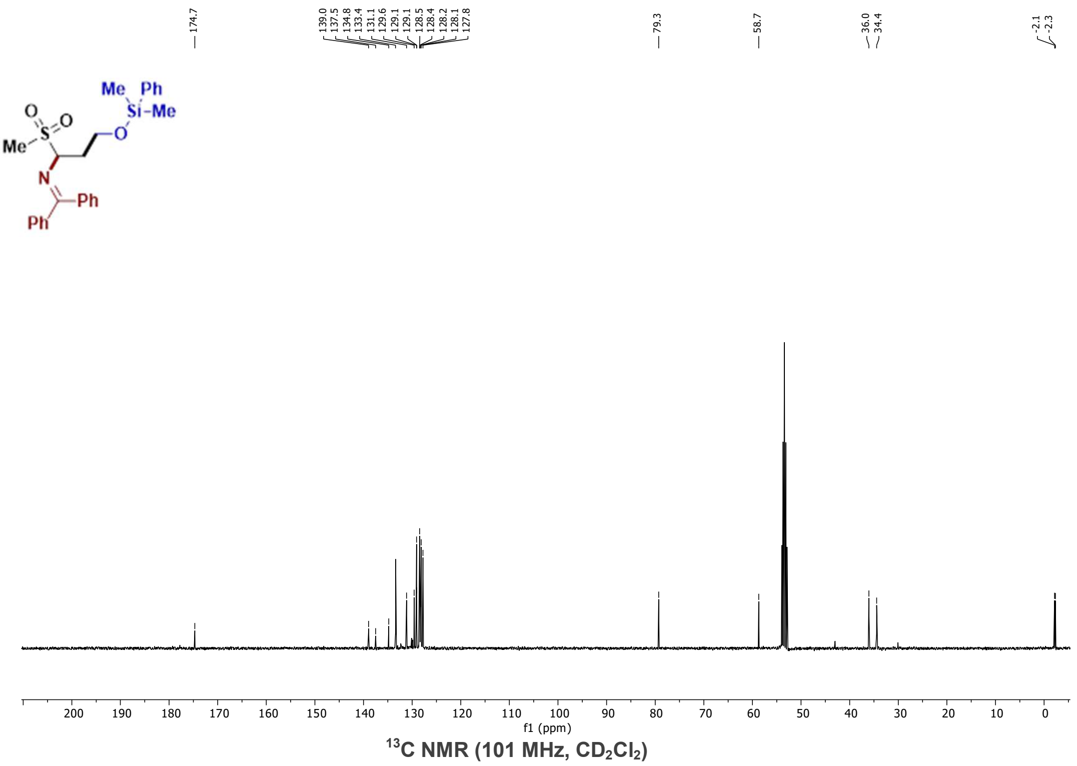

^{13}C NMR (101 MHz, CD_2Cl_2)

4-((Dimethyl(phenyl)silyl)oxy)-2-((diphenylmethylene)amino)-*N*,*N*-dimethylbutanamide (3u)

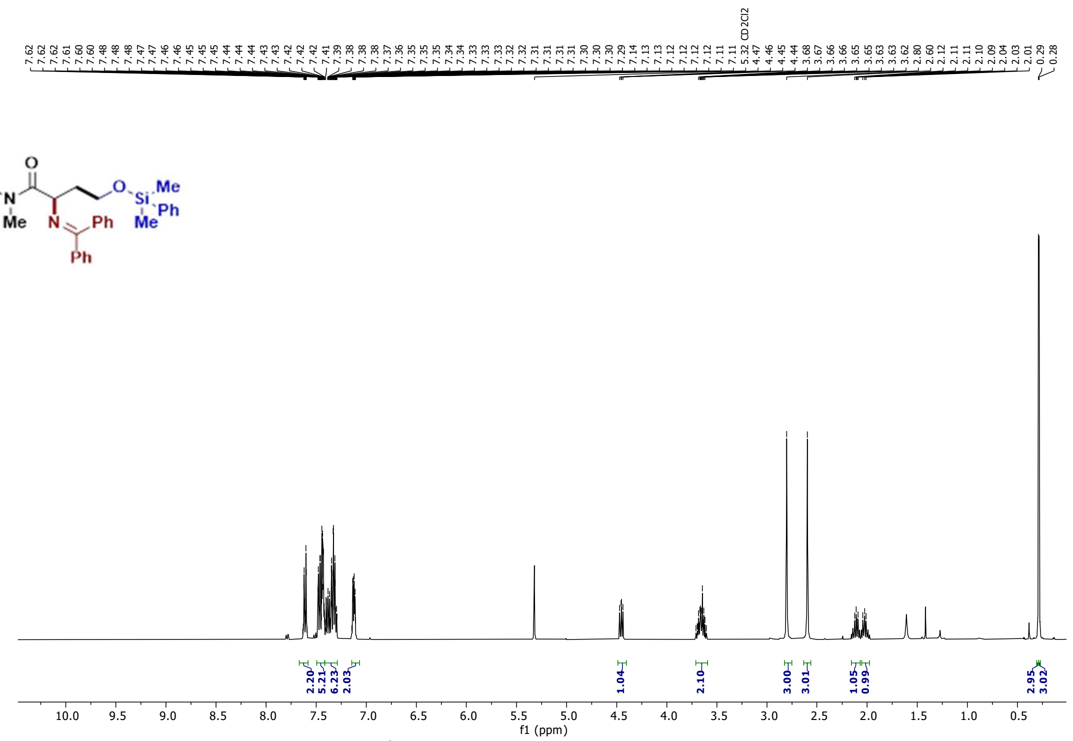

^1H NMR (400 MHz, CD_2Cl_2)

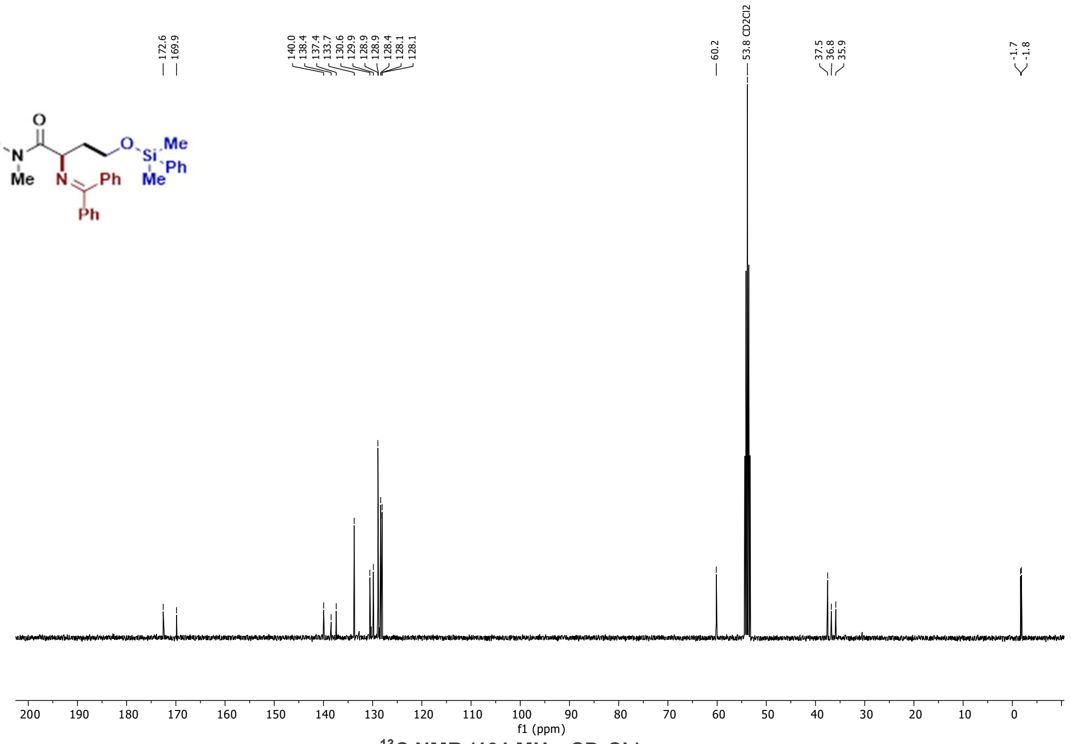

^{13}C NMR (101 MHz, CD_2Cl_2)

2,2,2-Trifluoroethyl -4-((dimethyl(phenyl)silyl)oxy)-2-((diphenylmethylene)amino)butanoate (3v)

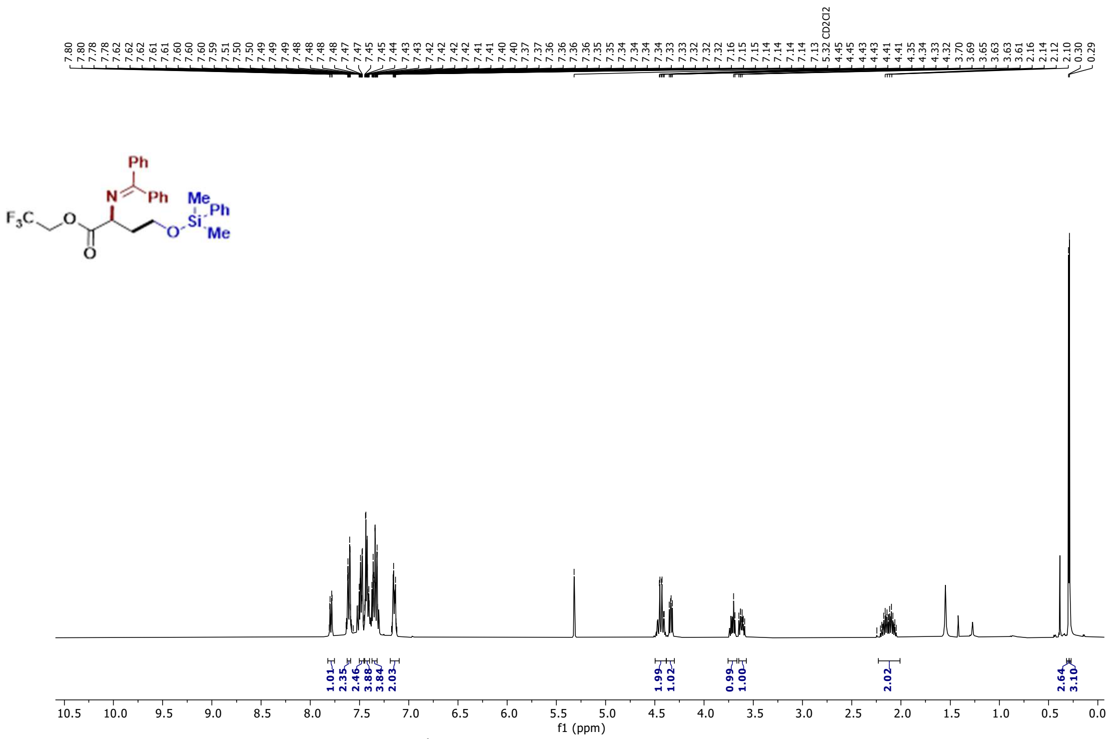

^{1}H NMR (400 MHz, CD_2Cl_2)

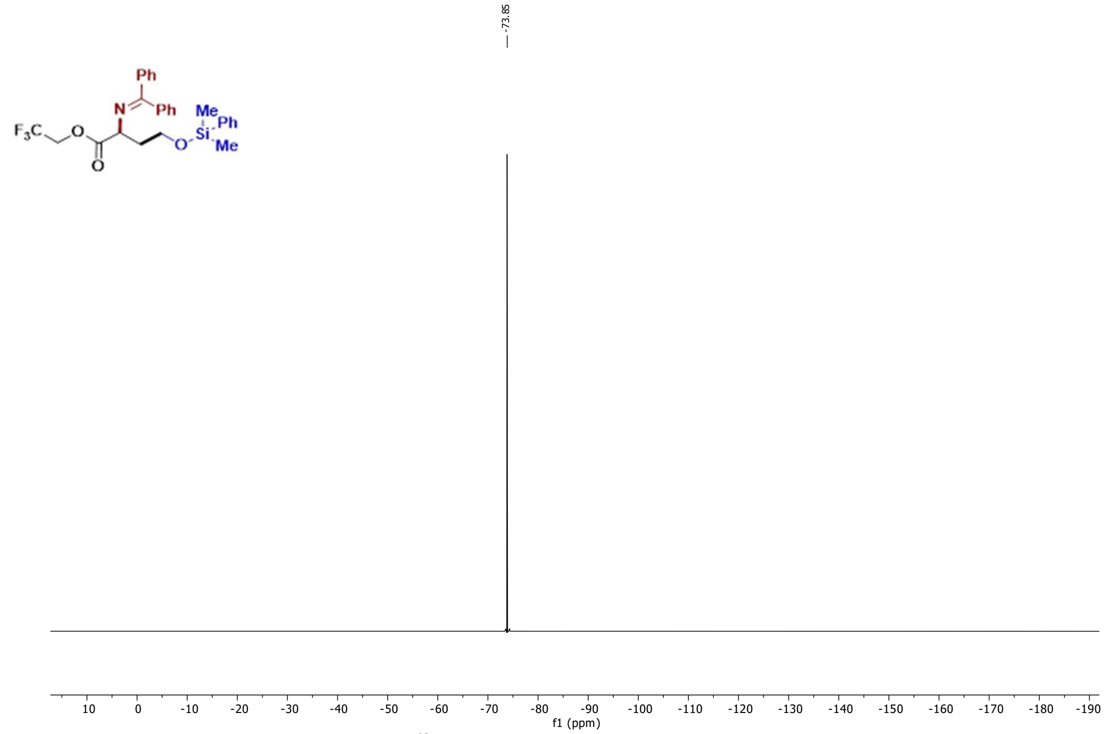

^{19}F NMR (400 MHz, CD_2Cl_2)

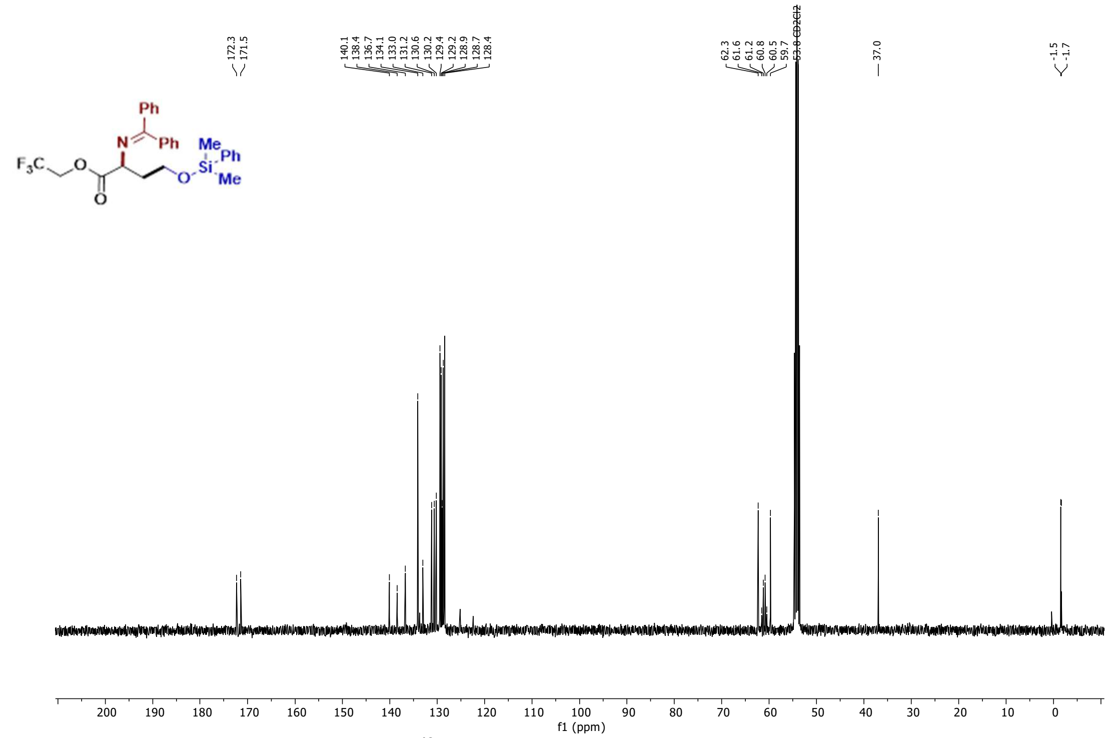

^{13}C NMR (101 MHz, CD_2Cl_2)

3-(((Dimethyl(phenyl)silyl)oxy)methyl)-2-((diphenylmethylene)amino)cyclohexan-1-one (3w)

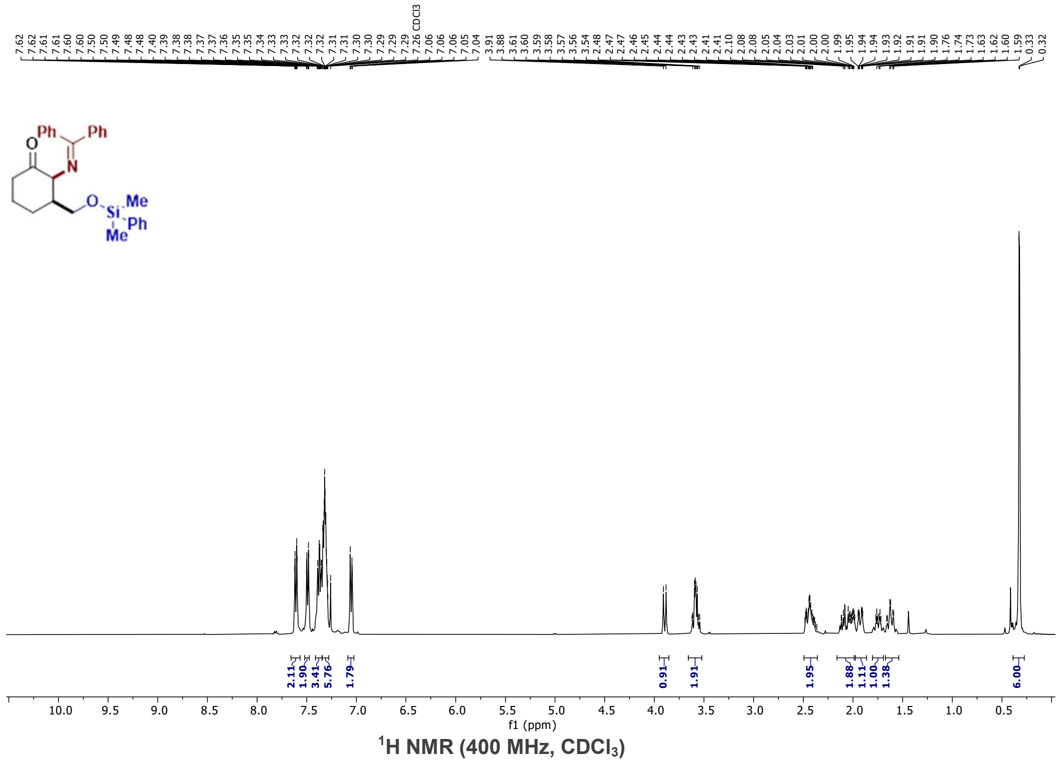

^{1}H NMR (400 MHz, $CDCl_3$)

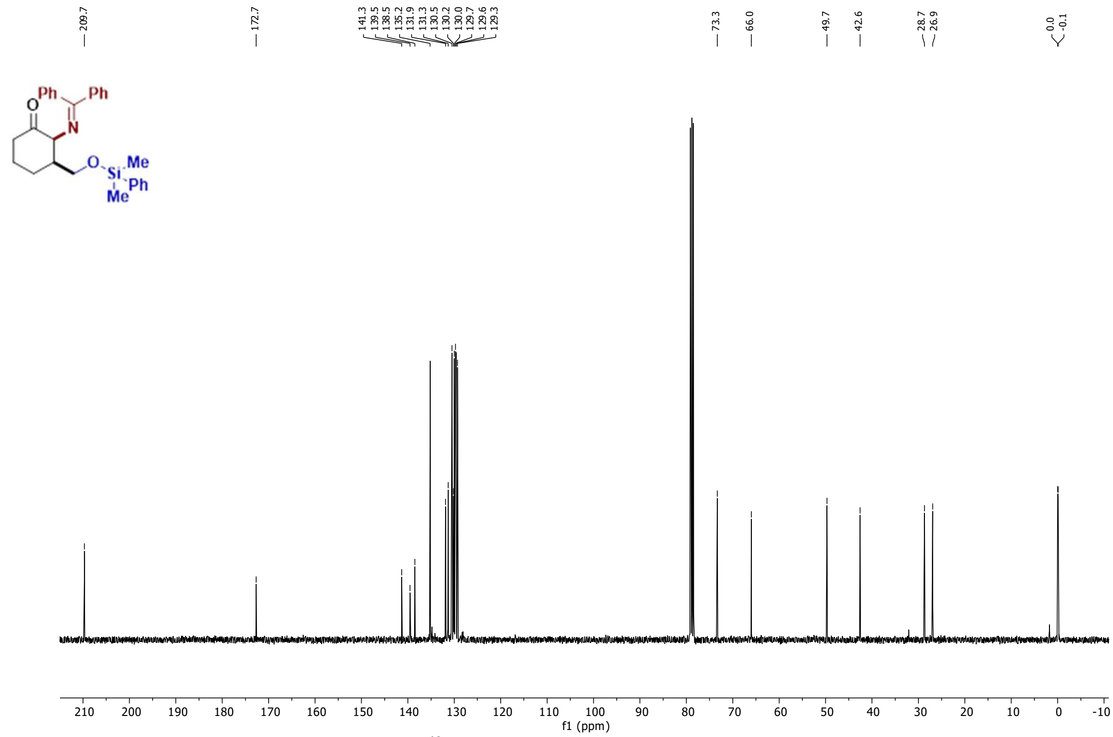

^{13}C NMR (101 MHz, $CDCl_3$)

3-(((Dimethyl(phenyl)silyl)oxy)methyl)-4-((diphenylmethylene)amino)-1-phenylpyrrolidine-2,5-dione (3x)

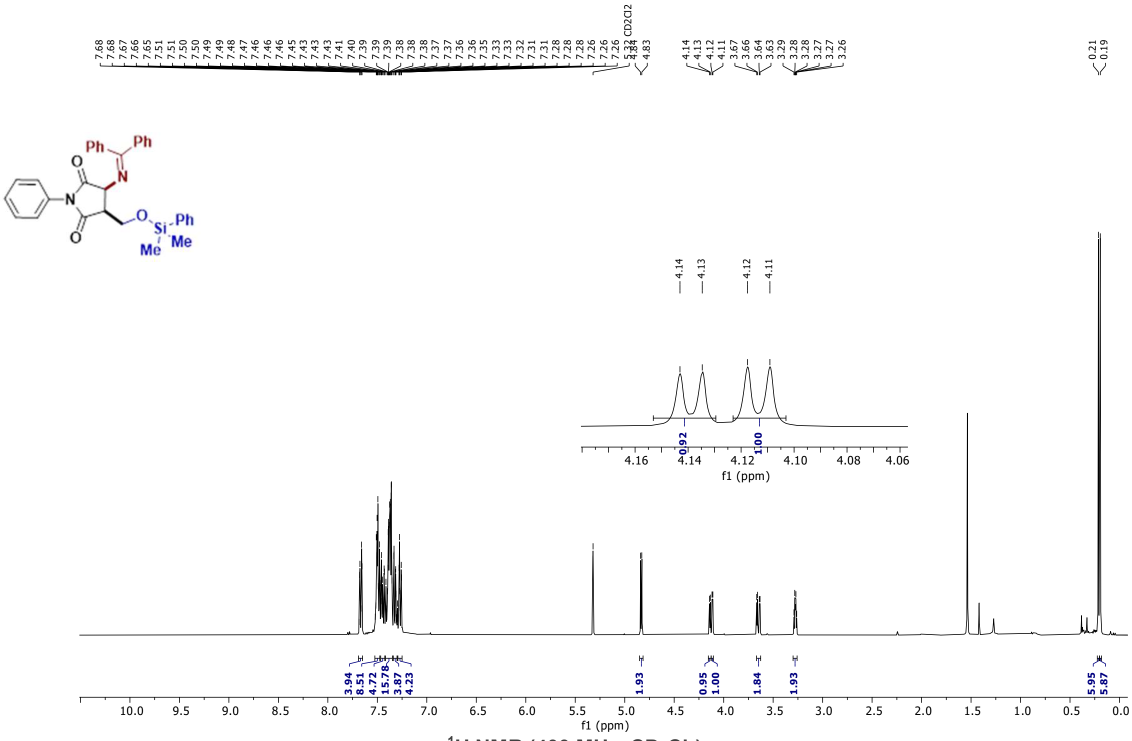

^{1}H NMR (400 MHz, CD_2Cl_2)

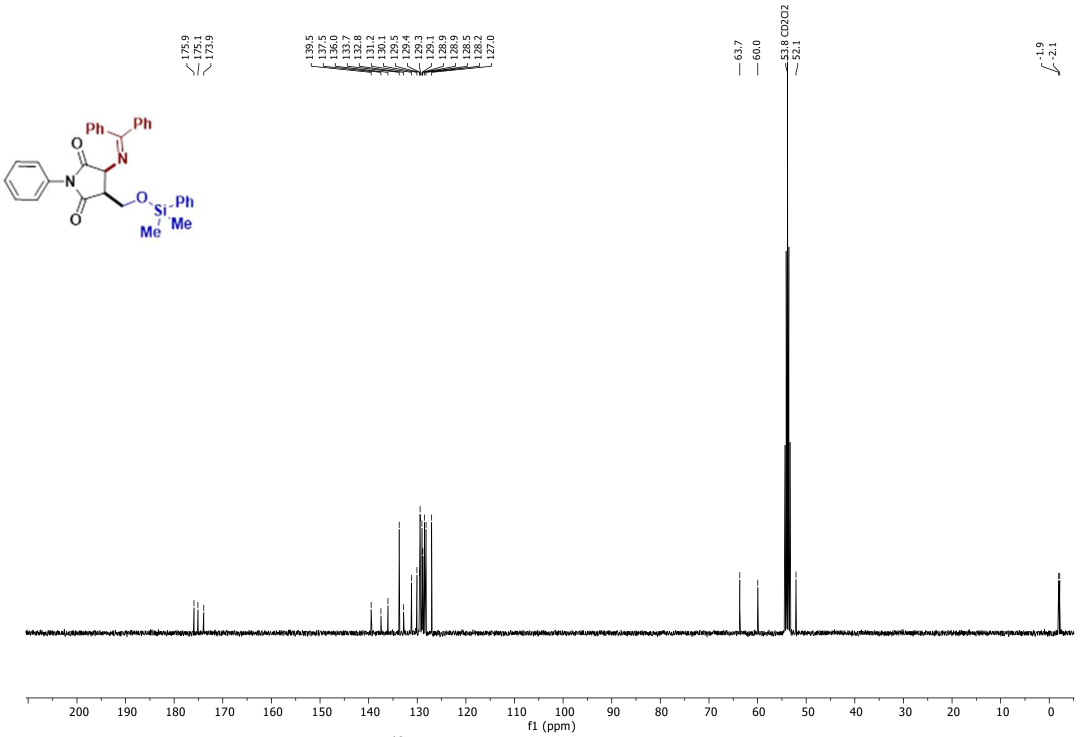

^{13}C NMR (101 MHz, CD_2Cl_2)

2-(((Dimethyl(phenyl)silyl)oxy)methyl)-3-((diphenylmethylene)amino)succinonitrile (3y)

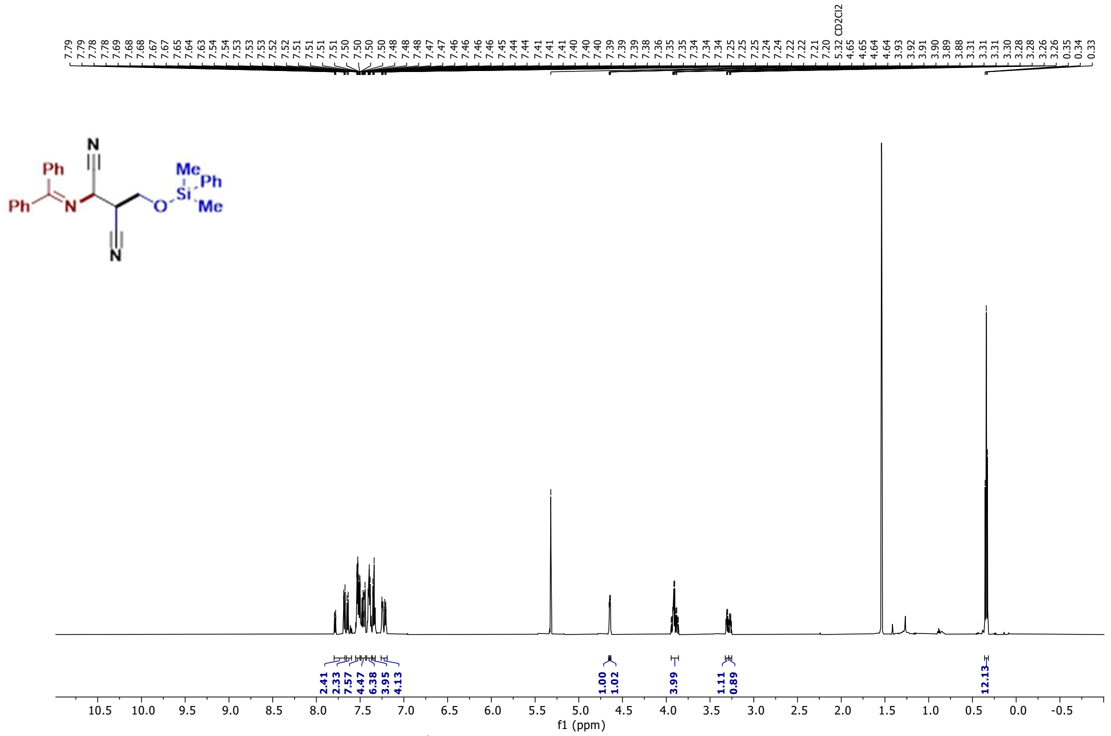

^{1}H NMR (599 MHz, CD_2Cl_2)

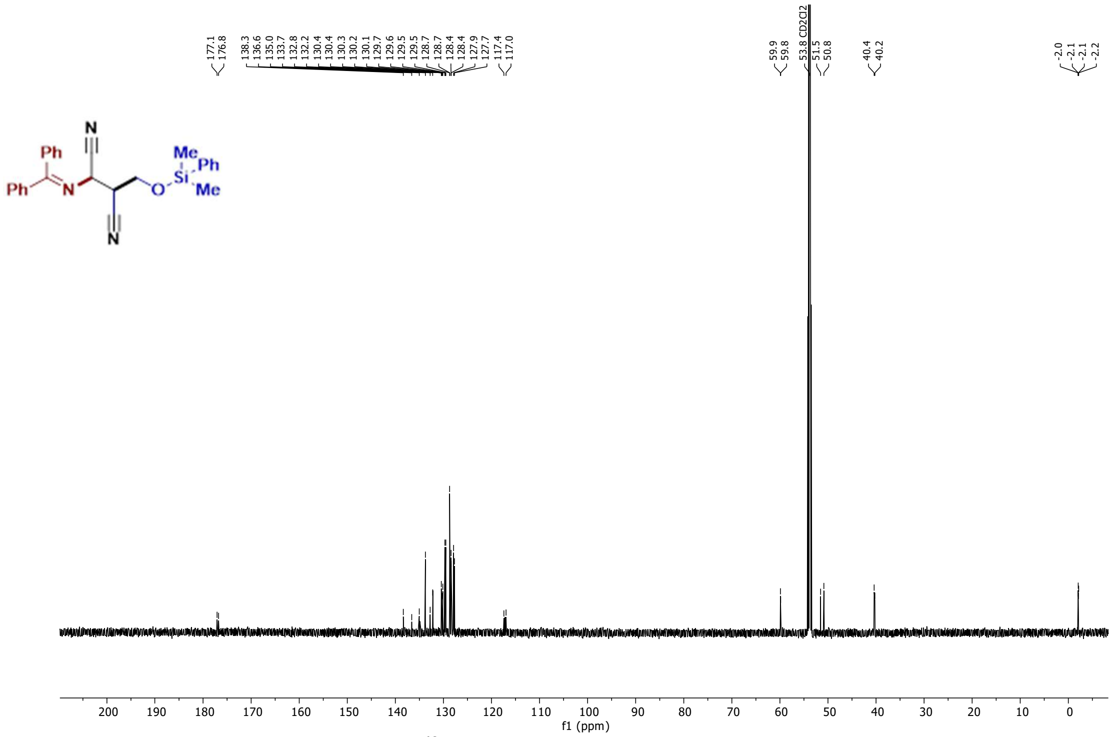

^{13}C NMR (151 MHz, CD_2Cl_2)

3-Amino-3-(2-hydroxyethyl)dihydrofuran-2(3*H*)-one hydrochloride (3z)

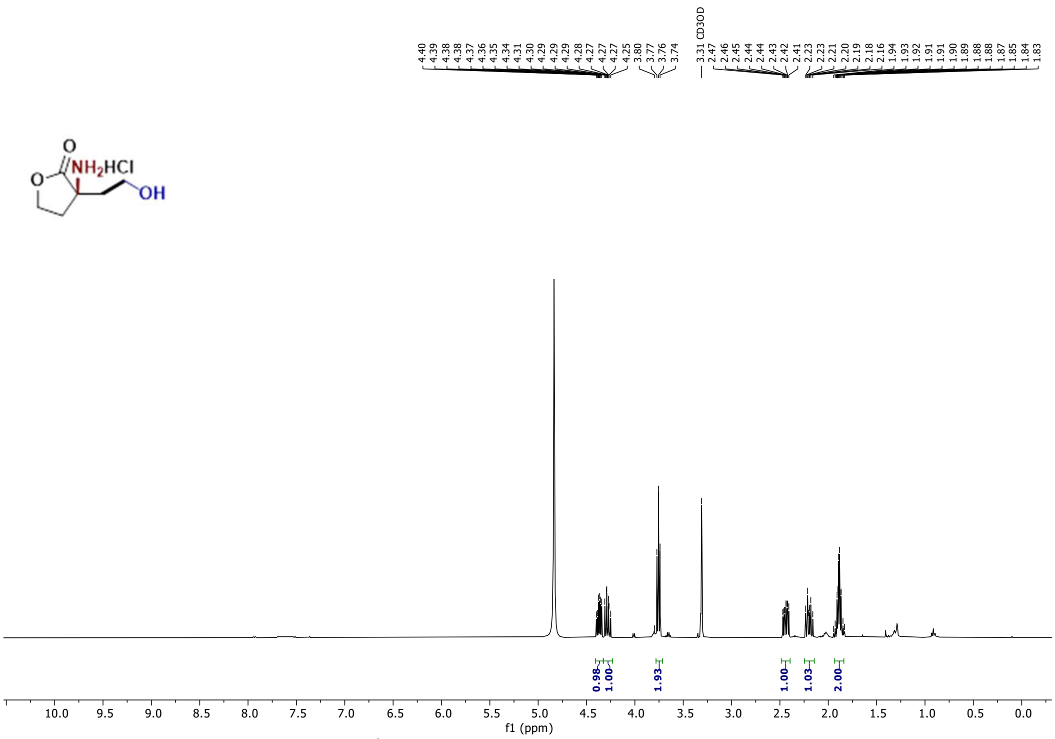

^{1}H NMR (400 MHz, MeOD)

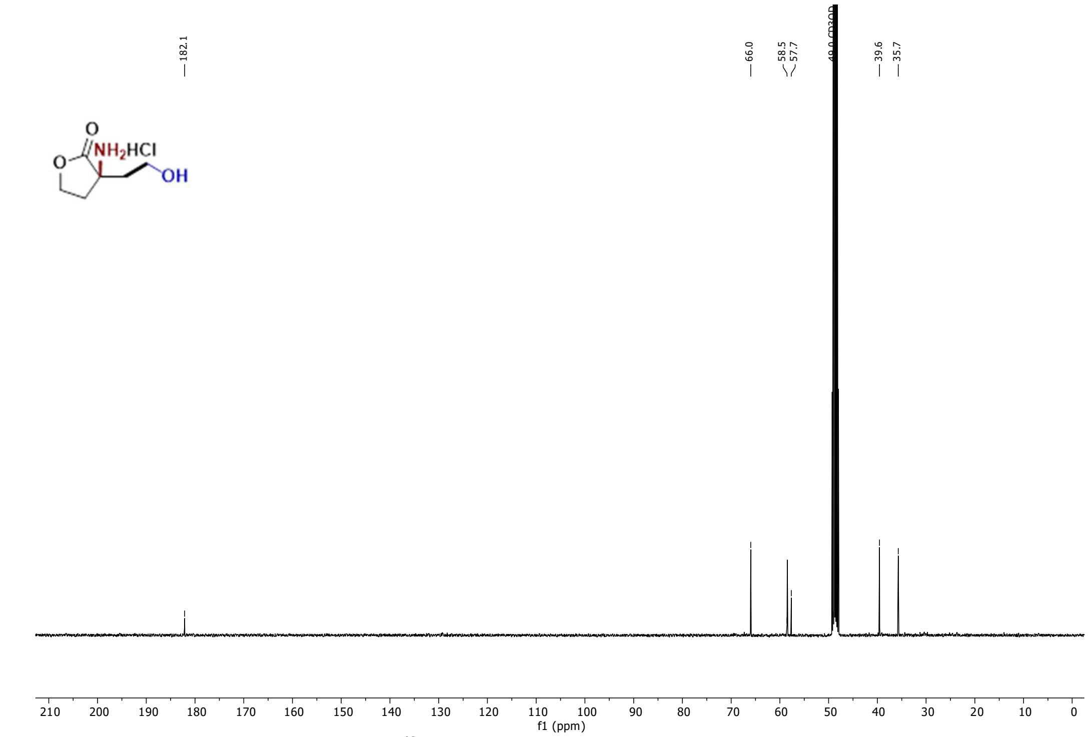

^{13}C NMR (101 MHz, MeOD)

4-((Dimethyl(phenyl)silyl)oxy)-2-((diphenylmethylene)amino)-2-methylbutanenitrile (3aa)

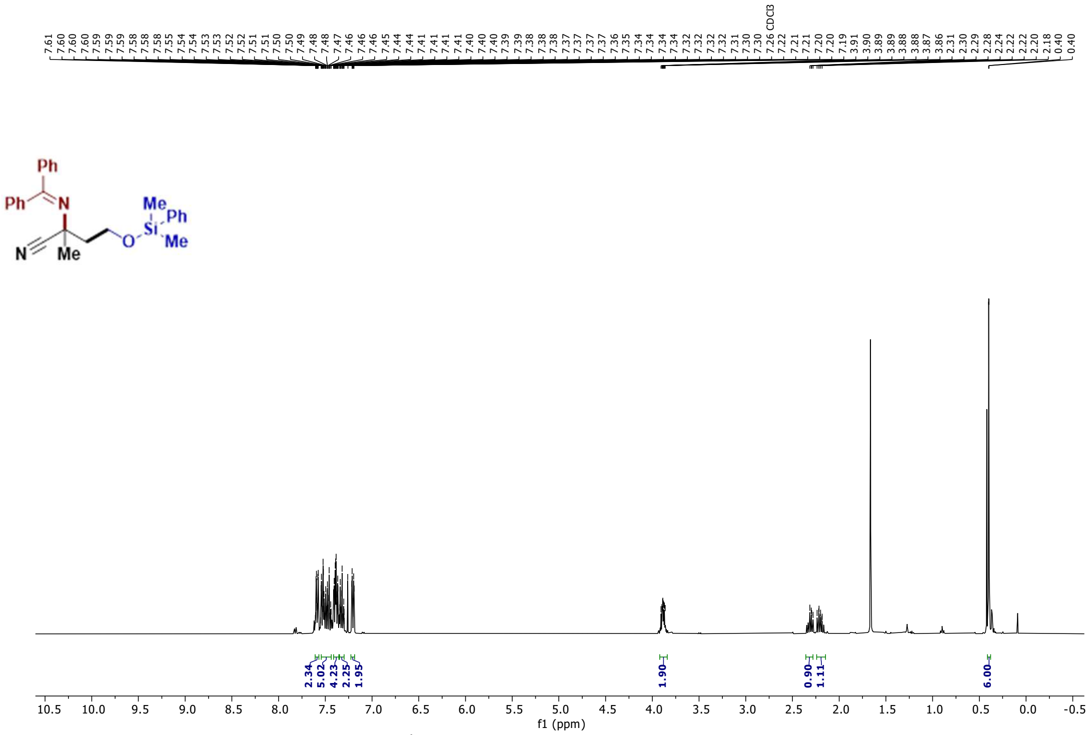

^{1}H NMR (400 MHz, $CDCl_3$)

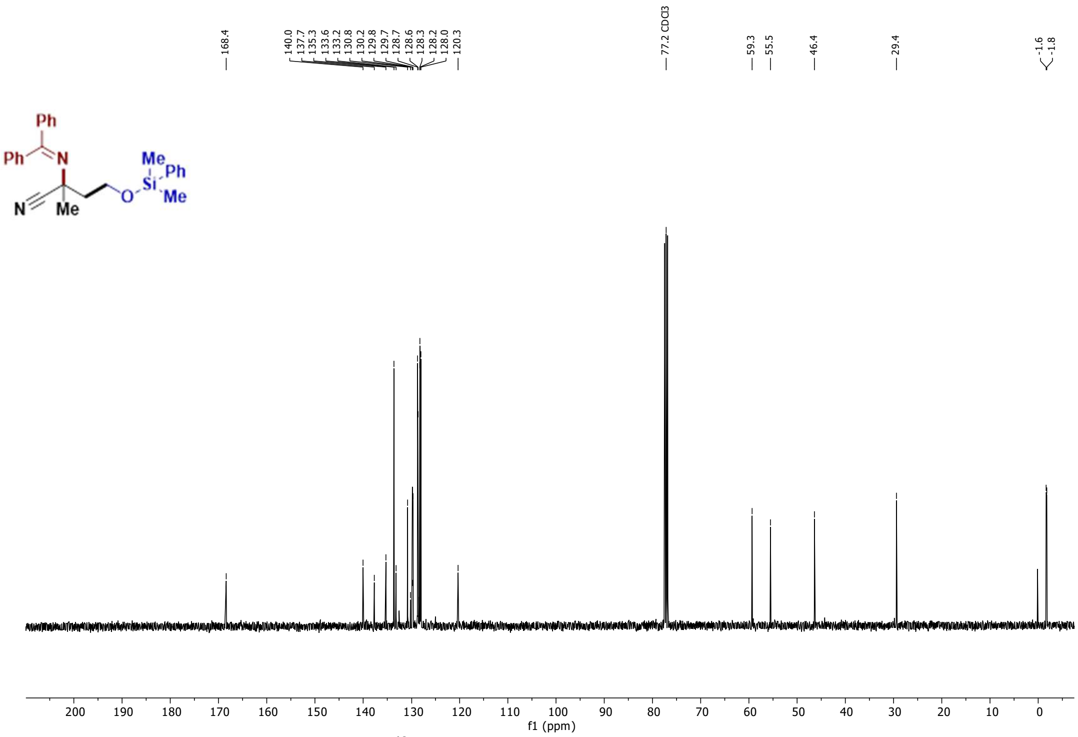

^{13}C NMR (101 MHz, $CDCl_3$)

***N*-(4-((Dimethyl(phenyl)silyl)oxy)-1,1,1-trifluoro-2-(naphthalen-1-yl)butan-2-yl)-1,1-diphenylmethanimine (3ab)**

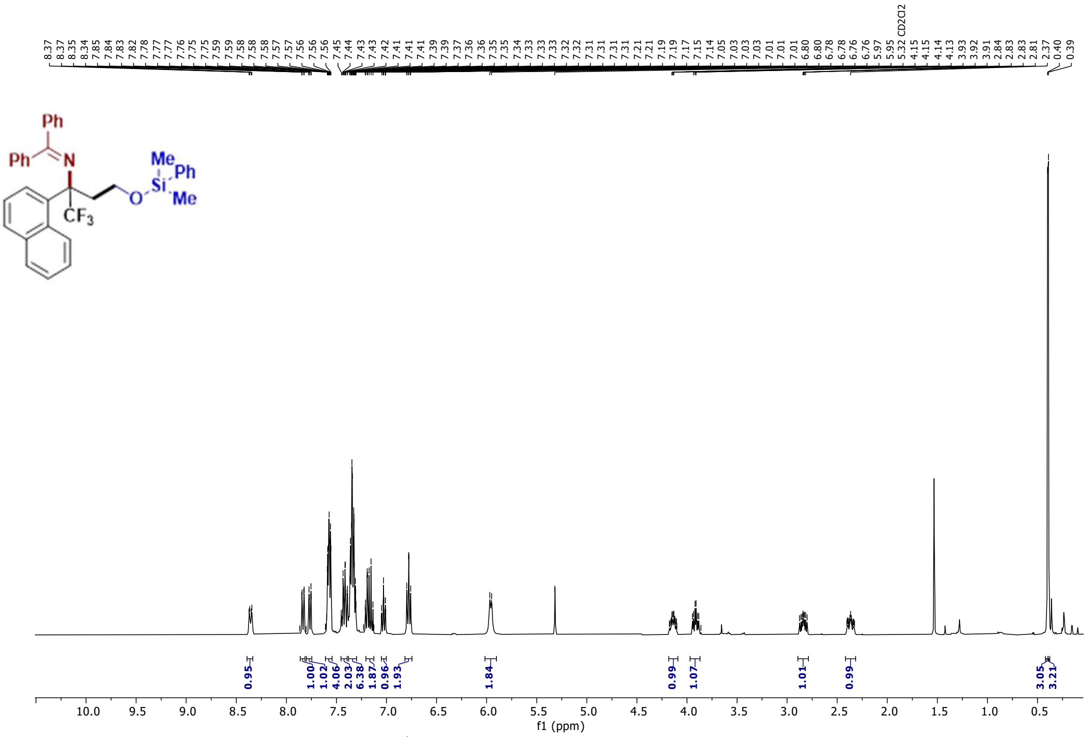

^{1}H NMR (400 MHz, CD_2Cl_2)

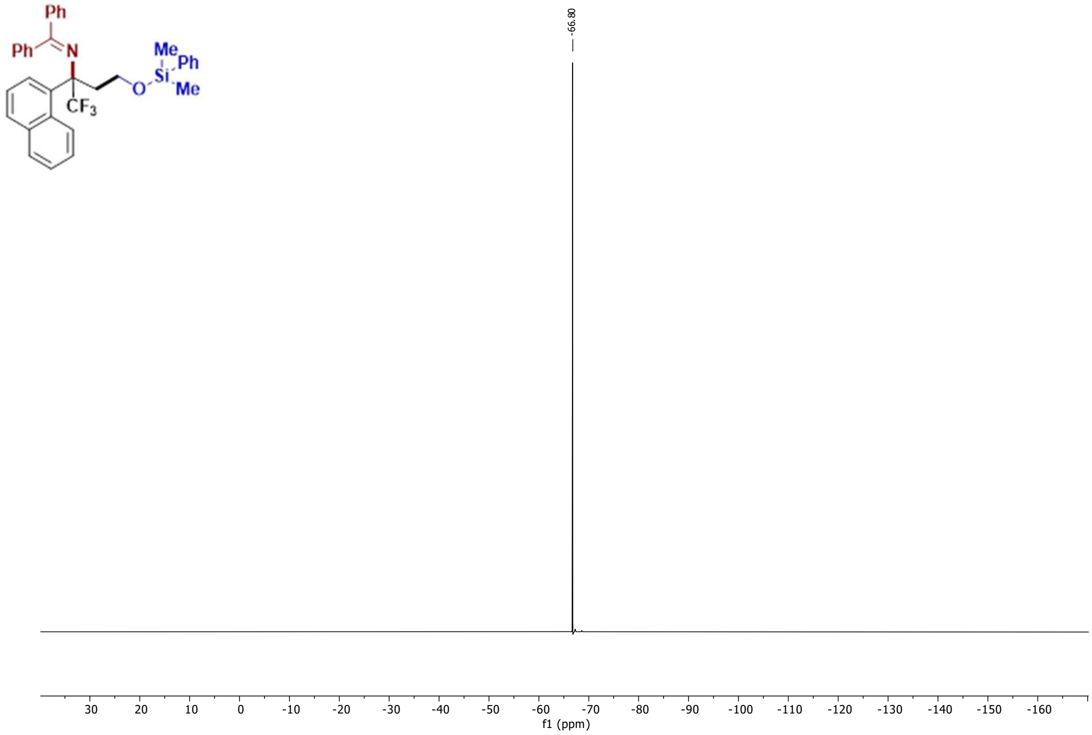

^{19}F NMR (376 MHz, CD_2Cl_2)

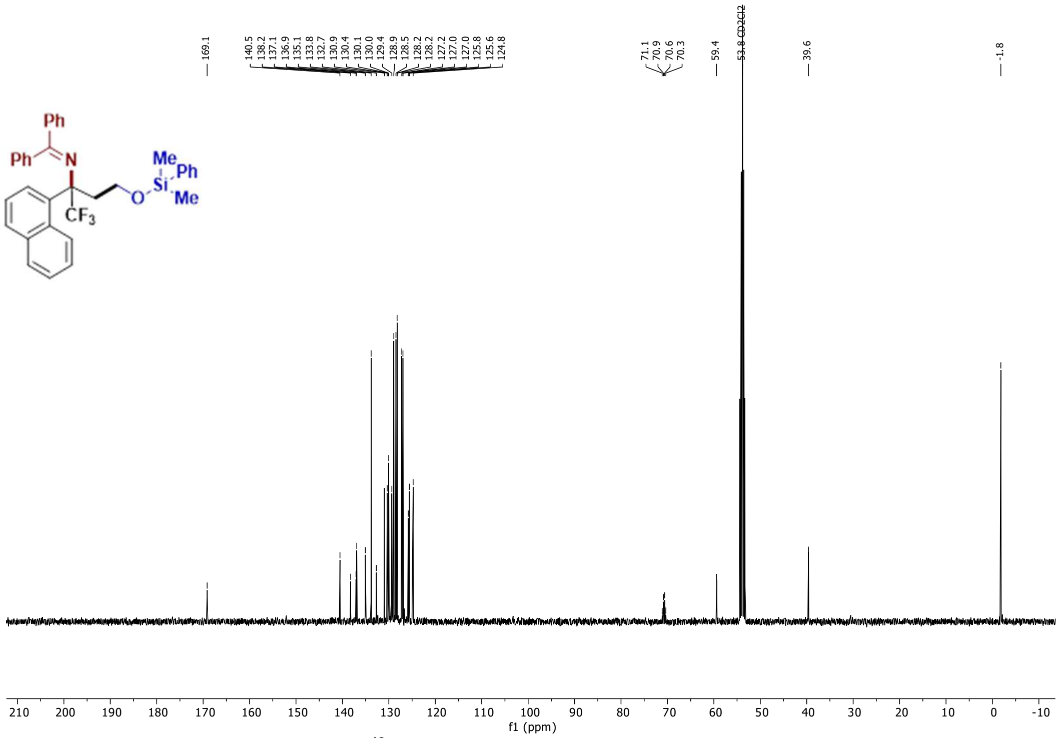

^{13}C NMR (101 MHz, CD_2Cl_2)

Methyl 4-(4-((dimethyl(phenyl)silyl)oxy)-2-((diphenylmethylene)amino)butan-2-yl)benzoate (3ac)

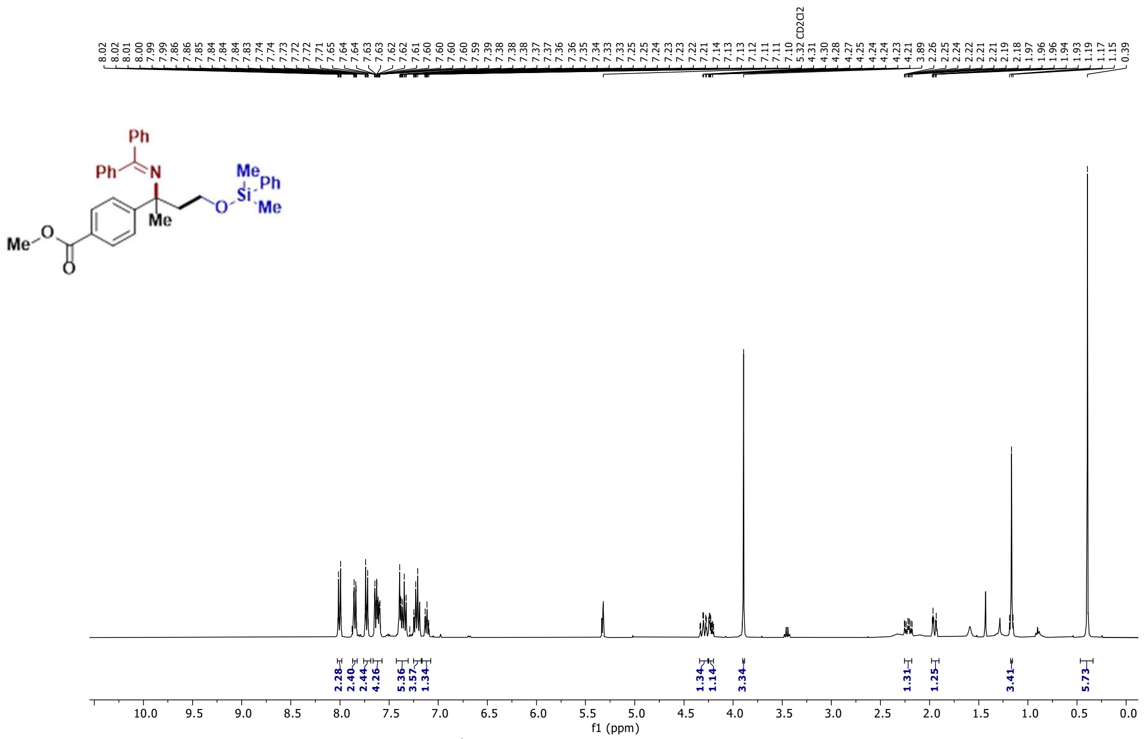

^{1}H NMR (400 MHz, CD_2Cl_2)

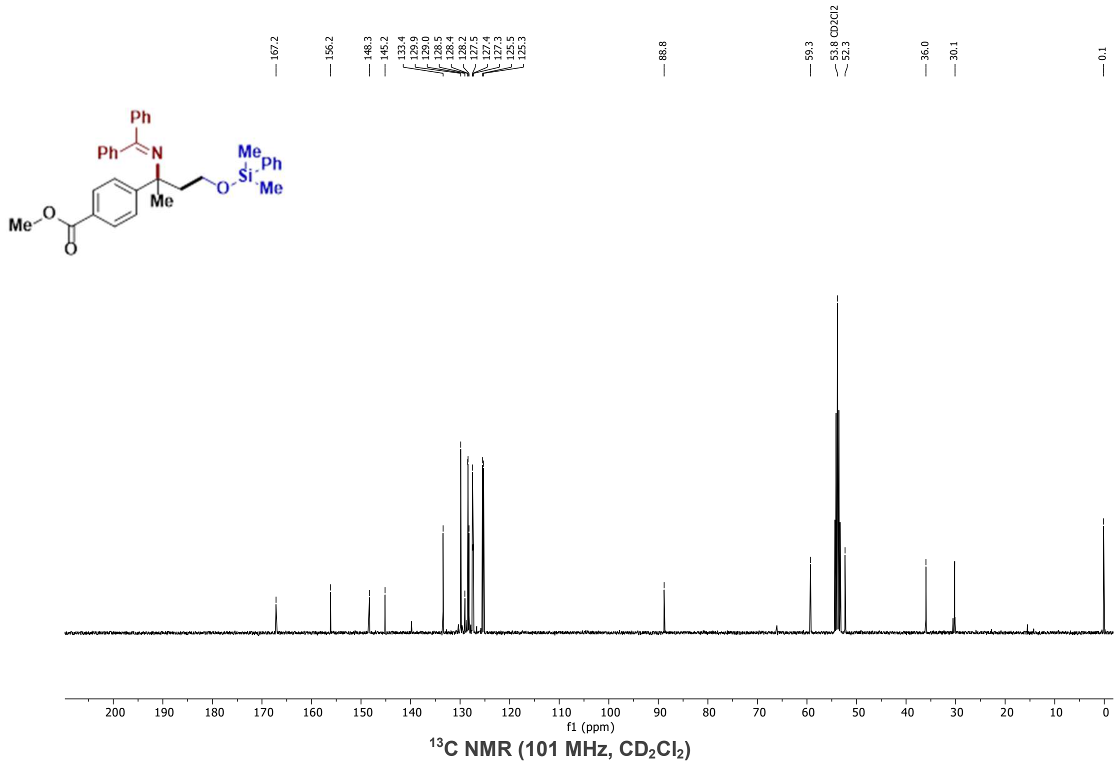

^{13}C NMR (101 MHz, CD_2Cl_2)

3-Amino-3,3-diphenylpropan-1-ol hydrochloride (3ad)

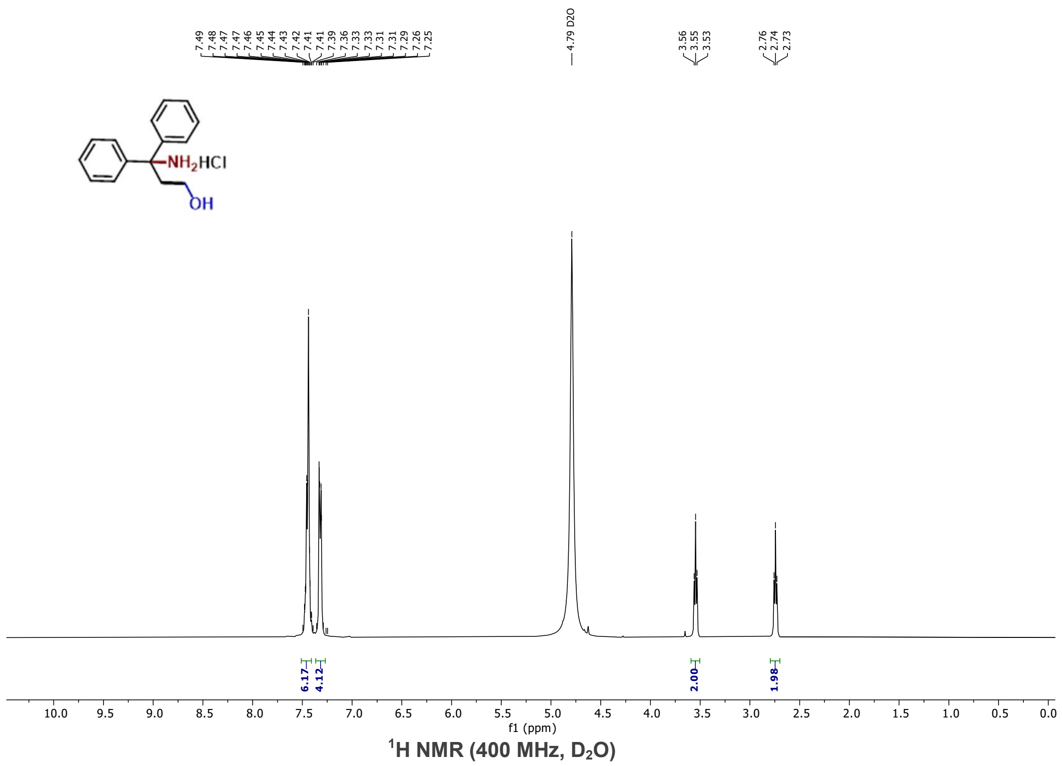

^1H NMR (400 MHz, D_2O)

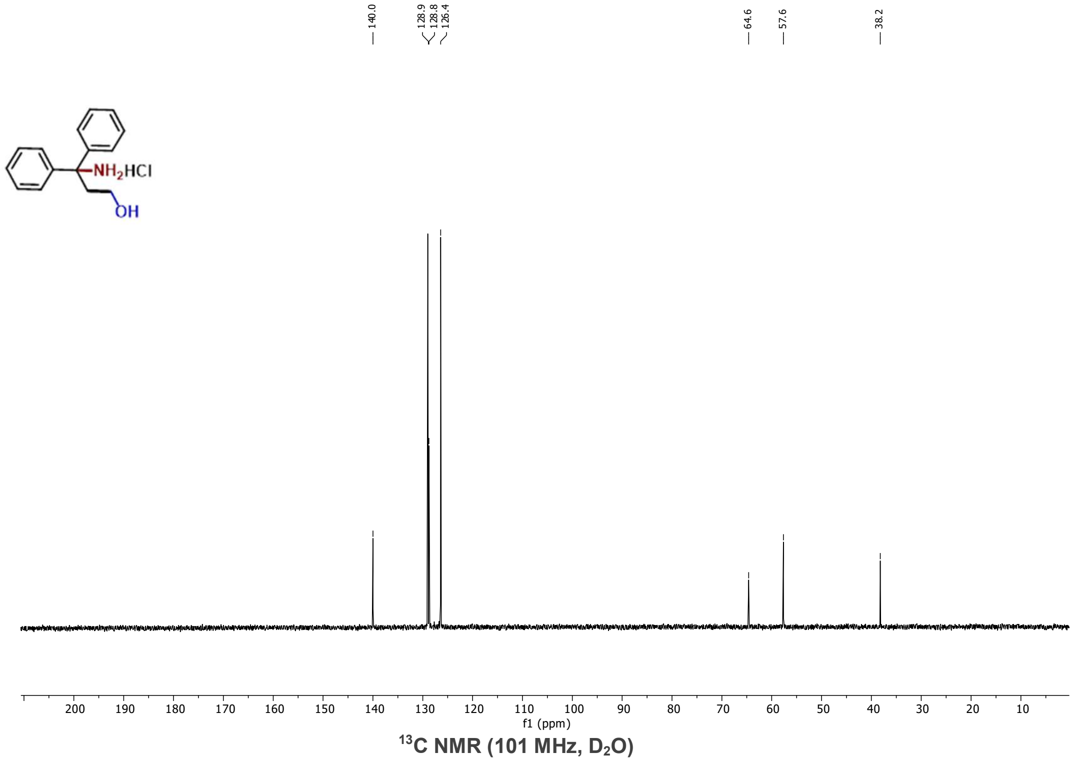

^{13}C NMR (101 MHz, D_2O)

Methyl 4-methyl-2,2-diphenyl-1,3-oxazinane-4-carboxylate (3ae)

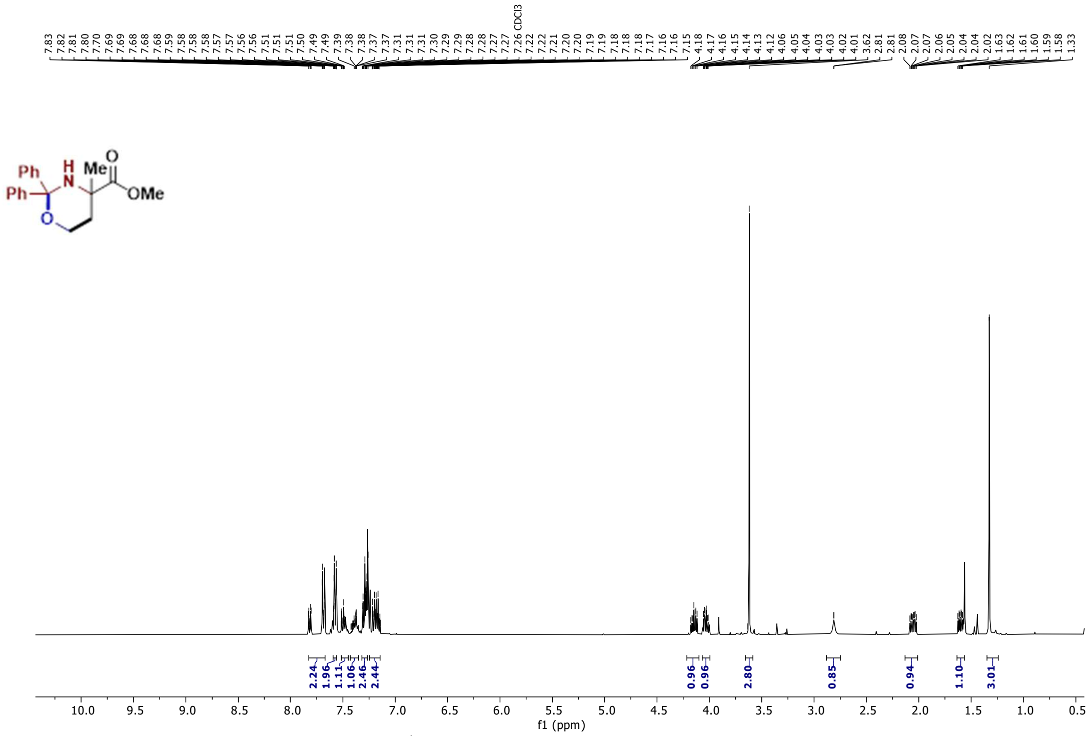

^1H NMR (400 MHz, $CDCl_3$)

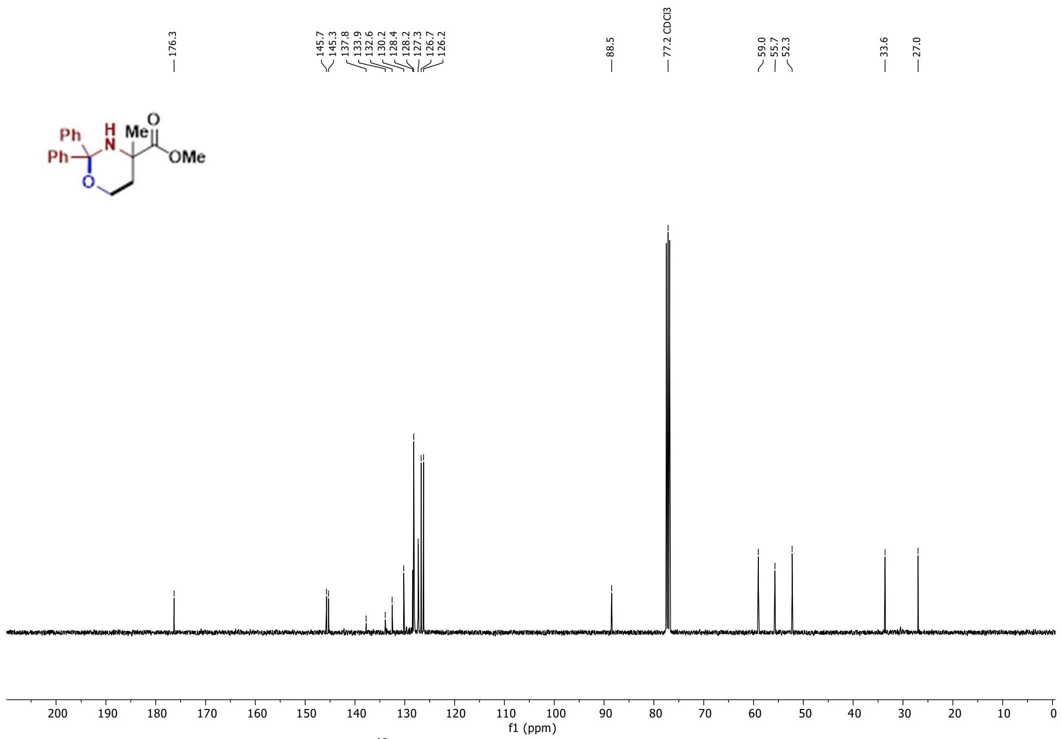

^{13}C NMR (101 MHz, $CDCl_3$)

Di-*tert*-butyl 2-(2-((dimethyl(phenyl)silyl)oxy)ethyl)-2-((diphenylmethylene)amino)malonate (3af)

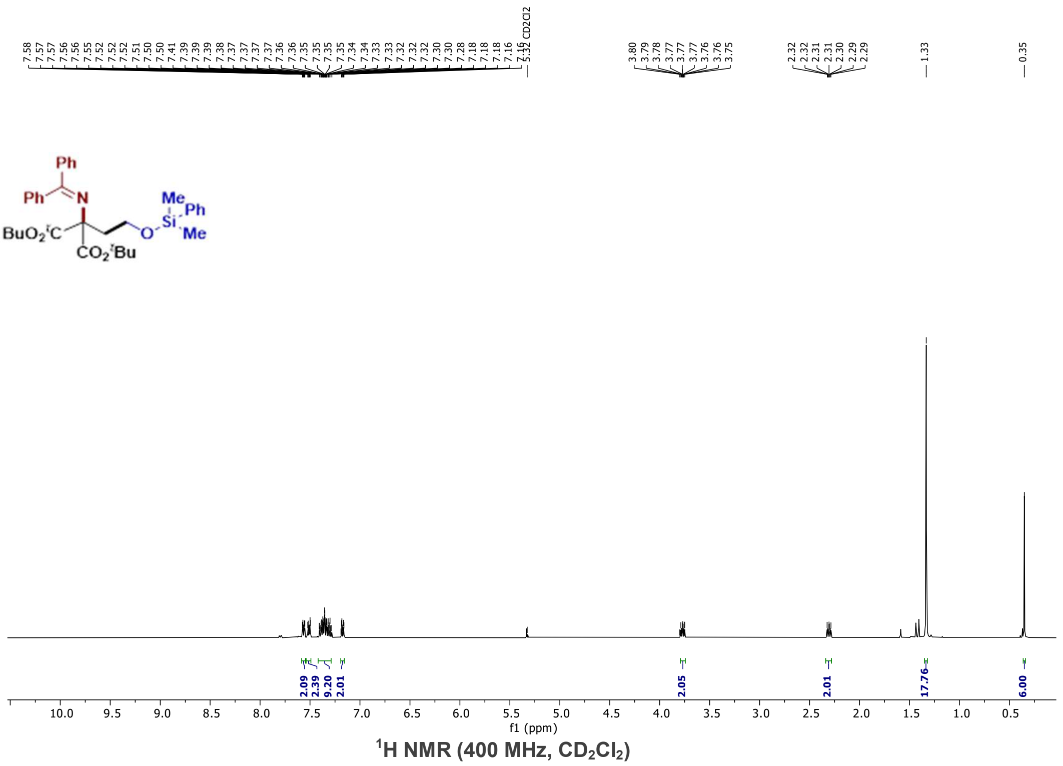

^{1}H NMR (400 MHz, CD_2Cl_2)

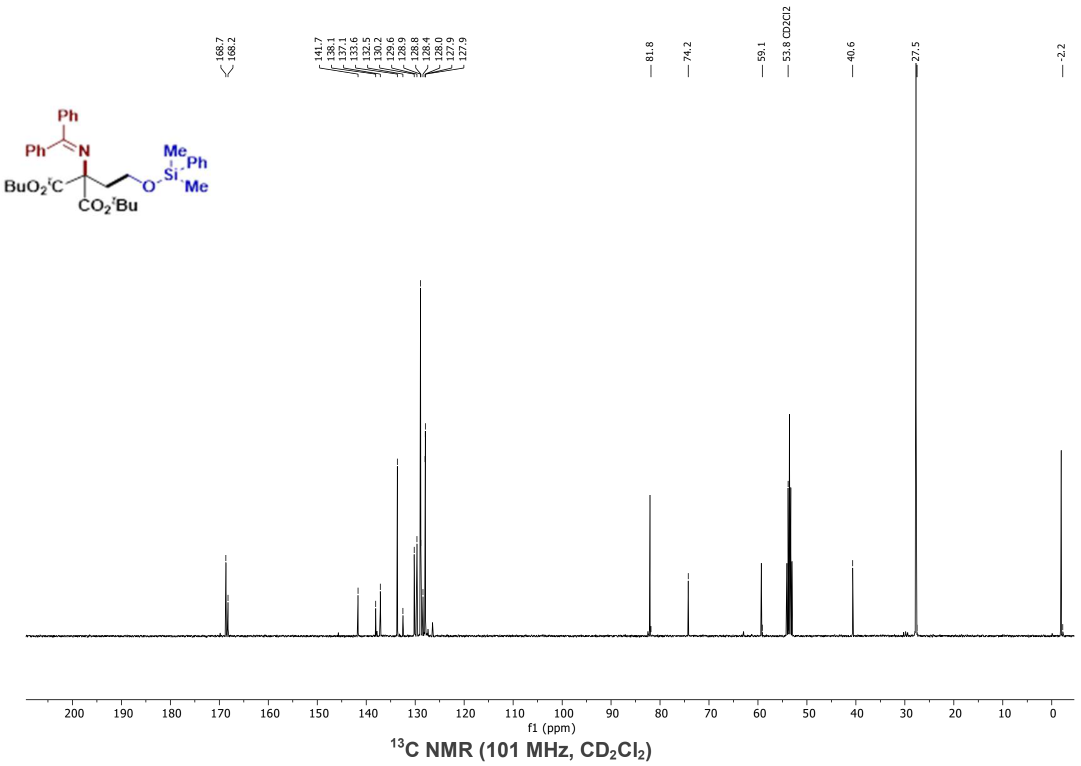

^{13}C NMR (101 MHz, CD_2Cl_2)

2-(2-((Dimethyl(phenyl)silyl)oxy)-1-phenylethyl)-2-((diphenylmethylene)amino)malononitrile (3ag)

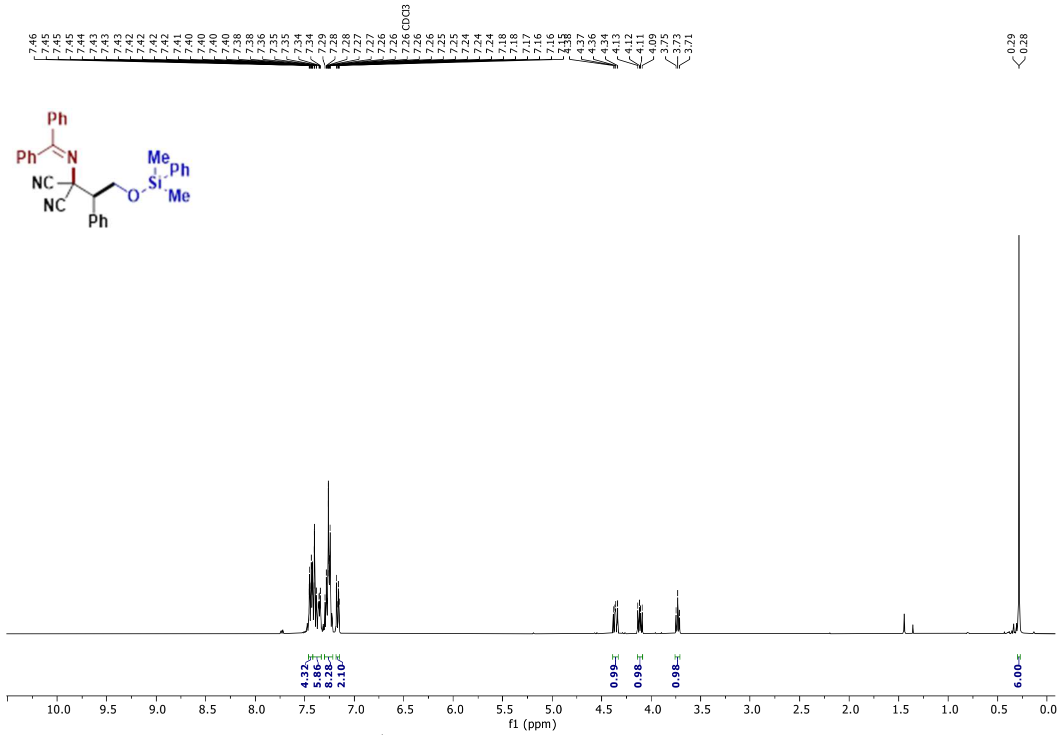

^{1}H NMR (400 MHz, $CDCl_3$)

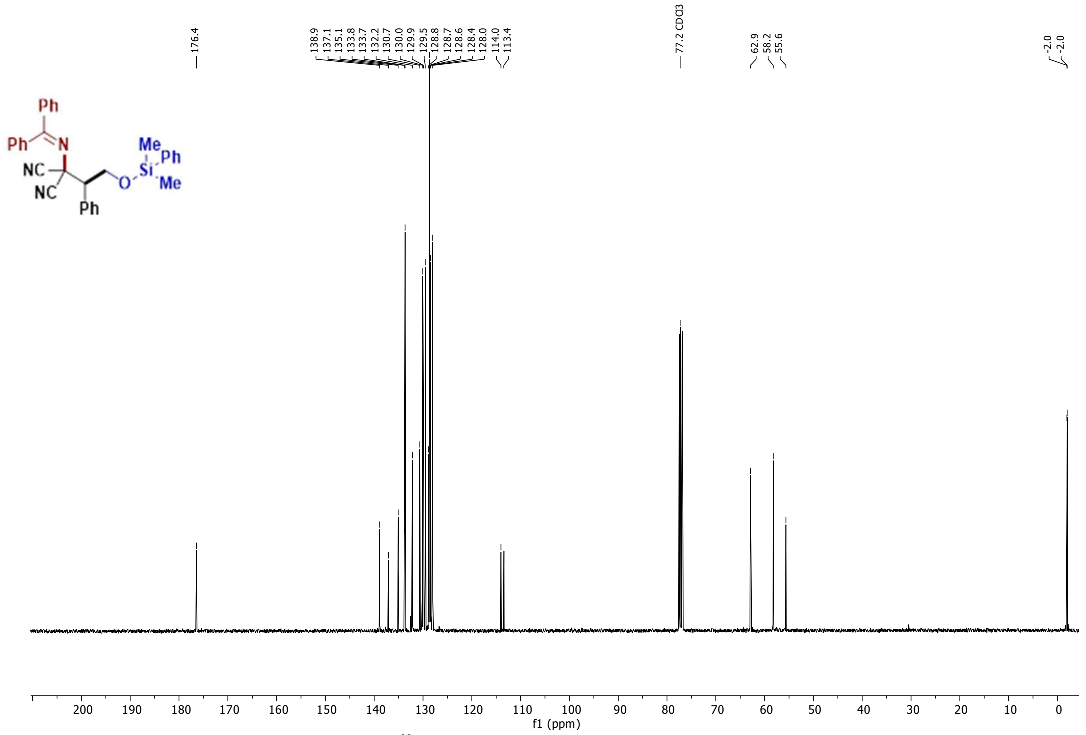

^{13}C NMR (101 MHz, $CDCl_3$)

2-(1-((Dimethyl(phenyl)silyl)oxy)-2-methylpropan-2-yl)-2-((diphenylmethylene)amino)malononitrile (3ah)

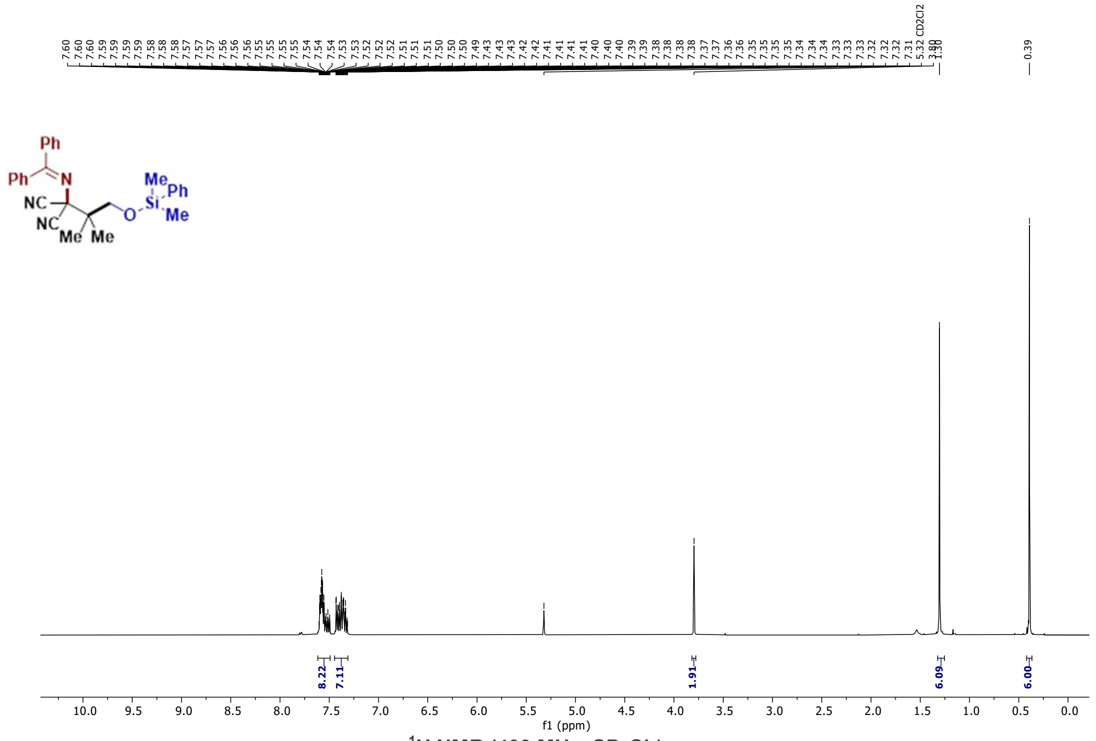

^{1}H NMR (400 MHz, CD_2Cl_2)

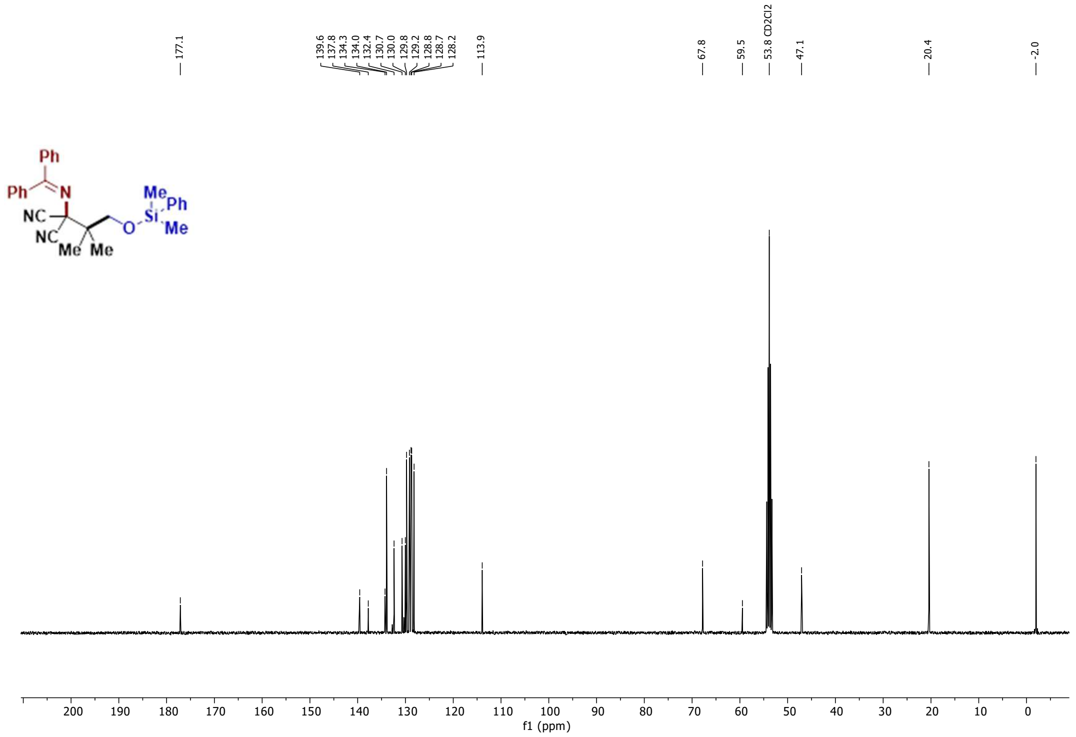

^{13}C NMR (101 MHz, CD_2Cl_2)

3-(Benzhydrylamino)-3,3-diphenylpropan-1-ol (4a)

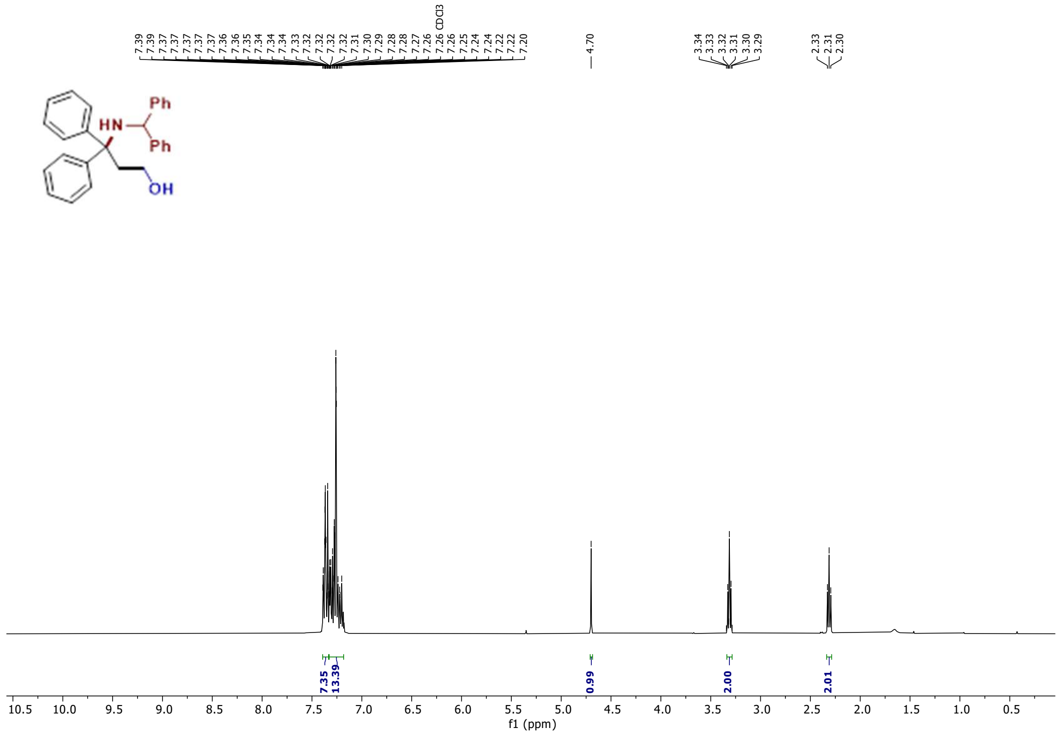

^{1}H NMR (400 MHz, $CDCl_3$)

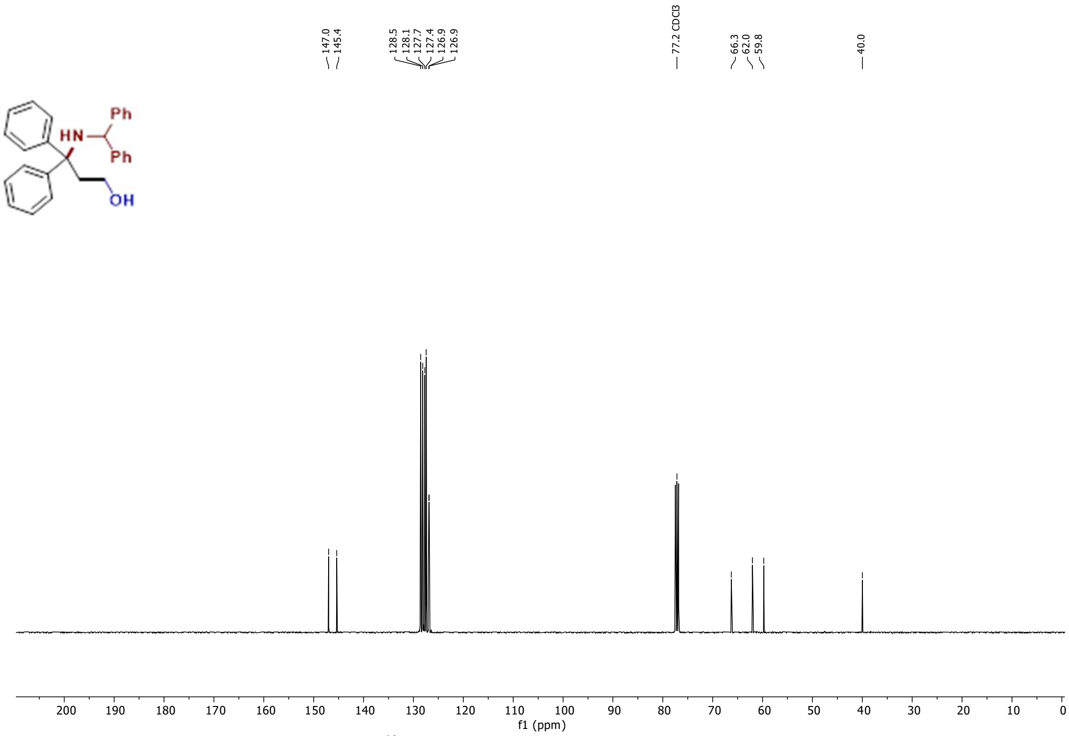

^{13}C NMR (101 MHz, $CDCl_3$)

4,4-Diphenyl-1,3-oxazinan-2-one (4b)

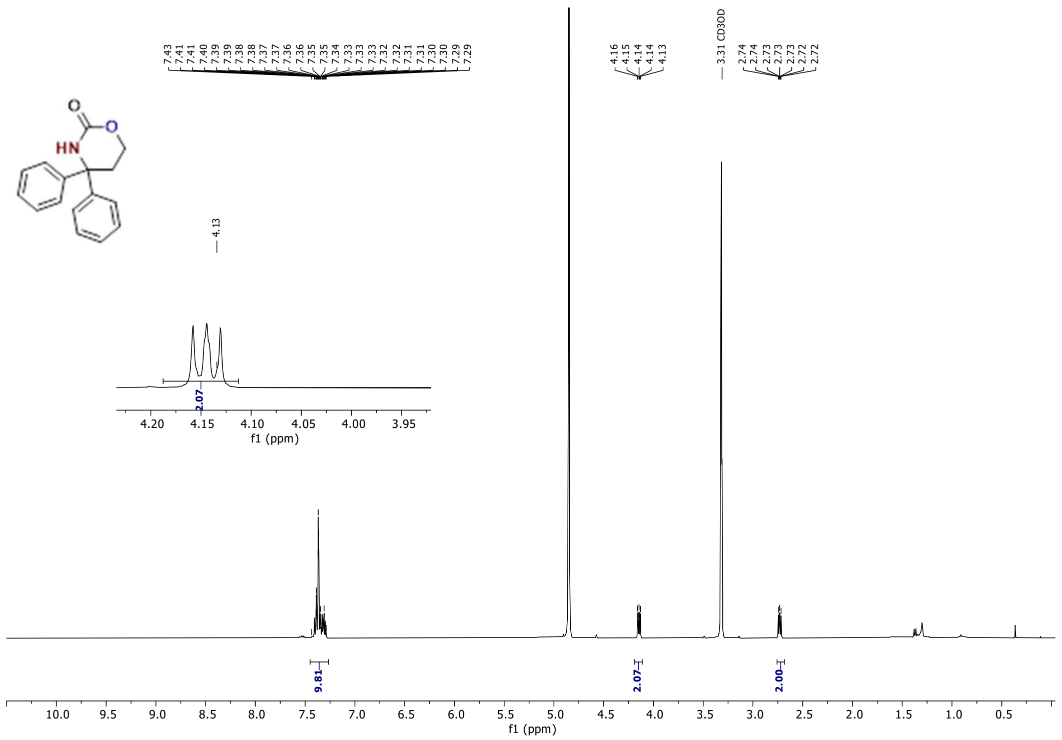

^{1}H NMR (400 MHz, MeOD)

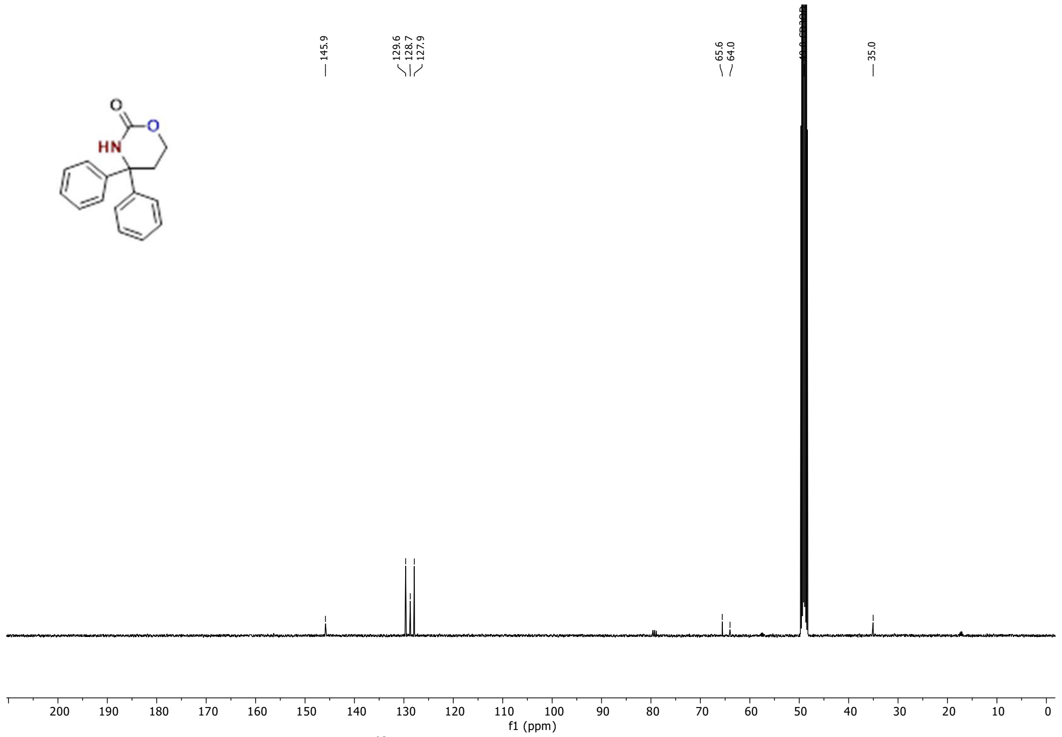

^{13}C NMR (101 MHz, MeOD)

2-(3-Hydroxy-1,1-diphenylpropyl) isoindoline-1,3-dione (4c)

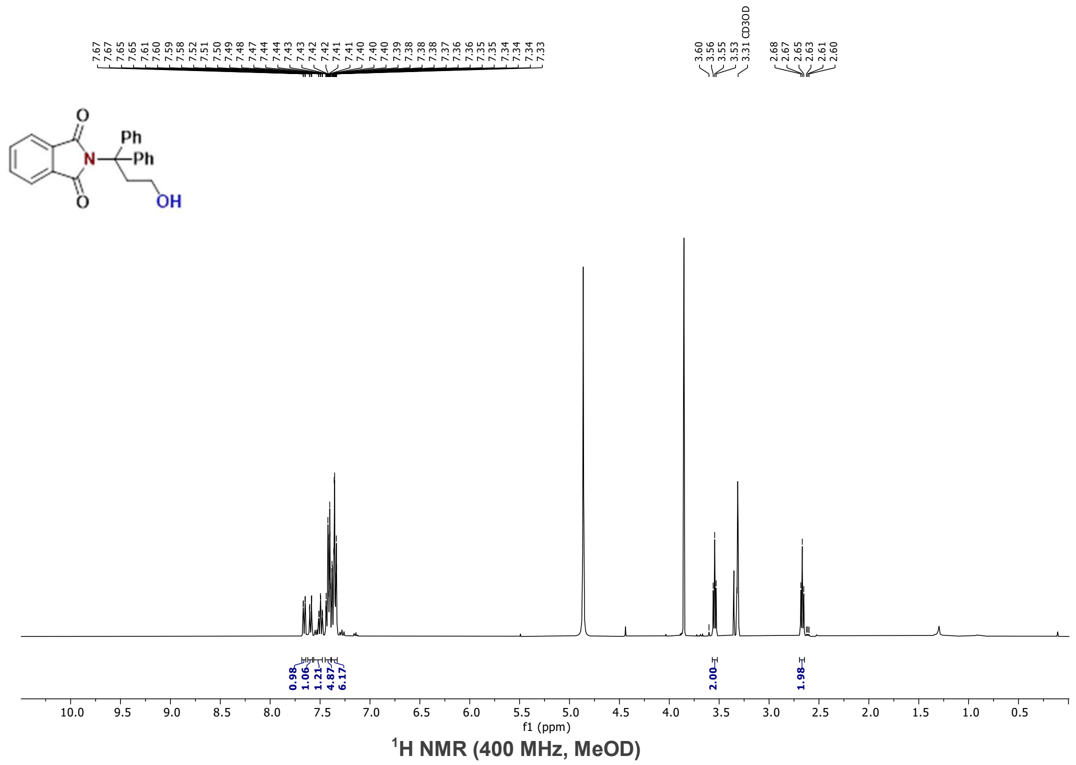

^{1}H NMR (400 MHz, MeOD)

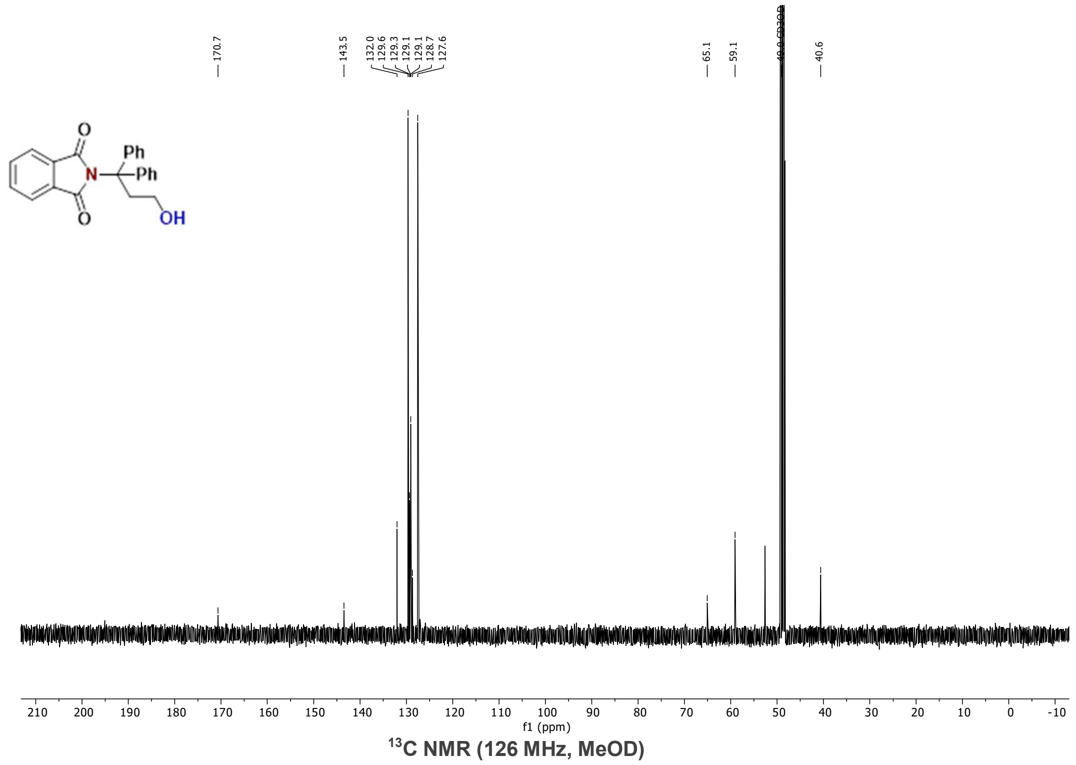

^{13}C NMR (126 MHz, MeOD)

*Note: inseparable hydrogen-bonded MeOH peaks in the spectra, as mentioned in the synthesis section.

(±)Homoserine hydrochloride (4d)

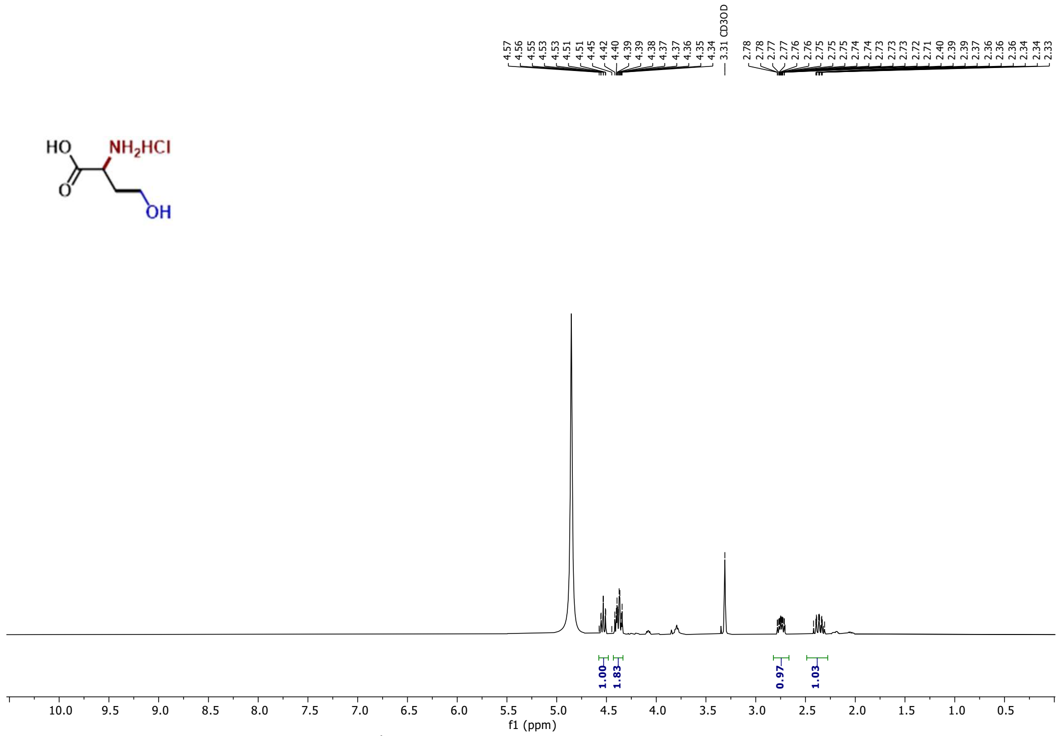

^{1}H NMR (400 MHz, MeOD)

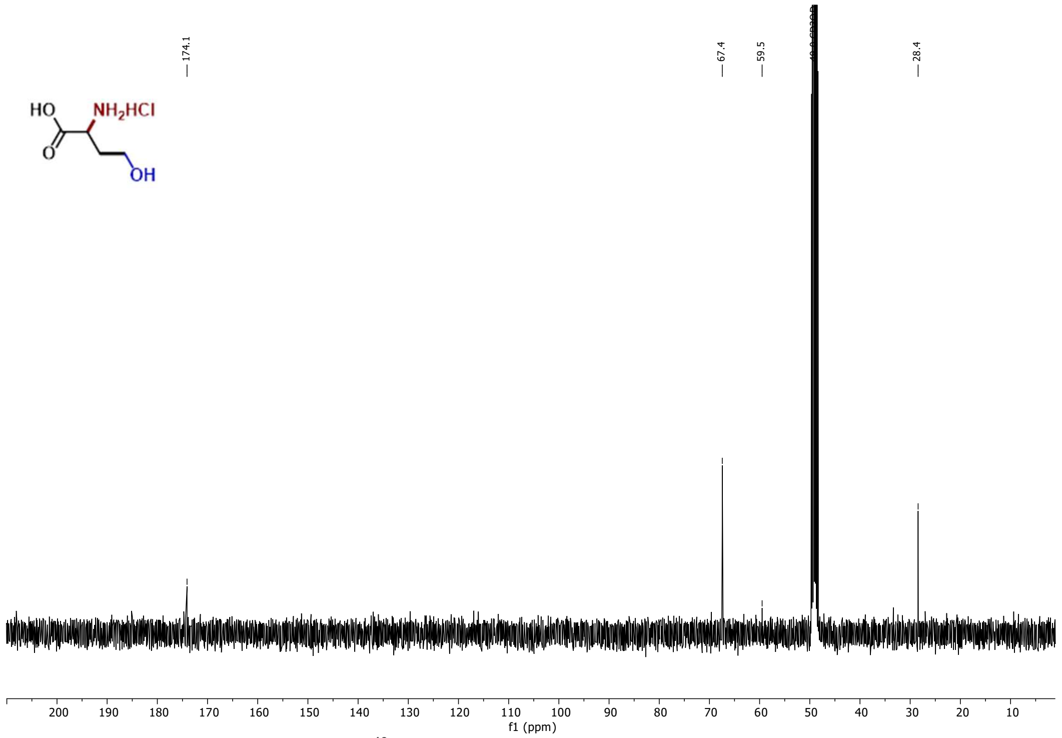

^{13}C NMR (101 MHz, MeOD)

6 References

(1) Kelly, C. B.; Patel, N. R.; Primer, D. N.; Jouffroy, M.; Tellis, J. C.; Molander, G. A. Preparation of visible-light-activated metal complexes and their use in photoredox/nickel dual catalysis. *Nat. Protoc.* **2017**, *12*, 472–492.

(2) Joshi-Pangu, A.; Lévesque, F.; Roth, H. G.; Oliver, S. F.; Campeau, L.-C.; Nicewicz, D.; DiRocco, D. A. Acridinium-Based Photocatalysts: A Sustainable Option in Photoredox Catalysis. *J. Org. Chem.* **2016**, *81*, 7244–7249.

(3) Ye, H.; Ye, Q.; Cheng, D.; Li, X.; Xu, X. The coupling of potassium organotrifluoroborates with Baylis–Hillman derivatives via visible-light photoredox catalysis. *Tetrahedron Lett.* **2018**, *59*, 2046–2049.

(4) Huang, H.-M.; Bellotti, P.; Chen, P.-P.; Houk, K. N.; Glorius, F. Allylic C(sp3)–H arylation of olefins via ternary catalysis. *Nat. Synth.* **2022**, *1*, 59–68.

(5) Singh, A.; Teegardin, K.; Kelly, M.; Prasad, K. S.; Krishnan, S.; Weaver, J. D. Facile synthesis and complete characterization of homoleptic and heteroleptic cyclometalated Iridium(III) complexes for photocatalysis. *J. Organomet. Chem.* **2015**, *776*, 51–59.

(6) Peric Simov, B.; Wuggenig, F.; Mereiter, K.; Andres, H.; France, J.; Schnelli, P.; Hammerschmidt, F. Direct chemical synthesis of chiral methanol of 98% ee and its conversion to (2)H1,(3)Hmethyl tosylate and (2)H1,(3)H-methylmethionine. *J. Am. Chem. Soc.* **2005**, *127*, 13934–13940.

(7) Gao, Y.; Liu, J.; Li, Z.; Guo, T.; Xu, S.; Zhu, H.; Wei, F.; Chen, S.; Gebru, H.; Guo, K. Dichloroimidazolidinedione-Activated Beckmann Rearrangement of Ketoximes for Accessing Amides and Lactams. *J. Org. Chem.* **2018**, *83*, 2040–2049.

(8) Patra, T.; Mukherjee, S.; Ma, J.; Strieth-Kalthoff, F.; Glorius, F. Visible-Light-Photosensitized Aryl and Alkyl Decarboxylative Functionalization Reactions. *Angew. Chem. Int. Ed.* **2019**, *58*, 10514–10520.

(9) Zheng, Y.-Y.; Shen, C.-W.; Zhu, M.-Y.; Zhou, Y.-M.; Li, J.-Q. Identification, Synthesis, and Strategy for the Reduction of Potential Impurities Observed in Dabigatran Etexilate Mesylate Processes. *Org. Process Res. Dev.* **2014**, *18*, 744–750.

(10) Tan, G.; Das, M.; Keum, H.; Bellotti, P.; Daniliuc, C.; Glorius, F. Photochemical single-step synthesis of β-amino acid derivatives from alkenes and (hetero)arenes. *Nat. Chem.* **2022**, *14*, 1174–1184.

(11) Bellotti, P.; Huang, H.-M.; Faber, T.; Laskar, R.; Glorius, F. Catalytic defluorinative ketyl-olefin coupling by halogen-atom transfer. *Chem. Sci.* **2022**, *13*, 7855–7862.

(12) Paulus, F.; Stein, C.; Heusel, C.; Stoffels, T. J.; Daniliuc, C. G.; Glorius, F. Three-Component Photochemical 1,2,5-Trifunctionalizations of Alkenes toward Densely Functionalized Lynchpins. *J. Am. Chem. Soc.* **2023**, *145*, 23814–23823.

(13) Pitzer, L.; Schäfers, F.; Glorius, F. Rapid Assessment of the Reaction-Condition-Based Sensitivity of Chemical Transformations. *Angew. Chem. Int. Ed.* **2019**, *58*, 8572–8576.

(14) Zhang, G.; Favela, D.; Chow, W. L.; Iyer, R. N.; Pell, A. J.; Olson, D. E. Synthesis of Tertiary Amines through Extrusive Alkylation of Carbamates. *Org. Lett.* **2022**, *24*, 6208–6212.

(15) Domeyer, S.; Bjerregaard, M.; Johansson, H.; Sejer Pedersen, D. Exploring endoperoxides as a new entry for the synthesis of branched azasugars. *Beilstein J. Org. Chem.* **2017**, *13*, 644–647.

(16) Bell, J. D.; Murphy, J. A. Recent advances in visible light-activated radical coupling reactions triggered by (i) ruthenium, (ii) iridium and (iii) organic photoredox agents. *Chem. Soc. Rev.* **2021**, *50*, 9540–9685.

(17) Nikitas, N. F.; Gkizis, P. L.; Kokotos, C. G. Thioxanthone: a powerful photocatalyst for organic reactions. *Org. Biomol. Chem.* **2021**, *19*, 5237–5253.

(18) Erchinger, J. E.; Hoogesteger, R.; Laskar, R.; Dutta, S.; Hümpel, C.; Rana, D.; Daniliuc, C. G.; Glorius, F. EnT-Mediated N-S Bond Homolysis of a Bifunctional Reagent Leading to Aliphatic Sulfonyl Fluorides. *J. Am. Chem. Soc.* **2023**, *145*, 2364–2374.

(19) Strieth-Kalthoff, F.; James, M. J.; Teders, M.; Pitzer, L.; Glorius, F. Energy transfer catalysis mediated by visible light: principles, applications, directions. *Chem. Soc. Rev.* **2018**, *47*, 7190–7202.

(20) Zhang, Y.; Chen, J.-J.; Huang, H.-M. Radical Brook Rearrangements: Concept and Recent Developments. *Angew. Chem. Int. Ed.* **2022**, *61*, e202205671.
(21) Kuhn, H. J.; Braslavsky, S. E.; Schmidt, R. Chemical actinometry (IUPAC Technical Report). *Pure Appl. Chem.* **2004**, *76*, 2105–2146.
(22) A new sensitive chemical actinometer - II. Potassium ferrioxalate as a standard chemical actinometer. *Proc. R. Soc. Lond. A* **1956**, *235*, 518–536.
(23) Cismesia, M. A.; Yoon, T. P. Characterizing Chain Processes in Visible Light Photoredox Catalysis. *Chem. Sci.* **2015**, *6*, 5426–5434.
(24) Wegner, E. E.; Adamson, A. W. Photochemistry of Complex Ions. III. Absolute Quantum Yields for the Photolysis of Some Aqueous Chromium(III) Complexes. Chemical Actinometry in the Long Wavelength Visible Region. *J. Am. Chem. Soc.* **1966**, *88*, 394–404.
(25) a) Becke, A. D. Density-functional thermochemistry. III. The role of exact exchange. *J. Chem. Phys.* **1993**, *98*, 5648–5652.; b) Lee, C.; Yang, W.; Parr, R. G. Development of the Colle-Salvetti correlation-energy formula into a functional of the electron density. *Phys. Rev. B Condens. Matter* **1988**, *37*, 785–789.;
(26) a) Ehrlich, S.; Moellmann, J.; Grimme, S. Dispersion-corrected density functional theory for aromatic interactions in complex systems. *Acc. Chem. Res.* **2013**, *46*, 916–926.; b) Grimme, S. Accurate description of van der Waals complexes by density functional theory including empirical corrections. *J. Comput. Chem.* **2004**, *25*, 1463–1473.; c) Grimme, S. Density functional theory with London dispersion corrections. *WIREs Comput. Mol. Sci.* **2011**, *1*, 211–228.; d) Grimme, S.; Antony, J.; Ehrlich, S.; Krieg, H. A consistent and accurate ab initio parametrization of density functional dispersion correction (DFT-D) for the 94 elements H-Pu. *J. Chem. Phys.* **2010**, *132*, 154104.;
(27) Weigend, F.; Ahlrichs, R. Balanced basis sets of split valence, triple zeta valence and quadruple zeta valence quality for H to Rn: Design and assessment of accuracy. *Phys. Chem. Chem. Phys.* **2005**, *7*, 3297–3305.
(28) Weigend, F. Accurate Coulomb-fitting basis sets for H to Rn. *Phys. Chem. Chem. Phys.* **2006**, *8*, 1057–1065.
(29) a) Klamt, A.; Schüürmann, G. COSMO: a new approach to dielectric screening in solvents with explicit expressions for the screening energy and its gradient. *J. Chem. Soc., Perkin Trans. 2* **1993**, 799–805.; b) Cossi, M.; Rega, N.; Scalmani, G.; Barone, V. Energies, structures, and electronic properties of molecules in solution with the C-PCM solvation model. *J. Comput. Chem.* **2003**, *24*, 669–681.; c) Tomasi, J.; Persico, M. Molecular Interactions in Solution: An Overview of Methods Based on Continuous Distributions of the Solvent. *Chem. Rev.* **1994**, *94*, 2027–2094.; d) Andzelm, J.; Kölmel, C.; Klamt, A. Incorporation of solvent effects into density functional calculations of molecular energies and geometries. *J. Chem. Phys.* **1995**, *103*, 9312–9320.; e) Barone, V.; Cossi, M. Quantum Calculation of Molecular Energies and Energy Gradients in Solution by a Conductor Solvent Model. *J. Phys. Chem. A* **1998**, *102*, 1995–2001.;
(30) Gaussian 16, revision C.01.
(31) Université de Sherbrooke, 2020; www.cylview.org.
